# A Ruthenophosphanorcaradiene as a Synthon for an Ambiphilic Metallophosphinidene

**DOI:** 10.1021/jacs.3c14779

**Published:** 2024-02-09

**Authors:** Tyler G. Saint-Denis, T. Alexander Wheeler, Qingchuan Chen, Gábor Balázs, Nicholas S. Settineri, Manfred Scheer, T. Don Tilley

**Affiliations:** † Department of Chemistry, University of California, Berkeley, Berkeley, California 94720-1460, United States; ‡ Department of Inorganic Chemistry, University of Regensburg, 93040 Regensburg, Germany

## Abstract

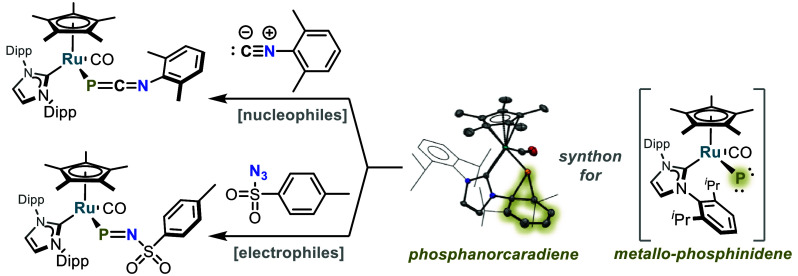

Reaction of the ruthenium carbene complex Cp*(IPr)RuCl (**1**) (IPr = 1,3-bis(Dipp)imidazol-2-ylidene; Dipp = 2,6-diisopropylphenyl) with sodium phosphaethynolate (NaOCP) led to intramolecular dearomatization of one of the Dipp substituents on the Ru-bound carbene to afford a Ru-bound phosphanorcaradiene, **2**. Computations by DFT reveal a transition state characterized by a concerted process whereby CO migrates to the Ru center as the P atom adds to the π system of the aryl group. The phosphanorcaradiene possesses ambiphilic properties and reacts with both nucleophilic and electrophilic substrates, resulting in rearomatization of the ligand aryl group with net P atom transfer to give several unusual metal-bound, P-containing main-group moieties. These new complexes include a metallo-1-phospha-3-azaallene (Ru—P=C=NR), a metalloiminophosphanide (Ru—P=N—R), and a metallophosphaformazan (Ru—P(=N—N=CPh_2_)_2_). Reaction of **2** with the carbene 2,3,4,5-tetramethylimidazol-2-ylidene (IMe_4_) produced the corresponding phosphaalkene DippP=IMe_4_.

Phosphinidenes possess six valence electrons and may exist in singlet or triplet ground states.^1^ These species are therefore regarded as analogues of carbenes and, more broadly, group 14 tetrylenes (R_2_E, E = C, Si, Ge, Sn, Pb). However, the monovalency of phosphinidenes (R—P) leads to higher reactivity and dramatically complicates strategies to obtain isolable examples.^1−6^ To date, there is only one report of an isolable, persistent phosphinidene, reported by the group of Bertrand.^7,8^ Given the highly reactive nature of phosphinidenes, they have been studied as transient intermediates generated from suitable precursors and trapped by added reagents. For example, the groups of Fritz and Protasiewicz have investigated phospha-Wittig compounds (**A**, Figure 1A) for the generation of phosphinidenes.^9−12^ More recently, the Cummins group has investigated phosphinidene transfer reactions using dibenzo-7λ^3^-phosphanorbornadienes (**B**, Figure 1A), which expel the phosphinidene fragment with formation of anthracene.^13−20^ The latter strategy has been used more generally for the generation of low-valent main-group entities.^21,22^

**Figure 1:**
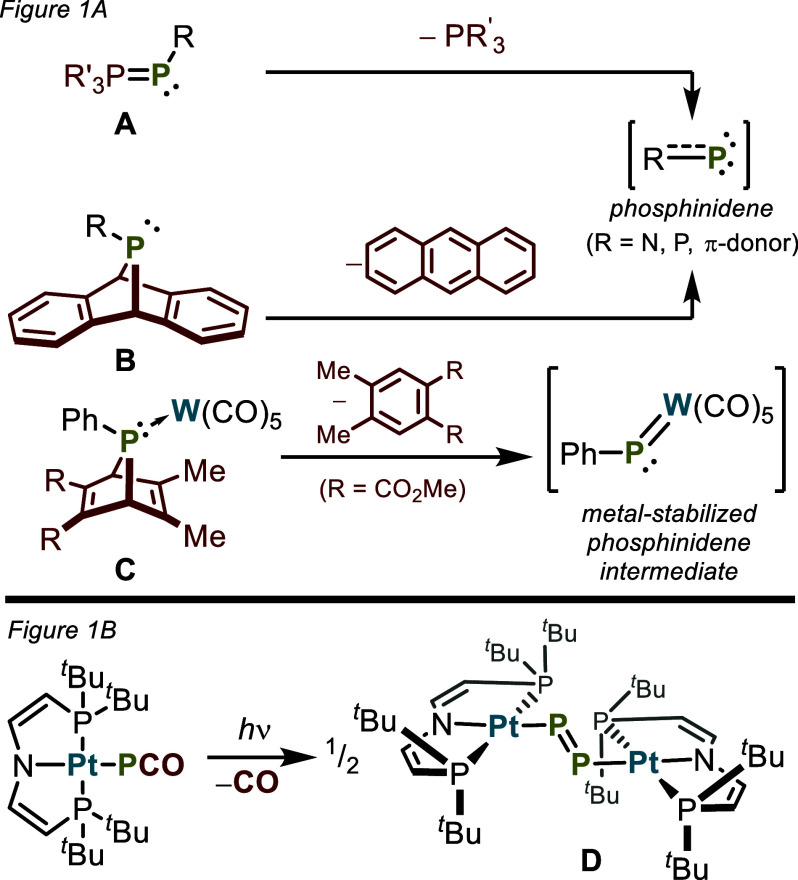
Synthetic strategies for the generation of transient metallophosphinidenes.

A strategy for stabilizing low-coordinate main-group species involves their coordination to transition metal centers, as exemplified by numerous multiply bonded terminal phosphido complexes, L_*n*_M≡P.^23−36^ Similar considerations apply to the stabilization of phosphinidene complexes (L_*n*_M=P—R), which can be generated from the cheleotropic elimination of an organic fragment from the corresponding phosphine complex. Representative examples of metal phosphinidenes include (CO)_5_W=PPh, generated transiently from a metal-coordinated phosphanorbornadiene via expulsion of a substituted benzene, reported by Mathey and co-workers (**C**, Figure 1A), and the isolable metal phosphinidene Cp_2_W=PMes* reported by Lappert and co-workers (Cp = C_5_H_5_, Mes* = 2,4,6-^t^Bu_3_C_6_H_2_).^1,37−43^ A related type of metal-stabilized, formally electron-deficient metal–phosphorus species is represented by the hitherto unknown *metallo*phosphinidenes (L_*n*_M—P̈:), which should result when M–P multiple bonding is unfavorable (as with late transition metals). The photochemical generation of such a complex as a transient species likely occurred from photolysis of (PNP)Pt—PCO to give (PNP)Pt—P=P—Pt(PNP) (**D**, Figure 1B), as reported by Schneider and co-workers.^44^

The Tilley group has employed 16-electron, yet inherently electron-rich, piano-stool complexes Cp*(L)MX (Cp* = η^5^-C_5_Me_5_; L = phosphine, NHC; M = Ru, Os; X = halide) for the synthesis of metal complexes containing reactive group 14 intermediates. This strategy, used to obtain silylene, silene, germylene, stannylene, and metallostannylene complexes, involves substitution of halide for an anionic species poised to undergo migratory cleavage, driven by the metal center’s propensity to achieve an 18-electron configuration (Scheme 1).^45−51^ Given this background, it was of interest to explore the possibility of intramolecular cleavage of phosphaethynolate (PCO^–^, readily available as NaOCP) in a Cp*(L)M—PCO complex to introduce the electron-withdrawing CO ligand and produce a metallophosphinidene (Scheme 1, A—B^–^ = OC—P^–^).^52,53^

**Scheme 1:**
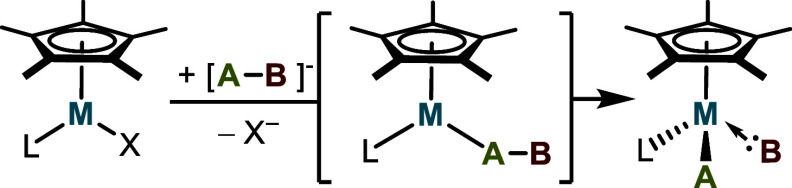
Synthetic Logic for the Preparation of Transition-Metal-Stabilized Low-Valent Main-Group Fragments by Metal (Fe, Ru, Os)-Mediated Fragmention of Anionic [A—B]^−^

Exposure of dark-purple Cp*(IPr)RuCl (**1**) (IPr = 1,3-bis(Dipp)imidazol-2-ylidene; Dipp = 2,6-diisopropylphenyl) to sodium phosphaethynolate (NaOCP) in THF led to the formation of a bright-yellow solution within 15 min at room temperature.^54^ Workup and NMR spectroscopic analysis revealed near-quantitative conversion to a product possessing an NHC ligand with different *N*-substituents. Moreover, ^1^H NMR spectroscopy revealed the presence of an ABC spin system between 6.51 and 5.71 ppm consistent with a *cis*-diene structural motif. These spectroscopic features suggested dearomatization of one of the Dipp groups of IPr. The IR spectrum contains a stretch at 1902 cm^–1^, and ^13^C{^1^H} NMR spectroscopy reveals a resonance at 210 ppm, consistent with a transition-metal-bound CO ligand. The molecular structure of **2**, determined by X-ray crystallography, corresponds to a metallophosphanorcaradiene with a Ru-bound CO ligand (Scheme 2). Compound **2** represents the second isolated phosphanorcaradiene, and the only one to feature a covalent transition metal–phosphorus bond.^55^

**Scheme 2:**
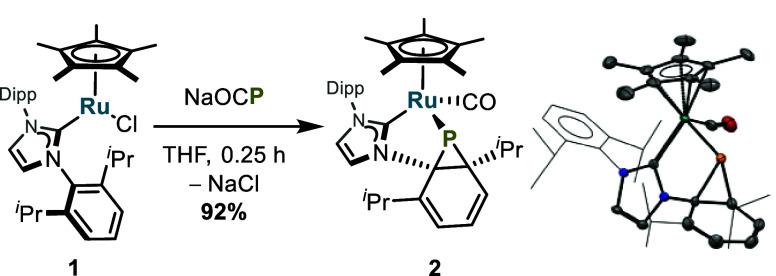
Reaction of **1** with NaOCP to Form Metallophosphanorcaradiene **2**

Interestingly, a phosphine analogue of **1**, Cp*(^i^Pr_3_P)RuCl, reacted with NaOCP in THF to give a complex mixture of products, and the reaction proceeded similarly in the presence of various unsaturated compounds added as potential traps for a metallophosphinidene (superstoichiometric amounts of olefins, dienes, anthracene, *etc.*; see the Supporting Information for details). Exposure of **1** to NaOCP in the presence of an excess of the same potential traps (*vide supra*) gave only **2**. These results indicate that the trapping of an incipient metallophosphinidene is highly favored by intramolecular transfer of phosphorus to a π-donor within the metal complex. This is consistent with DFT computations at the ωB97X-D4/def2-TZVPPD/CPM(THF) level of theory; after metathetical exchange between the Ru-bound chloride of Cp*(IPr)RuCl and the phosphaethynolate anion, a putative intermediate Cp*(IPr)RuPCO is formed, which converts to **2** with CO migration and Dipp dearomatization. This process proceeds in a concerted but somewhat asynchronous manner involving migration of CO to Ru prior to P–C bond formation, with no discrete intermediate (Figure 2). The barrier for this reaction is only 8.8 kcal mol^–1^, and the product **2** is 26.4 kcal mol^–1^ lower in energy than the putative Cp*(IPr)RuPCO, likely due to the thermodynamic favorability of the bonding between the electron-rich Ru and the CO ligand. Attempts to observe and isolate Cp*(L)RuPCO are currently underway.

**Figure 2:**
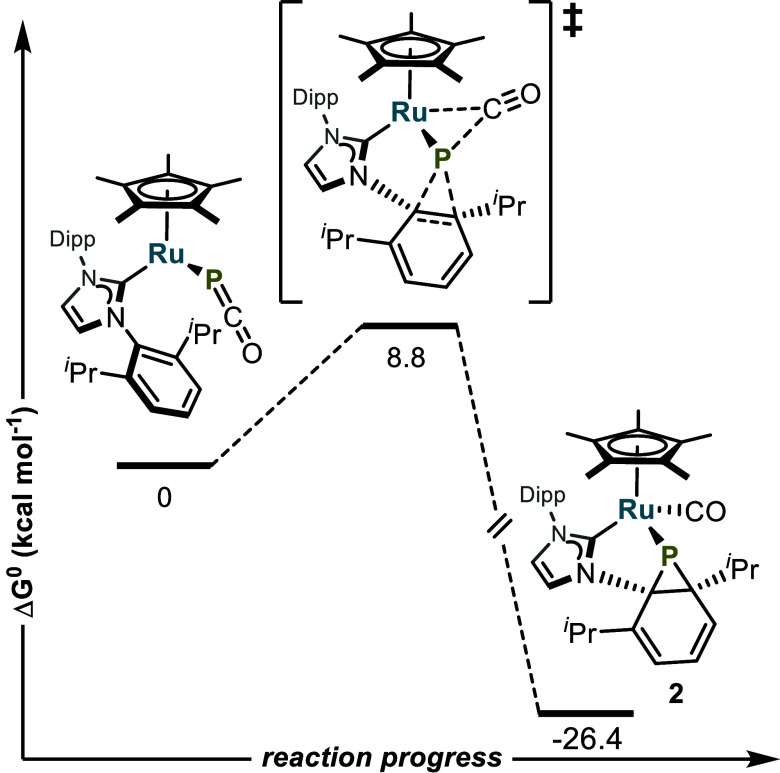
Proposed mechanism for the formation of **2** from putative Cp*(IPr)RuPCO by DFT computations at the ωB97X-D4/def2-TZVPPD CPM(THF) level of theory.

Reaction of the nucleophile xylyl (2,6-Me_2_C_6_H_3_) isocyanide with **2** at 70 °C over 72 h led to the formation of η^1^-1-phospha-3-azaallene **3** through rearomatization of the Dipp moiety and formal P atom transfer to xylyl isocyanide (Scheme 3 and Figure 3). Compound **3** possesses a ^31^P NMR resonance of −166 ppm, and X-ray crystallography revealed that the P=C=NR fragment is linear and cumulene-like (P1–C1 = 1.640(7) Å versus ∑*r*_cov_ = 1.80 Å; C1–N3 = 1.231(9) Å versus ∑*r*_cov_ = 1.51 Å; P1–C1–N1 angle = 173.1(5)°).^56^ The infrared spectrum contains a strong band at 1785 cm^–1^, corresponding to a P=C=N stretch; however, in contrast to reported P=C=N stretches of organo 1-phospha-3-azaallenes, such as Mes*—P=C=N—^*t*^Bu (1885 cm^–1^; Mes* = 2,4,6-^*t*^Bu_3_C_6_H_2_), the P=C=N stretch of compound **3** is significantly lower frequency, likely due to Ru—P bonding and polarization of the Ru—P bond.^57^ Several non-metal-containing 1-phospha-3-azaallene compounds have been reported, as well as Nb- and Ta-bound η^2^-(C,N)-1-phospha-3-azaallene complexes. In this context, compound **3** is unique in that the PCN motif is terminally bound only through the P atom.^7,8,57−61^

**Scheme 3:**
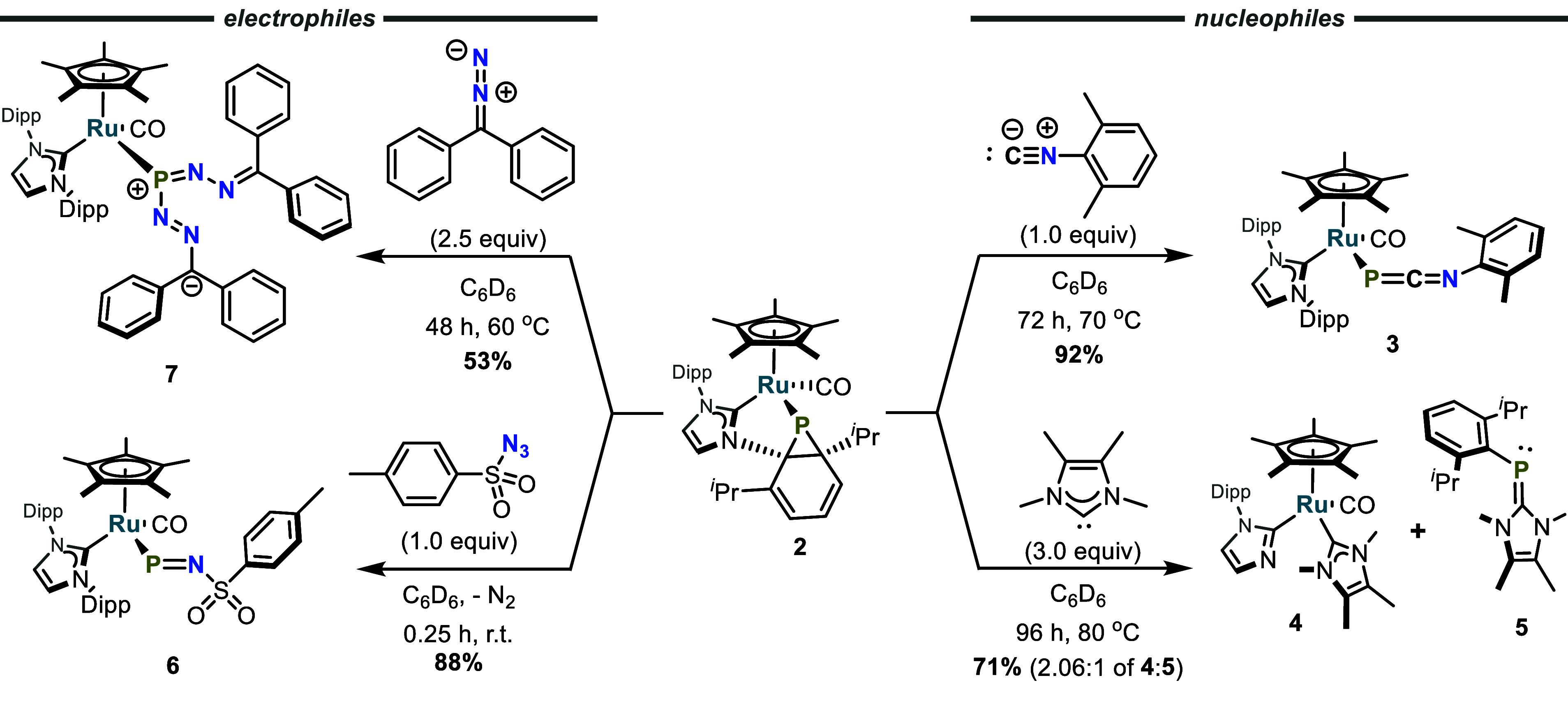
Reactions of Electrophiles and Nucleophiles with **2**

**Figure 3:**
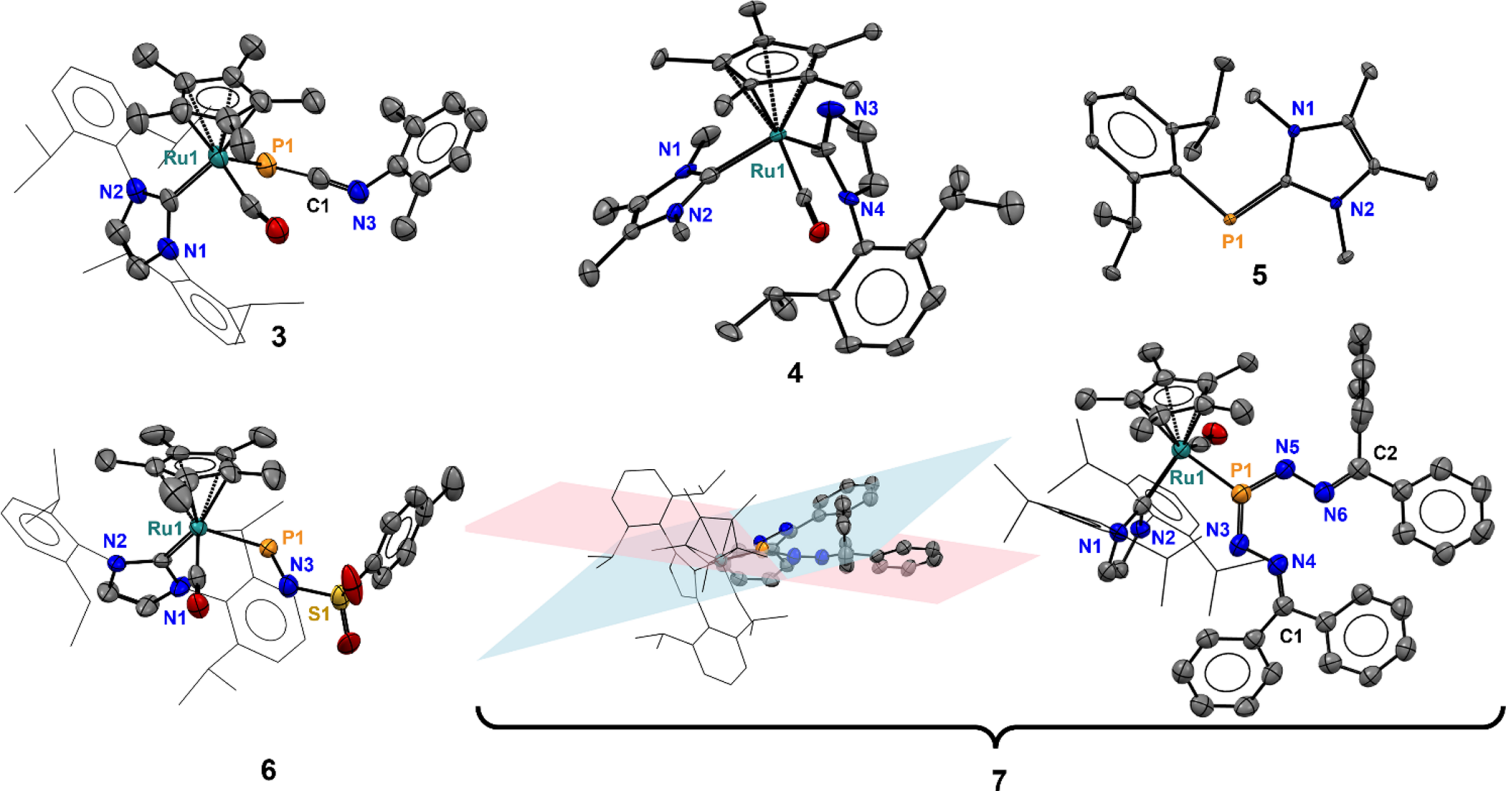
Solid-state structures of compounds **3** to **7**, with 50% probability thermal ellipsoids shown. Hydrogen atoms have been omitted and some ligand frameworks are shown in wireframe for clarity. For the intersecting planes of **7**, the blue plane is defined by P1, N5, N6, and C2, and the pink plane is defined by P1, N3, N4, and C1.

The reaction of **2** with an excess of 2,3,4,5-tetramethylimidazol-2-ylidene (IMe_4_) in toluene resulted in the formation of Ru—imidazol-2-yl (**4**) and the carbene-stabilized Dipp-phosphinidene (**5**) in 72% yield (Scheme 3 and Figure 3). Compound **5** was previously reported by Hering-Junghans,^62^ and all spectroscopic data from the isolated compound match those previously reported, while X-ray crystallography verifies the reported structural assignment (Scheme 3 and Figure 3).

Thus, nucleophilic additions to **2** have been observed to proceed by two different processes. The isocyanide attacks **2** to cleave the P–C bonds of the phosphanorcaradiene motif to rearomatize the Dipp group and form **3**, while IMe_4_ reacts to cleave a N–C bond to give **4** and **5**. The latter reaction is envisioned to proceed by attack onto the P atom to generate an intermediate, Cp*(IPr)(CO)Ru—P=IMe_4_, which undergoes P–C coupling to give **5** with trapping of the Ru product by a second equivalent of IMe_4_ (see Figure S1 for further mechanistic speculation). Note that a related intramolecular N–C bond cleavage mediated by a transient phosphinidene (generated from a carbene-stabilized phosphirene) has been reported by Stephan.^17^

The formations of **3**, **4**, and **5** are consistent with the reported electrophilic nature of phosphinidenes/phosphinidene equivalents.^7,8,20^ The possibility of **2** also possessing nucleophilic character at P is suggested by the pyramidal nature of the P atom and the presumed presence of a stereochemically active lone pair (Scheme 2). Indeed, **2** has been found to exhibit nucleophilic character. It reacted with tosyl azide at room temperature over 15 min to form **6**, which possesses a P–N double bond (P1–N1 = 1.604(3) Å versus ∑*r*_cov_ = 1.78 Å; Scheme 3 and Figure 3), in 88% yield.^56^ In contrast to the Staudinger reaction, in which azides oxidize trivalent phosphines to form pentavalent iminophosphiranes, the P atom of **6** remains trivalent due to the dissociation and rearomatization of the Dipp moiety. The P=NR fragment of **6** may be regarded as an iminophosphanide, and IR spectroscopy reveals the presence of a CO stretch at 1945 cm^–1^ as well as an intense stretch at 1140 cm^–1^, which is in good agreement with the theoretical P=N stretch of 1100 cm^–1^ calculated at the PBE0/def2-TZVP level of theory (see Figures S3 and S4 and the Supporting Information). Compound **6** is structurally similar to Cummins’ iminophosphenium complex (NRAr)_3_Mo—P≡N—Mes (R = C(CD_3_)_2_CH_3_, Ar = 3,5-Me_2_C_6_H_3_, Mes = 2,4,6-Me_3_C_6_H_2_); however, whereas the iminophosphenium is linear (Mo–P–N angle of 179°), compound **6** is bent (Ru1–P1–N3 angle of 113.0(1)°).^63,64^ Cummins has noted the isolobal analogy between the linear iminophosphenium ligand, [P≡NR^+^], and the nitrosyl cation, [N=O^+^], and in this context the bent iminophosphanide [P=NR^—^] is analogous to the bent nitrosyl anionic ligand, [N=O^–^].^65^

Exposure of **2** to an excess of diphenyldiazomethane at 60 °C led to formation of the Ru–(*E*,*E*)-phosphaformazan complex **7** (Scheme 3 and Figure 3). Presumably, **7** is formed via a transient Ru—iminophosphanide (Ru—P=N—N=CPh_2_), which then reacts with a second equivalent of diphenyldiazomethane. The solid-state structure of **7** reveals that the P–N bond distances of **7** (1.598(7) Å; Figure 3) are equivalent and shorter than the sum of covalent radii (1.78 Å).^56^ Also, the N–N bond distances in **7**, 1.398(8) and 1.383(7) Å, are shorter than the sum of covalent radii (1.46 Å).^56^ Interestingly, the atoms of the N–N–P–N–N motif do not reside in a single plane, but the planes that define P1–N3–N4–C1 (pink) and P1–N5–N6–C2 (blue) intersect with a dihedral angle of 25.20° (Figure 3). This suggests a lack of electronic delocalization in the phosphaformazan unit, even though P–N and N–N multiple-bond character is implicated by the observed bond lengths (see the Supporting Information). The IR spectrum of **7** contains a CO stretch at 1926 cm^–1^ as well as an intense band at 1128 cm^–1^ which is consistent with the P=N stretch observed in **6** (*vide supra*). Though the —P(=NN=CRR′)_2_ fragment of **7** seems to be unprecedented, a related bis(hydrazonato) ligand η^1^-Cp*P(NHN=CPh_2_)_2_ has been characterized in a tungsten complex.^66^

In summary, a ruthenophosphanorcaradiene, formed by reaction of **1** with NaOCP, serves as a synthetic equivalent for the metallophosphinidene Cp*(IPr)(CO)Ru—P. It is apparent that intramolecular interactions play a significant role in the stabilization of **2**, which forms via cycloaddition of the metal-bound P atom to the Dipp group of the NHC ligand. Interestingly, reactions of **2** reveal ambiphilic properties of the P atom and formal P atom transfer to both nucleophiles and electrophiles. The detailed mechanisms for formation of products **3**–**7** as well as the further potential of **2** as a metallophosphinidene synthon are under investigation.

## Supporting Information Available

Synthetic details, crystallographic data, full characterization data (e.g., NMR, IR, EA data, etc.), and computational details (PDF)
