# Supplementary material for: A Ruthenophosphanorcaradiene as a Synthon for an Ambiphilic Metallophosphinidene
Source: J Am Chem Soc. 2024 Feb 9;146(7):4369–74. doi: 10.1021/jacs.3c14779 (PMC10885142; doi:10.1021/jacs.3c14779)
Supplement: Supplementary file 1 — ja3c14779_si_001.pdf [file ja3c14779_si_001.pdf]

SUPPLEMENTARY INFORMATION FOR

A Rutheno-Phosphanorcaradiene as a Synthon for an Ambiphilic Metallo-Phosphinidene

Tyler G. Saint-Denis,[1] T. Alexander Wheeler,[1] Qingchuan Chen,[1] Gábor Balázs,[2] Nicholas S. Settineri,[1] Manfred Scheer,[2,*] and T. Don Tilley[1,*]

[1]Department of Chemistry, University of California, Berkeley, Berkeley, CA 94720-1460, USA

[2]Department of Inorganic Chemistry, University of Regensburg, Regensburg, 93040, Germany

Contents

General methods: 2

Synthesis of compounds: 3

Synthesis of phosphanorcaradiene (2) 3

Synthesis of phosphaazaallene (3) 4

Synthesis of Ru-imidazol-2-yl (4) and Dipp-P=(IMe_4) (5) 5

Proposed mechanism for formation of 4 and 5 from 2 and IMe4: 8

Synthesis of Iminophosphanide (6) 9

Synthesis of Phosphaformazan (7) 10

X-Ray Crystallography: 13

Phosphanorcaradiene (2) 13

Phosphaazaallene (3) 29

Ru-imidazol-2-yl (4) 67

DippP=(IMe_4) (5) 154

Iminophosphanide (6) 162

Phosphaformazan (7) 183

DFT 204

Reaction Mechanism for the formation of 2 204

Theoretical versus experimental IR spectroscopy of 6 213

NMR spectra of compounds: 218

Phosphanorcaradiene (2) 218

Phosphaazaallene (3) 220

Ru-imidazol-2-yl (4) and DippP=(IMe_4) (5) 222

Iminophosphanide (6) 224

Phosphaformazan (7) 226

References: 227

Notes: 228

General methods: All manipulations were carried out using standard Schlenk techniques or in inert atmosphere gloveboxes filled with dry dinitrogen. Solvents were stored over molecular sieves (3 Å) after collection from a JC Meyers Phoenix solvent purification system. Benzene-d_6 was degassed with three freeze-pump-thaw cycles and stored over activated molecular sieves (3 Å) for 1 d prior to use.

Reagents: Previously reported compounds and starting materials were prepared according to literature methods: Cp*(IPr)RuCl,[1] NaOCP,[2] N_2CPh_2,[3] IMe_4.[4] Xylyl isocyanide and Tosyl isocyanide were purchased from Sigma-Aldrich.

NMR spectroscopy: NMR spectra were recorded at the UC Berkeley College of Chemistry NMR facilities on Bruker Avance 400, 500, and 600 MHz spectrometers, and spectra were referenced to solvent residual signals (^{1}H, ^{13}C), or to external references (^{31}P). Unless otherwise indicated, all NMR spectra are reported at 292 K. Resonances obscured by the solvent residual signal(s) are not reported. The following abbreviations are used: br = broad; m = multiplet; s = singlet; d = doublet; t = triplet; h = heptet.

IR spectroscopy: IR spectra were collected on a Thermo Scientific Nicolet iS10 FTIR spectrometer as KBr pellets, unless otherwise noted. Abbreviations for IR spectroscopy: br, broad; s, sharp; m, medium; w, weak.

Elemental analysis: Microanalyses were conducted by Dr. Elena Kreimer at the UC Berkeley College of Chemistry Microanalytical Facility.

Synthesis of compounds:

Synthesis of phosphanorcaradiene (2)

In a glovebox Cp*(IPr)RuCl (0.205 g; 0.310 mmol) was loaded into a 20 mL vial with a magnetic stir bar, dissolved in tetrahydrofuran (1 mL) and stirred to produce a dark purple solution. To a separate 20 mL vial NaOCP (0.0816 g; 1.02 mmol, 3.3 equiv) was loaded and dissolved in 0.5 mL THF to produce a pale tan solution. The THF solution of NaOCP was then added dropwise to the rapidly stirring THF solution of Cp*(IPr)RuCl over 1 min; this resulted in a rapid color change from dark purple to bright yellow. Remaining amounts of NaOCP were dissolved in 0.5 mL THF and added to the reaction mixture. After stirring for 10 minutes all THF was removed in vacuo to afford a yellow orange foam, which was extracted with pentane (3 X 2 mL) and filtered over a celite plug (0.5 x 2 cm) into a new 20 mL vial. The yellow solution was concentrated to 0.5 mL and maintained at –35 °C for 12 h, affording yellow crystalline material. The pentane supernatant was removed and placed in an additional 20 mL vial, which was again cooled to –35 °C for 12 h affording a second crop of yellow crystalline material, the supernatant of which was discarded. The two crops were combined, affording 0.194 g (0.285 mol; 92% yield) of an analytically pure green crystalline material. X-ray quality crystals were grown from a saturated pentane solution cooled to –35 °C.

Characterization data:

^{1}H NMR (400 MHz, Benzene-d_6) δ 7.21 – 7.13 (Dipp C—H bonds overlapped with solvent signal), 7.02 (m, 2H, Dipp C—H bonds), 6.80 (d, J = 2.0 Hz, 1H, carbene methine N=C(**H**)-C(H)=N), 6.51 (d, J = 9.6 Hz, 1H, phosphanorcaradiene =C(**H**)-C(H)=C(H)-), 6.39 (d, J = 2.0 Hz, 1H, carbene methine N=C(**H**)-C(H)=N), 6.06 (dd J = 9.6, 6.0 Hz, 1H, phosphanorcaradiene =C(H)-C(**H**)=C(H)-), 5.71 (d, J = 6.0 Hz, 1H, phosphanorcaradiene =C(H)-C(H)=C(**H**)-), 3.36 (h, J = 6.8 Hz, 1H, ^{i}Pr methine C—H bond), 2.52 (h, J = 6.7 Hz, , ^{i}Pr methine C—H bond), 2.39 (h, J = 6.6 Hz, 1H, , ^{i}Pr methine C—H bond), 1.59 (s,

18H, Cp* overlapping with a , ^{i}Pr methine C—H bond), 1.51 (d, *J* = 6.7 Hz, 3H), 1.26 – 1.14 (m, 12H), 1.11 (br, 3H), 0.943 (d, *J* = 6.5 Hz, 3H ^{i}Pr methine CH**Me**$_2$), 0.940 (d, *J* = 6.9 Hz, 3H ^{i}Pr methine CH**Me**$_2$)**.** Note that residual toluene can be seen in the spectrum (2.11 ppm). **^{13}C NMR** (151 MHz, C_6D_6) δ 210.64 (CO), 188.78 (NHC N**C**N), 147.13, 146.22 (d, *J* = 136.2 Hz, phosphanorcaradiene vinyl **C**), 137.15, 129.57, 128.95, 128.18, 128.08 (d, *J* = 115.6 Hz, phosphanorcaradiene vinyl **C**),127.84, 127.68, 127.51, 125.31, 124.52, 124.05, 123.57, 121.62, 120.12, 112.62, 95.12 (**C**$_5$Me$_5$), 32.65 (^{i}Pr methine **C**HMe$_2$), 30.32 (^{i}Pr methine **C**HMe$_2$), 28.61 (^{i}Pr methine **C**HMe$_2$), 28.00 (^{i}Pr methine **C**HMe$_2$), 27.42 (^{i}Pr CH**Me**$_2$), 25.57(^{i}Pr CH**Me**$_2$), 24.43(^{i}Pr CH**Me**$_2$), 23.97(^{i}Pr CH**Me**$_2$), 23.25 (^{i}Pr CH**Me**$_2$), 22.44 (d, *J* = 84.0 Hz, phosphanorcaradiene **C**—P), 22.37 (d, *J* = 84.0 Hz, phosphanorcaradiene **C**—P),21.16 (^{i}Pr CH**Me**$_2$), 21.04(^{i}Pr CH**Me**$_2$) , 20.50(^{i}Pr CH**Me**$_2$), 19.96 (^{i}Pr CH**Me**$_2$), 9.77 (C$_5$**Me**$_5$) **IR:** CO at 1902 cm^{-1}**.** **Elemental Analysis:** C, 66.74; H, 7.52; N, 4.10. Found: C: 67.07; H: 7.80; N. 3.80.

Synthesis of phosphaazaallene (3)

In a glovebox **2** (0.30 g; 0.0439 mmol) was loaded into a 20 mL vial with a magnetic stir bar, dissolved in C_6D_6 (0.5 mL) and stirred to produce a yellow solution. To a separate 20 mL vial Xylyl isocyanide (0.00575 g; 0.0439 mmol, 1.0 equiv) was loaded and dissolved in 0.5 mL C_6D6 to produce a colorless solution. The C_6D_6 solution of Xylyl isocyanide was then added dropwise to the rapidly stirring C_6D_6 solution of **2** over 1 min; No color change was observed. Remaining amounts of Xylyl isocyanide were dissolved in 0.1 mL C_6D_6 and added to the reaction mixture. After stirring for 10 minutes, the reaction mixture was transferred to a J-Young NMR Spectroscopy tube, removed from a glovebox and initial NMR spectral readings were obtained. The solution was then heated to 70 ^{o}C in a oil bath and the consumption of **2** and Xylyl isocyanide were monitored. After heating for 72 hrs, the solution turned from bright yellow to bright red/orange, and approximately 95% conversion was observed by NMR spectroscopy. At this point the reaction mixture was cooled to room temperature and cycled back into a glovebox. All C_6D_6 was removed in vacuo to afford a bright orange residue, which was extracted with

toluene (3 X 2 mL) and filtered over a celite plug (0.5 x 2 cm) into a new 20 mL vial. The orange solution was concentrated to 0.5 mL and maintained at –35 °C for 12 h, affording orange crystalline material. The toluene supernatant was removed and placed in an additional 20 mL vial, which was again cooled to –35 °C for 12 h affording a second crop of bright orange crystalline material, the supernatant of which was discarded. The two crops were combined, affording 0.030 g (0.019 mol; 92% yield) of an analytically pure red/orange crystalline material. X-ray quality crystals were grown from a saturated pentane solution cooled to –35 °C.

Characterization data:

^{1}H NMR (600 MHz, C_6D_6) δ 7.25 (m, 5H Dipp arene C—H bonds, the rest is obscured by the residual solvent peak), 6.93 (m, 2H, *m*-C—H bonds of Xyl), 6.84 (m, 1H, *p*-C—H bond of Xyl), 6.41(s, 2H, methine C—H bonds of IPr ligand), 3.42 (br, 4H, Dipp ^{i}Pr methine C—H bonds) 2.65 (s, 6H, *o*-Me bonds of Xyl), 1.49-1.43 (br, 12H, Dipp ^{i}Pr methyl C—H bonds) 1.41 (s, 15H, Cp* Me C—H bonds) 1.05 (br, 6 H, Dipp ^{i}Pr methyl C—H bonds), 0.96 (br, 6 H, Dipp ^{i}Pr methyl C—H bonds). **^{13}C NMR** (151 MHz, Benzene-d_6) δ 207.87 (CO), 190.65 (NCN of N-heterocyclic carbene), 182.97 (d, J = 74.6 Hz, P=C=N-Xyl), 141.14 (*ipso*-**C** of Dipp or Xyl), 141.08 (*ipso*-**C** of Dipp or Xyl), (*ortho*-**C** of Dipp or Xyl), 130.33 (*meta*-**C** of Dipp or Xyl), 129.79 (methine **C**—H of NHC), 123.12, 95.33 ($\mathbf{C}_5Me_5$), 34.38 (residual pentane), 28.92 (residual pentane), 26.95 (**C**HMe$_2$), 25.64 (**C**HMe$_2$), 23.94 (CH$\mathbf{Me}_2$) , 22.66 (CH$\mathbf{Me}_2$), 19.57 (o-Me of Xyl), 14.21 (residual pentane), 10.11 ($C_5\mathbf{Me}_5$). Note several of the aromatic resonances are obscured by residual C_6D_6 solvent signal. **^{31}P** (162 MHz, C_6D_6) δ – 166**. IR:** 1919 cm^{-1} (CO), 1785 cm^{-1} (PCNXyl). **Elemental Analysis:** C, 69.26; H, 7.42; N, 5.16. Found: C: 69.15; H: 7.61; N: 4.96.

Synthesis of Ru-imidazol-2-yl (4) and Dipp-P=(IMe$_4$) (5)

In a glovebox **2** (0.067 g; 0.097 mmol) was loaded into a 20 mL vial with a magnetic stir bar, dissolved in C_6D_6 (0.5 mL) and stirred to produce a yellow solution. To a separate 20 mL vial IMe_4 (0.036 g; 0.291 mmol, 3.0 equiv) was loaded and dissolved in 0.5 mL C_6D6 to produce a colorless solution. The C_6D_6 solution of IMe_4 was then added dropwise to the rapidly stirring C_6D_6 solution of **2** over 1 min; No color change was observed. Remaining amounts of IMe_4 were dissolved in 0.1 mL C_6D_6 and added to the reaction mixture. After stirring for 10 minutes, the reaction mixture was transferred to a J-Young NMR Spectroscopy tube, removed from a glovebox and initial NMR spectral readings were obtained. The solution was then heated to 80 ^{o}C in a oil bath and the consumption of **2** and IMe_4 were monitored. After heating for 96 hrs, the solution turned from bright yellow to yellow/orange, and approximately 91% conversion was observed by NMR spectroscopy. At this point the reaction mixture was cooled to room temperature and cycled back into a glovebox. All C_6D_6 was removed in vacuo to afford a pale orange residue, which was extracted with pentane (3 X 2 mL) and filtered over a celite plug (0.5 x 2 cm) into a new 20 mL vial. The pentane solution was concentrated to 0.3 mL and the solution was transferred to a 4 mL via which was then placed inside of a 20 mL vial containing cryogenic Paratone® oil. After sitting at room temperature overnight the volume of pentane significantly decreased – having been absorbed by the pentane oil – affording yellow crystalline material. The pentane supernatant was removed. The single crop afforded 0.0671 g of **4** and **5** in a 2.06:1 (71% combined yield) as an inseparable mixture of X-ray quality crystals. All attempts at separating **4** and **5** failed. Because **5** has already been fully characterized by the group of Hering-Junghaus and coworkers,[5] and **4** and **5** could not be separated after multiple attempts, the mixture of **4** and **5** were characterized together:

Characterization data:

^{1}H NMR (400 MHz, Benzene-*d*$_6$) δ 7.69 (d, *J* = 0.93 Hz, 1H, imidazole C—H of **4**), 7.38 – 7.22 (m, 3H, 3H, *m*-C—**H** Dipp + *p*-C—**H** Dipp of DippP=IMe_4 (**5**)), 7.09 (dd, *J* = 7.6, 1.5 Hz, 1H, arene C—H bond of Dipp of **4**), 7.06 (m, 1H), 4.76 (hd, $^1J_{1H\text{-}1H}$ = 7.0, $^4J_{31P\text{-}1H}$ = 5.1 Hz, 2H, C**H**$(CH_3)_2$ of DippP=IMe_4 (**5**)),

3.60 (s, 6H, NC**H**$_3$ of **4**), 3.20 (s, 3H, NC**H**$_3$ of **4**), 2.79 (s, 6H, NC**H**$_3$ of DippP=IMe$_4$ (**5**)), 2.63 (h, *J* = 6.9 Hz, 1H, CH(C**H**$_3$)$_2$ of **4**), 2.13 (h, *J* = 6.9 Hz, 1H, CH(C**H**$_3$)$_2$ of **4**), 1.85 (s, 15H, Cp* C—**H** of **4**), 1.47 (d, *J* = 6.9 Hz, 3H, CH(C**H**$_3$)$_2$ of **4**), 1.45 (s, 3H, C—C**H**$_3$ of **4**),1.37 (d, *J* = 6.9 Hz, 12H, CH(C**H**$_3$)$_2$ of DippP=IMe$_4$ (**5**)), 1.26 (s, 6H C—C**H**$_3$ of DippP=IMe$_4$ (**5**)), 1.23 (d, *J* = 6.9 Hz, 3H, CH(C**H**$_3$)$_2$ of **4**), 1.03 (d, *J* = 6.8 Hz, 3H, CH(C**H**$_3$)$_2$ of **4**), 0.56 (d, *J* = 6.9 Hz, 3H, CH(C**H**$_3$)$_2$ of **4**), 0.12 (s, 3H, C—C**H**$_3$ of 4). **^{13}C NMR** (151 MHz, Benzene-d_6) δ 210.60 (**C**O of **5**), 181.72 (imidazole N**C**N of **5**), 169.70 (d, $^1J_{31P-13C}$ = 103.0 Hz, N**C**N DippP=IMe$_4$ (**5**)), 154.38 (*d*, $^2J_{31P-13C}$, *J* = 8.4 Hz of DippP=IMe$_4$ (**5**)), 148.75 (*o*-C of Dipp of **4**), 146.43 (*o*-C of Dipp of **4**), 140.51 (*o*-C of Dipp of DippP=IMe$_4$ (**5**)), 128.84 (*i*-C of Dipp of DippP=IMe$_4$ (**5**)), 128.53 (imidazole N-**C**=C-N(Dipp) of **5**), 128.29, (m-C of Dipp of **4),** 127.07 (*m*-C of Dipp of **4**), 125.10 (imidazole N-C=**C**-N(Dipp) of **5**),123.75 (*p*-C of Dipp of **4**), 122.46 (*m*-C of Dipp of DippP=IMe$_4$ (**5**)), 120.63 (*p*-C of Dipp of DippP=IMe$_4$ (**5**)), 95.78 (**C**$_5$Me$_5$), 35.20 (d, $^3J_{31P-13C}$ = 47.9 Hz, Dipp **C**H(CH$_3$)$_2$ of DippP=IMe$_4$ (**5**)), 33.86 (d, *J* = 12.1 Hz), 32.11 (d, *J* = 12.9 Hz), 28.13 (Dipp CH(**C**H$_3$)$_2$ of **4**), 26.19 (Dipp CH(**C**H$_3$)$_2$ of **4**), 24.32 (Dipp CH(**C**H$_3$)$_2$ of DippP=IMe$_4$ (**5**)), 23.47 (Dipp CH(**C**H$_3$)$_2$ of **4**), 10.15 (**Me**-C of IMe$_4$ of 4), 9.39 (C$_5$**Me**$_5$), 8.60 (**Me**-C of IMe$_4$ of DippP=IMe$_4$ (**5**)), 2.02 (**Me**-C of IMe$_4$ of 4). **^{31}P NMR** (122 MHz, C_6D_6) δ = -86.1 ppm.

Proposed mechanism for formation of 4 and 5 from 2 and IMe4:

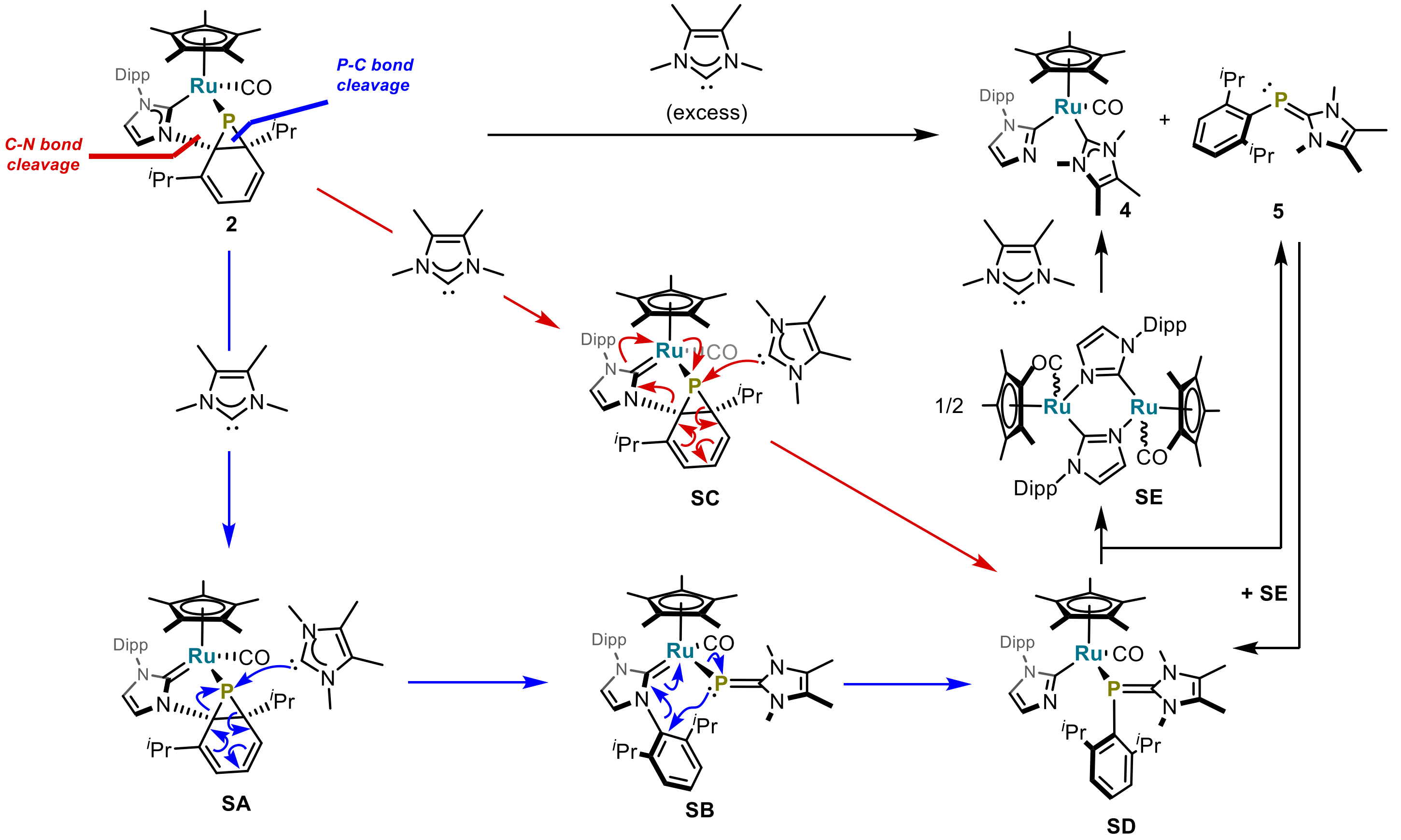

Figure S1: Proposed mechanism for formation of **4** and **5** from **2** and IMe_4

Commentary: The formation of **4** and **5** from the reaction of **2** with an excess of IMe_4 could conceivably proceed through two pathways: If IMe_4 initially attacks the phosphanorcaradiene P-atom (blue arrows) resulting in P—C bond cleavage (**SA**), a Ru-bound IMe_4 phosphinidene intermediate (**SB**) may form; this compound may directly attack the IPr-Dipp N—C bond, resulting in net Dipp transfer to the P-atom (**SD**). It is worth noting that this proposed process results in conversion of the covalent Ru—P bond and the dative Ru—IPr bond into a dative Ru—P bond and a covalent Ru—(C)imidazole bond; thus the Ru(II) oxidation state is preserved. Alternatively, IMe_4 may directly attack the P-atom of the phosphanorcaradiene (red arrows) resulting in C—N bond cleavage of the IPr—Dipp bonds; this would produce **SD** directly and in a single step from **2**. Note that the red arrows also result in a net transformation of the dative IPr into a covalent Ru—(C)imidazole bond and the conversion of the

phosphanorcaradiene into a dative Dipp-P=IMe_4. Liberation of Dipp-P=IMe_4 (**5**) from **SD** may then proceed due to the presence of excess IMe_4. At this time there is no spectroscopic evidence for formation of **SE**, although conversion of SD to **4** with liberation of **5** likely proceeds through a dissociative ligand pathway, as **SD** is coordinatively saturated and possesses eighteen valence electrons. Note that the reaction requires an excess (2.5-3 equiv) of IMe_4.

Synthesis of Iminophosphanide (6)

In a glovebox **2** (0.030 g; 0.044 mmol) was loaded into a 20 mL vial with a magnetic stir bar, dissolved in C_6D_6 (1 mL) and stirred to produce a pale yellow solution. To a separate 20 mL vial TsN_3 (0.00865 g; 0.044 mmol, 1.0 equiv) was loaded and dissolved in 0.5 mL C_6D_6 to produce a colorless solution. The THF solution of TsN_3 was then added dropwise to the rapidly stirring C_6D_6 solution of **2** over 1 min; this resulted in a rapid color change from pale yellow to dark black/purple. Remaining amounts of TsN_3 were dissolved in 0.1 mL C_6D_6 and added to the reaction mixture. After stirring for 10 minutes all C_6D_6 was removed in vacuo to afford a black tar, which was extracted with toluene (3 X 2 mL) and filtered over a celite plug (0.5 x 2 cm) into a new 20 mL vial. The dark purple solution was concentrated to 0.5 mL and maintained at –35 °C for 12 h, affording purple crystalline material. The toluene supernatant was removed and placed in an additional 20 mL vial, which was again cooled to –35 °C for 12 h affording a second crop of purple crystalline material, the supernatant of which was discarded. The two crops were combined, affording 0.033 g (0.039 mol; 88% yield) of crystalline purple material. X-ray quality crystals were grown from a saturated toluene solution cooled to –35 °C.

Characterization data:

^{1}H NMR (600 MHz, Benzene-d_6) δ 8.19 (d, *J* = 8.1 Hz, 2H, *o*-C—H bonds of -$S(O)_2$Tol), 7.25 (m, 2H, arene C—H bonds of Dipp), 7.08 – 6.98 (m, 2H), 6.89 (d, *J* = 8.1 Hz, 2H, *m*-C—H bonds of -$S(O)_2$Tol)

6.48 (s, 2H, methine C—H bonds of IPr ligand), 3.01 (br, 2H, Dipp ^{i}Pr methine C—H bonds), 2.88 (h, *J* = 6.7 Hz, 2H, Dipp ^{i}Pr methine C—H bonds), 1.95 (s, 3H, *p*-Me of Tol of $S(O)_2$Tol), 1.47 – 1.37 (m, 12H, Dipp ^{i}Pr methyl C—H bonds), 1.28 (s, 15H, C—H bonds of Cp*), 0.99 (d, *J* = 6.7 Hz, 6H, Dipp ^{i}Pr methyl C—H bonds), 0.92 (d, *J* = 6.7 Hz, 6H, Dipp ^{i}Pr methyl C—H bonds). Note, some of the aromatic peaks are obscured by the C_6D_6 solvent signal. **^{13}C NMR** (151 MHz, C_6D_6) δ 205.63 (**C**O), 184.33 (NHC N**C**N), 143.79 (**C**-Me of -$S(O)_2$Tol), 130.83 (**C**-Me of -$S(O)_2$Tol), 128.16 (methine **C**—H of NHC or *i*-C-Tol of -$S(O)_2$Tol or H_2N-**C** of Dipp), 127.84 16 (methine **C**—H of NHC or *i*-**C**-Tol of -$S(O)_2$Tol or H_2N-**C** of Dipp), 126.41 (*m*-**C** of -$S(O)_2$Tol or *m*-C of Dipp), 125.64 (*p*-**C** of Tol or *p*-**C** of Dipp), 125.25 (*p*-**C** of Tol or *p*-**C** of Dipp), 100.16 (**C**$_5$Me$_5$), 29.19 (Dipp **C**HMe$_2$), 28.57 (Dipp **C**HMe$_2$), 26.75 (Dipp CH**Me**$_2$) , 26.58 (Dipp CH**Me**$_2$), 26.16 (Dipp CH**Me**$_2$), 25.59 (Dipp CH**Me**$_2$), 23.48 (Dipp CH**Me**$_2$), 22.45 (Dipp CH**Me**$_2$), 21.36 (Me of Tol), 21.10 (Me of -$S(O)_2$Tol), 9.28 (C$_5$**Me**$_5$). Note that several of the aromatic resonances are obscured by residual C_6D_6 solvent signal. **^{31}P** (162 MHz, C_6D_6) δ 234**. IR Spectroscopy:** 1945 cm^{-1} (strong, Ru—CO) 1140 cm^{-1} (strong Ru-P=NR). See computational section (*vide supra*) for further discussion on assignment. **Elemental analysis:** *Despite three separate attempts, we were unable to obtain satisfactory elemental analyses; nevertheless, all other spectroscopic and crystallographic data are in accordance with the structural assignment of* **6***.*

Synthesis of Phosphaformazan (7)

In a glovebox **2** (0.030 g; 0.044 mmol) was loaded into a 20 mL vial with a magnetic stir bar, dissolved in C_6D_6 (0.5 mL) and stirred to produce a pale yellow solution. To a separate 20 mL vial diphenyldiazaomethane (0.0213 g; 0.110 mmol, 2.5 equiv) was loaded and dissolved in 0.4 mL C_6D_6 to produce a colorless solution. The C_6D_6 solution of TsN_3 was then added dropwise to the rapidly stirring C_6D_6 solution of **2** over 1 min; this resulted in no color change. Remaining amounts of diphenyldiazaomethane were dissolved in 0.1 mL C_6D_6 and added to the reaction mixture. After stirring

for 10 minutes the solution was transferred to a J-Young NMR tube, removed from the glovebox, and heated in a 60 °C oil bath. The reaction was monitored by ^{1}H and ^{31}P NMR Spectroscopy, which showed the sluggish formation of a new product whilst the reaction solution turned progressively orange and then red. After 48 hours, **2** was nearly entirely consumed as determined by ^{1}H NMR spectroscopy. At this time, the J-Young tube was cycled back into a glovebox, transferred into a 20 mL vial and all C_6D_6 was removed. The red residue was then extracted with toluene (3 X 2 mL) and filtered over a celite plug (0.5 x 2 cm) into a new 20 mL vial. The red solution was concentrated to 0.5 mL and maintained at –35 °C for 12 h, affording red crystalline material. The toluene supernatant was removed and placed in an additional 20 mL vial, which was again cooled to –35 °C for 12 h affording a second crop of red crystalline material, the supernatant of which was discarded. The two crops were combined, affording 0.025 g (0.028 mol; 53% yield) of crystalline red material. X-ray quality crystals were grown from a saturated toluene solution cooled to –35 °C.

Characterization data:

^{1}H NMR (600 MHz, Benzene-*d*$_6$) δ 7.98 – 7.91 (m, 4H), 7.78 – 7.72 (m, 4H), 7.15 – 7.10 (m, 6H), 7.07-7.03 (m, 6H), 6.53 (s, 2H, methine C—H bonds of the IPr ligand), 3.85-2.90 (br, 4H, Dipp ^{i}Pr methine C—H bonds), 1.43 (br, 6H, Dipp ^{i}Pr methine C—H bonds) 1.27 (s, 15H, Cp*Me), 1.24 (d, *J* = 6.7 Hz, 6H, Dipp ^{i}Pr methine C—H bonds), 1.00 (d, *J* = 6.7 Hz, 6H, Dipp ^{i}Pr methine C—H bonds), 0.86 (d, *J* = 6.7 Hz, 6H, Dipp ^{i}Pr methine C—H bonds). **^{13}C NMR** (151 MHz, Benzene-*d*$_6$) δ 204.92 (d, $^{2}J_{31P\text{-}13C}$ = 29.4 Hz, **C**O), 184.66 (d, $^{2}J_{31P\text{-}13C}$ = 19.1 Hz, N**C**N of N-heterocyclic carbene), 149.77 (Ph$_2$**C**=N-N=), 149.52 (*i*-**C** of Dipp or *i*-**C** of **Ph**$_2$C=N=N), 148.36 (*i*-**C** of Dipp or *i*-**C** of **Ph**$_2$C=N=N), 145.34 (*o*-**C** of Dipp), 141.95 (*o*-**C** of Dipp), 139.93 (methine **C**—H of NHC or *m*-**C** of Dipp or *m*-**C** of **Ph**$_2$C=N=N),139.04 (methine **C**—H of NHC or *m*-**C** of Dipp or *m*-**C** of **Ph**$_2$C=N=N), 134.77 (methine

C—H of NHC or *m*-**C** of Dipp or *m*-**C** of **Ph**$_2$C=N=N), 131.56 (methine **C**—H of NHC or *m*-**C** of Dipp or *m*-**C** of **Ph**$_2$C=N=N), 131.18 (methine **C**—H of NHC or *m*-**C** of Dipp or *m*-**C** of **Ph**$_2$C=N=N), 130.53 (methine **C**—H of NHC or *m*-**C** of Dipp or *m*-**C** of **Ph**$_2$C=N=N), 130.15 (methine **C**—H of NHC or *m*-**C** of Dipp or *m*-**C** of **Ph**$_2$C=N=N), 129.37 (methine **C**—H of NHC or *m*-**C** of Dipp or *m*-**C** of **Ph**$_2$C=N=N), 127.77 (*p*-C of Dipp), 126.97 (*o*-**C** of Ph$_2$C=N=N),126.91 (*o*-**C** of **Ph**$_2$C=N=N), 97.75 (**C**$_5$Me$_5$), 28.54 (Dipp **C**HMe$_2$), 26.10 (Dipp **C**HMe$_2$), 23.66 (Dipp CH**Me**$_2$), 22.82 (Dipp CH**Me**$_2$), 10.72 (**C**$_5$Me$_5$). **^{31}P** (162 MHz, C_6D_6) δ 197.5. **IR Spectroscopy:** 1926 cm^{-1} (strong, Ru—CO), 1239 cm^{-1} (strong, P=N). **Elemental Analysis:** C, 71.68; H, 7.67; N, 7.84; found: 71.31, 7.90, 7.66.

X-Ray Crystallography:

phosphanorcaradiene (2)

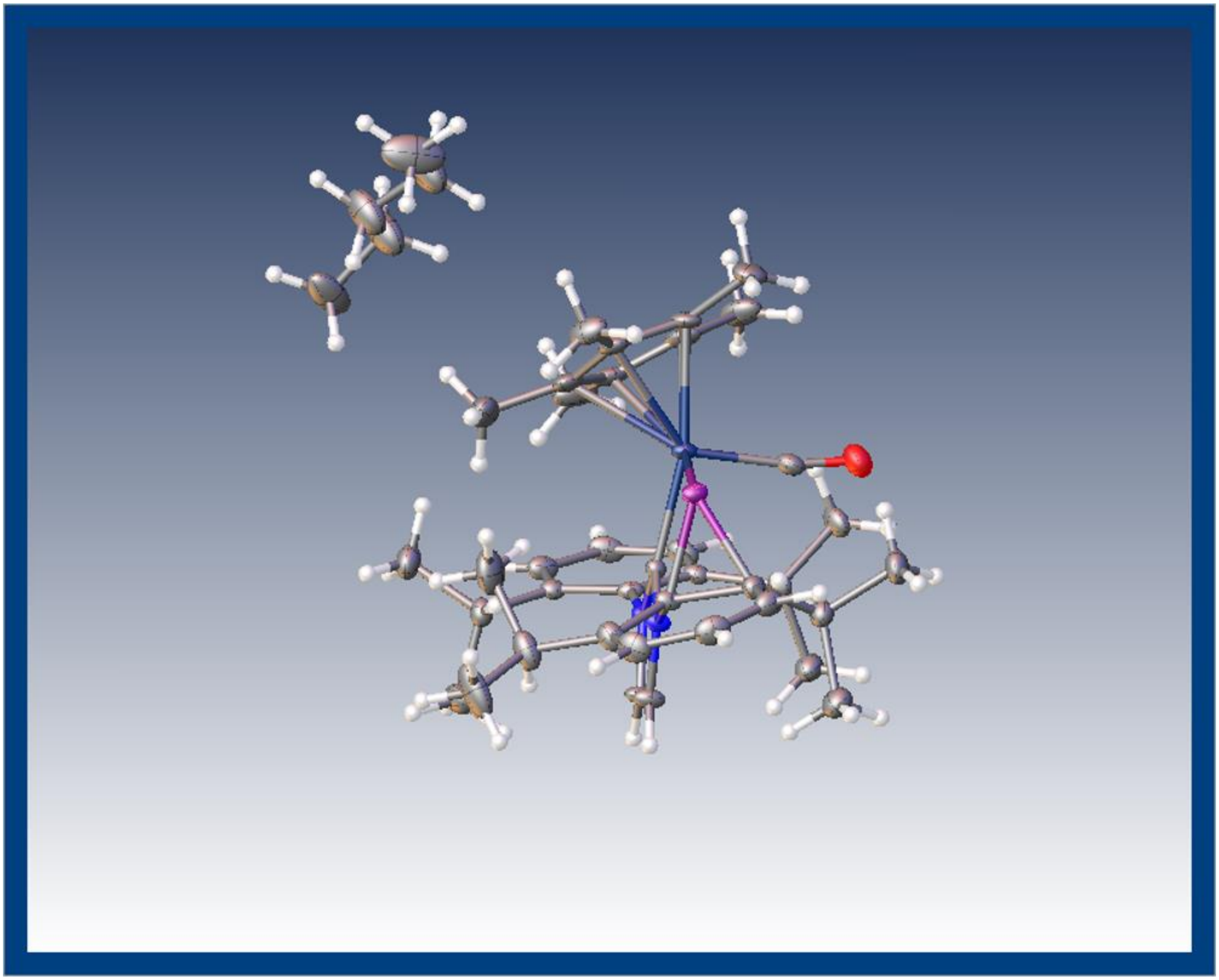

Table S1 Crystal data and structure refinement for 2.

Identification code	TSD40_Tilley
Empirical formula	$C_{40.5}H_{57}N_2OPRu$
Formula weight	719.92
Temperature/K	77
Crystal system	monoclinic

Space group	$P2_1/n$
a/Å	10.3207(4)
b/Å	16.5373(7)
c/Å	21.9400(8)
α/°	90
β/°	97.019(4)
γ/°	90
Volume/Å^3	3716.6(3)
Z	4
ρ_{calc}g/cm^3	1.287
μ/mm^{-1}	0.498
F(000)	1524.0
Crystal size/mm^3	0.31 × 0.16 × 0.1
Radiation	Mo Kα (λ = 0.71073)
2Θ range for data collection/°	6.188 to 52.744
Index ranges	-12 ≤ h ≤ 12, -20 ≤ k ≤ 20, -27 ≤ l ≤ 27
Reflections collected	41159
Independent reflections	7594 [R_{int} = 0.0583, R_{sigma} = 0.0374]
Data/restraints/parameters	7594/58/436
Goodness-of-fit on F^2	1.027
Final R indexes [I>=2σ (I)]	R_1 = 0.0376, wR_2 = 0.0865
Final R indexes [all data]	R_1 = 0.0445, wR_2 = 0.0892
Largest diff. peak/hole / e Å^{-3}	1.00/-0.70

Table S2 Fractional Atomic Coordinates (×10^4) and Equivalent Isotropic Displacement Parameters (Å^2×10^3) for 2. U_{eq} is defined as 1/3 of the trace of the orthogonalised U_{IJ} tensor.

Atom	*x*	*y*	*z*	U(eq)
Ru1	5393.3(2)	3891.7(2)	6745.2(2)	15.27(7)
P1	5049.7(6)	4341.7(4)	7733.3(3)	16.20(13)
N1	5247(2)	2686.9(12)	7769.0(9)	15.4(4)
N2	5592.8(19)	1984.1(12)	6981.2(9)	13.3(4)
O1	8282(2)	4160.4(13)	6924.7(11)	34.8(5)
C11	5356(2)	2763.4(14)	7154.2(11)	13.5(5)
C4	4996(3)	5017.2(16)	6194.3(12)	21.5(5)
C20	4630(2)	1285.7(14)	6048.7(11)	16.6(5)
C12	5706(2)	1685.5(14)	6370.1(10)	14.4(5)
C26	4972(2)	3360.0(14)	8158.5(11)	16.0(5)
C5	5381(3)	4392.1(18)	5791.3(12)	26.1(6)
C38	7182(3)	4001.6(16)	6896.7(12)	22.3(5)
C24	5671(3)	1455.5(15)	7479.1(11)	19.5(5)
C1	4428(3)	3771.5(17)	5752.6(12)	28.7(6)
C13	6912(2)	1749.2(15)	6142.9(11)	17.2(5)
C3	3776(3)	4775.8(16)	6370.9(12)	22.4(6)
C34	6157(2)	3870.9(15)	8401.4(11)	18.7(5)
C19	4785(3)	971.0(16)	5469.6(12)	22.8(5)
C2	3446(3)	4003.0(18)	6120.1(13)	28.8(6)
C25	5452(3)	1898.1(15)	7969.4(11)	21.0(5)
C17	7006(3)	1427.5(16)	5560.7(12)	23.0(6)
C14	8105(2)	2094.1(16)	6526.8(12)	19.7(5)
C27	3909(3)	3218.8(17)	8552.3(12)	23.2(6)
C16	8967(3)	2591.8(18)	6149.2(14)	28.9(6)

Table S2 Fractional Atomic Coordinates (×10^4) and Equivalent Isotropic Displacement Parameters (Å^2×10^3) for 2. U_{eq} is defined as 1/3 of the trace of the orthogonalised U_{IJ} tensor.

**Atom**	***x***	***y***	***z***	**U(eq)**
C33	6028(3)	4273.7(16)	8995.4(12)	24.2(6)
C18	5952(3)	1050.6(16)	5226.9(12)	25.9(6)
C21	3346(2)	1162.1(17)	6313.0(13)	22.5(5)
C35	7536(3)	3566.1(17)	8347.5(13)	23.4(6)
C32	4999(3)	4159.8(18)	9304.1(12)	28.1(6)
C31	3948(3)	3632.3(18)	9083.9(13)	28.1(6)
C8	2950(3)	5292(2)	6737.8(14)	35.3(7)
C9	5679(3)	5809.5(18)	6318.9(15)	34.7(7)
C28	2761(3)	2687.5(19)	8296.2(15)	32.7(7)
C15	8888(3)	1402.6(18)	6859.9(15)	31.0(6)
C37	8500(3)	4272.7(19)	8367.3(16)	33.6(7)
C36	7984(3)	2951.9(19)	8855.2(15)	32.2(7)
C23	3305(3)	322.0(19)	6609.1(15)	31.7(7)
C10	6510(3)	4431(2)	5429.9(14)	38.2(8)
C6	4299(4)	3077.2(19)	5306.3(14)	40.4(8)
C7	2177(3)	3563(2)	6159.1(17)	46.7(9)
C22	2146(3)	1241(2)	5829.9(16)	34.6(7)
C29	2089(3)	3056(2)	7704.6(17)	46.8(9)
C30	1779(4)	2551(3)	8753(2)	54.2(11)
C43	-14(9)	3342(6)	4592(3)	58(2)
C42	559(11)	4171(6)	4581(7)	53.1(18)
C41	-187(10)	4755(5)	4933(6)	53.1(18)
C40	448(11)	5531(5)	5108(6)	53.1(18)
C39	-214(15)	6086(7)	5482(5)	70(4)

Table S3 Anisotropic Displacement Parameters (Å^2×10^3) for 2. The Anisotropic displacement factor exponent takes the form: -2π^2[h^{2}a*^{2}U$_{11}$+2hka*b*U$_{12}$+…].

Atom	U_{11}	U_{22}	U_{33}	U_{23}	U_{13}	U_{12}
Ru1	22.72(11)	10.46(10)	12.93(10)	2.22(7)	3.36(7)	2.04(8)
P1	22.4(3)	11.3(3)	14.5(3)	-0.8(2)	0.5(2)	2.9(2)
N1	22.8(11)	11.7(10)	12.0(9)	0.0(8)	3.5(8)	-0.1(8)
N2	18.6(10)	10.3(9)	11.2(9)	0.3(7)	3.5(7)	0.1(8)
O1	29.0(11)	31.7(12)	44.4(13)	3.5(10)	7.2(9)	-7.7(9)
C11	14.2(11)	12.3(11)	14.0(11)	-0.2(9)	1.7(9)	-1.2(9)
C4	30.3(14)	14.5(12)	20.0(12)	8.8(10)	3.8(11)	3.9(10)
C20	21.1(12)	11.5(11)	17.2(11)	0.6(9)	1.9(9)	0.5(9)
C12	21.7(12)	10.2(11)	11.7(11)	-0.1(9)	3.6(9)	3.9(9)
C26	24.0(12)	11.2(11)	12.7(11)	-1.7(9)	2.3(9)	-0.7(9)
C5	34.3(15)	30.6(15)	14.3(12)	11.3(11)	6.6(11)	12.4(12)
C38	27.3(14)	17.5(13)	22.9(13)	5.1(10)	6.3(11)	-1.1(11)
C24	31.2(14)	11.7(12)	15.8(11)	3.8(9)	3.8(10)	2.2(10)
C1	46.7(17)	19.5(14)	17.1(12)	5.5(11)	-6.9(12)	7.4(12)
C13	22.4(12)	12.2(11)	17.7(12)	1.7(9)	5.7(10)	0.9(10)
C3	25.5(13)	22.4(14)	18.8(12)	9.6(10)	1.1(10)	6.6(11)
C34	19.9(12)	15.9(12)	19.8(12)	-1.3(10)	0.7(9)	-2.1(10)
C19	33.8(15)	15.6(13)	18.1(12)	-2.7(10)	0.0(11)	-1.1(11)
C2	33.8(15)	24.7(15)	25.1(14)	12.5(11)	-8.3(12)	0.0(12)
C25	34.8(15)	14.3(12)	14.2(11)	2.0(9)	4.5(10)	1.6(10)
C17	30.4(14)	19.9(13)	21.1(13)	-0.5(10)	13.3(11)	0.6(11)
C14	18.3(12)	17.0(12)	24.9(13)	0.1(10)	7.0(10)	-0.6(10)
C27	24.7(13)	21.2(14)	24.9(13)	-4.0(11)	8.1(11)	-0.9(11)
C16	21.8(14)	27.1(15)	39.8(16)	3.5(13)	12.4(12)	-3.0(11)

Table S3 Anisotropic Displacement Parameters (Å^2×10^3) for 2. The Anisotropic displacement factor exponent takes the form: $-2\pi^2[h^2a^{*2}U_{11}+2hka^*b^*U_{12}+\ldots]$.

Atom	U_{11}	U_{22}	U_{33}	U_{23}	U_{13}	U_{12}
C33	29.2(14)	19.2(13)	22.3(13)	-3.0(11)	-4.3(11)	0.0(11)
C18	44.5(17)	18.8(13)	16.1(12)	-3.0(10)	10.4(11)	1.2(12)
C21	18.2(12)	21.8(13)	27.3(13)	-3.7(11)	1.7(10)	-0.9(10)
C35	18.9(13)	22.4(14)	28.6(14)	0.1(11)	1.2(11)	-0.3(10)
C32	40.5(17)	26.7(15)	16.9(13)	-5.6(11)	3.1(12)	6.5(12)
C31	31.6(15)	29.3(15)	26.4(14)	-2.2(12)	15.8(12)	3.3(12)
C8	33.5(16)	42.2(18)	31.6(16)	10.3(14)	9.6(13)	17.8(14)
C9	44.1(18)	18.3(14)	40.9(18)	11.9(13)	1.6(14)	-2.5(13)
C28	27.6(15)	31.4(16)	41.8(17)	-12.4(14)	15.4(13)	-9.2(12)
C15	26.5(14)	24.5(15)	39.9(17)	4.0(13)	-4.2(12)	0.7(12)
C37	21.9(14)	31.7(17)	45.9(18)	-2.0(14)	-0.9(13)	-7.7(12)
C36	26.7(15)	31.9(16)	36.7(16)	3.2(13)	-1.4(12)	3.6(12)
C23	25.6(15)	32.7(16)	38.3(17)	6.8(13)	9.9(13)	-4.4(12)
C10	43.0(18)	48(2)	26.0(15)	16.1(14)	14.7(14)	15.4(15)
C6	69(2)	25.9(16)	22.9(15)	-0.2(12)	-8.6(15)	5.8(15)
C7	37.5(18)	51(2)	46(2)	18.6(17)	-15.4(15)	-12.2(16)
C22	23.5(14)	33.0(17)	44.7(18)	-2.9(14)	-5.9(13)	-3.8(12)
C29	36.6(18)	56(2)	47(2)	-15.9(17)	-0.1(15)	-17.6(17)
C30	44(2)	55(2)	71(3)	-26(2)	37(2)	-23.2(18)
C43	84(6)	67(5)	23(3)	4(4)	5(4)	-41(5)
C42	45(4)	46(5)	75(3)	-5(4)	32(3)	-9(3)
C41	45(4)	46(5)	75(3)	-5(4)	32(3)	-9(3)
C40	45(4)	46(5)	75(3)	-5(4)	32(3)	-9(3)
C39	140(12)	37(6)	35(4)	-11(5)	16(6)	4(6)

Table S4 Bond Lengths for 2.

Atom	Atom	Length/Å	Atom	Atom	Length/Å
Ru1	P1	2.3594(6)	C24	C25	1.343(4)
Ru1	C11	2.073(2)	C1	C2	1.423(4)
Ru1	C4	2.230(2)	C1	C6	1.504(4)
Ru1	C5	2.249(2)	C13	C17	1.398(4)
Ru1	C38	1.845(3)	C13	C14	1.516(4)
Ru1	C1	2.290(3)	C3	C2	1.416(4)
Ru1	C3	2.294(3)	C3	C8	1.507(4)
Ru1	C2	2.298(3)	C34	C33	1.484(4)
P1	C26	1.879(3)	C34	C35	1.528(4)
P1	C34	1.911(3)	C19	C18	1.381(4)
N1	C11	1.373(3)	C2	C7	1.510(4)
N1	C26	1.452(3)	C17	C18	1.384(4)
N1	C25	1.384(3)	C14	C16	1.528(4)
N2	C11	1.374(3)	C14	C15	1.533(4)
N2	C12	1.447(3)	C27	C31	1.348(4)
N2	C24	1.394(3)	C27	C28	1.526(4)
O1	C38	1.160(3)	C33	C32	1.341(4)
C4	C5	1.447(4)	C21	C23	1.536(4)
C4	C3	1.419(4)	C21	C22	1.534(4)
C4	C9	1.497(4)	C35	C37	1.532(4)
C20	C12	1.406(3)	C35	C36	1.536(4)
C20	C19	1.400(3)	C32	C31	1.430(4)
C20	C21	1.524(3)	C28	C29	1.523(5)
C12	C13	1.400(3)	C28	C30	1.526(4)
C26	C34	1.528(3)	C43	C42	1.493(8)

Table S4 Bond Lengths for 2.

Atom	Atom	Length/Å	Atom	Atom	Length/Å
C26	C27	1.496(4)	C42	C41	1.506(9)
C5	C1	1.416(4)	C41	C40	1.471(8)
C5	C10	1.489(4)	C40	C39	1.457(8)

Table S5 Bond Angles for 2.

Atom	Atom	Atom	Angle/˚	Atom	Atom	Atom	Angle/˚
C11	Ru1	P1	82.73(7)	C4	C5	C10	125.8(3)
C11	Ru1	C4	166.95(10)	C1	C5	Ru1	73.39(15)
C11	Ru1	C5	137.37(10)	C1	C5	C4	108.3(2)
C11	Ru1	C1	107.74(10)	C1	C5	C10	125.6(3)
C11	Ru1	C3	132.66(10)	C10	C5	Ru1	126.55(19)
C11	Ru1	C2	105.64(10)	O1	C38	Ru1	169.7(2)
C4	Ru1	P1	101.33(7)	C25	C24	N2	106.7(2)
C4	Ru1	C5	37.69(10)	C5	C1	Ru1	70.26(15)
C4	Ru1	C1	61.79(10)	C5	C1	C2	108.0(2)
C4	Ru1	C3	36.53(10)	C5	C1	C6	126.7(3)
C4	Ru1	C2	61.33(10)	C2	C1	Ru1	72.24(15)
C5	Ru1	P1	138.98(8)	C2	C1	C6	124.2(3)
C5	Ru1	C1	36.35(11)	C6	C1	Ru1	132.77(19)
C5	Ru1	C3	60.73(10)	C12	C13	C14	121.8(2)
C5	Ru1	C2	60.66(11)	C17	C13	C12	117.4(2)
C38	Ru1	P1	93.61(9)	C17	C13	C14	120.6(2)
C38	Ru1	C11	94.68(10)	C4	C3	Ru1	69.27(14)
C38	Ru1	C4	97.41(11)	C4	C3	C8	124.4(3)
C38	Ru1	C5	91.33(11)	C2	C3	Ru1	72.18(15)

Table S5 Bond Angles for 2.

Atom	Atom	Atom	Angle/˚	Atom	Atom	Atom	Angle/˚
C38	Ru1	C1	119.29(12)	C2	C3	C4	109.1(2)
C38	Ru1	C3	132.08(11)	C2	C3	C8	126.4(3)
C38	Ru1	C2	151.99(11)	C8	C3	Ru1	127.15(18)
C1	Ru1	P1	143.55(8)	C26	C34	P1	65.15(13)
C1	Ru1	C3	60.14(10)	C26	C34	C35	120.3(2)
C1	Ru1	C2	36.14(11)	C33	C34	P1	112.44(18)
C3	Ru1	P1	86.76(7)	C33	C34	C26	113.6(2)
C3	Ru1	C2	35.93(10)	C33	C34	C35	113.6(2)
C2	Ru1	P1	107.72(8)	C35	C34	P1	123.39(18)
C26	P1	Ru1	101.77(8)	C18	C19	C20	121.1(2)
C26	P1	C34	47.53(10)	C1	C2	Ru1	71.62(16)
C34	P1	Ru1	115.68(8)	C1	C2	C7	125.8(3)
C11	N1	C26	123.6(2)	C3	C2	Ru1	71.89(15)
C11	N1	C25	111.8(2)	C3	C2	C1	108.0(3)
C25	N1	C26	124.6(2)	C3	C2	C7	125.6(3)
C11	N2	C12	127.8(2)	C7	C2	Ru1	129.0(2)
C11	N2	C24	111.53(19)	C24	C25	N1	107.1(2)
C24	N2	C12	120.6(2)	C18	C17	C13	121.1(2)
N1	C11	Ru1	121.09(17)	C13	C14	C16	112.9(2)
N1	C11	N2	102.93(19)	C13	C14	C15	109.2(2)
N2	C11	Ru1	135.36(17)	C16	C14	C15	110.6(2)
C5	C4	Ru1	71.88(14)	C26	C27	C28	117.9(2)
C5	C4	C9	125.4(3)	C31	C27	C26	118.3(2)
C3	C4	Ru1	74.20(14)	C31	C27	C28	123.4(3)
C3	C4	C5	106.5(2)	C32	C33	C34	122.8(3)

Table S5 Bond Angles for 2.

Atom	Atom	Atom	Angle/˚	Atom	Atom	Atom	Angle/˚
C3	C4	C9	127.6(3)	C19	C18	C17	120.4(2)
C9	C4	Ru1	125.48(19)	C20	C21	C23	110.6(2)
C12	C20	C21	122.9(2)	C20	C21	C22	113.1(2)
C19	C20	C12	117.4(2)	C22	C21	C23	107.9(2)
C19	C20	C21	119.7(2)	C34	C35	C37	110.7(2)
C20	C12	N2	118.5(2)	C34	C35	C36	111.3(2)
C13	C12	N2	118.7(2)	C37	C35	C36	110.3(2)
C13	C12	C20	122.6(2)	C33	C32	C31	122.0(3)
N1	C26	P1	110.29(16)	C27	C31	C32	122.9(3)
N1	C26	C34	115.0(2)	C27	C28	C30	113.0(3)
N1	C26	C27	115.5(2)	C29	C28	C27	109.6(3)
C34	C26	P1	67.32(13)	C29	C28	C30	110.4(3)
C27	C26	P1	120.03(18)	C43	C42	C41	110.6(8)
C27	C26	C34	119.8(2)	C40	C41	C42	117.0(6)
C4	C5	Ru1	70.43(14)	C39	C40	C41	118.2(7)

Table S6 Torsion Angles for 2.

A	B	C	D	Angle/˚	A	B	C	D	Angle/˚
Ru1	P1	C26	N1	4.62(18)	C5	Ru1	C38	O1	24.8(14)
Ru1	P1	C26	C34	114.17(12)	C5	C4	C3	Ru1	65.09(17)
Ru1	P1	C26	C27	-133.43(18)	C5	C4	C3	C2	3.6(3)
Ru1	C4	C5	C1	64.07(18)	C5	C4	C3	C8	-173.3(2)
Ru1	C4	C5	C10	-121.5(3)	C5	C1	C2	Ru1	-61.53(18)
Ru1	C4	C3	C2	-61.49(18)	C5	C1	C2	C3	1.6(3)
Ru1	C4	C3	C8	121.6(3)	C5	C1	C2	C7	173.2(3)

Table S6 Torsion Angles for 2.

A	B	C	D	Angle/˚	A	B	C	D	Angle/˚
Ru1	C5	C1	C2	62.80(18)	C24	N2	C11	Ru1	168.5(2)
Ru1	C5	C1	C6	-129.2(3)	C24	N2	C11	N1	-2.1(3)
Ru1	C1	C2	C3	63.12(18)	C24	N2	C12	C20	76.4(3)
Ru1	C1	C2	C7	-125.3(3)	C24	N2	C12	C13	-98.6(3)
Ru1	C3	C2	C1	-62.95(18)	C1	Ru1	C38	O1	49.2(14)
Ru1	C3	C2	C7	125.4(3)	C13	C17	C18	C19	-1.3(4)
P1	Ru1	C38	O1	-114.5(14)	C3	Ru1	C38	O1	-25.6(15)
P1	C26	C34	C33	104.9(2)	C3	C4	C5	Ru1	-66.67(17)
P1	C26	C34	C35	-115.6(2)	C3	C4	C5	C1	-2.6(3)
P1	C26	C27	C31	-71.3(3)	C3	C4	C5	C10	171.8(3)
P1	C26	C27	C28	102.0(3)	C34	P1	C26	N1	-109.6(2)
P1	C34	C33	C32	75.2(3)	C34	P1	C26	C27	112.4(2)
P1	C34	C35	C37	78.3(3)	C34	C26	C27	C31	8.2(4)
P1	C34	C35	C36	-158.7(2)	C34	C26	C27	C28	-178.5(2)
N1	C26	C34	P1	102.68(19)	C34	C33	C32	C31	0.5(4)
N1	C26	C34	C33	-152.5(2)	C19	C20	C12	N2	-177.2(2)
N1	C26	C34	C35	-12.9(3)	C19	C20	C12	C13	-2.4(4)
N1	C26	C27	C31	152.7(3)	C19	C20	C21	C23	81.9(3)
N1	C26	C27	C28	-34.0(3)	C19	C20	C21	C22	-39.3(3)
N2	C12	C13	C17	177.6(2)	C2	Ru1	C38	O1	25.7(15)
N2	C12	C13	C14	2.2(3)	C25	N1	C11	Ru1	-170.29(17)
N2	C24	C25	N1	-0.2(3)	C25	N1	C11	N2	2.0(3)
C11	Ru1	C38	O1	162.6(14)	C25	N1	C26	P1	170.2(2)
C11	N1	C26	P1	-8.7(3)	C25	N1	C26	C34	96.6(3)
C11	N1	C26	C34	-82.3(3)	C25	N1	C26	C27	-49.6(3)

Table S6 Torsion Angles for 2.

A	B	C	D	Angle/˚	A	B	C	D	Angle/˚
C11	N1	C26	C27	131.5(2)	C17	C13	C14	C16	41.1(3)
C11	N1	C25	C24	-1.2(3)	C17	C13	C14	C15	-82.4(3)
C11	N2	C12	C20	-100.9(3)	C14	C13	C17	C18	174.6(2)
C11	N2	C12	C13	84.1(3)	C27	C26	C34	P1	-112.7(2)
C11	N2	C24	C25	1.5(3)	C27	C26	C34	C33	-7.8(3)
C4	Ru1	C38	O1	-12.5(14)	C27	C26	C34	C35	131.7(3)
C4	C5	C1	Ru1	-62.17(17)	C33	C34	C35	C37	-63.6(3)
C4	C5	C1	C2	0.6(3)	C33	C34	C35	C36	59.5(3)
C4	C5	C1	C6	168.6(3)	C33	C32	C31	C27	-0.5(5)
C4	C3	C2	Ru1	59.68(18)	C21	C20	C12	N2	0.7(3)
C4	C3	C2	C1	-3.3(3)	C21	C20	C12	C13	175.5(2)
C4	C3	C2	C7	-174.9(3)	C21	C20	C19	C18	-177.9(2)
C20	C12	C13	C17	2.8(4)	C35	C34	C33	C32	-138.7(3)
C20	C12	C13	C14	-172.6(2)	C31	C27	C28	C29	113.4(3)
C20	C19	C18	C17	1.6(4)	C31	C27	C28	C30	-10.2(5)
C12	N2	C11	Ru1	-14.0(4)	C8	C3	C2	Ru1	-123.4(3)
C12	N2	C11	N1	175.4(2)	C8	C3	C2	C1	173.6(2)
C12	N2	C24	C25	-176.2(2)	C8	C3	C2	C7	2.0(4)
C12	C20	C19	C18	0.1(4)	C9	C4	C5	Ru1	121.1(3)
C12	C20	C21	C23	-96.0(3)	C9	C4	C5	C1	-174.8(3)
C12	C20	C21	C22	142.8(3)	C9	C4	C5	C10	-0.4(4)
C12	C13	C17	C18	-0.9(4)	C9	C4	C3	Ru1	-122.9(3)
C12	C13	C14	C16	-143.6(2)	C9	C4	C3	C2	175.6(3)
C12	C13	C14	C15	92.9(3)	C9	C4	C3	C8	-1.4(4)
C26	N1	C11	Ru1	8.7(3)	C28	C27	C31	C32	-176.8(3)

Table S6 Torsion Angles for 2.

A	B	C	D	Angle/˚	A	B	C	D	Angle/˚
C26	N1	C11	N2	-179.0(2)	C10	C5	C1	Ru1	123.4(3)
C26	N1	C25	C24	179.8(2)	C10	C5	C1	C2	-173.8(3)
C26	C34	C33	C32	3.6(4)	C10	C5	C1	C6	-5.8(4)
C26	C34	C35	C37	156.9(2)	C6	C1	C2	Ru1	130.1(3)
C26	C34	C35	C36	-80.1(3)	C6	C1	C2	C3	-166.8(2)
C26	C27	C31	C32	-4.0(4)	C6	C1	C2	C7	4.8(4)
C26	C27	C28	C29	-59.5(3)	C43	C42	C41	C40	163.9(9)
C26	C27	C28	C30	176.9(3)	C42	C41	C40	C39	-176.2(12)

Table S7 Hydrogen Atom Coordinates (Å×10^4) and Isotropic Displacement Parameters (Å^2×10^3) for 2.

Atom	*x*	*y*	*z*	U(eq)
H24	5845.63	891.75	7472.9	23
H19	4075.74	698.45	5239.89	27
H25	5439.55	1706.26	8376.75	25
H17	7807.86	1468.3	5391.32	28
H14	7799.06	2457.3	6844.46	24
H16A	9349.6	2236.56	5861.5	43
H16B	9666.4	2849.22	6424.61	43
H16C	8438.3	3008.83	5919.49	43
H33	6704.22	4627.37	9164.25	29
H18	6030.43	845.55	4828.65	31
H21	3281.38	1579.41	6637.72	27
H35	7525.96	3288.69	7941.93	28
H32	4968.03	4438.11	9680.79	34

Table S7 Hydrogen Atom Coordinates (Å×10[4]) and Isotropic Displacement Parameters (Å[2]×10[3]) for 2.

**Atom**	***x***	***y***	***z***	**U(eq)**
H31	3245.64	3570.51	9322.56	34
H8A	2376.32	4944.12	6947.94	53
H8B	2420.15	5662.07	6461.81	53
H8C	3517.17	5604.15	7042.38	53
H9A	5494.79	6018.64	6717.25	52
H9B	5367.44	6196.14	5995.36	52
H9C	6622.26	5732.34	6325.57	52
H28	3116.56	2148.34	8193.77	39
H15A	8340.07	1117.73	7125.19	47
H15B	9657.99	1623.3	7110.28	47
H15C	9165.02	1024.55	6557.17	47
H37A	8142.79	4695.37	8081.88	50
H37B	9332.18	4081.49	8248.12	50
H37C	8642.73	4493.42	8784.7	50
H36A	7979.65	3208.01	9257.72	48
H36B	8870.19	2767.94	8809.76	48
H36C	7389.36	2487.84	8822.01	48
H23A	3406.31	-94.73	6300.87	48
H23B	2466.65	249.1	6768.78	48
H23C	4016.79	275.79	6946.54	48
H10A	7241.85	4705.8	5671.67	57
H10B	6258.49	4731.3	5048.67	57
H10C	6773.14	3881.56	5331.39	57
H6A	3829.84	2632.15	5476.97	61

Table S7 Hydrogen Atom Coordinates (Å×10⁴) and Isotropic Displacement Parameters (Å²×10³) for 2.

Atom	*x*	*y*	*z*	U(eq)
H6B	5169.11	2892.51	5233.72	61
H6C	3813.1	3254.61	4917.57	61
H7A	2357.22	2991.53	6254.12	70
H7B	1620.87	3605.73	5765.1	70
H7C	1729.04	3804.62	6483.37	70
H22A	2195.76	1750.23	5605.2	52
H22B	1352.38	1237.15	6033.62	52
H22C	2123.53	786.73	5542.09	52
H29A	1385.51	2697.8	7527.83	70
H29B	2725.26	3124.45	7411.54	70
H29C	1723.71	3584.23	7793.61	70
H30A	2223.12	2308.24	9128.84	81
H30B	1087.44	2187.27	8571.02	81
H30C	1395.53	3069.96	8850.81	81
H43A	429.12	2983.02	4328.44	87
H43B	-946.41	3365.51	4440.39	87
H43C	100.61	3135.24	5013.37	87
H42A	531.9	4356.34	4150.78	64
H42B	1483.49	4155.72	4765.28	64
H41A	-1030.91	4870.38	4683.27	64
H41B	-384.28	4481.42	5312.89	64
H40A	1318.8	5412.28	5332.92	64
H40B	593.03	5817.4	4726.37	64
H39A	-1079.97	6216.46	5269.44	106

Table S7 Hydrogen Atom Coordinates (Å×10^4) and Isotropic Displacement Parameters (Å^2×10^3) for 2.

Atom	*x*	*y*	*z*	U(eq)
H39B	299.02	6583.79	5550.88	106
H39C	-308.27	5832.83	5878.27	106

Table S8 Atomic Occupancy for 3.

Atom	*Occupancy*	Atom	*Occupancy*	Atom	*Occupancy*
C43	0.5	H43A	0.5	H43B	0.5
H43C	0.5	C42	0.5	H42A	0.5
H42B	0.5	C41	0.5	H41A	0.5
H41B	0.5	C40	0.5	H40A	0.5
H40B	0.5	C39	0.5	H39A	0.5
H39B	0.5	H39C	0.5		

Experimental

Single crystals of $C_{40.5}H_{57}N_2OPRu$ **3** were grown from pentane at – 35 ^oC. A suitable crystal was selected and mounted on a **ROD, Synergy Custom DW system, Pilatus 200K** diffractometer. The crystal was kept at 77 K during data collection. Using Olex2 [1], the structure was solved with the ShelX [2] structure solution program.

1. Dolomanov, O.V., Bourhis, L.J., Gildea, R.J, Howard, J.A.K. & Puschmann, H. (2009), J. Appl. Cryst. 42, 339-341.

Crystal structure determination of 2

Crystal Data for $C_{40.5}H_{57}N_2OPRu$ (*M* =719.92 g/mol): monoclinic, space group P2$_1$/n (no. 14), a = 10.3207(4) Å, b = 16.5373(7) Å, c = 21.9400(8) Å, β = 97.019(4)°, V = 3716.6(3) Å^3, Z = 4, T = 77 K, μ(Mo Kα) = 0.498 mm^{-1}, $Dcalc$ = 1.287 g/cm^3, 41159 reflections measured (6.188° ≤ 2Θ ≤ 52.744°), 7594 unique (R_{int} = 0.0583, R_{sigma} = 0.0374) which were used in all calculations. The final R_1 was 0.0376 (I > 2σ(I)) and wR_2 was 0.0892 (all data).

Refinement model description

Number of restraints - 58, number of constraints - unknown.

Details:

1. Restrained distances
C43-C42 = C42-C41 = C41-C40 = C40-C39
1.52 with sigma of 0.02
C43-C41 = C42-C40 = C41-C39
2.54 with sigma of 0.04
C43-C42 ≈ C42-C41 ≈ C41-C40 ≈ C40-C39
with sigma of 0.02
2. Uiso/Uaniso restraints and constraints
C43 ≈ C42 ≈ C41 ≈ C40 ≈ C39: within 2A with sigma of 0.04 and
sigma for terminal atoms of 0.08 within 2A
Uanis(C41) = Uanis(C40) = Uanis(C42)
3. Rigid body (RIGU) restrains
C43, C42, C41, C40, C39
with sigma for 1-2 distances of 0.004 and sigma for 1-3 distances of 0.004
4. Others
Fixed Sof: C43(0.5) H43A(0.5) H43B(0.5) H43C(0.5) C42(0.5) H42A(0.5)
H42B(0.5) C41(0.5) H41A(0.5) H41B(0.5) C40(0.5) H40A(0.5) H40B(0.5) C39(0.5)
H39A(0.5) H39B(0.5) H39C(0.5)
5.a Ternary CH refined with riding coordinates:
C14(H14), C21(H21), C35(H35), C28(H28)
5.b Secondary CH2 refined with riding coordinates:
C42(H42A,H42B), C41(H41A,H41B), C40(H40A,H40B)
5.c Aromatic/amide H refined with riding coordinates:
C24(H24), C19(H19), C25(H25), C17(H17), C33(H33), C18(H18), C32(H32), C31(H31)
5.d Idealised Me refined as rotating group:
C16(H16A,H16B,H16C), C8(H8A,H8B,H8C), C9(H9A,H9B,H9C), C15(H15A,H15B,H15C), C37(H37A,H37B,H37C), C36(H36A,H36B,H36C), C23(H23A,H23B,H23C), C10(H10A,H10B, H10C), C6(H6A,H6B,H6C), C7(H7A,H7B,H7C), C22(H22A,H22B,H22C), C29(H29A,H29B, H29C), C30(H30A,H30B,H30C), C43(H43A,H43B,H43C), C39(H39A,H39B,H39C)

phosphaazaallene (3)

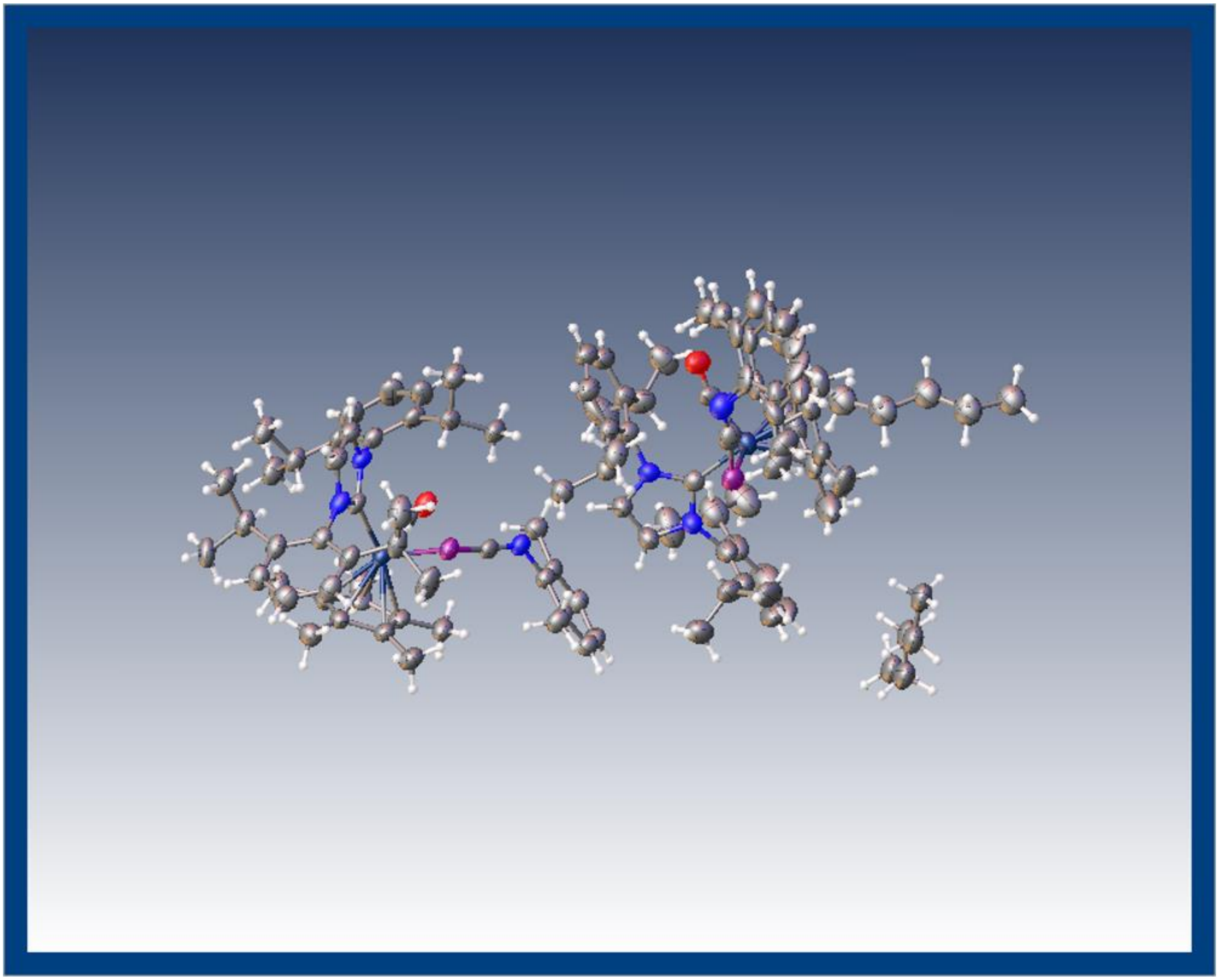

Table S9 Crystal data and structure refinement for 3.

Identification code	TSD41_Tilley
Empirical formula	$C_{48.25}H_{63}N_3OPRu$
Formula weight	833.05
Temperature/K	293(2)
Crystal system	triclinic
Space group	P-1
a/Å	11.9764(3)
b/Å	19.1730(5)

c/Å	22.4039(6)
α/°	75.824(2)
β/°	86.064(2)
γ/°	79.796(2)
Volume/$Å^3$	4907.2(2)
Z	4
ρ_{calc}g/cm^3	1.128
μ/mm^{-1}	3.145
F(000)	1762.0
Crystal size/mm^3	0.2 × 0.05 × 0.03
Radiation	Cu Kα (λ = 1.54184)
2Θ range for data collection/°	5.522 to 149.008
Index ranges	-14 ≤ h ≤ 14, -23 ≤ k ≤ 23, -27 ≤ l ≤ 27
Reflections collected	98678
Independent reflections	19975 [R_{int} = 0.1226, R_{sigma} = 0.0774]
Data/restraints/parameters	19975/360/1089
Goodness-of-fit on F^2	1.051
Final R indexes [I>=2σ (I)]	R_1 = 0.0800, wR_2 = 0.2198
Final R indexes [all data]	R_1 = 0.1115, wR_2 = 0.2516
Largest diff. peak/hole / e $Å^{-3}$	1.79/-1.28

Table S10 Fractional Atomic Coordinates (×10^4) and Equivalent Isotropic Displacement Parameters ($Å^2$×10^3) for 3. U_{eq} is defined as 1/3 of the trace of the orthogonalised U_{IJ} tensor.

**Atom**	***x***	***y***	***z***	**U(eq)**
Ru2	1642.8(3)	7953.7(2)	4423.4(2)	40.50(15)
Ru1	6750.2(4)	6626.6(3)	8861.5(2)	51.34(17)

Table S10 Fractional Atomic Coordinates (×10^4) and Equivalent Isotropic Displacement Parameters (Å^2×10^3) for 3. U_{eq} is defined as 1/3 of the trace of the orthogonalised U_{IJ} tensor.

Atom	*x*	*y*	*z*	U(eq)
P2	3419.2(11)	7478.1(8)	4943.6(7)	45.5(3)
P1	8121.3(14)	6321.1(10)	8095.4(8)	59.6(4)
O2	537(3)	8115(2)	5625(2)	56.1(10)
N4	3330(4)	8154(2)	5958(2)	45.3(10)
N2	5156(4)	7091(2)	7732(2)	47.1(10)
N5	2026(4)	6386(2)	4125(2)	44.1(10)
N6	1023(4)	6410(2)	4952(2)	41.6(9)
N3	5800(4)	8030(3)	7816(2)	52.6(11)
O1	5832(4)	5249(3)	8957(2)	71.4(13)
C57	948(4)	7962(3)	5179(3)	45.9(12)
C55	3582(5)	8833(3)	6010(2)	43.4(11)
C58	1519(4)	6862(3)	4474(2)	40.0(11)
C59	2564(5)	6526(3)	3511(3)	46.2(12)
C53	4582(5)	9081(3)	5759(3)	44.6(11)
C60	3743(5)	6466(3)	3465(3)	53.5(14)
C50	3025(5)	9887(3)	6429(3)	48.8(12)
C12	4746(5)	6404(3)	7822(3)	50.6(13)
C24	4780(5)	7646(3)	7232(3)	50.8(13)
C73	243(5)	6603(3)	5429(3)	48.0(12)
C74	620(5)	6420(3)	6036(3)	53.5(14)
C49	2807(5)	9217(3)	6360(3)	46.6(12)
C51	3991(5)	10148(3)	6165(3)	54.6(14)
C88	1819(5)	9128(3)	4193(3)	50.8(13)
C56	3350(4)	7906(3)	5499(3)	43.7(11)

Table S10 Fractional Atomic Coordinates (×10[4]) and Equivalent Isotropic Displacement Parameters (Å[2]×10[3]) for 3. U_{eq} is defined as 1/3 of the trace of the orthogonalised U_{IJ} tensor.

Atom	*x*	*y*	*z*	U(eq)
C52	4763(5)	9753(3)	5845(3)	50.9(13)
C82	-1267(5)	7087(3)	4615(3)	53.3(13)
C89	660(5)	9074(3)	4100(3)	50.7(13)
N1	8437(5)	4757(3)	8356(3)	66.9(14)
C48	1771(5)	8918(4)	6656(3)	57.2(14)
C61	4495(5)	6257(4)	4026(3)	60.9(16)
C72	1260(5)	5685(3)	4916(3)	50.4(13)
C67	1894(5)	6612(3)	3003(3)	52.4(13)
C54	5466(5)	8637(3)	5436(3)	53.7(14)
C71	1881(5)	5663(3)	4401(3)	52.5(13)
C11	5817(5)	7309(3)	8116(3)	49.4(12)
C90	-362(6)	8615(4)	3321(3)	62.3(16)
C68	624(5)	6618(3)	3050(3)	54.5(14)
C75	1837(5)	6117(3)	6210(3)	54.9(14)
C25	5189(6)	8227(3)	7274(3)	56.7(14)
C10	6095(5)	5814(3)	8838(3)	53.5(14)
C85	656(5)	8699(3)	3617(3)	52.0(13)
C20	5199(5)	5921(3)	7465(3)	52.1(13)
C81	-875(4)	6921(3)	5268(3)	50.6(13)
C13	3805(5)	6295(4)	8239(3)	56.1(14)
C87	2500(5)	8827(3)	3742(3)	50.3(13)
C21	6098(5)	6072(3)	6961(3)	52.6(13)
C23	5565(5)	6344(4)	6339(3)	58.4(15)
C66	2445(6)	6666(4)	2428(3)	59.2(15)

Table S10 Fractional Atomic Coordinates (×10^4) and Equivalent Isotropic Displacement Parameters (Å^2×10^3) for 3. U_{eq} is defined as 1/3 of the trace of the orthogonalised U_{IJ} tensor.

Atom	*x*	*y*	*z*	U(eq)
C41	7753(5)	6065(3)	9706(3)	57.1(14)
C77	2252(6)	6526(4)	6635(3)	61.1(16)
C64	4249(6)	6532(4)	2877(3)	61.3(16)
C78	-189(5)	6536(4)	6500(3)	65.7(17)
C86	1809(5)	8558(3)	3394(3)	50.2(13)
C83	-2322(5)	7680(4)	4495(4)	64.7(17)
C65	3604(6)	6635(4)	2364(3)	65.4(17)
C84	-1497(6)	6400(4)	4445(4)	64.9(17)
C91	2180(7)	8327(4)	2809(3)	64.0(16)
C94	-347(6)	9399(4)	4429(4)	66.0(17)
C93	2185(6)	9523(3)	4620(3)	59.1(15)
C26	6262(7)	8598(3)	7981(3)	65.5(18)
C92	3732(6)	8851(4)	3625(3)	65.7(17)
C70	348(5)	5850(4)	3123(4)	63.5(16)
C18	3872(6)	5141(4)	7982(3)	65.0(17)
C19	4743(6)	5274(4)	7558(3)	63.6(16)
C42	6611(6)	6276(4)	9892(3)	70.4(17)
C80	-1621(5)	7044(4)	5757(3)	64.7(17)
C76	2006(7)	5294(4)	6513(4)	72.5(19)
C17	3393(6)	5652(4)	8302(3)	64.5(17)
C79	-1283(6)	6850(5)	6356(3)	72(2)
C35	8031(7)	8321(5)	7324(4)	79(2)
C22	7002(6)	5397(4)	6956(4)	66.5(17)
C7	9126(13)	4111(8)	8691(10)	57(4)

Table S10 Fractional Atomic Coordinates (×10[4]) and Equivalent Isotropic Displacement Parameters (Å[2]×10[3]) for 3. U_{eq} is defined as 1/3 of the trace of the orthogonalised U_{IJ} tensor.

Atom	*x*	*y*	*z*	U(eq)
C34	7291(7)	8761(4)	7735(3)	75(2)
C14	3232(6)	6869(4)	8565(4)	70.7(18)
C9	8344(5)	5423(4)	8280(3)	62.0(16)
C40	8212(6)	6703(4)	9438(3)	66.6(16)
C69	-28(6)	7134(4)	2497(3)	65.2(17)
C27	5514(9)	9055(4)	8304(4)	83(2)
C15	2906(7)	6550(5)	9224(4)	84(2)
C36	7997(8)	8752(5)	6658(4)	91(3)
C63	4988(7)	5448(5)	4153(4)	82(2)
C38	6340(7)	7042(4)	9727(3)	76.6(18)
C62	5441(6)	6714(5)	3944(4)	82(2)
C2	8559(18)	3546(9)	9015(10)	66(3)
C39	7336(7)	7315(4)	9455(3)	75.8(18)
C28	4326(9)	8898(5)	8542(4)	91(3)
C46	8386(7)	5308(5)	9845(4)	80(2)
C37	9279(7)	8042(6)	7528(5)	102(3)
C1	7270(20)	3621(19)	9074(15)	70(5)
C6	10309(13)	4036(12)	8729(11)	70(5)
C31	5905(12)	9655(5)	8407(5)	104(3)
C30	3901(11)	9208(6)	9121(5)	114(4)
C32	6944(13)	9811(5)	8176(5)	114(4)
C5	10940(12)	3372(10)	9031(10)	80(5)
C33	7656(10)	9374(5)	7837(5)	102(3)
C16	2162(9)	7278(6)	8219(4)	105(3)

Table S10 Fractional Atomic Coordinates (×10^4) and Equivalent Isotropic Displacement Parameters (Å^2×10^3) for 3. U_{eq} is defined as 1/3 of the trace of the orthogonalised U_{IJ} tensor.

Atom	*x*	*y*	*z*	U(eq)
C45	9427(7)	6709(6)	9264(4)	102(3)
C44	7517(12)	8099(5)	9340(5)	123(4)
C29	3435(10)	9247(7)	8060(5)	118(4)
C43	5249(10)	7476(7)	9921(5)	134(5)
C4	10363(17)	2803(11)	9316(10)	93(6)
C3	9177(17)	2888(9)	9326(10)	79(5)
C8	10830(20)	4681(14)	8355(15)	90(5)
C100	3950(20)	8411(19)	11109(14)	84(4)
C101	5150(20)	8302(19)	11296(11)	84(4)
C102	5420(20)	8273(19)	11940(11)	84(4)
C103	6580(20)	8300(19)	12096(12)	84(4)
C104	6810(30)	8414(19)	12697(12)	84(4)
C99	10480(30)	8905(13)	9268(14)	84(4)
C98	10250(20)	9699(12)	8927(13)	84(4)
C97	9200(20)	10146(11)	9149(13)	84(4)
C96	9000(20)	10955(12)	8877(14)	84(4)
C95	7860(20)	11371(15)	9027(15)	84(4)
C8A	10800(40)	4970(20)	8150(20)	90(5)
C6A	10360(30)	4265(19)	8553(17)	70(6)
C7A	9240(30)	4247(15)	8756(16)	70(8)
C2A	8850(30)	3607(16)	9079(18)	66(3)
C3A	9610(30)	2984(18)	9335(14)	82(7)
C4A	10760(20)	3015(13)	9189(14)	75(7)
C5A	11110(30)	3681(16)	8885(13)	83(7)

Table S10 Fractional Atomic Coordinates (×10[4]) and Equivalent Isotropic Displacement Parameters (Å^2×10^3) for 3. U_{eq} is defined as 1/3 of the trace of the orthogonalised U_{IJ} tensor.

Atom	*x*	*y*	*z*	U(eq)
C1A	7620(40)	3620(30)	9150(30)	70(5)
C47	5836(7)	5768(6)	10255(4)	97(3)

Table S11 Anisotropic Displacement Parameters (Å^2×10^3) for 3. The Anisotropic displacement factor exponent takes the form: $-2\pi^2[h^2a^{*2}U_{11}+2hka^*b^*U_{12}+\ldots]$.

Atom	U_{11}	U_{22}	U_{33}	U_{23}	U_{13}	U_{12}
Ru2	37.3(2)	40.6(2)	46.7(3)	-11.86(16)	-6.24(16)	-10.69(15)
Ru1	52.6(3)	59.3(3)	41.3(3)	-7.68(19)	-6.70(18)	-11.0(2)
P2	38.8(7)	49.8(7)	52.1(8)	-16.2(6)	-6.3(6)	-11.2(6)
P1	53.0(9)	72.5(10)	55.3(9)	-10.9(8)	-3.3(7)	-20.8(8)
O2	47(2)	65(2)	64(3)	-27(2)	2.9(19)	-16.4(19)
N4	41(2)	48(2)	50(3)	-13(2)	-4.6(19)	-13.8(19)
N2	48(3)	47(2)	44(2)	-3.9(19)	-1.3(19)	-13(2)
N5	38(2)	41(2)	59(3)	-23(2)	-2.6(19)	-8.4(18)
N6	37(2)	39(2)	52(3)	-10.9(18)	-3.6(18)	-12.9(17)
N3	62(3)	49(3)	47(3)	-7(2)	-11(2)	-12(2)
O1	59(3)	73(3)	76(3)	4(2)	-11(2)	-21(2)
C57	37(3)	44(3)	63(3)	-17(3)	-2(2)	-15(2)
C55	43(3)	43(3)	47(3)	-10(2)	-6(2)	-12(2)
C58	33(2)	42(2)	52(3)	-15(2)	-7(2)	-14(2)
C59	47(3)	46(3)	52(3)	-20(2)	-1(2)	-12(2)
C53	42(3)	44(3)	49(3)	-9(2)	-6(2)	-14(2)
C60	45(3)	61(3)	64(4)	-27(3)	-2(3)	-19(3)
C50	44(3)	47(3)	57(3)	-15(2)	-8(2)	-6(2)
C12	49(3)	46(3)	55(3)	-2(2)	-8(2)	-11(2)

Table S11 Anisotropic Displacement Parameters ($Å^2×10^3$) for 3. The Anisotropic displacement factor exponent takes the form: $-2π^2[h^2a^{*2}U_{11}+2hka^*b^*U_{12}+…]$.

Atom	U_{11}	U_{22}	U_{33}	U_{23}	U_{13}	U_{12}
C24	56(3)	51(3)	46(3)	-7(2)	-9(2)	-15(3)
C73	42(3)	55(3)	53(3)	-17(3)	1(2)	-19(2)
C74	38(3)	62(3)	64(4)	-14(3)	3(2)	-22(3)
C49	40(3)	49(3)	52(3)	-9(2)	-13(2)	-10(2)
C51	59(3)	46(3)	62(4)	-12(3)	-9(3)	-16(3)
C88	62(3)	35(3)	58(3)	-7(2)	-9(3)	-18(2)
C56	33(2)	47(3)	51(3)	-6(2)	-7(2)	-14(2)
C52	48(3)	50(3)	56(3)	-8(3)	-8(2)	-16(2)
C82	40(3)	58(3)	66(4)	-18(3)	-10(3)	-11(2)
C89	53(3)	29(2)	65(4)	-4(2)	-20(3)	4(2)
N1	61(3)	74(4)	69(4)	-19(3)	-5(3)	-15(3)
C48	50(3)	61(4)	62(4)	-12(3)	0(3)	-16(3)
C61	39(3)	84(4)	71(4)	-40(4)	-5(3)	-10(3)
C72	49(3)	44(3)	59(3)	-7(2)	-8(3)	-15(2)
C67	49(3)	55(3)	61(4)	-23(3)	-9(3)	-12(3)
C54	43(3)	57(3)	65(4)	-17(3)	1(3)	-14(3)
C71	46(3)	45(3)	69(4)	-15(3)	-5(3)	-10(2)
C11	52(3)	53(3)	45(3)	-13(2)	4(2)	-15(3)
C90	59(4)	58(4)	76(4)	-24(3)	-21(3)	-7(3)
C68	46(3)	63(3)	63(4)	-29(3)	-10(3)	-10(3)
C75	49(3)	63(3)	52(3)	-2(3)	-8(3)	-21(3)
C25	69(4)	55(3)	46(3)	-6(3)	-9(3)	-14(3)
C10	39(3)	61(4)	54(3)	4(3)	-2(2)	-16(3)
C85	55(3)	48(3)	54(3)	-11(3)	-15(3)	-7(3)

Table S11 Anisotropic Displacement Parameters (Å^2×10^3) for 3. The Anisotropic displacement factor exponent takes the form: $-2\pi^2[h^2a^{*2}U_{11}+2hka^*b^*U_{12}+\ldots]$.

Atom	U_{11}	U_{22}	U_{33}	U_{23}	U_{13}	U_{12}
C20	50(3)	52(3)	55(3)	-8(3)	-12(3)	-14(3)
C81	33(3)	58(3)	64(4)	-14(3)	-1(2)	-17(2)
C13	48(3)	65(4)	53(3)	-2(3)	-6(3)	-15(3)
C87	58(3)	47(3)	49(3)	-5(2)	-6(3)	-24(3)
C21	51(3)	53(3)	56(3)	-14(3)	-11(3)	-7(3)
C23	54(3)	64(4)	60(4)	-15(3)	-4(3)	-13(3)
C66	58(4)	70(4)	58(4)	-28(3)	-4(3)	-15(3)
C41	54(3)	71(3)	44(3)	-5(3)	-11(2)	-11(3)
C77	55(3)	68(4)	64(4)	-7(3)	-13(3)	-26(3)
C64	49(3)	75(4)	71(4)	-34(3)	4(3)	-17(3)
C78	48(3)	97(5)	52(4)	-9(3)	2(3)	-25(3)
C86	63(4)	45(3)	43(3)	-2(2)	-10(3)	-20(3)
C83	39(3)	73(4)	85(5)	-26(4)	-10(3)	-6(3)
C65	64(4)	76(4)	64(4)	-30(3)	10(3)	-18(3)
C84	49(3)	61(4)	92(5)	-27(3)	-19(3)	-10(3)
C91	78(5)	65(4)	50(3)	-9(3)	-6(3)	-21(3)
C94	55(4)	60(4)	88(5)	-32(3)	-18(3)	4(3)
C93	64(4)	49(3)	66(4)	-8(3)	-20(3)	-17(3)
C26	98(5)	50(3)	53(4)	-6(3)	-18(3)	-25(3)
C92	67(4)	67(4)	69(4)	-12(3)	-1(3)	-33(3)
C70	45(3)	67(4)	86(5)	-29(4)	-11(3)	-11(3)
C18	65(4)	61(4)	67(4)	-1(3)	-10(3)	-24(3)
C19	61(4)	56(4)	74(4)	-11(3)	-7(3)	-16(3)
C42	67(4)	100(4)	35(3)	0(3)	-10(3)	-8(3)

Table S11 Anisotropic Displacement Parameters (Å^2×10^3) for 3. The Anisotropic displacement factor exponent takes the form: $-2\pi^2[h^2a^{*2}U_{11}+2hka^*b^*U_{12}+\ldots]$.

Atom	U_{11}	U_{22}	U_{33}	U_{23}	U_{13}	U_{12}
C80	36(3)	84(5)	76(4)	-21(4)	6(3)	-17(3)
C76	69(4)	66(4)	83(5)	-10(4)	-17(4)	-20(3)
C17	57(4)	70(4)	63(4)	2(3)	-9(3)	-24(3)
C79	47(3)	112(6)	61(4)	-23(4)	17(3)	-27(4)
C35	69(5)	88(5)	79(5)	4(4)	-12(4)	-40(4)
C22	53(4)	69(4)	82(5)	-32(4)	-12(3)	2(3)
C7	64(6)	68(7)	54(7)	-40(6)	-17(5)	-7(5)
C34	95(6)	75(5)	59(4)	0(3)	-22(4)	-37(4)
C14	64(4)	71(4)	74(5)	-11(4)	9(3)	-16(3)
C9	45(3)	82(5)	63(4)	-21(3)	-1(3)	-15(3)
C40	70(4)	88(4)	50(3)	-14(3)	-16(3)	-33(3)
C69	60(4)	77(4)	67(4)	-31(3)	-27(3)	-5(3)
C27	126(7)	55(4)	71(5)	-10(3)	-24(5)	-20(4)
C15	70(5)	107(6)	61(4)	-7(4)	4(4)	1(4)
C36	91(6)	101(6)	76(5)	8(5)	-8(4)	-42(5)
C63	69(5)	87(5)	95(6)	-40(5)	-23(4)	1(4)
C38	83(4)	94(4)	50(4)	-20(3)	-20(3)	6(3)
C62	40(3)	116(6)	107(6)	-54(5)	1(4)	-21(4)
C2	78(9)	80(5)	49(6)	-25(4)	-25(6)	-15(5)
C39	111(5)	72(4)	55(4)	-21(3)	-33(3)	-23(3)
C28	107(7)	77(5)	84(6)	-20(4)	-14(5)	6(5)
C46	80(5)	89(5)	68(5)	-16(4)	-29(4)	0(4)
C37	69(5)	134(8)	98(6)	15(6)	-19(5)	-56(5)
C1	78(18)	79(5)	64(9)	-23(6)	-5(11)	-28(12)

Table S11 Anisotropic Displacement Parameters (Å^2×10^3) for 3. The Anisotropic displacement factor exponent takes the form: -2π^2[h^{2}a*^{2}U$_{11}$+2hka*b*U$_{12}$+…].

Atom	U_{11}	U_{22}	U_{33}	U_{23}	U_{13}	U_{12}
C6	58(7)	90(11)	62(13)	-19(8)	-21(6)	-6(6)
C31	169(11)	61(5)	88(6)	-16(4)	-21(7)	-30(6)
C30	152(10)	87(6)	93(7)	-27(5)	-8(7)	16(6)
C32	189(13)	69(5)	98(7)	-10(5)	-44(8)	-56(7)
C5	76(8)	47(9)	135(15)	-49(10)	-33(8)	-8(7)
C33	142(9)	87(6)	86(6)	4(5)	-30(6)	-69(6)
C16	109(7)	105(7)	75(5)	-6(5)	7(5)	29(6)
C45	75(5)	164(9)	85(6)	-32(6)	-6(4)	-65(5)
C44	204(13)	79(5)	98(7)	-16(5)	-64(8)	-40(6)
C29	116(8)	139(9)	96(7)	-44(7)	-30(6)	21(7)
C43	122(8)	175(11)	90(7)	-55(7)	-28(6)	56(8)
C4	87(10)	83(10)	106(12)	-6(9)	-40(10)	-18(8)
C3	90(10)	78(7)	75(10)	-21(6)	-22(9)	-15(7)
C8	60(5)	99(18)	120(20)	-37(12)	2(10)	-30(12)
C100	98(10)	80(8)	77(8)	-28(7)	-3(8)	-7(8)
C101	98(10)	80(8)	77(8)	-28(7)	-3(8)	-7(8)
C102	98(10)	80(8)	77(8)	-28(7)	-3(8)	-7(8)
C103	98(10)	80(8)	77(8)	-28(7)	-3(8)	-7(8)
C104	98(10)	80(8)	77(8)	-28(7)	-3(8)	-7(8)
C99	90(9)	107(10)	70(8)	-21(7)	-17(7)	-48(8)
C98	90(9)	107(10)	70(8)	-21(7)	-17(7)	-48(8)
C97	90(9)	107(10)	70(8)	-21(7)	-17(7)	-48(8)
C96	90(9)	107(10)	70(8)	-21(7)	-17(7)	-48(8)
C95	90(9)	107(10)	70(8)	-21(7)	-17(7)	-48(8)

Table S11 Anisotropic Displacement Parameters (Å^2×10^3) for 3. The Anisotropic displacement factor exponent takes the form: $-2\pi^2[h^2a^{*2}U_{11}+2hka^*b^*U_{12}+…]$.

Atom	U_{11}	U_{22}	U_{33}	U_{23}	U_{13}	U_{12}
C8A	60(5)	99(18)	120(20)	-37(12)	2(10)	-30(12)
C6A	91(12)	71(14)	46(16)	-11(9)	-23(9)	-1(9)
C7A	91(12)	79(11)	52(12)	-31(11)	-27(8)	-15(10)
C2A	78(9)	80(5)	49(6)	-25(4)	-25(6)	-15(5)
C3A	92(15)	98(11)	64(13)	-23(9)	-51(14)	-10(10)
C4A	98(13)	43(12)	99(18)	-42(10)	-30(13)	-7(10)
C5A	111(15)	50(14)	91(16)	-15(11)	-55(12)	-6(10)
C1A	78(18)	79(5)	64(9)	-23(6)	-5(11)	-28(12)
C47	69(5)	155(9)	52(4)	8(5)	-1(4)	-28(5)

Table S12 Bond Lengths for 3.

Atom	Atom	Length/Å	Atom	Atom	Length/Å
Ru2	P2	2.4184(14)	C68	C70	1.533(9)
Ru2	C57	1.836(6)	C68	C69	1.543(9)
Ru2	C58	2.099(5)	C75	C77	1.529(9)
Ru2	C88	2.227(5)	C75	C76	1.541(9)
Ru2	C89	2.232(5)	C85	C86	1.439(9)
Ru2	C85	2.265(6)	C20	C21	1.517(9)
Ru2	C87	2.308(5)	C20	C19	1.407(8)
Ru2	C86	2.326(6)	C81	C80	1.404(9)
Ru1	P1	2.3979(18)	C13	C17	1.380(9)
Ru1	C11	2.102(6)	C13	C14	1.507(10)
Ru1	C10	1.877(6)	C87	C86	1.413(8)
Ru1	C41	2.244(6)	C87	C92	1.487(9)

Table S12 Bond Lengths for 3.

Atom	Atom	Length/Å	Atom	Atom	Length/Å
Ru1	C42	2.245(6)	C21	C23	1.507(9)
Ru1	C40	2.291(6)	C21	C22	1.536(9)
Ru1	C38	2.264(6)	C66	C65	1.377(9)
Ru1	C39	2.306(6)	C41	C42	1.420(8)
P2	C56	1.640(6)	C41	C40	1.413(8)
P1	C9	1.647(8)	C41	C46	1.482(10)
O2	C57	1.163(7)	C64	C65	1.384(10)
N4	C55	1.421(7)	C78	C79	1.366(10)
N4	C56	1.231(7)	C86	C91	1.500(9)
N2	C12	1.449(7)	C26	C34	1.371(11)
N2	C24	1.381(7)	C26	C27	1.427(12)
N2	C11	1.390(7)	C18	C19	1.376(10)
N5	C58	1.379(7)	C18	C17	1.376(11)
N5	C59	1.463(7)	C42	C38	1.406(9)
N5	C71	1.408(7)	C42	C47	1.517(11)
N6	C58	1.375(7)	C80	C79	1.373(10)
N6	C73	1.444(7)	C35	C34	1.533(13)
N6	C72	1.389(7)	C35	C36	1.518(11)
N3	C11	1.378(7)	C35	C37	1.555(11)
N3	C25	1.398(8)	C7	C2	1.402(12)
N3	C26	1.437(8)	C7	C6	1.405(12)
O1	C10	1.145(7)	C34	C33	1.396(11)
C55	C53	1.399(7)	C14	C15	1.506(10)
C55	C49	1.398(8)	C14	C16	1.530(12)
C59	C60	1.395(8)	C40	C39	1.435(9)

Table S12 Bond Lengths for 3.

Atom	Atom	Length/Å	Atom	Atom	Length/Å
C59	C67	1.397(8)	C40	C45	1.482(10)
C53	C52	1.405(8)	C27	C28	1.540(14)
C53	C54	1.497(8)	C27	C31	1.389(12)
C60	C61	1.527(9)	C38	C39	1.427(9)
C60	C64	1.399(9)	C38	C43	1.517(12)
C50	C49	1.402(8)	C2	C1	1.53(3)
C50	C51	1.380(8)	C2	C3	1.391(12)
C12	C20	1.385(9)	C39	C44	1.513(11)
C12	C13	1.424(8)	C28	C30	1.574(13)
C24	C25	1.320(8)	C28	C29	1.517(13)
C73	C74	1.403(9)	C6	C5	1.398(13)
C73	C81	1.402(8)	C6	C8	1.52(2)
C74	C75	1.510(8)	C31	C32	1.369(17)
C74	C78	1.403(9)	C32	C33	1.406(17)
C49	C48	1.502(8)	C5	C4	1.394(13)
C51	C52	1.365(9)	C4	C3	1.399(13)
C88	C89	1.443(8)	C100	C101	1.489(13)
C88	C87	1.422(9)	C101	C102	1.484(13)
C88	C93	1.487(8)	C102	C103	1.470(13)
C82	C81	1.507(9)	C103	C104	1.467(13)
C82	C83	1.533(9)	C99	C98	1.513(13)
C82	C84	1.532(8)	C98	C97	1.513(13)
C89	C85	1.439(8)	C97	C96	1.502(13)
C89	C94	1.491(9)	C96	C95	1.521(13)
N1	C7	1.430(13)	C8A	C6A	1.59(3)

Table S12 Bond Lengths for 3.

Atom	Atom	Length/Å	Atom	Atom	Length/Å
N1	C9	1.232(9)	C6A	C7A	1.396(15)
N1	C7A	1.425(17)	C6A	C5A	1.393(14)
C61	C63	1.523(10)	C7A	C2A	1.400(14)
C61	C62	1.525(9)	C2A	C3A	1.390(14)
C72	C71	1.336(9)	C2A	C1A	1.47(5)
C67	C68	1.516(8)	C3A	C4A	1.400(15)
C67	C66	1.396(9)	C4A	C5A	1.412(14)
C90	C85	1.479(8)			

Table S13 Bond Angles for 3.

Atom	Atom	Atom	Angle/˚	Atom	Atom	Atom	Angle/˚
C57	Ru2	P2	88.25(18)	N3	C11	N2	100.9(5)
C57	Ru2	C58	94.5(2)	C67	C68	C70	111.3(5)
C57	Ru2	C88	95.0(2)	C67	C68	C69	113.9(6)
C57	Ru2	C89	86.4(2)	C70	C68	C69	108.5(5)
C57	Ru2	C85	114.3(2)	C74	C75	C77	111.3(6)
C57	Ru2	C87	130.6(2)	C74	C75	C76	112.1(5)
C57	Ru2	C86	147.3(2)	C77	C75	C76	109.9(5)
C58	Ru2	P2	86.14(14)	C24	C25	N3	106.8(5)
C58	Ru2	C88	170.0(2)	O1	C10	Ru1	163.1(5)
C58	Ru2	C89	140.1(2)	C89	C85	Ru2	70.1(3)
C58	Ru2	C85	110.2(2)	C89	C85	C90	125.8(6)
C58	Ru2	C87	134.6(2)	C89	C85	C86	107.2(5)
C58	Ru2	C86	108.9(2)	C90	C85	Ru2	130.6(5)
C88	Ru2	P2	97.36(16)	C86	C85	Ru2	74.1(3)

Table S13 Bond Angles for 3.

Atom	Atom	Atom	Angle/˚	Atom	Atom	Atom	Angle/˚
C88	Ru2	C89	37.8(2)	C86	C85	C90	125.9(6)
C88	Ru2	C85	62.6(2)	C12	C20	C21	123.8(5)
C88	Ru2	C87	36.5(2)	C12	C20	C19	117.2(6)
C88	Ru2	C86	61.1(2)	C19	C20	C21	118.9(6)
C89	Ru2	P2	133.74(15)	C73	C81	C82	122.2(5)
C89	Ru2	C85	37.3(2)	C73	C81	C80	115.8(6)
C89	Ru2	C87	60.9(2)	C80	C81	C82	122.0(5)
C89	Ru2	C86	61.0(2)	C12	C13	C14	122.2(6)
C85	Ru2	P2	149.91(17)	C17	C13	C12	116.5(6)
C85	Ru2	C87	60.6(2)	C17	C13	C14	121.2(6)
C85	Ru2	C86	36.5(2)	C88	C87	Ru2	68.7(3)
C87	Ru2	P2	89.77(16)	C88	C87	C92	124.8(5)
C87	Ru2	C86	35.51(19)	C86	C87	Ru2	72.9(3)
C86	Ru2	P2	115.15(16)	C86	C87	C88	109.5(5)
C11	Ru1	P1	85.53(16)	C86	C87	C92	125.5(6)
C11	Ru1	C41	170.7(2)	C92	C87	Ru2	128.5(4)
C11	Ru1	C42	139.7(2)	C20	C21	C22	112.3(5)
C11	Ru1	C40	136.4(2)	C23	C21	C20	110.7(5)
C11	Ru1	C38	111.1(2)	C23	C21	C22	109.3(5)
C11	Ru1	C39	110.1(2)	C65	C66	C67	121.3(6)
C10	Ru1	P1	88.82(19)	C42	C41	Ru1	71.6(3)
C10	Ru1	C11	93.4(2)	C42	C41	C46	125.2(6)
C10	Ru1	C41	94.9(2)	C40	C41	Ru1	73.7(3)
C10	Ru1	C42	87.3(3)	C40	C41	C42	108.1(6)
C10	Ru1	C40	129.7(3)	C40	C41	C46	126.2(6)

Table S13 Bond Angles for 3.

Atom	Atom	Atom	Angle/˚	Atom	Atom	Atom	Angle/˚
C10	Ru1	C38	114.8(3)	C46	C41	Ru1	126.5(5)
C10	Ru1	C39	147.5(3)	C65	C64	C60	121.0(6)
C41	Ru1	P1	98.86(17)	C79	C78	C74	120.6(6)
C41	Ru1	C42	36.9(2)	C85	C86	Ru2	69.4(3)
C41	Ru1	C40	36.3(2)	C85	C86	C91	125.8(5)
C41	Ru1	C38	61.3(2)	C87	C86	Ru2	71.6(3)
C41	Ru1	C39	60.6(2)	C87	C86	C85	108.1(5)
C42	Ru1	P1	134.77(18)	C87	C86	C91	124.8(6)
C42	Ru1	C40	60.8(3)	C91	C86	Ru2	135.0(4)
C42	Ru1	C38	36.3(2)	C66	C65	C64	120.0(6)
C42	Ru1	C39	60.3(3)	C34	C26	N3	119.8(7)
C40	Ru1	P1	88.69(19)	C34	C26	C27	122.6(7)
C40	Ru1	C39	36.4(2)	C27	C26	N3	116.5(7)
C38	Ru1	P1	149.1(2)	C17	C18	C19	120.8(6)
C38	Ru1	C40	61.1(3)	C18	C19	C20	120.5(7)
C38	Ru1	C39	36.4(2)	C41	C42	Ru1	71.5(3)
C39	Ru1	P1	114.2(2)	C41	C42	C47	125.8(7)
C56	P2	Ru2	104.29(19)	C38	C42	Ru1	72.6(3)
C9	P1	Ru1	103.0(2)	C38	C42	C41	108.9(6)
C56	N4	C55	129.6(5)	C38	C42	C47	125.2(7)
C24	N2	C12	118.3(5)	C47	C42	Ru1	125.6(5)
C24	N2	C11	112.6(5)	C79	C80	C81	122.1(6)
C11	N2	C12	128.8(5)	C18	C17	C13	121.7(6)
C58	N5	C59	130.6(4)	C78	C79	C80	120.8(6)
C58	N5	C71	111.7(5)	C34	C35	C37	115.0(7)

Table S13 Bond Angles for 3.

Atom	Atom	Atom	Angle/˚	Atom	Atom	Atom	Angle/˚
C71	N5	C59	117.4(4)	C36	C35	C34	110.3(7)
C58	N6	C73	128.7(4)	C36	C35	C37	110.5(7)
C58	N6	C72	112.4(4)	C2	C7	N1	116.8(12)
C72	N6	C73	118.6(4)	C2	C7	C6	119.9(12)
C11	N3	C25	112.4(5)	C6	C7	N1	123.3(13)
C11	N3	C26	130.9(5)	C26	C34	C35	122.6(6)
C25	N3	C26	116.6(5)	C26	C34	C33	118.8(9)
O2	C57	Ru2	166.0(5)	C33	C34	C35	118.5(8)
C53	C55	N4	121.7(5)	C13	C14	C16	108.6(7)
C49	C55	N4	116.1(5)	C15	C14	C13	112.6(6)
C49	C55	C53	122.0(5)	C15	C14	C16	108.9(7)
N5	C58	Ru2	131.5(4)	N1	C9	P1	172.2(6)
N6	C58	Ru2	125.5(4)	C41	C40	Ru1	70.1(3)
N6	C58	N5	102.2(4)	C41	C40	C39	107.4(6)
C60	C59	N5	118.3(5)	C41	C40	C45	124.0(7)
C60	C59	C67	122.8(5)	C39	C40	Ru1	72.4(3)
C67	C59	N5	118.3(5)	C39	C40	C45	128.0(7)
C55	C53	C52	117.4(5)	C45	C40	Ru1	129.5(5)
C55	C53	C54	122.1(5)	C26	C27	C28	122.5(7)
C52	C53	C54	120.4(5)	C31	C27	C26	117.3(9)
C59	C60	C61	123.0(6)	C31	C27	C28	120.2(9)
C59	C60	C64	117.4(6)	C42	C38	Ru1	71.1(3)
C64	C60	C61	119.3(6)	C42	C38	C39	107.5(6)
C51	C50	C49	120.2(5)	C42	C38	C43	124.3(9)
C20	C12	N2	119.6(5)	C39	C38	Ru1	73.4(4)

Table S13 Bond Angles for 3.

Atom	Atom	Atom	Angle/˚	Atom	Atom	Atom	Angle/˚
C20	C12	C13	123.0(5)	C39	C38	C43	127.2(9)
C13	C12	N2	117.1(5)	C43	C38	Ru1	129.4(6)
C25	C24	N2	107.3(5)	C7	C2	C1	123.3(18)
C74	C73	N6	118.5(5)	C3	C2	C7	119.9(15)
C81	C73	N6	118.1(5)	C3	C2	C1	116.7(18)
C81	C73	C74	123.3(5)	C40	C39	Ru1	71.2(3)
C73	C74	C75	123.7(5)	C40	C39	C44	125.1(8)
C78	C74	C73	117.3(6)	C38	C39	Ru1	70.2(3)
C78	C74	C75	119.0(6)	C38	C39	C40	108.0(6)
C55	C49	C50	118.1(5)	C38	C39	C44	125.5(8)
C55	C49	C48	121.1(5)	C44	C39	Ru1	134.5(5)
C50	C49	C48	120.8(5)	C27	C28	C30	113.0(9)
C52	C51	C50	120.9(5)	C29	C28	C27	111.7(8)
C89	C88	Ru2	71.3(3)	C29	C28	C30	105.4(8)
C89	C88	C93	125.3(6)	C7	C6	C8	115.1(15)
C87	C88	Ru2	74.9(3)	C5	C6	C7	120.3(14)
C87	C88	C89	106.9(5)	C5	C6	C8	124.1(15)
C87	C88	C93	127.3(6)	C32	C31	C27	120.3(10)
C93	C88	Ru2	125.8(4)	C31	C32	C33	122.0(8)
N4	C56	P2	173.0(5)	C4	C5	C6	118.5(14)
C51	C52	C53	121.2(5)	C34	C33	C32	118.9(10)
C81	C82	C83	112.7(5)	C5	C4	C3	121.8(15)
C81	C82	C84	111.5(5)	C2	C3	C4	119.2(16)
C84	C82	C83	109.7(5)	C102	C101	C100	121(2)
C88	C89	Ru2	70.9(3)	C103	C102	C101	120(2)

Table S13 Bond Angles for 3.

Atom	Atom	Atom	Angle/˚	Atom	Atom	Atom	Angle/˚
C88	C89	C94	124.6(5)	C104	C103	C102	120(2)
C85	C89	Ru2	72.6(3)	C97	C98	C99	115.1(18)
C85	C89	C88	108.2(5)	C96	C97	C98	117.6(19)
C85	C89	C94	127.1(5)	C97	C96	C95	116.3(18)
C94	C89	Ru2	125.1(5)	C7A	C6A	C8A	124(3)
C9	N1	C7	138.5(10)	C5A	C6A	C8A	121(3)
C9	N1	C7A	123.5(15)	C5A	C6A	C7A	112(2)
C63	C61	C60	109.7(5)	C6A	C7A	N1	113(2)
C63	C61	C62	110.5(6)	C6A	C7A	C2A	123(2)
C62	C61	C60	112.0(6)	C2A	C7A	N1	115(2)
C71	C72	N6	107.1(5)	C7A	C2A	C1A	118(3)
C59	C67	C68	123.6(6)	C3A	C2A	C7A	121(2)
C66	C67	C59	117.4(6)	C3A	C2A	C1A	121(3)
C66	C67	C68	119.0(6)	C2A	C3A	C4A	116(3)
C72	C71	N5	106.5(5)	C3A	C4A	C5A	121(2)
N2	C11	Ru1	126.2(4)	C6A	C5A	C4A	122(3)
N3	C11	Ru1	132.3(4)				

Table S14 Torsion Angles for 3.

A	B	C	D	Angle/˚	A	B	C	D	Angle/˚
Ru2	C88	C89	C85	-63.4(4)	C90	C85	C86	Ru2	-128.9(6)
Ru2	C88	C89	C94	120.0(6)	C90	C85	C86	C87	169.6(6)
Ru2	C88	C87	C86	61.8(4)	C90	C85	C86	C91	2.4(10)
Ru2	C88	C87	C92	-122.9(6)	C68	C67	C66	C65	-177.4(6)
Ru2	C89	C85	C90	126.3(6)	C75	C74	C78	C79	176.0(6)

Table S14 Torsion Angles for 3.

A	B	C	D	Angle/˚	A	B	C	D	Angle/˚
Ru2	C89	C85	C86	-65.4(4)	C25	N3	C11	Ru1	169.5(5)
Ru2	C85	C86	C87	-61.5(4)	C25	N3	C11	N2	-1.6(6)
Ru2	C85	C86	C91	131.3(6)	C25	N3	C26	C34	-84.8(8)
Ru2	C87	C86	C85	60.1(4)	C25	N3	C26	C27	83.7(7)
Ru2	C87	C86	C91	-132.5(6)	C85	Ru2	C57	O2	-63(2)
Ru1	C41	C42	C38	-63.4(4)	C20	C12	C13	C17	3.7(9)
Ru1	C41	C42	C47	121.1(7)	C20	C12	C13	C14	-172.3(6)
Ru1	C41	C40	C39	63.2(4)	C81	C73	C74	C75	-176.3(5)
Ru1	C41	C40	C45	-124.9(7)	C81	C73	C74	C78	3.0(9)
Ru1	C42	C38	C39	-64.9(4)	C81	C80	C79	C78	0.9(12)
Ru1	C42	C38	C43	125.4(8)	C13	C12	C20	C21	171.8(6)
Ru1	C40	C39	C38	60.9(4)	C13	C12	C20	C19	-4.3(9)
Ru1	C40	C39	C44	-131.8(7)	C87	Ru2	C57	O2	8(2)
Ru1	C38	C39	C40	-61.6(4)	C87	C88	C89	Ru2	67.0(4)
Ru1	C38	C39	C44	131.2(7)	C87	C88	C89	C85	3.6(6)
P2	Ru2	C57	O2	97(2)	C87	C88	C89	C94	-173.0(6)
P1	Ru1	C10	O1	103.9(19)	C21	C20	C19	C18	-175.6(6)
N4	C55	C53	C52	-178.8(5)	C66	C67	C68	C70	86.0(7)
N4	C55	C53	C54	-2.0(8)	C66	C67	C68	C69	-36.9(8)
N4	C55	C49	C50	179.5(5)	C41	Ru1	C10	O1	5.2(19)
N4	C55	C49	C48	-0.2(8)	C41	C42	C38	Ru1	62.7(4)
N2	C12	C20	C21	-1.7(9)	C41	C42	C38	C39	-2.2(7)
N2	C12	C20	C19	-177.9(5)	C41	C42	C38	C43	-171.9(7)
N2	C12	C13	C17	177.4(5)	C41	C40	C39	Ru1	-61.7(4)
N2	C12	C13	C14	1.4(9)	C41	C40	C39	C38	-0.8(7)

Table S14 Torsion Angles for 3.

A	B	C	D	Angle/˚	A	B	C	D	Angle/˚
N2	C24	C25	N3	-1.6(7)	C41	C40	C39	C44	166.6(7)
N5	C59	C60	C61	0.3(8)	C64	C60	C61	C63	-75.0(8)
N5	C59	C60	C64	173.9(5)	C64	C60	C61	C62	48.2(8)
N5	C59	C67	C68	4.2(8)	C78	C74	C75	C77	-47.9(8)
N5	C59	C67	C66	-173.3(5)	C78	C74	C75	C76	75.6(8)
N6	C73	C74	C75	7.9(8)	C86	Ru2	C57	O2	-41(2)
N6	C73	C74	C78	-172.8(5)	C83	C82	C81	C73	-157.6(6)
N6	C73	C81	C82	-2.0(8)	C83	C82	C81	C80	25.6(8)
N6	C73	C81	C80	175.1(5)	C84	C82	C81	C73	78.7(7)
N6	C72	C71	N5	0.5(6)	C84	C82	C81	C80	-98.2(7)
N3	C26	C34	C35	-4.9(10)	C94	C89	C85	Ru2	-121.1(6)
N3	C26	C34	C33	171.8(7)	C94	C89	C85	C90	5.2(10)
N3	C26	C27	C28	7.7(10)	C94	C89	C85	C86	173.5(6)
N3	C26	C27	C31	-173.2(7)	C93	C88	C89	Ru2	-121.1(6)
C55	C53	C52	C51	0.2(8)	C93	C88	C89	C85	175.5(5)
C58	Ru2	C57	O2	-178(2)	C93	C88	C89	C94	-1.1(9)
C58	N5	C59	C60	97.8(7)	C93	C88	C87	Ru2	123.7(6)
C58	N5	C59	C67	-91.1(7)	C93	C88	C87	C86	-174.5(5)
C58	N5	C71	C72	1.4(6)	C93	C88	C87	C92	0.8(10)
C58	N6	C73	C74	-111.3(6)	C26	N3	C11	Ru1	-13.1(10)
C58	N6	C73	C81	72.8(7)	C26	N3	C11	N2	175.9(6)
C58	N6	C72	C71	-2.2(6)	C26	N3	C25	C24	-175.8(6)
C59	N5	C58	Ru2	-19.2(8)	C26	C34	C33	C32	-1.4(13)
C59	N5	C58	N6	170.5(5)	C26	C27	C28	C30	152.7(8)
C59	N5	C71	C72	-172.7(5)	C26	C27	C28	C29	-88.7(10)

Table S14 Torsion Angles for 3.

A	B	C	D	Angle/°	A	B	C	D	Angle/°
C59	C60	C61	C63	98.5(7)	C26	C27	C31	C32	3.3(13)
C59	C60	C61	C62	-138.4(6)	C92	C87	C86	Ru2	125.5(6)
C59	C60	C64	C65	-1.6(9)	C92	C87	C86	C85	-174.3(6)
C59	C67	C68	C70	-91.4(7)	C92	C87	C86	C91	-7.0(10)
C59	C67	C68	C69	145.6(6)	C19	C20	C21	C23	78.1(7)
C59	C67	C66	C65	0.2(9)	C19	C20	C21	C22	-44.5(8)
C53	C55	C49	C50	3.3(8)	C19	C18	C17	C13	-3.9(11)
C53	C55	C49	C48	-176.4(5)	C42	Ru1	C10	O1	-31.0(19)
C60	C59	C67	C68	175.0(6)	C42	C41	C40	Ru1	-63.7(4)
C60	C59	C67	C66	-2.5(9)	C42	C41	C40	C39	-0.6(7)
C60	C64	C65	C66	-0.6(10)	C42	C41	C40	C45	171.4(6)
C50	C51	C52	C53	2.0(9)	C42	C38	C39	Ru1	63.4(4)
C12	N2	C24	C25	175.0(5)	C42	C38	C39	C40	1.8(7)
C12	N2	C11	Ru1	15.1(8)	C42	C38	C39	C44	-165.5(7)
C12	N2	C11	N3	-173.1(5)	C17	C13	C14	C15	43.9(9)
C12	C20	C21	C23	-98.0(7)	C17	C13	C14	C16	-76.8(8)
C12	C20	C21	C22	139.4(6)	C17	C18	C19	C20	3.2(11)
C12	C20	C19	C18	0.8(9)	C35	C34	C33	C32	175.4(8)
C12	C13	C17	C18	0.5(9)	C7	C2	C3	C4	1(3)
C12	C13	C14	C15	-140.3(7)	C7	C6	C5	C4	-3(3)
C12	C13	C14	C16	99.0(8)	C34	C26	C27	C28	175.9(7)
C24	N2	C12	C20	77.4(7)	C34	C26	C27	C31	-5.0(11)
C24	N2	C12	C13	-96.5(6)	C14	C13	C17	C18	176.5(6)
C24	N2	C11	Ru1	-171.2(4)	C9	N1	C7	C2	133.6(15)
C24	N2	C11	N3	0.6(6)	C9	N1	C7	C6	-43(3)

Table S14 Torsion Angles for 3.

A	B	C	D	Angle/˚	A	B	C	D	Angle/˚
C73	N6	C58	Ru2	17.7(7)	C9	N1	C7A	C6A	-68(4)
C73	N6	C58	N5	-171.3(5)	C9	N1	C7A	C2A	143(3)
C73	N6	C72	C71	172.6(5)	C40	Ru1	C10	O1	17(2)
C73	C74	C75	C77	131.4(6)	C40	C41	C42	Ru1	65.1(4)
C73	C74	C75	C76	-105.1(7)	C40	C41	C42	C38	1.7(7)
C73	C74	C78	C79	-3.3(10)	C40	C41	C42	C47	-173.8(7)
C73	C81	C80	C79	-1.3(10)	C27	C26	C34	C35	-172.6(7)
C74	C73	C81	C82	-177.8(5)	C27	C26	C34	C33	4.1(11)
C74	C73	C81	C80	-0.7(8)	C27	C31	C32	C33	-0.9(16)
C74	C78	C79	C80	1.5(12)	C36	C35	C34	C26	106.5(8)
C49	C55	C53	C52	-2.8(8)	C36	C35	C34	C33	-70.2(9)
C49	C55	C53	C54	174.0(5)	C38	Ru1	C10	O1	-55(2)
C49	C50	C51	C52	-1.5(9)	C2	C7	C6	C5	7(4)
C51	C50	C49	C55	-1.1(8)	C2	C7	C6	C8	180(2)
C51	C50	C49	C48	178.6(6)	C39	Ru1	C10	O1	-33(2)
C88	Ru2	C57	O2	-1(2)	C28	C27	C31	C32	-177.5(9)
C88	C89	C85	Ru2	62.4(4)	C46	C41	C42	Ru1	-122.2(6)
C88	C89	C85	C90	-171.3(6)	C46	C41	C42	C38	174.4(6)
C88	C89	C85	C86	-3.0(6)	C46	C41	C42	C47	-1.1(11)
C88	C87	C86	Ru2	-59.1(4)	C46	C41	C40	Ru1	123.6(7)
C88	C87	C86	C85	1.0(6)	C46	C41	C40	C39	-173.2(6)
C88	C87	C86	C91	168.4(6)	C46	C41	C40	C45	-1.2(10)
C56	N4	C55	C53	-50.1(8)	C37	C35	C34	C26	-127.7(8)
C56	N4	C55	C49	133.6(6)	C37	C35	C34	C33	55.6(10)
C82	C81	C80	C79	175.7(6)	C1	C2	C3	C4	-177(2)

Table S14 Torsion Angles for 3.

A	B	C	D	Angle/˚	A	B	C	D	Angle/˚
C89	Ru2	C57	O2	-38(2)	C6	C7	C2	C1	171(3)
C89	C88	C87	Ru2	-64.6(4)	C6	C7	C2	C3	-6(3)
C89	C88	C87	C86	-2.8(6)	C6	C5	C4	C3	-2(3)
C89	C88	C87	C92	172.5(6)	C31	C27	C28	C30	-26.4(11)
C89	C85	C86	Ru2	62.7(4)	C31	C27	C28	C29	92.2(10)
C89	C85	C86	C87	1.3(6)	C31	C32	C33	C34	-0.2(15)
C89	C85	C86	C91	-166.0(6)	C5	C4	C3	C2	3(3)
N1	C7	C2	C1	-6(3)	C45	C40	C39	Ru1	126.8(7)
N1	C7	C2	C3	176.8(18)	C45	C40	C39	C38	-172.3(7)
N1	C7	C6	C5	-175.8(19)	C45	C40	C39	C44	-5.0(11)
N1	C7	C6	C8	-3(4)	C43	C38	C39	Ru1	-127.3(8)
N1	C7A	C2A	C3A	161(3)	C43	C38	C39	C40	171.1(7)
N1	C7A	C2A	C1A	-20(5)	C43	C38	C39	C44	3.8(12)
C61	C60	C64	C65	172.2(6)	C8	C6	C5	C4	-175(2)
C72	N6	C58	Ru2	-168.2(4)	C100	C101	C102	C103	-169(3)
C72	N6	C58	N5	2.9(5)	C101	C102	C103	C104	168(3)
C72	N6	C73	C74	74.9(6)	C99	C98	C97	C96	173(3)
C72	N6	C73	C81	-101.1(6)	C98	C97	C96	C95	170(3)
C67	C59	C60	C61	-170.4(6)	C8A	C6A	C7A	N1	29(6)
C67	C59	C60	C64	3.2(9)	C8A	C6A	C7A	C2A	176(4)
C67	C66	C65	C64	1.4(10)	C8A	C6A	C5A	C4A	-172(4)
C54	C53	C52	C51	-176.7(6)	C6A	C7A	C2A	C3A	14(6)
C71	N5	C58	Ru2	167.7(4)	C6A	C7A	C2A	C1A	-167(4)
C71	N5	C58	N6	-2.6(5)	C7A	C6A	C5A	C4A	25(5)
C71	N5	C59	C60	-89.4(6)	C7A	C2A	C3A	C4A	-7(5)

Table S14 Torsion Angles for 3.

A	B	C	D	Angle/˚	A	B	C	D	Angle/˚
C71	N5	C59	C67	81.7(6)	C2A	C3A	C4A	C5A	10(4)
C11	Ru1	C10	O1	-170.6(19)	C3A	C4A	C5A	C6A	-20(5)
C11	N2	C12	C20	-109.2(7)	C5A	C6A	C7A	N1	-169(3)
C11	N2	C12	C13	76.9(7)	C5A	C6A	C7A	C2A	-22(6)
C11	N2	C24	C25	0.6(7)	C1A	C2A	C3A	C4A	174(4)
C11	N3	C25	C24	2.1(7)	C47	C42	C38	Ru1	-121.7(7)
C11	N3	C26	C34	97.8(8)	C47	C42	C38	C39	173.4(7)
C11	N3	C26	C27	-93.7(8)	C47	C42	C38	C43	3.7(11)

Table S15 Hydrogen Atom Coordinates (Å×10^4) and Isotropic Displacement Parameters (Å^2×10^3) for 3.

Atom	*x*	*y*	*z*	U(eq)
H50	2516.62	10157.04	6652.95	59
H24	4322.95	7616	6922.61	61
H51	4118.39	10598.31	6205.07	65
H52	5419.14	9932.76	5682.59	61
H82	-653.19	7267.32	4342	64
H48A	1999.41	8466.16	6950.16	86
H48B	1328.82	8836.6	6346.04	86
H48C	1324.73	9262.06	6861.39	86
H61	4019.9	6344.45	4383.05	73
H72	1030	5290.04	5198.33	60
H54A	5170.29	8600.1	5059.93	81
H54B	5669.04	8158.23	5697.08	81
H54C	6125.77	8869.4	5345.64	81

Table S15 Hydrogen Atom Coordinates (Å×10[4]) and Isotropic Displacement Parameters (Å[2]×10[3]) for 3.

Atom	*x*	*y*	*z*	U(eq)
H71	2162.49	5251.7	4252.59	63
H90A	-269.96	8130.97	3255.88	94
H90B	-458.1	8964.43	2932.15	94
H90C	-1018.1	8694.4	3582.05	94
H68	336.14	6783.63	3420.33	65
H75	2306.44	6187.06	5830.68	66
H25	5090.84	8680.02	6995.72	68
H21	6476.17	6455.03	7035.65	63
H23A	5147.85	5989.27	6268.61	88
H23B	6148.7	6420.46	6025.69	88
H23C	5060.02	6795.58	6326.97	88
H66	2021.36	6724.87	2081.24	71
H77A	1842	6436.74	7022	92
H77B	2125.56	7039.78	6447.82	92
H77C	3047.53	6358.95	6701.03	92
H64	5032.1	6506.95	2829.7	74
H78	21.94	6398.12	6909.73	79
H83A	-2958.11	7500.34	4728.21	97
H83B	-2482.49	7808.09	4064.36	97
H83C	-2186.53	8103.09	4617.76	97
H65	3952.38	6682.48	1975.03	78
H84A	-828.33	6033.63	4510.5	97
H84B	-1693.03	6515.98	4019.25	97
H84C	-2112.47	6218.9	4697.36	97

Table S15 Hydrogen Atom Coordinates (Å×10[4]) and Isotropic Displacement Parameters (Å[2]×10[3]) for 3.

Atom	*x*	*y*	*z*	U(eq)
H91A	2885.85	7994.83	2869.61	96
H91B	2277.52	8748.07	2487.1	96
H91C	1613.48	8090.08	2694.56	96
H94A	-957.45	9132.53	4436.98	99
H94B	-579.28	9899.13	4219.62	99
H94C	-152.1	9373.29	4843.6	99
H93A	1770.02	9416.19	5004.88	89
H93B	2037.5	10037.92	4441.96	89
H93C	2982.04	9368.63	4689.82	89
H92A	4115.25	8706.26	4009.44	99
H92B	3834.88	9337.66	3419.67	99
H92C	4040.55	8522.95	3370.24	99
H70A	612.69	5676.2	2762.68	95
H70B	-457.71	5865.87	3173.02	95
H70C	716.59	5527.68	3478.2	95
H18	3604.66	4700.01	8052.46	78
H19	5031.97	4932.77	7330.19	76
H80	-2368.19	7263.68	5671.4	78
H76A	2768.05	5133.7	6654.95	109
H76B	1874.06	5031.9	6217.33	109
H76C	1480.84	5203.69	6855.48	109
H17	2775.94	5561.55	8567.26	77
H79	-1804.49	6934.22	6668.45	87
H35	7687.12	7888.59	7342.78	95

Table S15 Hydrogen Atom Coordinates (Å×10^4) and Isotropic Displacement Parameters (Å^2×10^3) for 3.

Atom	*x*	*y*	*z*	U(eq)
H22A	7270.42	5184.26	7365.76	100
H22B	7624.61	5537.08	6687.29	100
H22C	6676.56	5046.59	6812.86	100
H14	3749.23	7214.19	8557.61	85
H69A	204.08	7603.61	2415.23	98
H69B	-827.95	7184.79	2591.12	98
H69C	134.89	6935.2	2140.84	98
H15A	2519.93	6933.02	9406.36	126
H15B	3577	6311.68	9448.83	126
H15C	2413.59	6202.38	9235.54	126
H36A	7221.51	8916.29	6538.73	136
H36B	8376.66	8447.47	6399.04	136
H36C	8369.95	9166.41	6615.33	136
H63A	4388.46	5166.64	4278.44	122
H63B	5528.33	5330.76	4474.42	122
H63C	5358.16	5337.34	3785.37	122
H62A	5942.45	6614.23	3611.42	123
H62B	5858.09	6592.18	4316.99	123
H62C	5116.94	7222.14	3853.43	123
H28	4342.66	8368.82	8649.84	109
H46A	8785.55	5216.89	10220.62	120
H46B	8918.48	5246.16	9514.31	120
H46C	7862.84	4971.47	9890.24	120
H37A	9650.31	8451.53	7512.25	154

Table S15 Hydrogen Atom Coordinates (Å×10^4) and Isotropic Displacement Parameters (Å^2×10^3) for 3.

Atom	*x*	*y*	*z*	U(eq)
H37B	9671.57	7758.11	7256.02	154
H37C	9286.47	7745.96	7941.02	154
H1A	6984.39	3922.96	9353.92	105
H1B	7056.39	3147.36	9227.11	105
H1C	6943.26	3839.99	8677.18	105
H31	5458.52	9951.12	8634.6	125
H30A	4503.26	9095.56	9408.43	171
H30B	3264.45	8990.2	9313.62	171
H30C	3677.28	9727.38	8994.37	171
H32	7185.78	10218.66	8245.56	137
H5	11726.93	3311.93	9040.08	96
H33	8358.03	9490.36	7683.37	122
H16A	1620.1	6954.91	8257.6	158
H16B	2354.32	7452.64	7791.48	158
H16C	1840.15	7684.14	8390.32	158
H45A	9868.03	6570.26	9629.03	152
H45B	9526.49	7190.42	9039.63	152
H45C	9674.54	6370.44	9010.31	152
H44A	8112.15	8183.07	9034.44	185
H44B	7726.64	8191.29	9716.03	185
H44C	6828.72	8419.81	9196.63	185
H29A	3507.4	9749.39	7895.23	177
H29B	2692.49	9219.73	8246.6	177
H29C	3539.79	8994.58	7734.94	177

Table S15 Hydrogen Atom Coordinates (Å×10^4) and Isotropic Displacement Parameters (Å^2×10^3) for 3.

Atom	*x*	*y*	*z*	U(eq)
H43A	5125.52	7954.74	9651.01	201
H43B	5308.12	7516.87	10335.97	201
H43C	4624.78	7232.42	9898.56	201
H4	10777.1	2354.09	9505.56	111
H3	8807.56	2509.64	9538.47	95
H8A	10524.64	4829.26	7949.14	135
H8B	11637.07	4542.23	8325.95	135
H8C	10651.47	5079.5	8553.31	135
H10A	3795.28	8851.17	10791.79	126
H10B	3455.3	8448.81	11458.89	126
H10C	3834.97	8004.08	10957.44	126
H10D	5535.33	7850.61	11201.61	101
H10E	5499.24	8692.18	11031.57	101
H10F	4925.46	8674.56	12062.44	101
H10G	5211.28	7826.64	12196.04	101
H10H	7050.47	7845.28	12055.49	101
H10I	6851.57	8685.52	11782.98	101
H10J	7565.29	8526.14	12688.52	126
H10K	6753.73	7979.5	13010.32	126
H10L	6271.78	8812.42	12783.93	126
H99A	10018.5	8635.22	9104.53	127
H99B	10292.79	8862.89	9697.42	127
H99C	11264.1	8711.8	9216.89	127
H98A	10901.04	9919.42	8962.63	101

Table S15 Hydrogen Atom Coordinates (Å×10[4]) and Isotropic Displacement Parameters (Å[2]×10[3]) for 3.

Atom	*x*	*y*	*z*	U(eq)
H98B	10168.15	9724.89	8493.7	101
H97A	8544.25	9954.98	9069.41	101
H97B	9242.56	10068.04	9591.66	101
H96A	9087.11	11035.82	8432.8	101
H96B	9589.56	11161.13	9016.5	101
H95A	7469.28	11613.02	8652.33	127
H95B	7967.31	11726.52	9244.23	127
H95C	7408.01	11036.91	9280.18	127
H8AA	10318.45	5183	7802.34	135
H8AB	11563.38	4841.32	8003.93	135
H8AC	10782.68	5320.82	8393.5	135
H3A	9371.87	2572.8	9588.55	99
H4A	11296.98	2593.25	9291.95	90
H5A	11864.8	3730.51	8907.3	99
H1AA	7318.13	3885.95	9448.59	105
H1AB	7470.41	3130.16	9274.82	105
H1AC	7276.37	3854.31	8758	105
H47A	5059.32	5987.1	10174.83	145
H47B	5966.85	5681.78	10687.03	145
H47C	5991.41	5313.36	10133	145

Table S16 Atomic Occupancy for 3.

Atom	*Occupancy*	Atom	*Occupancy*	Atom	*Occupancy*
C7	0.62(3)	C2	0.62(3)	C1	0.62(3)

Table S16 Atomic Occupancy for 3.

Atom	*Occupancy*	Atom	*Occupancy*	Atom	*Occupancy*
H1A	0.62(3)	H1B	0.62(3)	H1C	0.62(3)
C6	0.62(3)	C5	0.62(3)	H5	0.62(3)
C4	0.62(3)	H4	0.62(3)	C3	0.62(3)
H3	0.62(3)	C8	0.62(3)	H8A	0.62(3)
H8B	0.62(3)	H8C	0.62(3)	C100	0.25
H10A	0.25	H10B	0.25	H10C	0.25
C101	0.25	H10D	0.25	H10E	0.25
C102	0.25	H10F	0.25	H10G	0.25
C103	0.25	H10H	0.25	H10I	0.25
C104	0.25	H10J	0.25	H10K	0.25
H10L	0.25	C99	0.25	H99A	0.25
H99B	0.25	H99C	0.25	C98	0.25
H98A	0.25	H98B	0.25	C97	0.25
H97A	0.25	H97B	0.25	C96	0.25
H96A	0.25	H96B	0.25	C95	0.25
H95A	0.25	H95B	0.25	H95C	0.25
C8A	0.38(3)	H8AA	0.38(3)	H8AB	0.38(3)
H8AC	0.38(3)	C6A	0.38(3)	C7A	0.38(3)
C2A	0.38(3)	C3A	0.38(3)	H3A	0.38(3)
C4A	0.38(3)	H4A	0.38(3)	C5A	0.38(3)
H5A	0.38(3)	C1A	0.38(3)	H1AA	0.38(3)
H1AB	0.38(3)	H1AC	0.38(3)		

Experimental

Single crystals of $C_{48.25}H_{63}N_3OPRu$ **3** were grown from pentane at – 30 ^oC. A suitable crystal was selected and mounted on on a **ROD, Synergy Custom DW system, Pilatus 200K** diffractometer. The crystal was kept at 293(2) K during data collection. Using Olex2 [1], the structure was solved with the ShelX2 structure solution program.

1. Dolomanov, O.V., Bourhis, L.J., Gildea, R.J, Howard, J.A.K. & Puschmann, H. (2009), J. Appl. Cryst. 42, 339-341.

Crystal structure determination of 3

Crystal Data for $C_{48.25}H_{63}N_3OPRu$ (M =833.05 g/mol): triclinic, space group P-1 (no. 2), a = 11.9764(3) Å, b = 19.1730(5) Å, c = 22.4039(6) Å, α = 75.824(2)°, β = 86.064(2)°, γ = 79.796(2)°, V = 4907.2(2) Å^3, Z = 4, T = 293(2) K, μ(Cu Kα) = 3.145 mm^{-1}, $Dcalc$ = 1.128 g/cm^3, 98678 reflections measured (5.522° ≤ 2Θ ≤ 149.008°), 19975 unique (R_{int} = 0.1226, R_{sigma} = 0.0774) which were used in all calculations. The final R_1 was 0.0800 (I > 2σ(I)) and wR_2 was 0.2516 (all data).

Refinement model description

Number of restraints - 360, number of constraints - unknown.

Details:

1. Restrained distances
C100-C101 = C101-C102 = C102-C103 = C103-C104
1.52 with sigma of 0.02
C99-C98 = C98-C97 = C97-C96 = C96-C95
1.52 with sigma of 0.02
C100-C102 = C101-C103 = C102-C104
2.54 with sigma of 0.04
C99-C97 = C98-C96 = C97-C95
2.54 with sigma of 0.04
C100-C101 ≈ C101-C102 ≈ C102-C103 ≈ C103-C104
with sigma of 0.02
C100-C102 ≈ C102-C104 ≈ C101-C103
with sigma of 0.04
C99-C98 ≈ C98-C97 ≈ C97-C96 ≈ C96-C95
with sigma of 0.02
C99-C97 ≈ C97-C95 ≈ C98-C96
with sigma of 0.04
C6A-C8 ≈ C6-C8A
with sigma of 0.02
C5-C8 ≈ C5A-C8A
with sigma of 0.04
C7A-C2A ≈ C7-C2 ≈ C2A-C3A ≈ C2-C3 ≈ C3A-C4A ≈ C3-C4 ≈
C4A-C5A ≈ C4-C5 ≈ C5A-
C6A ≈ C5-C6 ≈ C6A-C7A ≈ C6-C7
with sigma of 0.02
C7A-N1 ≈ C7-N1
with sigma of 0.02
C39-C40 ≈ C40-C41 ≈ C41-C42 ≈ C42-C38 ≈ C38-C39
with sigma of 0.02
Ru1-C41 ≈ Ru1-C42 ≈ Ru1-C38 ≈ Ru1-C40 ≈ Ru1-C39

with sigma of 0.02
C39-C41 ≈ C40-C42 ≈ C41-C38 ≈ C42-C39 ≈ C38-C40
with sigma of 0.04
2. Rigid bond restraints
C8, C8A, C6A, C6
with sigma for 1-2 distances of 0.01 and sigma for 1-3 distances of 0.01
3. Uiso/Uaniso restraints and constraints
C100 ≈ C101 ≈ C102 ≈ C103 ≈ C104: within 2A with sigma of 0.04
and sigma for terminal atoms of 0.08 within 2A
C99 ≈ C98 ≈ C97 ≈ C96 ≈ C95: within 2A with sigma of 0.04 and
sigma for terminal atoms of 0.08 within 2A
C102 ≈ C100 ≈ C101 ≈ C103 ≈ C104: within 2A with sigma of 0.04
and sigma for terminal atoms of 0.08 within 2A
C42: within 2A with sigma of 0.04 and sigma for terminal atoms of 0.08 within 2A
Uanis(C99) = Uanis(C98) = Uanis(C97) = Uanis(C96) = Uanis(C95)
Uanis(C100) = Uanis(C101) = Uanis(C102) = Uanis(C103) = Uanis(C104)
Uanis(C8A) = Uanis(C8)
Uanis(C1A) = Uanis(C1)
Uanis(C2A) = Uanis(C2)
4. Rigid body (RIGU) restrains
C100, C101, C102, C103, C104
with sigma for 1-2 distances of 0.004 and sigma for 1-3 distances of 0.004
C99, C98, C97, C96, C95
with sigma for 1-2 distances of 0.004 and sigma for 1-3 distances of 0.004
C7, C7A, C6, C6A, C2, C2A, C3, C3A, C4, C4A, C5, C5A
with sigma for 1-2 distances of 0.004 and sigma for 1-3 distances of 0.004
C39, C44, C40, C45, C41, C46, C42, C38, C43
with sigma for 1-2 distances of 0.004 and sigma for 1-3 distances of 0.004
5. Others
Sof(C8A)=Sof(H8AA)=Sof(H8AB)=Sof(H8AC)=Sof(C6A)=Sof(C7A)=Sof(C2A)=Sof(C3A)=
Sof(H3A)=Sof(C4A)=Sof(H4A)=Sof(C5A)=Sof(H5A)=Sof(C1A)=Sof(H1AA)=Sof(H1AB)=
Sof(H1AC)=1-FVAR(1)
Sof(C7)=Sof(C2)=Sof(C1)=Sof(H1A)=Sof(H1B)=Sof(H1C)=Sof(C6)=Sof(C5)=Sof(H5)=
Sof(C4)=Sof(H4)=Sof(C3)=Sof(H3)=Sof(C8)=Sof(H8A)=Sof(H8B)=Sof(H8C)=FVAR(1)
Fixed Sof: C100(0.25) H10A(0.25) H10B(0.25) H10C(0.25) C101(0.25) H10D(0.25)
H10E(0.25) C102(0.25) H10F(0.25) H10G(0.25) C103(0.25) H10H(0.25) H10I(0.25)
C104(0.25) H10J(0.25) H10K(0.25) H10L(0.25) C99(0.25) H99A(0.25) H99B(0.25)
H99C(0.25) C98(0.25) H98A(0.25) H98B(0.25) C97(0.25) H97A(0.25) H97B(0.25)
C96(0.25) H96A(0.25) H96B(0.25) C95(0.25) H95A(0.25) H95B(0.25) H95C(0.25)
6.a Ternary CH refined with riding coordinates:
C82(H82), C61(H61), C68(H68), C75(H75), C21(H21), C35(H35), C14(H14), C28(H28)
6.b Secondary CH2 refined with riding coordinates:
C101(H10D,H10E), C102(H10F,H10G), C103(H10H,H10I), C98(H98A,H98B), C97(H97A,
H97B), C96(H96A,H96B)
6.c Aromatic/amide H refined with riding coordinates:

C50(H50), C24(H24), C51(H51), C52(H52), C72(H72), C71(H71), C25(H25), C66(H66), C64(H64), C78(H78), C65(H65), C18(H18), C19(H19), C80(H80), C17(H17), C79(H79), C31(H31), C32(H32), C5(H5), C33(H33), C4(H4), C3(H3), C3A(H3A), C4A(H4A), C5A(H5A)

6.d Idealised Me refined as rotating group:

C48(H48A,H48B,H48C), C54(H54A,H54B,H54C), C90(H90A,H90B,H90C), C23(H23A,H23B, H23C), C77(H77A,H77B,H77C), C83(H83A,H83B,H83C), C84(H84A,H84B,H84C), C91(H91A, H91B,H91C), C94(H94A,H94B,H94C), C93(H93A,H93B,H93C), C92(H92A,H92B,H92C), C70(H70A,H70B,H70C), C76(H76A,H76B,H76C), C22(H22A,H22B,H22C), C69(H69A,H69B, H69C), C15(H15A,H15B,H15C), C36(H36A,H36B,H36C), C63(H63A,H63B,H63C), C62(H62A, H62B,H62C), C46(H46A,H46B,H46C), C37(H37A,H37B,H37C), C1(H1A,H1B,H1C), C30(H30A,H30B,H30C), C16(H16A,H16B,H16C), C45(H45A,H45B,H45C), C44(H44A,H44B, H44C), C29(H29A,H29B,H29C), C43(H43A,H43B,H43C), C8(H8A,H8B,H8C), C100(H10A, H10B,H10C), C104(H10J,H10K,H10L), C99(H99A,H99B,H99C), C95(H95A,H95B,H95C), C8A(H8AA,H8AB,H8AC), C1A(H1AA,H1AB,H1AC), C47(H47A,H47B,H47C)

Ru-imidazol-2-yl (4)

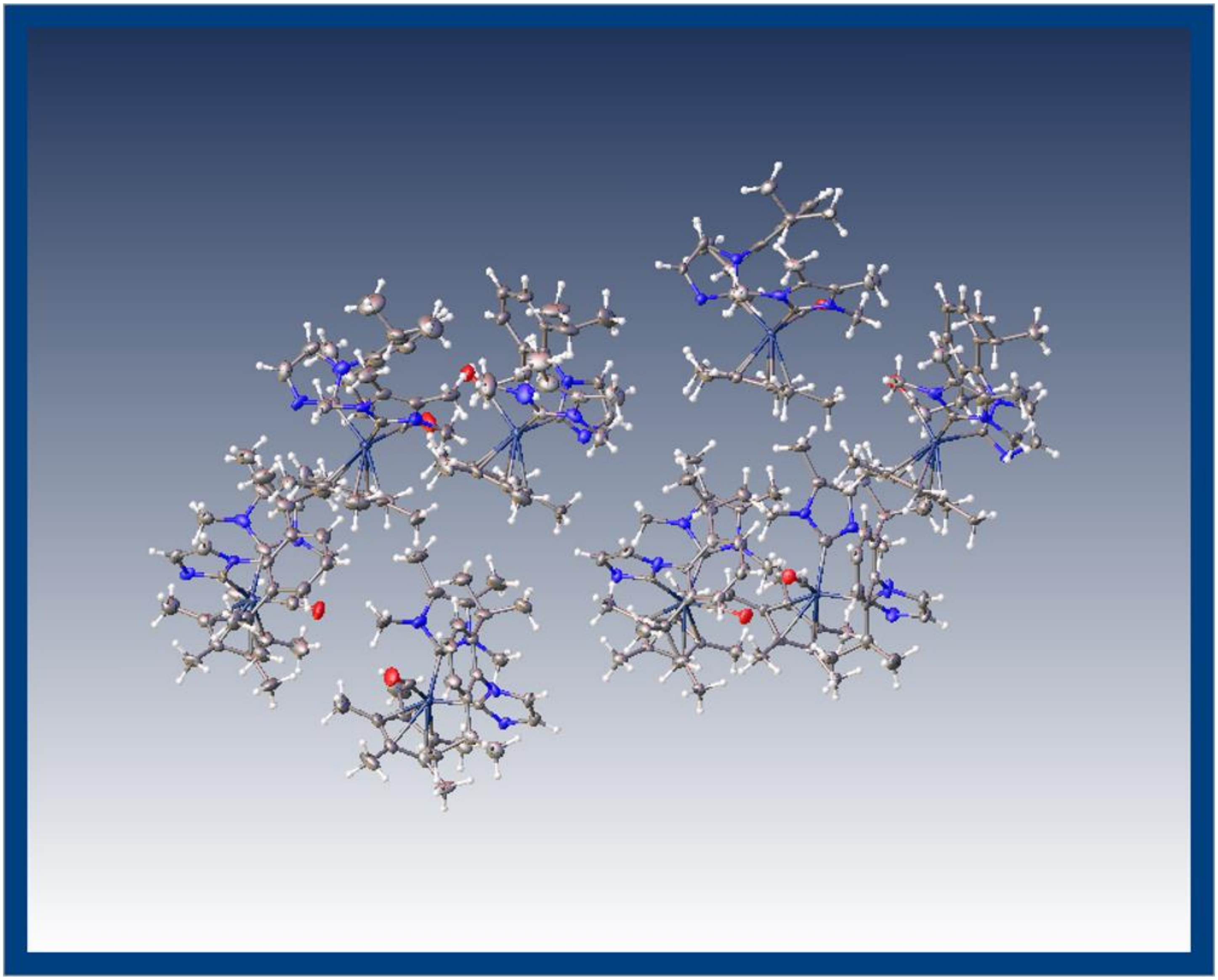

Table S17 Crystal data and structure refinement for 4.

Identification code	TSD66_Tilley_finished
Empirical formula	$C_{33}H_{46}N_4ORu$
Formula weight	615.81
Temperature/K	100.00
Crystal system	triclinic
Space group	P1
a/Å	11.37380(10)

b/Å	19.4936(2)
c/Å	28.0779(3)
α/°	88.6070(10)
β/°	89.9770(10)
γ/°	89.7810(10)
Volume/$Å^3$	6223.44(11)
Z	8
$\rho_{calc}g/cm^3$	1.314
μ/mm^{-1}	0.535
F(000)	2592.0
Crystal size/mm^3	0.2 × 0.14 × 0.1
Radiation	Mo Kα (λ = 0.71073)
2Θ range for data collection/°	5.638 to 52.744
Index ranges	-14 ≤ h ≤ 14, -24 ≤ k ≤ 24, -35 ≤ l ≤ 35
Reflections collected	253374
Independent reflections	50364 [R_{int} = 0.0432, R_{sigma} = 0.0349]
Data/restraints/parameters	50364/1707/2908
Goodness-of-fit on F^2	1.062
Final R indexes [I>=2σ (I)]	R_1 = 0.0446, wR_2 = 0.1000
Final R indexes [all data]	R_1 = 0.0485, wR_2 = 0.1018
Largest diff. peak/hole / e $Å^{-3}$	1.45/-0.98
Flack parameter	-0.145(5)

Table S18 Fractional Atomic Coordinates (×10[4]) and Equivalent Isotropic Displacement Parameters (Å[2]×10[3]) for 4. U_{eq} is defined as 1/3 of the trace of the orthogonalised U_{IJ} tensor.

Atom	*x*	*y*	*z*	U(eq)
Ru1A	4943.2(6)	5098.2(4)	3684.8(3)	22.81(16)
O1A	6268(8)	5684(4)	2856(3)	53(2)
N1A	5914(6)	5266(4)	4675(3)	25.8(17)
N2A	6681(7)	6065(4)	4229(3)	30.4(18)
N3A	3234(7)	6168(4)	4132(3)	28.7(17)
N4A	3268(10)	6279(5)	3376(4)	51(3)
C1A	5796(9)	5518(5)	3203(3)	31(2)
C2A	5912(7)	5502(4)	4234(3)	21.3(18)
C3A	6635(8)	5640(5)	4954(3)	29(2)
C4A	7132(9)	6148(5)	4677(3)	31(2)
C5A	7210(9)	6412(5)	3819(3)	31(2)
C6A	6731(10)	7024(5)	3629(4)	36(2)
C7A	5598(11)	7338(5)	3830(5)	49(3)
C8A	5903(14)	7745(8)	4273(6)	78(4)
C9A	4935(15)	7783(7)	3465(7)	84(5)
C10A	7303(11)	7349(5)	3255(4)	43(3)
C11A	8349(11)	7077(5)	3074(4)	46(3)
C12A	8809(11)	6475(5)	3263(4)	46(3)
C13A	8252(10)	6120(5)	3643(3)	30(2)
C14A	8791(10)	5472(6)	3849(4)	39(2)
C15A	9919(10)	5635(6)	4121(4)	46(3)
C16A	9037(11)	4945(6)	3468(5)	48(3)
C17A	3735(8)	5895(5)	3742(4)	30(2)
C18A	3366(10)	5901(6)	4615(4)	40(3)

Table S18 Fractional Atomic Coordinates (×10^4) and Equivalent Isotropic Displacement Parameters (Å^2×10^3) for 4. U_{eq} is defined as 1/3 of the trace of the orthogonalised U_{IJ} tensor.

Atom	*x*	*y*	*z*	U(eq)
C19A	2493(8)	6722(5)	4015(4)	34(2)
C20A	1868(10)	7108(7)	4388(5)	56(3)
C21A	2505(10)	6789(6)	3544(5)	48(3)
C22A	1872(13)	7297(8)	3223(6)	72(4)
C23A	3476(17)	6162(8)	2865(5)	78(5)
C24A	5422(8)	3998(5)	3852(3)	25.0(18)
C25A	6500(9)	3784(5)	4104(4)	30(2)
C26A	5242(9)	4085(5)	3355(3)	29.9(19)
C27A	6094(12)	3918(6)	2978(4)	47(3)
C28A	4044(10)	4267(5)	3286(4)	35(2)
C29A	3451(15)	4307(7)	2795(5)	64(4)
C30A	3503(10)	4276(5)	3727(4)	35(2)
C31A	2213(11)	4432(6)	3814(5)	58(3)
C32A	4327(8)	4128(5)	4083(3)	28.3(19)
C33A	4068(13)	4015(6)	4605(4)	48(3)
Ru1B	5243.0(5)	8635.3(3)	6400.8(2)	14.20(15)
O1B	7034(5)	9151(3)	7077(2)	25.1(13)
N1B	5673(7)	8767(4)	5347(3)	23.4(11)
N2B	6784(7)	9544(4)	5687(3)	23.4(11)
N3B	3485(6)	9797(4)	6093(3)	23.1(16)
N4B	3871(6)	9841(4)	6831(3)	19.5(15)
C1B	6360(7)	9006(4)	6793(3)	15.1(16)
C2B	5974(8)	9023(5)	5770(3)	22.7(19)
C3B	6292(10)	9125(5)	5005(3)	31(2)

Table S18 Fractional Atomic Coordinates (×10⁴) and Equivalent Isotropic Displacement Parameters (Å²×10³) for 4. U_{eq} is defined as 1/3 of the trace of the orthogonalised U_{IJ} tensor.

Atom	*x*	*y*	*z*	U(eq)
C4B	6987(9)	9593(5)	5201(3)	31(2)
C5B	7560(8)	9852(4)	6016(3)	22.8(17)
C6B	7273(9)	10503(5)	6203(3)	26.5(18)
C7B	6097(8)	10843(5)	6083(3)	28.3(19)
C8B	5623(10)	11283(5)	6493(4)	35(2)
C9B	6199(11)	11286(5)	5625(4)	39(2)
C10B	8097(9)	10816(5)	6477(3)	29.5(19)
C11B	9193(9)	10518(5)	6566(4)	35(2)
C12B	9453(9)	9888(5)	6384(3)	31(2)
C13B	8652(8)	9546(5)	6101(3)	27.1(18)
C14B	8985(9)	8860(5)	5897(4)	33(2)
C15B	10113(10)	8911(6)	5597(4)	44(3)
C16B	9130(9)	8311(6)	6288(4)	36(2)
C17B	4146(7)	9485(4)	6441(3)	18.3(18)
C18B	3425(10)	9612(6)	5590(4)	37(3)
C19B	2825(8)	10336(4)	6271(3)	21.7(19)
C20B	2077(9)	10791(5)	5954(4)	39(3)
C21B	3087(8)	10367(5)	6736(4)	27(2)
C22B	2657(9)	10848(6)	7109(4)	41(3)
C23B	4364(8)	9712(5)	7310(3)	24(2)
C24B	5368(7)	7545(4)	6172(3)	19.5(16)
C25B	6185(9)	7275(5)	5798(3)	28(2)
C26B	4184(8)	7760(5)	6091(3)	24.0(18)
C27B	3531(10)	7731(5)	5623(4)	29(2)

Table S18 Fractional Atomic Coordinates (×10^4) and Equivalent Isotropic Displacement Parameters (Å^2×10^3) for 4. U_{eq} is defined as 1/3 of the trace of the orthogonalised U_{IJ} tensor.

Atom	*x*	*y*	*z*	U(eq)
C28B	3680(8)	7905(4)	6544(3)	20.8(17)
C29B	2437(7)	8137(5)	6632(4)	29(2)
C30B	4547(7)	7803(4)	6901(3)	18.8(16)
C31B	4367(8)	7829(5)	7429(3)	25.7(19)
C32B	5590(7)	7556(4)	6670(3)	18.4(16)
C33B	6654(8)	7312(5)	6933(3)	24.5(18)
Ru1C	821.7(6)	139.6(4)	3653.5(3)	23.90(17)
O1C	-727(7)	689(4)	2871(2)	45.3(19)
N1C	113(6)	323(4)	4678(2)	22.3(15)
N2C	-840(7)	1118(4)	4238(3)	28.0(18)
N3C	2581(7)	1240(4)	4022(3)	30.4(17)
N4C	2406(8)	1295(4)	3257(3)	37.8(19)
C1C	-151(9)	537(5)	3198(3)	31(2)
C2C	-45(7)	554(4)	4227(3)	18.0(16)
C3C	-546(7)	727(5)	4969(3)	26.4(19)
C4C	-1144(7)	1205(5)	4718(3)	22.9(18)
C5C	-1500(9)	1451(5)	3855(3)	27.2(19)
C6C	-2596(9)	1160(5)	3746(3)	31(2)
C7C	-3055(9)	498(5)	3973(4)	33(2)
C8C	-4180(9)	636(6)	4244(4)	42(2)
C9C	-3219(9)	-46(5)	3597(4)	35(2)
C10C	-3289(10)	1518(5)	3410(4)	39(2)
C11C	-2891(11)	2120(5)	3199(4)	44(3)
C12C	-1796(11)	2370(5)	3312(4)	40(2)

Table S18 Fractional Atomic Coordinates (×10^4) and Equivalent Isotropic Displacement Parameters (Å^2×10^3) for 4. U_{eq} is defined as 1/3 of the trace of the orthogonalised U_{IJ} tensor.

Atom	*x*	*y*	*z*	U(eq)
C13C	-1078(9)	2042(5)	3648(3)	29.8(19)
C14C	114(11)	2343(5)	3785(4)	40(2)
C15C	678(12)	2773(6)	3373(5)	51(3)
C16C	-8(11)	2784(6)	4226(4)	45(3)
C17C	2022(8)	937(5)	3659(4)	29(2)
C18C	2498(10)	1048(5)	4514(4)	35(2)
C19C	3296(9)	1785(5)	3854(4)	36(2)
C20C	3947(11)	2234(6)	4183(5)	56(3)
C21C	3187(9)	1802(5)	3375(4)	40(2)
C22C	3743(12)	2271(6)	2997(5)	54(3)
C23C	2047(12)	1163(6)	2761(4)	49(3)
C24C	1709(9)	-782(6)	4015(4)	36(2)
C25C	2246(11)	-820(6)	4504(4)	43(3)
C26C	2303(10)	-629(5)	3581(4)	38(2)
C27C	3572(10)	-420(6)	3511(6)	63(4)
C28C	1494(11)	-720(5)	3199(4)	40(2)
C29C	1749(15)	-687(6)	2671(4)	61(4)
C30C	422(9)	-912(5)	3391(4)	33(2)
C31C	-629(12)	-1133(6)	3103(5)	56(3)
C32C	520(8)	-976(5)	3907(4)	29.9(19)
C33C	-386(9)	-1214(5)	4240(4)	40(3)
Ru1D	1169.9(5)	3642.9(3)	6401.7(2)	14.34(14)
O1D	-698(5)	4143(4)	7057(2)	29.0(15)
N1D	857(7)	3795(4)	5345(3)	24.8(16)

Table S18 Fractional Atomic Coordinates (×10^4) and Equivalent Isotropic Displacement Parameters (Å^2×10^3) for 4. U_{eq} is defined as 1/3 of the trace of the orthogonalised U_{IJ} tensor.

Atom	*x*	*y*	*z*	U(eq)
N2D	-361(7)	4529(4)	5670(3)	28.0(17)
N3D	2436(7)	4850(4)	6871(3)	23.6(16)
N4D	2903(7)	4823(4)	6130(3)	22.3(16)
C1D	1(8)	4000(4)	6775(3)	21.2(17)
C2D	495(7)	4025(4)	5764(3)	15.4(16)
C3D	255(9)	4160(5)	4992(3)	31(2)
C4D	-497(8)	4595(5)	5186(3)	29(2)
C5D	-1194(7)	4838(5)	5989(3)	20.1(17)
C6D	-2254(8)	4497(5)	6071(3)	27.0(18)
C7D	-2504(9)	3791(5)	5884(4)	31(2)
C8D	-3525(11)	3806(6)	5534(4)	47(3)
C9D	-2714(10)	3263(6)	6273(4)	36(2)
C10D	-3097(9)	4828(5)	6345(3)	32(2)
C11D	-2898(9)	5465(6)	6519(4)	36(2)
C12D	-1831(9)	5794(5)	6442(4)	33(2)
C13D	-924(8)	5477(5)	6171(3)	25.3(18)
C14D	194(8)	5841(5)	6073(4)	31(2)
C15D	616(11)	6249(5)	6481(4)	39(2)
C16D	51(12)	6313(6)	5616(4)	46(3)
C17D	2216(8)	4497(5)	6475(3)	22.1(19)
C18D	1932(8)	4702(5)	7341(3)	26(2)
C19D	3233(8)	5383(4)	6788(3)	23.6(18)
C20D	3568(10)	5871(5)	7167(4)	39(2)
C21D	3520(8)	5376(5)	6324(4)	27(2)

Table S18 Fractional Atomic Coordinates (×10[4]) and Equivalent Isotropic Displacement Parameters (Å[2]×10[3]) for 4. U_{eq} is defined as 1/3 of the trace of the orthogonalised U_{IJ} tensor.

Atom	*x*	*y*	*z*	U(eq)
C22D	4246(9)	5844(5)	6020(4)	33(2)
C23D	3019(10)	4637(5)	5636(3)	31(2)
C24D	1860(7)	2808(4)	6897(3)	20.4(16)
C25D	2033(9)	2819(5)	7431(3)	28(2)
C26D	2728(7)	2925(4)	6544(3)	20.0(17)
C27D	3966(7)	3148(5)	6641(4)	27.3(19)
C28D	2246(8)	2771(5)	6095(3)	24.3(18)
C29D	2885(9)	2737(5)	5628(4)	30(2)
C30D	1042(7)	2550(4)	6172(3)	19.3(16)
C31D	224(8)	2295(5)	5798(3)	28(2)
C32D	818(8)	2568(4)	6666(3)	20.4(16)
C33D	-264(8)	2322(5)	6934(3)	25.1(19)
Ru1E	215.8(5)	8603.5(3)	8579.0(2)	15.35(15)
O1E	2006(5)	9159(3)	7896(2)	26.9(14)
N1E	660(8)	8682(4)	9627(3)	31.6(19)
N2E	1767(7)	9470(3)	9286(3)	20.7(15)
N3E	-1538(6)	9745(4)	8896(3)	23.3(15)
N4E	-1154(6)	9838(4)	8147(3)	22.9(15)
C1E	1339(7)	8992(4)	8186(3)	18.4(17)
C2E	961(8)	8949(4)	9206(3)	21.5(19)
C3E	1279(10)	9013(5)	9970(3)	36(2)
C4E	1971(10)	9491(5)	9770(3)	37(2)
C5E	2548(8)	9799(4)	8955(3)	22.2(17)
C6E	2253(8)	10449(4)	8760(3)	22.1(17)

Table S18 Fractional Atomic Coordinates (×10^4) and Equivalent Isotropic Displacement Parameters (Å^2×10^3) for 4. U_{eq} is defined as 1/3 of the trace of the orthogonalised U_{IJ} tensor.

Atom	*x*	*y*	*z*	U(eq)
C7E	1095(8)	10786(4)	8865(3)	26.9(19)
C8E	1176(11)	11197(6)	9326(4)	43(3)
C9E	605(11)	11234(5)	8459(4)	41(2)
C10E	3107(9)	10784(5)	8477(3)	29(2)
C11E	4194(9)	10486(5)	8401(4)	33(2)
C12E	4455(9)	9826(5)	8593(4)	33(2)
C13E	3643(8)	9484(5)	8874(3)	25.9(18)
C14E	3951(10)	8787(5)	9079(4)	35(2)
C15E	5060(11)	8810(6)	9399(4)	49(3)
C16E	4130(10)	8260(5)	8690(4)	37(2)
C17E	-870(8)	9445(5)	8545(3)	23.8(19)
C18E	-1585(10)	9532(6)	9396(4)	36(2)
C19E	-2200(8)	10293(5)	8722(4)	29.6(19)
C20E	-2938(10)	10748(6)	9025(5)	46(3)
C21E	-1945(8)	10363(5)	8248(4)	27.0(18)
C22E	-2358(10)	10874(6)	7878(4)	43(3)
C23E	-686(8)	9737(5)	7669(3)	23.4(18)
C24E	341(7)	7496(4)	8807(3)	20.4(16)
C25E	1167(8)	7207(4)	9187(3)	20.8(17)
C26E	561(7)	7539(4)	8309(3)	18.1(16)
C27E	1643(7)	7310(4)	8049(3)	22.4(18)
C28E	-481(7)	7801(4)	8082(3)	17.6(15)
C29E	-635(8)	7862(5)	7549(3)	25.4(19)
C30E	-1344(8)	7900(5)	8439(3)	21.8(17)

Table S18 Fractional Atomic Coordinates (×10⁴) and Equivalent Isotropic Displacement Parameters (Å²×10³) for 4. U_{eq} is defined as 1/3 of the trace of the orthogonalised U_{IJ} tensor.

Atom	*x*	*y*	*z*	U(eq)
C31E	-2590(7)	8131(5)	8349(4)	26.3(19)
C32E	-838(8)	7706(4)	8890(3)	21.7(17)
C33E	-1491(10)	7662(5)	9355(4)	30(2)
Ru1F	6184.4(5)	3613.1(3)	8580.8(2)	16.07(15)
O1F	4320(5)	4153(4)	7920(2)	29.2(14)
N1F	5878(8)	3744(4)	9639(3)	31.1(19)
N2F	4655(6)	4479(5)	9299(3)	29.1(19)
N3F	7466(7)	4841(4)	8104(3)	23.4(16)
N4F	7923(7)	4776(4)	8840(3)	22.0(16)
C1F	5016(7)	3993(4)	8208(3)	19.2(16)
C2F	5499(8)	3960(4)	9220(3)	23.6(19)
C3F	5244(10)	4068(5)	9985(3)	35(2)
C4F	4493(8)	4511(5)	9791(3)	32(2)
C5F	3822(8)	4797(5)	8970(3)	22.5(17)
C6F	2750(8)	4470(5)	8894(3)	24.8(18)
C7F	2505(9)	3754(5)	9098(4)	32(2)
C8F	1439(12)	3757(7)	9441(4)	54(3)
C9F	2302(9)	3241(5)	8714(4)	33(2)
C10F	1929(9)	4811(6)	8626(4)	33(2)
C11F	2137(10)	5462(6)	8434(4)	35(2)
C12F	3187(9)	5758(5)	8516(3)	31(2)
C13F	4059(8)	5442(5)	8784(3)	26.9(18)
C14F	5229(9)	5793(5)	8893(4)	30(2)
C15F	5106(13)	6227(7)	9334(4)	50(3)

Table S18 Fractional Atomic Coordinates (×10^4) and Equivalent Isotropic Displacement Parameters (Å^2×10^3) for 4. U_{eq} is defined as 1/3 of the trace of the orthogonalised U_{IJ} tensor.

Atom	*x*	*y*	*z*	U(eq)
C16F	5686(9)	6242(4)	8468(4)	28(2)
C17F	7250(8)	4470(5)	8507(3)	23(2)
C18F	6959(8)	4720(5)	7635(3)	26(2)
C19F	8247(8)	5377(5)	8185(4)	29(2)
C20F	8611(9)	5881(5)	7799(4)	38(2)
C21F	8533(8)	5341(5)	8649(4)	27(2)
C22F	9256(9)	5791(5)	8942(4)	36(2)
C23F	8046(10)	4570(5)	9341(4)	33(2)
C24F	6069(7)	2509(4)	8822(3)	22.1(17)
C25F	5262(9)	2236(5)	9210(3)	28(2)
C26F	7253(8)	2714(4)	8898(3)	22.8(17)
C27F	7895(10)	2653(6)	9359(4)	31(2)
C28F	7744(7)	2897(4)	8444(3)	20.3(17)
C29F	8977(8)	3128(5)	8348(4)	31(2)
C30F	6869(7)	2798(4)	8088(3)	21.2(17)
C31F	7017(8)	2843(5)	7556(3)	25.9(19)
C32F	5823(7)	2540(4)	8331(3)	22.7(17)
C33F	4739(8)	2316(4)	8072(3)	25.1(19)
Ru1G	-42.6(7)	4944.5(4)	1287.3(3)	24.67(17)
O1G	1320(10)	5506(4)	2104(3)	65(3)
N1G	923(6)	5154(4)	298(3)	29.8(19)
N2G	1697(8)	5926(5)	721(3)	41(2)
N3G	-1793(9)	6022(4)	834(3)	36(2)
N4G	-1721(9)	6117(5)	1593(3)	41(2)

Table S18 Fractional Atomic Coordinates (×10^4) and Equivalent Isotropic Displacement Parameters (Å^2×10^3) for 4. U_{eq} is defined as 1/3 of the trace of the orthogonalised U_{IJ} tensor.

Atom	*x*	*y*	*z*	U(eq)
C1G	862(10)	5379(6)	1755(3)	38(2)
C2G	909(8)	5359(5)	725(3)	29(2)
C3G	1624(10)	5522(6)	2(4)	43(3)
C4G	2153(10)	6016(6)	261(4)	46(3)
C5G	2215(11)	6272(5)	1122(4)	41(2)
C6G	3300(13)	5984(6)	1268(5)	55(3)
C7G	3825(12)	5327(7)	1096(5)	59(3)
C8G	4957(13)	5491(7)	810(5)	64(3)
C9G	4050(13)	4809(8)	1487(5)	65(4)
C10G	3918(15)	6329(7)	1637(5)	67(4)
C11G	3476(15)	6942(7)	1810(5)	70(4)
C12G	2402(15)	7208(7)	1637(5)	64(3)
C13G	1772(13)	6862(6)	1287(5)	56(3)
C14G	595(12)	7174(6)	1101(5)	53(3)
C15G	860(18)	7554(9)	643(7)	97(6)
C16G	90(20)	7658(8)	1447(7)	100(6)
C17G	-1274(10)	5751(5)	1227(4)	33(2)
C18G	-1659(11)	5776(6)	361(4)	41(3)
C19G	-2529(10)	6573(5)	949(4)	40(2)
C20G	-3174(12)	6985(6)	572(5)	57(3)
C21G	-2513(11)	6620(5)	1428(4)	44(2)
C22G	-3134(13)	7111(6)	1745(5)	61(4)
C23G	-1461(13)	6014(6)	2094(4)	51(3)
C24G	-886(10)	4104(5)	1722(4)	37(2)

Table S18 Fractional Atomic Coordinates (×10[4]) and Equivalent Isotropic Displacement Parameters (Å[2]×10[3]) for 4. U_{eq} is defined as 1/3 of the trace of the orthogonalised U_{IJ} tensor.

Atom	*x*	*y*	*z*	U(eq)
C25G	-1444(15)	4140(7)	2214(5)	66(4)
C26G	287(9)	3902(4)	1633(3)	29.6(19)
C27G	1190(11)	3721(5)	2004(4)	42(3)
C28G	425(8)	3850(4)	1123(3)	23.5(17)
C29G	1498(11)	3608(7)	871(5)	53(3)
C30G	-686(9)	3995(5)	911(4)	34(2)
C31G	-968(13)	3902(6)	394(4)	49(3)
C32G	-1492(10)	4141(5)	1282(5)	40(2)
C33G	-2787(11)	4317(7)	1211(6)	68(4)
Ru1H	5739.5(7)	-22.6(4)	1335.3(3)	27.10(18)
O1H	4132(8)	515(4)	2089(3)	52(2)
N1H	5062(6)	195(4)	302(3)	24.0(16)
N2H	4099(7)	960(4)	707(3)	26.9(17)
N3H	7297(9)	1133(4)	1718(3)	41(2)
N4H	7509(8)	1084(4)	961(3)	34.0(18)
C1H	4734(10)	374(5)	1775(4)	34(2)
C2H	4897(8)	406(4)	741(3)	23.0(18)
C3H	4431(8)	598(5)	-9(3)	30(2)
C4H	3817(8)	1075(6)	230(3)	33(2)
C5H	3422(10)	1284(5)	1076(3)	33(2)
C6H	2311(11)	1004(6)	1182(4)	39(2)
C7H	1866(9)	356(6)	975(4)	40(2)
C8H	1719(11)	-208(6)	1342(5)	47(3)
C9H	713(10)	479(6)	700(4)	47(3)

Table S18 Fractional Atomic Coordinates (×10⁴) and Equivalent Isotropic Displacement Parameters (Å²×10³) for 4. U_{eq} is defined as 1/3 of the trace of the orthogonalised U_{IJ} tensor.

Atom	*x*	*y*	*z*	U(eq)
C10H	1580(11)	1357(6)	1498(4)	44(2)
C11H	1941(11)	1971(6)	1691(4)	45(3)
C12H	3067(12)	2220(6)	1598(4)	48(3)
C13H	3829(11)	1897(5)	1274(4)	39(2)
C14H	5024(11)	2188(5)	1152(4)	47(2)
C15H	5550(14)	2614(7)	1559(5)	65(4)
C16H	4946(12)	2626(7)	700(5)	62(3)
C17H	6903(10)	787(5)	1324(4)	34(2)
C18H	6896(12)	1013(6)	2208(4)	47(3)
C19H	8085(10)	1638(5)	1596(4)	43(2)
C20H	8599(12)	2113(6)	1957(4)	52(3)
C21H	8210(10)	1637(5)	1126(4)	39(2)
C22H	8896(11)	2073(6)	786(5)	53(3)
C23H	7444(10)	906(6)	465(4)	40(2)
C24H	6452(12)	-882(6)	1814(4)	48(2)
C25H	6756(16)	-877(7)	2333(5)	68(4)
C26H	7212(10)	-796(5)	1428(4)	39(2)
C27H	8513(11)	-601(6)	1466(6)	64(4)
C28H	6639(10)	-926(5)	984(4)	39(2)
C29H	7148(12)	-976(6)	497(5)	52(3)
C30H	5468(9)	-1101(5)	1129(4)	33(2)
C31H	4499(9)	-1370(5)	809(4)	35(2)
C32H	5376(10)	-1068(4)	1616(4)	36(2)
C33H	4319(14)	-1297(6)	1920(5)	60(3)

Table S19 Anisotropic Displacement Parameters (Å^2×10^3) for 4. The Anisotropic displacement factor exponent takes the form: $-2\pi^2[h^2a^{*2}U_{11}+2hka^*b^*U_{12}+\ldots]$.

Atom	U_{11}	U_{22}	U_{33}	U_{23}	U_{13}	U_{12}
Ru1A	24.7(4)	23.7(4)	20.1(4)	-2.7(3)	-4.1(3)	-0.4(3)
O1A	77(6)	49(5)	32(4)	-2(3)	7(4)	-14(4)
N1A	21(4)	30(4)	26(4)	1(3)	-2(3)	6(3)
N2A	27(4)	40(5)	24(4)	-4(3)	7(3)	-10(4)
N3A	23(4)	22(4)	41(4)	-1(3)	-13(3)	5(3)
N4A	57(6)	54(6)	41(4)	6(4)	-18(4)	23(5)
C1A	48(6)	27(5)	17(4)	1(4)	6(4)	-11(4)
C2A	15(4)	24(4)	25(4)	2(3)	-1(3)	5(3)
C3A	26(5)	40(5)	21(4)	0(4)	1(4)	2(4)
C4A	33(5)	31(5)	31(5)	-10(4)	1(4)	-9(4)
C5A	41(5)	21(4)	31(5)	-7(3)	12(4)	-15(3)
C6A	50(5)	15(4)	44(5)	-7(3)	7(4)	-3(4)
C7A	50(6)	17(4)	79(7)	0(4)	21(5)	2(4)
C8A	78(10)	54(8)	103(9)	-31(7)	42(7)	-11(7)
C9A	83(10)	37(7)	130(11)	39(7)	16(8)	11(7)
C10A	58(6)	20(4)	50(6)	8(4)	11(5)	-1(4)
C11A	65(6)	32(5)	41(6)	-2(4)	15(5)	-8(4)
C12A	65(7)	35(5)	37(5)	1(4)	20(5)	-1(5)
C13A	41(5)	26(4)	24(4)	0(3)	5(4)	-2(3)
C14A	32(5)	36(5)	49(6)	8(4)	7(4)	-4(4)
C15A	45(6)	56(7)	37(5)	7(5)	9(5)	-4(5)
C16A	35(6)	48(6)	61(7)	0(5)	10(5)	12(5)
C17A	18(4)	29(5)	42(5)	4(4)	-11(4)	3(4)
C18A	45(6)	37(6)	38(5)	0(4)	4(4)	19(5)

Table S19 Anisotropic Displacement Parameters (Å^2×10^3) for 4. The Anisotropic displacement factor exponent takes the form: $-2\pi^2[h^2a^{*2}U_{11}+2hka^*b^*U_{12}+\ldots]$.

Atom	U_{11}	U_{22}	U_{33}	U_{23}	U_{13}	U_{12}
C19A	19(4)	22(4)	61(5)	4(4)	-9(4)	2(3)
C20A	35(6)	53(7)	82(8)	-15(6)	-4(5)	18(5)
C21A	38(6)	42(5)	65(6)	12(4)	-13(4)	13(5)
C22A	55(8)	63(8)	95(10)	28(7)	-25(7)	19(7)
C23A	114(13)	65(9)	53(7)	4(6)	-17(7)	35(9)
C24A	21(4)	25(4)	28(4)	2(3)	3(3)	-10(3)
C25A	30(2)	29(2)	31(2)	2.3(14)	1.3(14)	-1.9(14)
C26A	40(5)	24(4)	26(4)	-6(3)	-2(3)	2(4)
C27A	68(7)	32(6)	41(6)	-8(4)	18(5)	2(5)
C28A	44(5)	22(5)	39(5)	-7(4)	-12(4)	-7(4)
C29A	93(10)	44(7)	55(7)	-8(5)	-43(7)	9(7)
C30A	36(5)	21(5)	50(5)	-5(4)	-3(4)	-10(4)
C31A	41(6)	42(7)	90(10)	-10(6)	3(5)	-8(5)
C32A	31(4)	24(4)	31(4)	-3(3)	9(3)	-15(3)
C33A	75(8)	42(6)	28(5)	-7(4)	19(5)	-18(6)
Ru1B	13.9(4)	11.7(3)	17.1(3)	-1.6(3)	1.1(3)	-1.1(3)
O1B	25(3)	26(3)	24(3)	-1(3)	-3(3)	-8(3)
N1B	25(3)	22(3)	23(3)	0(2)	3(2)	0(2)
N2B	25(3)	22(3)	23(3)	0(2)	3(2)	0(2)
N3B	17(4)	18(4)	35(4)	6(3)	-5(3)	-3(3)
N4B	12(3)	16(3)	31(4)	0(3)	1(3)	-1(3)
C1B	18(4)	14(4)	13(4)	1(3)	4(3)	1(3)
C2B	27(5)	22(4)	20(4)	0(3)	-1(4)	4(4)
C3B	55(6)	21(4)	16(4)	-4(3)	6(4)	-3(4)

Table S19 Anisotropic Displacement Parameters (Å^2×10^3) for 4. The Anisotropic displacement factor exponent takes the form: $-2\pi^2[h^2a^{*2}U_{11}+2hka^*b^*U_{12}+\ldots]$.

Atom	U_{11}	U_{22}	U_{33}	U_{23}	U_{13}	U_{12}
C4B	37(5)	33(5)	22(5)	0(4)	10(4)	-4(4)
C5B	27(4)	18(4)	23(4)	6(3)	3(3)	-9(3)
C6B	31(4)	19(4)	29(4)	8(3)	4(3)	-8(3)
C7B	29(4)	18(4)	37(5)	1(3)	3(3)	-3(3)
C8B	37(4)	29(4)	38(4)	5(3)	11(4)	-3(4)
C9B	43(6)	33(5)	40(5)	10(4)	4(4)	8(5)
C10B	33(4)	24(4)	31(5)	3(4)	6(3)	-9(3)
C11B	30(4)	39(5)	34(5)	2(4)	0(4)	-14(4)
C12B	27(4)	37(5)	27(5)	14(3)	-1(3)	-5(4)
C13B	25(4)	34(4)	22(4)	12(3)	6(3)	-3(3)
C14B	35(5)	31(4)	33(5)	4(4)	7(4)	7(4)
C15B	42(6)	58(7)	32(5)	13(5)	14(5)	10(5)
C16B	23(5)	41(5)	44(6)	11(4)	19(4)	10(4)
C17B	12(3)	18(4)	25(4)	-1(3)	4(3)	-6(3)
C18B	34(6)	42(6)	34(6)	-3(5)	-13(5)	5(5)
C19B	17(4)	11(3)	36(4)	9(3)	2(3)	-3(3)
C20B	29(5)	26(5)	62(7)	13(5)	-13(5)	4(4)
C21B	13(4)	21(4)	47(6)	-1(4)	4(4)	-1(3)
C22B	25(5)	43(6)	56(7)	-4(5)	9(5)	14(4)
C23B	25(5)	26(5)	23(5)	-5(4)	4(4)	7(4)
C24B	18(4)	15(4)	25(4)	2(3)	3(3)	-1(3)
C25B	31(5)	25(5)	27(4)	2(4)	9(4)	-5(4)
C26B	25(4)	22(4)	25(3)	3(3)	-1(3)	-5(3)
C27B	33(5)	23(5)	31(5)	1(4)	-12(4)	-11(4)

Table S19 Anisotropic Displacement Parameters (Å^2×10^3) for 4. The Anisotropic displacement factor exponent takes the form: $-2\pi^2[h^2a^{*2}U_{11}+2hka^*b^*U_{12}+\ldots]$.

Atom	U_{11}	U_{22}	U_{33}	U_{23}	U_{13}	U_{12}
C28B	19(4)	15(4)	27(4)	6(3)	2(3)	-8(3)
C29B	14(4)	35(5)	37(5)	5(4)	-1(3)	-4(3)
C30B	14(3)	18(4)	24(4)	4(3)	3(3)	-4(3)
C31B	22(4)	24(5)	30(4)	2(3)	4(3)	-3(4)
C32B	17(3)	15(3)	24(3)	0(3)	2(3)	0(3)
C33B	20(3)	27(4)	26(4)	4(3)	2(3)	1(3)
Ru1C	30.6(4)	19.8(4)	21.4(4)	-2.2(3)	4.1(3)	-4.3(3)
O1C	61(5)	49(5)	25(4)	5(3)	-15(4)	8(4)
N1C	14(3)	34(4)	20(3)	3(3)	0(3)	-6(3)
N2C	29(4)	24(4)	31(4)	-11(3)	-14(3)	3(3)
N3C	23(4)	25(4)	43(4)	-2(3)	13(3)	-2(3)
N4C	43(5)	34(4)	37(4)	6(3)	18(3)	-5(4)
C1C	39(6)	23(5)	32(5)	4(4)	10(4)	-4(4)
C2C	17(4)	15(4)	22(4)	-2(3)	-2(3)	-3(3)
C3C	19(4)	38(5)	22(4)	-2(4)	0(3)	-7(4)
C4C	13(4)	36(5)	20(4)	-4(4)	2(3)	6(3)
C5C	37(5)	19(4)	26(4)	2(3)	-12(4)	4(3)
C6C	38(5)	24(4)	32(5)	-5(3)	-10(4)	1(3)
C7C	28(5)	30(4)	42(5)	2(4)	-19(4)	2(4)
C8C	31(5)	44(6)	52(6)	-1(5)	-15(4)	5(4)
C9C	26(5)	23(4)	55(6)	-1(4)	-5(4)	-5(4)
C10C	43(5)	35(5)	40(5)	7(4)	-16(4)	2(4)
C11C	65(6)	26(5)	40(6)	9(4)	-20(5)	5(4)
C12C	65(6)	20(4)	35(5)	7(4)	-9(4)	2(4)

Table S19 Anisotropic Displacement Parameters (Å^2×10^3) for 4. The Anisotropic displacement factor exponent takes the form: $-2\pi^2[h^2a^{*2}U_{11}+2hka^*b^*U_{12}+\ldots]$.

Atom	U_{11}	U_{22}	U_{33}	U_{23}	U_{13}	U_{12}
C13C	41(5)	17(4)	31(5)	1(3)	2(4)	3(3)
C14C	49(5)	18(4)	51(5)	8(4)	-2(4)	-3(4)
C15C	63(7)	31(5)	59(7)	12(5)	7(6)	-6(5)
C16C	45(6)	32(5)	57(6)	-3(4)	-9(5)	-9(5)
C17C	24(4)	27(4)	37(4)	-1(3)	17(3)	-10(4)
C18C	33(5)	35(5)	37(5)	-4(4)	1(4)	-7(4)
C19C	29(5)	18(4)	62(5)	1(4)	16(4)	-5(3)
C20C	38(6)	46(6)	85(8)	-16(6)	14(6)	-19(5)
C21C	30(5)	29(5)	59(5)	6(4)	19(4)	-2(4)
C22C	55(7)	41(6)	64(7)	15(5)	22(6)	-9(5)
C23C	61(7)	47(7)	38(5)	4(4)	24(5)	-10(6)
C24C	27(4)	38(6)	42(5)	-6(4)	-2(3)	2(4)
C25C	42(6)	30(5)	58(6)	-3(5)	-24(5)	9(5)
C26C	41(5)	17(4)	56(5)	-8(4)	7(4)	2(4)
C27C	32(5)	40(6)	118(11)	-18(7)	17(6)	4(5)
C28C	57(5)	25(5)	40(5)	-12(4)	8(4)	-1(4)
C29C	97(10)	37(6)	49(6)	-8(5)	18(6)	7(7)
C30C	40(5)	23(4)	36(4)	-6(3)	-3(3)	5(4)
C31C	71(7)	28(6)	69(8)	-11(5)	-34(6)	0(5)
C32C	21(4)	35(5)	34(4)	-8(4)	-5(3)	10(3)
C33C	30(5)	31(5)	59(6)	26(5)	11(5)	17(4)
Ru1D	11.0(3)	12.1(3)	20.1(3)	-2.9(3)	2.2(3)	0.1(3)
O1D	17(3)	42(4)	28(3)	0(3)	6(3)	8(3)
N1D	25(4)	28(4)	21(4)	-1(3)	-4(3)	5(3)

Table S19 Anisotropic Displacement Parameters ($Å^2 \times 10^3$) for 4. The Anisotropic displacement factor exponent takes the form: $-2\pi^2[h^2a^{*2}U_{11}+2hka^*b^*U_{12}+…]$.

Atom	U_{11}	U_{22}	U_{33}	U_{23}	U_{13}	U_{12}
N2D	21(4)	43(5)	20(4)	-1(3)	3(3)	4(3)
N3D	20(4)	25(4)	27(4)	-1(3)	3(3)	0(3)
N4D	22(4)	15(4)	30(4)	-2(3)	1(3)	3(3)
C1D	22(4)	23(4)	19(4)	7(3)	-2(4)	3(4)
C2D	15(4)	9(3)	23(4)	2(3)	-6(3)	-4(3)
C3D	37(5)	34(5)	22(4)	1(4)	6(4)	4(4)
C4D	28(5)	33(5)	25(5)	13(4)	-2(4)	4(4)
C5D	20(4)	28(4)	12(4)	12(3)	3(3)	3(3)
C6D	21(4)	39(4)	21(4)	5(3)	-1(3)	6(3)
C7D	26(5)	36(5)	31(5)	4(4)	4(4)	-7(4)
C8D	57(7)	42(6)	40(6)	3(5)	-14(5)	-8(5)
C9D	34(6)	36(5)	38(5)	12(4)	-4(4)	-8(4)
C10D	24(4)	45(5)	26(5)	8(4)	3(4)	11(4)
C11D	34(5)	48(5)	26(5)	4(4)	6(4)	16(4)
C12D	35(4)	29(5)	36(5)	-3(4)	4(4)	15(4)
C13D	23(3)	25(4)	27(4)	1(3)	-3(3)	15(3)
C14D	27(4)	20(4)	46(5)	1(4)	4(4)	13(3)
C15D	44(5)	26(4)	48(5)	-8(4)	2(4)	1(4)
C16D	60(7)	34(6)	45(6)	8(4)	6(5)	-13(5)
C17D	19(4)	17(4)	31(5)	-1(4)	6(4)	-4(4)
C18D	18(4)	35(5)	24(5)	-10(4)	-6(3)	4(4)
C19D	25(4)	16(4)	29(5)	4(3)	-2(4)	-5(3)
C20D	42(6)	35(6)	41(6)	-12(5)	-1(5)	-9(5)
C21D	21(4)	15(4)	44(6)	3(4)	0(4)	-4(3)

Table S19 Anisotropic Displacement Parameters (Å^2×10^3) for 4. The Anisotropic displacement factor exponent takes the form: $-2\pi^2[h^2a^{*2}U_{11}+2hka^*b^*U_{12}+\ldots]$.

Atom	U_{11}	U_{22}	U_{33}	U_{23}	U_{13}	U_{12}
C22D	28(5)	19(5)	49(6)	8(4)	5(4)	-9(4)
C23D	43(6)	21(5)	29(5)	4(4)	11(4)	-9(4)
C24D	13(3)	18(4)	30(4)	-2(3)	-2(3)	4(3)
C25D	27(5)	28(5)	29(4)	4(4)	0(3)	0(4)
C26D	14(3)	17(4)	29(4)	3(3)	1(3)	2(3)
C27D	12(4)	27(5)	43(5)	4(4)	1(3)	-1(3)
C28D	18(4)	25(4)	30(4)	1(3)	2(3)	10(3)
C29D	33(5)	19(5)	36(5)	1(4)	11(4)	12(4)
C30D	19(4)	11(4)	28(4)	1(3)	-3(3)	5(3)
C31D	23(4)	24(4)	36(4)	3(3)	-4(3)	1(3)
C32D	19(3)	12(3)	31(3)	-2(3)	1(3)	4(3)
C33D	19(4)	23(4)	33(5)	-1(4)	2(3)	0(3)
Ru1E	14.3(3)	13.7(3)	18.1(3)	-0.9(3)	3.1(3)	-3.8(3)
O1E	20(3)	36(4)	24(3)	3(3)	5(3)	-4(3)
N1E	42(5)	29(4)	23(4)	7(3)	7(4)	-9(4)
N2E	29(4)	11(3)	21(4)	2(3)	-1(3)	-1(3)
N3E	15(3)	17(3)	38(4)	-6(3)	15(3)	-6(3)
N4E	13(3)	24(4)	32(4)	-2(3)	3(3)	0(3)
C1E	13(4)	21(4)	21(4)	-3(3)	-1(3)	0(3)
C2E	30(5)	19(4)	17(4)	-2(3)	4(4)	-11(4)
C3E	58(7)	31(5)	19(5)	-1(4)	-4(5)	-9(5)
C4E	53(7)	35(5)	24(5)	-5(4)	-3(5)	-9(5)
C5E	24(4)	20(4)	23(4)	-5(3)	-1(3)	-10(3)
C6E	27(4)	11(3)	29(4)	-2(3)	0(3)	-8(3)

Table S19 Anisotropic Displacement Parameters (Å^2×10^3) for 4. The Anisotropic displacement factor exponent takes the form: $-2\pi^2[h^2a^{*2}U_{11}+2hka^*b^*U_{12}+\ldots]$.

Atom	U_{11}	U_{22}	U_{33}	U_{23}	U_{13}	U_{12}
C7E	28(4)	14(4)	38(5)	5(3)	3(3)	-4(3)
C8E	53(7)	34(6)	43(5)	-6(4)	2(5)	13(5)
C9E	56(5)	14(4)	51(5)	3(3)	-12(4)	-1(4)
C10E	35(4)	24(4)	28(5)	-2(4)	3(4)	-12(3)
C11E	32(5)	31(4)	37(5)	-4(4)	7(4)	-16(4)
C12E	26(4)	41(5)	32(5)	-15(4)	7(4)	-10(4)
C13E	24(4)	29(4)	25(4)	-8(3)	0(3)	-6(3)
C14E	38(5)	33(5)	34(5)	-6(4)	4(4)	1(4)
C15E	51(6)	55(7)	40(6)	-17(5)	-11(5)	7(5)
C16E	31(5)	32(5)	48(6)	-8(4)	-12(5)	7(4)
C17E	21(4)	19(4)	31(4)	-3(3)	4(3)	-6(3)
C18E	33(6)	43(6)	34(5)	-8(4)	23(4)	-11(5)
C19E	19(4)	22(4)	48(5)	-3(3)	6(3)	-6(3)
C20E	31(5)	38(6)	70(7)	-15(5)	14(5)	4(5)
C21E	16(4)	18(4)	47(5)	2(3)	3(3)	-2(3)
C22E	31(5)	37(5)	59(6)	4(5)	1(5)	12(4)
C23E	20(4)	20(4)	29(4)	4(3)	-2(3)	3(3)
C24E	22(4)	14(4)	26(4)	1(3)	1(3)	-5(3)
C25E	21(2)	20(2)	21(2)	2.1(13)	-1.3(14)	-2.7(14)
C26E	17(3)	14(4)	23(4)	4(3)	2(3)	-3(3)
C27E	17(4)	20(4)	31(5)	-5(3)	4(3)	-1(3)
C28E	14(3)	12(4)	27(4)	0(3)	3(3)	-6(3)
C29E	22(4)	26(5)	28(4)	-6(3)	0(3)	2(4)
C30E	18(4)	21(4)	27(4)	-5(3)	3(3)	-8(3)

Table S19 Anisotropic Displacement Parameters (Å^2×10^3) for 4. The Anisotropic displacement factor exponent takes the form: $-2\pi^2[h^2a^{*2}U_{11}+2hka^*b^*U_{12}+\ldots]$.

Atom	U_{11}	U_{22}	U_{33}	U_{23}	U_{13}	U_{12}
C31E	10(4)	28(5)	42(5)	-6(4)	5(3)	0(3)
C32E	23(4)	16(4)	26(4)	3(3)	8(3)	-8(3)
C33E	32(5)	26(5)	30(5)	1(4)	13(4)	-11(4)
Ru1F	13.4(3)	14.2(3)	20.6(4)	0.7(3)	1.9(3)	1.9(3)
O1F	18(3)	40(4)	30(3)	0(3)	-3(3)	5(3)
N1F	40(5)	26(4)	27(4)	6(3)	1(3)	13(4)
N2F	14(4)	56(6)	18(4)	-9(4)	-2(3)	13(4)
N3F	23(4)	25(4)	22(4)	4(3)	6(3)	0(3)
N4F	21(4)	19(4)	25(4)	5(3)	5(3)	1(3)
C1F	15(4)	18(4)	24(4)	-2(3)	6(3)	3(3)
C2F	25(5)	24(4)	23(4)	-3(3)	4(4)	-5(4)
C3F	53(6)	31(5)	20(4)	5(4)	5(4)	8(5)
C4F	28(5)	45(6)	22(5)	-6(4)	1(4)	11(4)
C5F	20(4)	28(4)	20(4)	-4(3)	-6(3)	12(3)
C6F	22(4)	35(4)	18(4)	-8(3)	3(3)	3(3)
C7F	24(5)	42(5)	30(5)	-6(4)	2(4)	-5(4)
C8F	62(7)	57(7)	44(6)	-9(5)	27(6)	-12(6)
C9F	24(5)	39(5)	37(5)	-4(4)	11(4)	-9(4)
C10F	20(4)	47(5)	33(5)	-7(4)	-1(4)	9(4)
C11F	39(5)	40(5)	27(5)	-2(4)	-6(4)	14(4)
C12F	36(4)	25(4)	31(5)	3(4)	1(4)	20(3)
C13F	28(4)	29(4)	24(4)	4(3)	1(3)	16(3)
C14F	35(5)	13(4)	42(5)	11(3)	-1(4)	7(3)
C15F	59(8)	49(7)	43(6)	-1(5)	2(5)	-6(6)

Table S19 Anisotropic Displacement Parameters (Å^2×10^3) for 4. The Anisotropic displacement factor exponent takes the form: $-2\pi^2[h^2a^{*2}U_{11}+2hka^*b^*U_{12}+\ldots]$.

Atom	U_{11}	U_{22}	U_{33}	U_{23}	U_{13}	U_{12}
C16F	29(4)	12(3)	43(4)	3(3)	9(4)	12(3)
C17F	15(4)	25(5)	30(5)	0(4)	4(4)	5(4)
C18F	22(5)	26(5)	29(5)	9(4)	2(4)	3(4)
C19F	25(5)	21(5)	39(5)	4(4)	3(4)	-1(4)
C20F	33(5)	29(5)	52(6)	12(5)	6(5)	-13(4)
C21F	17(4)	21(5)	44(6)	4(4)	-2(4)	2(3)
C22F	25(5)	28(5)	56(7)	-6(5)	0(5)	0(4)
C23F	43(6)	24(5)	32(5)	0(4)	-10(5)	0(4)
C24F	19(4)	12(4)	35(4)	6(3)	3(3)	8(3)
C25F	28(2)	28(2)	28(2)	1.5(14)	2.7(14)	0.8(14)
C26F	23(4)	11(4)	34(4)	4(3)	3(3)	8(3)
C27F	32(5)	28(5)	32(5)	9(4)	-5(4)	14(4)
C28F	11(3)	19(4)	31(4)	-3(3)	3(3)	5(3)
C29F	13(4)	30(5)	48(6)	-2(4)	5(4)	0(3)
C30F	10(3)	26(4)	27(4)	-3(3)	6(3)	1(3)
C31F	28(5)	25(5)	25(4)	-2(3)	9(3)	-2(4)
C32F	11(3)	20(4)	37(4)	-3(3)	8(3)	7(3)
C33F	19(4)	14(4)	42(5)	5(4)	1(4)	-4(3)
Ru1G	35.3(4)	17.1(4)	21.6(4)	-0.8(3)	6.4(3)	-2.9(3)
O1G	113(8)	42(5)	39(4)	-5(3)	-26(5)	3(5)
N1G	16(4)	44(5)	29(4)	13(4)	12(3)	11(3)
N2G	38(5)	46(6)	38(5)	9(4)	-2(4)	-5(4)
N3G	43(5)	20(4)	46(4)	4(3)	17(4)	-3(4)
N4G	55(5)	30(4)	37(4)	-4(3)	16(4)	-3(4)

Table S19 Anisotropic Displacement Parameters (Å^2×10^3) for 4. The Anisotropic displacement factor exponent takes the form: $-2\pi^2[h^2a^{*2}U_{11}+2hka^*b^*U_{12}+\ldots]$.

Atom	U_{11}	U_{22}	U_{33}	U_{23}	U_{13}	U_{12}
C1G	54(6)	47(6)	14(4)	-6(4)	3(4)	-11(5)
C2G	30(5)	23(4)	33(5)	5(4)	1(4)	7(4)
C3G	34(6)	61(7)	34(6)	-4(5)	-6(5)	13(5)
C4G	40(6)	54(7)	43(6)	19(5)	-1(5)	-9(5)
C5G	61(6)	28(4)	34(5)	17(4)	-13(4)	-19(4)
C6G	58(6)	40(6)	69(8)	-5(5)	-9(5)	-10(5)
C7G	53(7)	54(6)	69(7)	4(5)	-29(5)	-1(5)
C8G	67(7)	62(8)	64(7)	-2(6)	-24(6)	0(6)
C9G	62(8)	67(8)	64(8)	-1(6)	-26(7)	6(7)
C10G	89(9)	47(6)	66(7)	5(5)	-25(7)	-16(6)
C11G	100(9)	42(6)	69(8)	5(5)	-22(7)	-15(6)
C12G	90(8)	37(6)	65(8)	8(5)	-12(6)	-16(6)
C13G	72(7)	34(5)	60(7)	9(4)	2(5)	-15(5)
C14G	63(6)	32(5)	63(7)	10(4)	-8(5)	-13(5)
C15G	111(14)	81(11)	99(10)	43(8)	-8(9)	12(10)
C16G	131(14)	51(8)	118(11)	-7(8)	3(10)	29(10)
C17G	36(5)	23(4)	40(5)	-4(4)	16(4)	-12(4)
C18G	52(7)	34(5)	38(5)	11(4)	5(4)	12(5)
C19G	37(5)	26(4)	57(5)	4(4)	20(4)	-1(4)
C20G	58(7)	40(6)	71(7)	16(5)	24(6)	18(5)
C21G	52(6)	23(4)	59(5)	2(4)	23(4)	1(4)
C22G	82(9)	23(5)	77(8)	6(5)	44(7)	4(5)
C23G	84(9)	27(5)	42(5)	-5(4)	22(5)	8(6)
C24G	53(5)	16(4)	42(5)	7(4)	14(4)	-7(4)

Table S19 Anisotropic Displacement Parameters (Å^2×10^3) for 4. The Anisotropic displacement factor exponent takes the form: $-2\pi^2[h^2a^{*2}U_{11}+2hka^*b^*U_{12}+\ldots]$.

Atom	U_{11}	U_{22}	U_{33}	U_{23}	U_{13}	U_{12}
C25G	99(10)	41(7)	56(7)	10(5)	45(7)	10(7)
C26G	44(5)	18(4)	28(4)	0(3)	-1(3)	-10(4)
C27G	60(6)	26(5)	39(5)	11(4)	-19(5)	-5(5)
C28G	32(4)	13(4)	25(4)	3(3)	0(3)	-1(3)
C29G	40(6)	68(9)	54(7)	-25(6)	2(5)	22(6)
C30G	34(4)	26(5)	41(5)	8(4)	0(3)	-7(4)
C31G	71(8)	35(6)	42(5)	14(4)	-14(5)	-12(6)
C32G	37(5)	19(5)	64(5)	11(4)	10(4)	-16(4)
C33G	43(6)	45(7)	115(12)	15(7)	4(6)	-9(5)
Ru1H	39.9(5)	17.9(4)	23.6(4)	-1.0(3)	-9.0(4)	-2.5(3)
O1H	77(6)	56(5)	24(4)	-5(3)	11(4)	-1(4)
N1H	9(3)	35(4)	28(4)	11(3)	-6(3)	-2(3)
N2H	38(5)	18(4)	25(4)	3(3)	3(3)	-5(3)
N3H	50(5)	35(4)	37(4)	-3(3)	-21(4)	0(4)
N4H	37(4)	24(4)	41(4)	1(3)	-17(3)	0(3)
C1H	44(6)	31(5)	29(5)	-3(4)	-2(5)	4(5)
C2H	30(5)	23(4)	17(4)	-6(3)	-5(3)	-6(4)
C3H	18(4)	43(6)	29(5)	2(4)	5(4)	-3(4)
C4H	19(4)	48(6)	33(5)	8(4)	-2(4)	5(4)
C5H	44(5)	29(4)	25(5)	6(3)	7(4)	7(4)
C6H	49(5)	38(5)	30(5)	2(4)	10(4)	3(4)
C7H	35(5)	41(5)	42(5)	-6(4)	15(4)	2(4)
C8H	44(6)	35(5)	63(7)	-9(5)	0(5)	-4(5)
C9H	40(5)	57(7)	43(6)	-5(5)	13(4)	10(5)

Table S19 Anisotropic Displacement Parameters ($Å^2×10^3$) for 4. The Anisotropic displacement factor exponent takes the form: $-2π^2[h^2a^{*2}U_{11}+2hka^*b^*U_{12}+…]$.

Atom	U_{11}	U_{22}	U_{33}	U_{23}	U_{13}	U_{12}
C10H	56(6)	41(5)	36(5)	2(4)	18(5)	6(4)
C11H	63(6)	35(5)	39(6)	-1(4)	19(5)	8(4)
C12H	70(6)	28(5)	46(6)	-2(4)	6(5)	9(4)
C13H	50(5)	29(5)	37(5)	0(4)	4(4)	4(4)
C14H	50(6)	25(5)	64(6)	2(4)	2(5)	7(4)
C15H	71(8)	40(6)	85(8)	-13(6)	-7(7)	-5(6)
C16H	51(7)	54(7)	81(8)	18(6)	15(6)	-1(6)
C17H	47(6)	19(4)	38(5)	5(3)	-18(4)	2(4)
C18H	60(7)	47(6)	35(5)	-3(4)	-21(5)	-5(5)
C19H	45(5)	28(5)	55(5)	-3(4)	-25(4)	-3(4)
C20H	68(8)	32(5)	56(7)	0(5)	-22(6)	-16(5)
C21H	37(5)	22(4)	58(5)	-2(4)	-21(4)	0(4)
C22H	49(6)	39(6)	72(7)	8(5)	-18(5)	-9(5)
C23H	34(6)	39(6)	46(5)	5(4)	-8(4)	-9(5)
C24H	65(6)	35(6)	41(5)	9(4)	-12(4)	0(5)
C25H	112(12)	42(7)	50(6)	10(5)	-35(7)	-3(8)
C26H	44(5)	10(4)	61(5)	8(4)	-19(4)	5(4)
C27H	46(6)	31(6)	114(11)	15(6)	-30(6)	0(5)
C28H	35(5)	30(5)	51(5)	4(4)	0(4)	3(4)
C29H	52(7)	41(7)	64(7)	0(5)	17(5)	7(6)
C30H	38(5)	23(5)	38(4)	-4(4)	-9(4)	-5(4)
C31H	19(4)	27(5)	58(6)	1(4)	-10(4)	-9(4)
C32H	59(5)	12(4)	36(4)	8(3)	4(4)	0(4)
C33H	87(8)	35(6)	56(7)	11(5)	28(6)	-7(6)

Table S20 Bond Lengths for 4.

Atom	Atom	Length/Å	Atom	Atom	Length/Å
Ru1A	C1A	1.841(9)	Ru1E	C1E	1.840(8)
Ru1A	C2A	2.069(9)	Ru1E	C2E	2.083(9)
Ru1A	C17A	2.080(10)	Ru1E	C17E	2.049(10)
Ru1A	C24A	2.249(9)	Ru1E	C24E	2.241(8)
Ru1A	C26A	2.226(9)	Ru1E	C26E	2.259(8)
Ru1A	C28A	2.242(9)	Ru1E	C28E	2.267(8)
Ru1A	C30A	2.297(10)	Ru1E	C30E	2.287(9)
Ru1A	C32A	2.286(9)	Ru1E	C32E	2.281(8)
O1A	C1A	1.153(11)	O1E	C1E	1.155(10)
N1A	C2A	1.311(11)	N1E	C2E	1.324(11)
N1A	C3A	1.360(12)	N1E	C3E	1.368(13)
N2A	C2A	1.405(12)	N2E	C2E	1.393(11)
N2A	C4A	1.372(12)	N2E	C4E	1.382(12)
N2A	C5A	1.453(12)	N2E	C5E	1.427(11)
N3A	C17A	1.353(14)	N3E	C17E	1.383(12)
N3A	C18A	1.447(13)	N3E	C18E	1.456(13)
N3A	C19A	1.401(12)	N3E	C19E	1.385(13)
N4A	C17A	1.363(13)	N4E	C17E	1.378(12)
N4A	C21A	1.407(16)	N4E	C21E	1.395(11)
N4A	C23A	1.478(18)	N4E	C23E	1.462(12)
C3A	C4A	1.367(14)	C3E	C4E	1.334(15)
C5A	C6A	1.404(14)	C5E	C6E	1.408(13)
C5A	C13A	1.408(14)	C5E	C13E	1.407(13)
C6A	C7A	1.538(15)	C6E	C7E	1.503(13)
C6A	C10A	1.377(15)	C6E	C10E	1.407(13)

Table S20 Bond Lengths for 4.

Atom	Atom	Length/Å	Atom	Atom	Length/Å
C7A	C8A	1.532(19)	C7E	C8E	1.542(14)
C7A	C9A	1.525(19)	C7E	C9E	1.524(14)
C10A	C11A	1.401(16)	C10E	C11E	1.383(15)
C11A	C12A	1.378(16)	C11E	C12E	1.414(15)
C12A	C13A	1.411(14)	C12E	C13E	1.378(14)
C13A	C14A	1.505(15)	C13E	C14E	1.504(14)
C14A	C15A	1.531(16)	C14E	C15E	1.552(15)
C14A	C16A	1.527(16)	C14E	C16E	1.531(14)
C19A	C20A	1.484(16)	C19E	C20E	1.499(14)
C19A	C21A	1.325(16)	C19E	C21E	1.364(15)
C21A	C22A	1.504(15)	C21E	C22E	1.497(14)
C24A	C25A	1.470(13)	C24E	C25E	1.518(12)
C24A	C26A	1.417(13)	C24E	C26E	1.421(12)
C24A	C32A	1.429(13)	C24E	C32E	1.421(13)
C26A	C27A	1.476(14)	C26E	C27E	1.503(11)
C26A	C28A	1.419(15)	C26E	C28E	1.434(12)
C28A	C29A	1.533(15)	C28E	C29E	1.509(12)
C28A	C30A	1.384(15)	C28E	C30E	1.420(12)
C30A	C31A	1.518(16)	C30E	C31E	1.505(12)
C30A	C32A	1.395(15)	C30E	C32E	1.433(13)
C32A	C33A	1.506(13)	C32E	C33E	1.502(12)
Ru1B	C1B	1.842(8)	Ru1F	C1F	1.836(9)
Ru1B	C2B	2.083(9)	Ru1F	C2F	2.083(9)
Ru1B	C17B	2.075(9)	Ru1F	C17F	2.075(10)
Ru1B	C24B	2.240(8)	Ru1F	C24F	2.244(8)

Table S20 Bond Lengths for 4.

Atom	Atom	Length/Å	Atom	Atom	Length/Å
Ru1B	C26B	2.282(9)	Ru1F	C26F	2.291(9)
Ru1B	C28B	2.311(8)	Ru1F	C28F	2.290(8)
Ru1B	C30B	2.264(8)	Ru1F	C30F	2.267(8)
Ru1B	C32B	2.252(8)	Ru1F	C32F	2.261(9)
O1B	C1B	1.148(10)	O1F	C1F	1.167(11)
N1B	C2B	1.345(12)	N1F	C2F	1.313(12)
N1B	C3B	1.369(12)	N1F	C3F	1.373(12)
N2B	C2B	1.389(12)	N2F	C2F	1.413(12)
N2B	C4B	1.384(12)	N2F	C4F	1.397(12)
N2B	C5B	1.424(12)	N2F	C5F	1.451(11)
N3B	C17B	1.361(12)	N3F	C17F	1.350(12)
N3B	C18B	1.469(13)	N3F	C18F	1.462(12)
N3B	C19B	1.393(12)	N3F	C19F	1.397(12)
N4B	C17B	1.347(11)	N4F	C17F	1.359(12)
N4B	C21B	1.377(11)	N4F	C21F	1.399(12)
N4B	C23B	1.474(11)	N4F	C23F	1.461(12)
C3B	C4B	1.339(14)	C3F	C4F	1.321(14)
C5B	C6B	1.421(13)	C5F	C6F	1.396(13)
C5B	C13B	1.394(13)	C5F	C13F	1.376(14)
C6B	C7B	1.524(14)	C6F	C7F	1.523(14)
C6B	C10B	1.368(14)	C6F	C10F	1.362(14)
C7B	C8B	1.547(13)	C7F	C8F	1.548(14)
C7B	C9B	1.535(13)	C7F	C9F	1.507(14)
C10B	C11B	1.392(15)	C10F	C11F	1.388(15)
C11B	C12B	1.374(15)	C11F	C12F	1.351(15)

Table S20 Bond Lengths for 4.

Atom	Atom	Length/Å	Atom	Atom	Length/Å
C12B	C13B	1.390(14)	C12F	C13F	1.380(13)
C13B	C14B	1.514(14)	C13F	C14F	1.533(14)
C14B	C15B	1.538(14)	C14F	C15F	1.521(15)
C14B	C16B	1.524(14)	C14F	C16F	1.554(13)
C19B	C20B	1.503(13)	C19F	C20F	1.503(13)
C19B	C21B	1.341(14)	C19F	C21F	1.344(14)
C21B	C22B	1.504(14)	C21F	C22F	1.470(14)
C24B	C25B	1.504(12)	C24F	C25F	1.513(13)
C24B	C26B	1.426(13)	C24F	C26F	1.424(13)
C24B	C32B	1.422(12)	C24F	C32F	1.405(13)
C26B	C27B	1.512(13)	C26F	C27F	1.488(14)
C26B	C28B	1.430(13)	C26F	C28F	1.430(13)
C28B	C29B	1.504(13)	C28F	C29F	1.497(12)
C28B	C30B	1.416(12)	C28F	C30F	1.427(12)
C30B	C31B	1.500(12)	C30F	C31F	1.505(12)
C30B	C32B	1.438(11)	C30F	C32F	1.456(12)
C32B	C33B	1.487(12)	C32F	C33F	1.504(12)
Ru1C	C1C	1.846(10)	Ru1G	C1G	1.886(10)
Ru1C	C2C	2.068(8)	Ru1G	C2G	2.063(9)
Ru1C	C17C	2.074(9)	Ru1G	C17G	2.106(11)
Ru1C	C24C	2.275(11)	Ru1G	C24G	2.237(9)
Ru1C	C26C	2.263(11)	Ru1G	C26G	2.260(9)
Ru1C	C28C	2.261(10)	Ru1G	C28G	2.256(8)
Ru1C	C30C	2.244(9)	Ru1G	C30G	2.276(10)
Ru1C	C32C	2.300(10)	Ru1G	C32G	2.278(10)

Table S20 Bond Lengths for 4.

Atom	Atom	Length/Å	Atom	Atom	Length/Å
O1C	C1C	1.160(12)	O1G	C1G	1.143(12)
N1C	C2C	1.344(11)	N1G	C2G	1.276(12)
N1C	C3C	1.370(12)	N1G	C3G	1.347(14)
N2C	C2C	1.423(11)	N2G	C2G	1.424(13)
N2C	C4C	1.405(12)	N2G	C4G	1.399(14)
N2C	C5C	1.452(12)	N2G	C5G	1.452(14)
N3C	C17C	1.350(13)	N3G	C17G	1.348(15)
N3C	C18C	1.427(13)	N3G	C18G	1.431(14)
N3C	C19C	1.413(12)	N3G	C19G	1.403(13)
N4C	C17C	1.385(12)	N4G	C17G	1.360(13)
N4C	C21C	1.377(14)	N4G	C21G	1.400(15)
N4C	C23C	1.479(15)	N4G	C23G	1.447(15)
C3C	C4C	1.338(13)	C3G	C4G	1.363(17)
C5C	C6C	1.408(14)	C5G	C6G	1.411(18)
C5C	C13C	1.366(13)	C5G	C13G	1.347(18)
C6C	C7C	1.520(14)	C6G	C7G	1.502(19)
C6C	C10C	1.401(14)	C6G	C10G	1.434(18)
C7C	C8C	1.515(15)	C7G	C8G	1.55(2)
C7C	C9C	1.526(14)	C7G	C9G	1.495(18)
C10C	C11C	1.381(15)	C10G	C11G	1.39(2)
C11C	C12C	1.378(16)	C11G	C12G	1.41(2)
C12C	C13C	1.389(14)	C12G	C13G	1.404(19)
C13C	C14C	1.533(15)	C13G	C14G	1.55(2)
C14C	C15C	1.551(15)	C14G	C15G	1.50(2)
C14C	C16C	1.532(15)	C14G	C16G	1.48(2)

Table S20 Bond Lengths for 4.

Atom	Atom	Length/Å	Atom	Atom	Length/Å
C19C	C20C	1.486(16)	C19G	C20G	1.503(17)
C19C	C21C	1.351(16)	C19G	C21G	1.348(17)
C21C	C22C	1.523(14)	C21G	C22G	1.499(15)
C24C	C25C	1.501(15)	C24G	C25G	1.522(15)
C24C	C26C	1.419(16)	C24G	C26G	1.415(15)
C24C	C32C	1.440(14)	C24G	C32G	1.414(17)
C26C	C27C	1.512(16)	C26G	C27G	1.499(14)
C26C	C28C	1.429(16)	C26G	C28G	1.445(13)
C28C	C29C	1.511(16)	C28G	C29G	1.491(14)
C28C	C30C	1.382(16)	C28G	C30G	1.421(14)
C30C	C31C	1.513(15)	C30G	C31G	1.502(15)
C30C	C32C	1.458(14)	C30G	C32G	1.421(16)
C32C	C33C	1.460(14)	C32G	C33G	1.523(18)
Ru1D	C1D	1.837(9)	Ru1H	C1H	1.863(10)
Ru1D	C2D	2.069(8)	Ru1H	C2H	2.080(9)
Ru1D	C17D	2.065(9)	Ru1H	C17H	2.063(10)
Ru1D	C24D	2.254(8)	Ru1H	C24H	2.271(11)
Ru1D	C26D	2.283(8)	Ru1H	C26H	2.261(10)
Ru1D	C28D	2.277(9)	Ru1H	C28H	2.279(11)
Ru1D	C30D	2.246(8)	Ru1H	C30H	2.216(9)
Ru1D	C32D	2.243(8)	Ru1H	C32H	2.208(9)
O1D	C1D	1.161(10)	O1H	C1H	1.155(12)
N1D	C2D	1.335(11)	N1H	C2H	1.324(11)
N1D	C3D	1.385(12)	N1H	C3H	1.364(12)
N2D	C2D	1.401(11)	N2H	C2H	1.409(12)

Table S20 Bond Lengths for 4.

Atom	Atom	Length/Å	Atom	Atom	Length/Å
N2D	C4D	1.370(12)	N2H	C4H	1.389(12)
N2D	C5D	1.444(11)	N2H	C5H	1.447(12)
N3D	C17D	1.347(12)	N3H	C17H	1.387(13)
N3D	C18D	1.460(12)	N3H	C18H	1.463(15)
N3D	C19D	1.397(11)	N3H	C19H	1.372(15)
N4D	C17D	1.386(12)	N4H	C17H	1.348(14)
N4D	C21D	1.409(12)	N4H	C21H	1.429(12)
N4D	C23D	1.450(12)	N4H	C23H	1.448(14)
C3D	C4D	1.328(13)	C3H	C4H	1.351(14)
C5D	C6D	1.395(13)	C5H	C6H	1.407(16)
C5D	C13D	1.393(13)	C5H	C13H	1.411(15)
C6D	C7D	1.513(14)	C6H	C7H	1.492(15)
C6D	C10D	1.395(13)	C6H	C10H	1.408(15)
C7D	C8D	1.521(15)	C7H	C8H	1.498(16)
C7D	C9D	1.503(14)	C7H	C9H	1.537(16)
C10D	C11D	1.365(16)	C10H	C11H	1.389(16)
C11D	C12D	1.389(16)	C11H	C12H	1.394(18)
C12D	C13D	1.429(13)	C12H	C13H	1.413(16)
C13D	C14D	1.482(14)	C13H	C14H	1.512(17)
C14D	C15D	1.492(15)	C14H	C15H	1.551(17)
C14D	C16D	1.567(14)	C14H	C16H	1.515(17)
C19D	C20D	1.493(13)	C19H	C20H	1.510(14)
C19D	C21D	1.343(14)	C19H	C21H	1.326(17)
C21D	C22D	1.488(13)	C21H	C22H	1.487(17)
C24D	C25D	1.512(13)	C24H	C25H	1.497(16)

Table S20 Bond Lengths for 4.

Atom	Atom	Length/Å	Atom	Atom	Length/Å
C24D	C26D	1.415(12)	C24H	C26H	1.393(17)
C24D	C32D	1.438(12)	C24H	C32H	1.397(17)
C26D	C27D	1.502(12)	C26H	C27H	1.534(17)
C26D	C28D	1.415(13)	C26H	C28H	1.436(16)
C28D	C29D	1.500(13)	C28H	C29H	1.491(16)
C28D	C30D	1.451(13)	C28H	C30H	1.431(15)
C30D	C31D	1.499(12)	C30H	C31H	1.524(13)
C30D	C32D	1.409(13)	C30H	C32H	1.376(14)
C32D	C33D	1.516(12)	C32H	C33H	1.535(16)

Table S21 Bond Angles for 4.

Atom	Atom	Atom	Angle/˚	Atom	Atom	Atom	Angle/˚
C1A	Ru1A	C2A	95.5(4)	C1E	Ru1E	C2E	94.9(3)
C1A	Ru1A	C17A	94.9(4)	C1E	Ru1E	C17E	94.1(4)
C1A	Ru1A	C24A	115.6(4)	C1E	Ru1E	C24E	120.6(3)
C1A	Ru1A	C26A	89.9(4)	C1E	Ru1E	C26E	92.8(3)
C1A	Ru1A	C28A	100.9(4)	C1E	Ru1E	C28E	99.0(3)
C1A	Ru1A	C30A	135.5(4)	C1E	Ru1E	C30E	132.7(3)
C1A	Ru1A	C32A	150.5(4)	C1E	Ru1E	C32E	153.8(3)
C2A	Ru1A	C17A	89.7(4)	C2E	Ru1E	C24E	93.6(3)
C2A	Ru1A	C24A	95.4(3)	C2E	Ru1E	C26E	122.2(3)
C2A	Ru1A	C26A	126.0(3)	C2E	Ru1E	C28E	155.1(3)
C2A	Ru1A	C28A	155.6(4)	C2E	Ru1E	C30E	132.1(3)
C2A	Ru1A	C30A	128.4(4)	C2E	Ru1E	C32E	98.8(3)
C2A	Ru1A	C32A	97.2(3)	C17E	Ru1E	C2E	90.5(4)

Table S21 Bond Angles for 4.

Atom	Atom	Atom	Angle/˚	Atom	Atom	Atom	Angle/˚
C17A	Ru1A	C24A	148.3(4)	C17E	Ru1E	C24E	144.4(3)
C17A	Ru1A	C26A	143.3(4)	C17E	Ru1E	C26E	145.8(3)
C17A	Ru1A	C28A	106.7(4)	C17E	Ru1E	C28E	108.9(3)
C17A	Ru1A	C30A	92.7(4)	C17E	Ru1E	C30E	90.5(4)
C17A	Ru1A	C32A	111.7(4)	C17E	Ru1E	C32E	107.9(4)
C24A	Ru1A	C30A	60.0(4)	C24E	Ru1E	C26E	36.8(3)
C24A	Ru1A	C32A	36.7(3)	C24E	Ru1E	C28E	61.5(3)
C26A	Ru1A	C24A	36.9(3)	C24E	Ru1E	C30E	61.2(3)
C26A	Ru1A	C28A	37.0(4)	C24E	Ru1E	C32E	36.6(3)
C26A	Ru1A	C30A	60.2(4)	C26E	Ru1E	C28E	36.9(3)
C26A	Ru1A	C32A	61.2(3)	C26E	Ru1E	C30E	61.0(3)
C28A	Ru1A	C24A	61.1(3)	C26E	Ru1E	C32E	61.0(3)
C28A	Ru1A	C30A	35.5(4)	C28E	Ru1E	C30E	36.3(3)
C28A	Ru1A	C32A	60.2(4)	C28E	Ru1E	C32E	61.0(3)
C32A	Ru1A	C30A	35.4(4)	C32E	Ru1E	C30E	36.6(3)
C2A	N1A	C3A	111.3(8)	C2E	N1E	C3E	108.2(8)
C2A	N2A	C5A	128.1(8)	C2E	N2E	C5E	128.7(7)
C4A	N2A	C2A	109.5(8)	C4E	N2E	C2E	108.0(7)
C4A	N2A	C5A	120.5(8)	C4E	N2E	C5E	120.8(8)
C17A	N3A	C18A	125.2(8)	C17E	N3E	C18E	126.3(8)
C17A	N3A	C19A	112.1(9)	C17E	N3E	C19E	112.4(8)
C19A	N3A	C18A	122.6(9)	C19E	N3E	C18E	121.3(8)
C17A	N4A	C21A	111.4(10)	C17E	N4E	C21E	112.4(8)
C17A	N4A	C23A	125.1(10)	C17E	N4E	C23E	125.2(7)
C21A	N4A	C23A	123.4(10)	C21E	N4E	C23E	122.4(8)

Table S21 Bond Angles for 4.

Atom	Atom	Atom	Angle/˚	Atom	Atom	Atom	Angle/˚
O1A	C1A	Ru1A	168.0(8)	O1E	C1E	Ru1E	170.0(7)
N1A	C2A	Ru1A	125.0(6)	N1E	C2E	Ru1E	121.4(6)
N1A	C2A	N2A	105.4(7)	N1E	C2E	N2E	107.5(7)
N2A	C2A	Ru1A	129.6(6)	N2E	C2E	Ru1E	131.1(6)
N1A	C3A	C4A	108.4(8)	C4E	C3E	N1E	110.4(9)
C3A	C4A	N2A	105.4(8)	C3E	C4E	N2E	106.0(9)
C6A	C5A	N2A	121.2(9)	C6E	C5E	N2E	119.5(8)
C6A	C5A	C13A	122.5(9)	C13E	C5E	N2E	117.8(8)
C13A	C5A	N2A	116.3(9)	C13E	C5E	C6E	122.4(8)
C5A	C6A	C7A	121.6(9)	C5E	C6E	C7E	121.6(8)
C10A	C6A	C5A	118.6(10)	C10E	C6E	C5E	117.3(9)
C10A	C6A	C7A	119.8(10)	C10E	C6E	C7E	121.0(8)
C8A	C7A	C6A	109.0(11)	C6E	C7E	C8E	110.5(8)
C9A	C7A	C6A	112.9(11)	C6E	C7E	C9E	114.7(9)
C9A	C7A	C8A	111.2(12)	C9E	C7E	C8E	110.6(8)
C6A	C10A	C11A	120.4(10)	C11E	C10E	C6E	120.8(9)
C12A	C11A	C10A	120.4(10)	C10E	C11E	C12E	120.5(9)
C11A	C12A	C13A	121.3(11)	C13E	C12E	C11E	120.1(9)
C5A	C13A	C12A	116.7(10)	C5E	C13E	C14E	122.3(9)
C5A	C13A	C14A	123.3(9)	C12E	C13E	C5E	118.7(9)
C12A	C13A	C14A	120.0(10)	C12E	C13E	C14E	119.0(9)
C13A	C14A	C15A	110.3(9)	C13E	C14E	C15E	111.7(9)
C13A	C14A	C16A	112.1(9)	C13E	C14E	C16E	111.9(9)
C16A	C14A	C15A	110.5(10)	C16E	C14E	C15E	109.7(9)
N3A	C17A	Ru1A	130.3(7)	N3E	C17E	Ru1E	130.3(7)

Table S21 Bond Angles for 4.

Atom	Atom	Atom	Angle/˚	Atom	Atom	Atom	Angle/˚
N3A	C17A	N4A	103.2(9)	N4E	C17E	Ru1E	127.3(7)
N4A	C17A	Ru1A	126.5(8)	N4E	C17E	N3E	102.3(8)
N3A	C19A	C20A	121.4(10)	N3E	C19E	C20E	124.3(10)
C21A	C19A	N3A	106.5(10)	C21E	C19E	N3E	106.6(8)
C21A	C19A	C20A	132.0(10)	C21E	C19E	C20E	128.8(10)
N4A	C21A	C22A	123.5(12)	N4E	C21E	C22E	122.5(9)
C19A	C21A	N4A	106.7(9)	C19E	C21E	N4E	106.2(8)
C19A	C21A	C22A	129.8(12)	C19E	C21E	C22E	131.3(9)
C25A	C24A	Ru1A	123.9(6)	C25E	C24E	Ru1E	125.2(6)
C26A	C24A	Ru1A	70.7(5)	C26E	C24E	Ru1E	72.3(5)
C26A	C24A	C25A	128.4(9)	C26E	C24E	C25E	126.4(8)
C26A	C24A	C32A	107.6(8)	C26E	C24E	C32E	108.5(8)
C32A	C24A	Ru1A	73.1(5)	C32E	C24E	Ru1E	73.2(5)
C32A	C24A	C25A	124.0(8)	C32E	C24E	C25E	124.8(8)
C24A	C26A	Ru1A	72.4(5)	C24E	C26E	Ru1E	70.9(5)
C24A	C26A	C27A	126.1(10)	C24E	C26E	C27E	127.7(8)
C24A	C26A	C28A	107.3(9)	C24E	C26E	C28E	107.7(7)
C27A	C26A	Ru1A	127.5(7)	C27E	C26E	Ru1E	126.0(6)
C28A	C26A	Ru1A	72.1(5)	C28E	C26E	Ru1E	71.8(5)
C28A	C26A	C27A	126.1(10)	C28E	C26E	C27E	124.4(7)
C26A	C28A	Ru1A	70.9(5)	C26E	C28E	Ru1E	71.3(5)
C26A	C28A	C29A	123.5(11)	C26E	C28E	C29E	123.8(7)
C29A	C28A	Ru1A	129.0(8)	C29E	C28E	Ru1E	127.8(6)
C30A	C28A	Ru1A	74.4(6)	C30E	C28E	Ru1E	72.6(5)
C30A	C28A	C26A	108.2(9)	C30E	C28E	C26E	108.0(7)

Table S21 Bond Angles for 4.

Atom	Atom	Atom	Angle/˚	Atom	Atom	Atom	Angle/˚
C30A	C28A	C29A	127.4(11)	C30E	C28E	C29E	127.7(8)
C28A	C30A	Ru1A	70.1(6)	C28E	C30E	Ru1E	71.1(5)
C28A	C30A	C31A	125.4(11)	C28E	C30E	C31E	125.2(8)
C28A	C30A	C32A	109.7(10)	C28E	C30E	C32E	108.0(8)
C31A	C30A	Ru1A	123.9(7)	C31E	C30E	Ru1E	125.7(6)
C32A	C30A	Ru1A	71.9(5)	C32E	C30E	Ru1E	71.5(5)
C32A	C30A	C31A	124.9(11)	C32E	C30E	C31E	126.7(8)
C24A	C32A	Ru1A	70.2(5)	C24E	C32E	Ru1E	70.2(5)
C24A	C32A	C33A	126.1(10)	C24E	C32E	C30E	107.7(8)
C30A	C32A	Ru1A	72.7(6)	C24E	C32E	C33E	126.7(9)
C30A	C32A	C24A	107.2(8)	C30E	C32E	Ru1E	71.9(5)
C30A	C32A	C33A	126.1(10)	C30E	C32E	C33E	125.2(9)
C33A	C32A	Ru1A	129.5(7)	C33E	C32E	Ru1E	128.4(6)
C1B	Ru1B	C2B	95.2(3)	C1F	Ru1F	C2F	95.0(4)
C1B	Ru1B	C17B	93.2(3)	C1F	Ru1F	C17F	93.1(4)
C1B	Ru1B	C24B	121.3(3)	C1F	Ru1F	C24F	119.9(4)
C1B	Ru1B	C26B	154.5(3)	C1F	Ru1F	C26F	153.8(4)
C1B	Ru1B	C28B	132.7(3)	C1F	Ru1F	C28F	134.5(3)
C1B	Ru1B	C30B	99.1(3)	C1F	Ru1F	C30F	100.2(3)
C1B	Ru1B	C32B	93.3(3)	C1F	Ru1F	C32F	93.0(3)
C2B	Ru1B	C24B	93.4(3)	C2F	Ru1F	C24F	92.7(3)
C2B	Ru1B	C26B	98.5(3)	C2F	Ru1F	C26F	97.1(3)
C2B	Ru1B	C28B	131.8(3)	C2F	Ru1F	C28F	130.1(4)
C2B	Ru1B	C30B	155.2(3)	C2F	Ru1F	C30F	154.1(3)
C2B	Ru1B	C32B	122.2(3)	C2F	Ru1F	C32F	121.4(3)

Table S21 Bond Angles for 4.

Atom	Atom	Atom	Angle/˚	Atom	Atom	Atom	Angle/˚
C17B	Ru1B	C2B	90.7(3)	C17F	Ru1F	C2F	91.6(4)
C17B	Ru1B	C24B	144.6(3)	C17F	Ru1F	C24F	146.2(3)
C17B	Ru1B	C26B	107.9(3)	C17F	Ru1F	C26F	109.6(3)
C17B	Ru1B	C28B	91.0(3)	C17F	Ru1F	C28F	91.3(3)
C17B	Ru1B	C30B	108.4(3)	C17F	Ru1F	C30F	108.3(3)
C17B	Ru1B	C32B	145.6(3)	C17F	Ru1F	C32F	145.8(3)
C24B	Ru1B	C26B	36.7(3)	C24F	Ru1F	C26F	36.6(3)
C24B	Ru1B	C28B	60.7(3)	C24F	Ru1F	C28F	61.0(3)
C24B	Ru1B	C30B	61.9(3)	C24F	Ru1F	C30F	61.6(3)
C24B	Ru1B	C32B	36.9(3)	C24F	Ru1F	C32F	36.3(3)
C26B	Ru1B	C28B	36.3(3)	C28F	Ru1F	C26F	36.4(3)
C30B	Ru1B	C26B	61.2(3)	C30F	Ru1F	C26F	61.1(3)
C30B	Ru1B	C28B	36.1(3)	C30F	Ru1F	C28F	36.5(3)
C32B	Ru1B	C26B	61.2(3)	C32F	Ru1F	C26F	60.9(3)
C32B	Ru1B	C28B	60.5(3)	C32F	Ru1F	C28F	61.3(3)
C32B	Ru1B	C30B	37.1(3)	C32F	Ru1F	C30F	37.5(3)
C2B	N1B	C3B	107.1(8)	C2F	N1F	C3F	108.6(8)
C2B	N2B	C5B	128.6(7)	C2F	N2F	C5F	129.6(7)
C4B	N2B	C2B	108.0(8)	C4F	N2F	C2F	107.1(8)
C4B	N2B	C5B	121.1(8)	C4F	N2F	C5F	121.1(7)
C17B	N3B	C18B	126.9(8)	C17F	N3F	C18F	126.0(8)
C17B	N3B	C19B	111.5(8)	C17F	N3F	C19F	111.5(8)
C19B	N3B	C18B	121.6(8)	C19F	N3F	C18F	122.5(8)
C17B	N4B	C21B	112.7(8)	C17F	N4F	C21F	111.9(8)
C17B	N4B	C23B	125.2(7)	C17F	N4F	C23F	127.0(8)

Table S21 Bond Angles for 4.

Atom	Atom	Atom	Angle/˚	Atom	Atom	Atom	Angle/˚
C21B	N4B	C23B	122.1(7)	C21F	N4F	C23F	121.1(8)
O1B	C1B	Ru1B	169.9(7)	O1F	C1F	Ru1F	169.1(7)
N1B	C2B	Ru1B	120.9(7)	N1F	C2F	Ru1F	123.1(7)
N1B	C2B	N2B	107.9(7)	N1F	C2F	N2F	107.2(7)
N2B	C2B	Ru1B	131.1(6)	N2F	C2F	Ru1F	129.5(6)
C4B	C3B	N1B	110.9(8)	C4F	C3F	N1F	110.7(8)
C3B	C4B	N2B	106.1(8)	C3F	C4F	N2F	106.1(8)
C6B	C5B	N2B	119.5(8)	C6F	C5F	N2F	118.5(9)
C13B	C5B	N2B	118.6(8)	C13F	C5F	N2F	118.9(8)
C13B	C5B	C6B	121.4(9)	C13F	C5F	C6F	122.2(8)
C5B	C6B	C7B	120.3(8)	C5F	C6F	C7F	121.6(8)
C10B	C6B	C5B	117.7(9)	C10F	C6F	C5F	117.7(9)
C10B	C6B	C7B	121.9(9)	C10F	C6F	C7F	120.7(9)
C6B	C7B	C8B	112.6(8)	C6F	C7F	C8F	111.2(9)
C6B	C7B	C9B	110.4(8)	C9F	C7F	C6F	112.2(8)
C9B	C7B	C8B	109.8(8)	C9F	C7F	C8F	109.6(9)
C6B	C10B	C11B	121.7(9)	C6F	C10F	C11F	121.6(10)
C12B	C11B	C10B	119.7(9)	C12F	C11F	C10F	118.6(9)
C11B	C12B	C13B	121.2(9)	C11F	C12F	C13F	122.8(10)
C5B	C13B	C14B	122.2(9)	C5F	C13F	C12F	117.1(9)
C12B	C13B	C5B	118.2(9)	C5F	C13F	C14F	120.4(8)
C12B	C13B	C14B	119.6(9)	C12F	C13F	C14F	122.5(9)
C13B	C14B	C15B	111.5(9)	C13F	C14F	C16F	112.6(8)
C13B	C14B	C16B	111.4(8)	C15F	C14F	C13F	110.1(9)
C16B	C14B	C15B	110.0(8)	C15F	C14F	C16F	109.9(8)

Table S21 Bond Angles for 4.

Atom	Atom	Atom	Angle/˚	Atom	Atom	Atom	Angle/˚
N3B	C17B	Ru1B	129.3(7)	N3F	C17F	Ru1F	127.2(7)
N4B	C17B	Ru1B	127.5(7)	N3F	C17F	N4F	103.8(8)
N4B	C17B	N3B	103.1(7)	N4F	C17F	Ru1F	128.9(7)
N3B	C19B	C20B	122.0(9)	N3F	C19F	C20F	122.6(9)
C21B	C19B	N3B	106.3(8)	C21F	C19F	N3F	107.0(8)
C21B	C19B	C20B	131.5(9)	C21F	C19F	C20F	130.5(9)
N4B	C21B	C22B	123.1(9)	N4F	C21F	C22F	122.8(9)
C19B	C21B	N4B	106.4(8)	C19F	C21F	N4F	105.8(8)
C19B	C21B	C22B	130.5(9)	C19F	C21F	C22F	131.4(9)
C25B	C24B	Ru1B	125.9(6)	C25F	C24F	Ru1F	124.9(6)
C26B	C24B	Ru1B	73.3(5)	C26F	C24F	Ru1F	73.5(5)
C26B	C24B	C25B	125.2(8)	C26F	C24F	C25F	124.2(8)
C32B	C24B	Ru1B	72.0(5)	C32F	C24F	Ru1F	72.5(5)
C32B	C24B	C25B	126.2(8)	C32F	C24F	C25F	126.2(8)
C32B	C24B	C26B	108.3(7)	C32F	C24F	C26F	109.4(8)
C24B	C26B	Ru1B	70.0(5)	C24F	C26F	Ru1F	69.9(5)
C24B	C26B	C27B	125.8(9)	C24F	C26F	C27F	125.4(9)
C24B	C26B	C28B	107.4(8)	C24F	C26F	C28F	107.5(8)
C27B	C26B	Ru1B	129.5(6)	C27F	C26F	Ru1F	129.9(7)
C28B	C26B	Ru1B	72.9(5)	C28F	C26F	Ru1F	71.8(5)
C28B	C26B	C27B	126.1(9)	C28F	C26F	C27F	126.6(9)
C26B	C28B	Ru1B	70.8(5)	C26F	C28F	Ru1F	71.9(5)
C26B	C28B	C29B	126.0(8)	C26F	C28F	C29F	126.5(9)
C29B	C28B	Ru1B	124.5(6)	C29F	C28F	Ru1F	125.0(6)
C30B	C28B	Ru1B	70.2(5)	C30F	C28F	Ru1F	70.9(5)

Table S21 Bond Angles for 4.

Atom	Atom	Atom	Angle/˚	Atom	Atom	Atom	Angle/˚
C30B	C28B	C26B	108.9(8)	C30F	C28F	C26F	108.4(8)
C30B	C28B	C29B	125.1(8)	C30F	C28F	C29F	125.0(8)
C28B	C30B	Ru1B	73.8(5)	C28F	C30F	Ru1F	72.6(5)
C28B	C30B	C31B	126.7(8)	C28F	C30F	C31F	127.7(8)
C28B	C30B	C32B	107.3(7)	C28F	C30F	C32F	107.1(8)
C31B	C30B	Ru1B	128.4(6)	C31F	C30F	Ru1F	128.2(6)
C32B	C30B	Ru1B	71.0(4)	C32F	C30F	Ru1F	71.0(5)
C32B	C30B	C31B	125.3(8)	C32F	C30F	C31F	124.6(8)
C24B	C32B	Ru1B	71.1(5)	C24F	C32F	Ru1F	71.2(5)
C24B	C32B	C30B	108.0(7)	C24F	C32F	C30F	107.6(8)
C24B	C32B	C33B	128.3(8)	C24F	C32F	C33F	129.2(8)
C30B	C32B	Ru1B	71.9(5)	C30F	C32F	Ru1F	71.5(5)
C30B	C32B	C33B	123.5(7)	C30F	C32F	C33F	123.1(8)
C33B	C32B	Ru1B	126.2(6)	C33F	C32F	Ru1F	125.8(6)
C1C	Ru1C	C2C	95.2(4)	C1G	Ru1G	C2G	94.0(4)
C1C	Ru1C	C17C	95.7(4)	C1G	Ru1G	C17G	94.1(5)
C1C	Ru1C	C24C	152.4(4)	C1G	Ru1G	C24G	101.1(4)
C1C	Ru1C	C26C	130.5(4)	C1G	Ru1G	C26G	91.5(4)
C1C	Ru1C	C28C	96.5(4)	C1G	Ru1G	C28G	117.2(4)
C1C	Ru1C	C30C	91.1(4)	C1G	Ru1G	C30G	152.1(4)
C1C	Ru1C	C32C	120.1(4)	C1G	Ru1G	C32G	136.2(4)
C2C	Ru1C	C17C	90.1(3)	C2G	Ru1G	C17G	90.5(4)
C2C	Ru1C	C24C	100.4(3)	C2G	Ru1G	C24G	155.8(4)
C2C	Ru1C	C26C	133.9(4)	C2G	Ru1G	C26G	125.0(4)
C2C	Ru1C	C28C	155.2(4)	C2G	Ru1G	C28G	94.4(3)

Table S21 Bond Angles for 4.

Atom	Atom	Atom	Angle/˚	Atom	Atom	Atom	Angle/˚
C2C	Ru1C	C30C	122.4(3)	C2G	Ru1G	C30G	97.1(4)
C2C	Ru1C	C32C	94.0(3)	C2G	Ru1G	C32G	129.3(4)
C17C	Ru1C	C24C	106.8(4)	C17G	Ru1G	C24G	107.1(4)
C17C	Ru1C	C26C	90.5(4)	C17G	Ru1G	C26G	143.5(4)
C17C	Ru1C	C28C	110.4(4)	C17G	Ru1G	C28G	147.8(4)
C17C	Ru1C	C30C	146.1(4)	C17G	Ru1G	C30G	111.2(4)
C17C	Ru1C	C32C	143.4(4)	C17G	Ru1G	C32G	91.7(4)
C24C	Ru1C	C32C	36.7(3)	C24G	Ru1G	C26G	36.7(4)
C26C	Ru1C	C24C	36.4(4)	C24G	Ru1G	C28G	61.8(3)
C26C	Ru1C	C32C	61.1(4)	C24G	Ru1G	C30G	61.3(4)
C28C	Ru1C	C24C	61.1(4)	C24G	Ru1G	C32G	36.5(4)
C28C	Ru1C	C26C	36.8(4)	C26G	Ru1G	C30G	61.3(3)
C28C	Ru1C	C32C	61.2(4)	C26G	Ru1G	C32G	60.7(4)
C30C	Ru1C	C24C	61.3(4)	C28G	Ru1G	C26G	37.3(3)
C30C	Ru1C	C26C	60.8(4)	C28G	Ru1G	C30G	36.6(3)
C30C	Ru1C	C28C	35.7(4)	C28G	Ru1G	C32G	60.9(4)
C30C	Ru1C	C32C	37.4(3)	C30G	Ru1G	C32G	36.4(4)
C2C	N1C	C3C	107.8(7)	C2G	N1G	C3G	114.4(9)
C2C	N2C	C5C	130.0(8)	C2G	N2G	C5G	128.7(9)
C4C	N2C	C2C	106.7(7)	C4G	N2G	C2G	108.8(9)
C4C	N2C	C5C	121.6(8)	C4G	N2G	C5G	120.9(9)
C17C	N3C	C18C	126.2(8)	C17G	N3G	C18G	125.5(9)
C17C	N3C	C19C	111.2(9)	C17G	N3G	C19G	110.9(9)
C19C	N3C	C18C	122.6(9)	C19G	N3G	C18G	123.5(10)
C17C	N4C	C23C	125.7(9)	C17G	N4G	C21G	111.4(9)

Table S21 Bond Angles for 4.

Atom	Atom	Atom	Angle/˚	Atom	Atom	Atom	Angle/˚
C21C	N4C	C17C	111.0(9)	C17G	N4G	C23G	126.6(10)
C21C	N4C	C23C	123.2(9)	C21G	N4G	C23G	122.1(9)
O1C	C1C	Ru1C	168.7(9)	O1G	C1G	Ru1G	163.2(10)
N1C	C2C	Ru1C	122.9(6)	N1G	C2G	Ru1G	126.9(7)
N1C	C2C	N2C	107.7(7)	N1G	C2G	N2G	104.2(8)
N2C	C2C	Ru1C	129.3(6)	N2G	C2G	Ru1G	128.9(7)
C4C	C3C	N1C	111.3(8)	N1G	C3G	C4G	107.8(10)
C3C	C4C	N2C	106.5(8)	C3G	C4G	N2G	104.7(10)
C6C	C5C	N2C	116.3(8)	C6G	C5G	N2G	113.1(11)
C13C	C5C	N2C	119.4(9)	C13G	C5G	N2G	122.2(11)
C13C	C5C	C6C	124.2(9)	C13G	C5G	C6G	124.1(11)
C5C	C6C	C7C	124.0(8)	C5G	C6G	C7G	125.9(11)
C10C	C6C	C5C	116.6(9)	C5G	C6G	C10G	116.7(12)
C10C	C6C	C7C	119.5(9)	C10G	C6G	C7G	117.3(13)
C6C	C7C	C9C	110.5(9)	C6G	C7G	C8G	109.1(12)
C8C	C7C	C6C	110.1(9)	C9G	C7G	C6G	113.3(13)
C8C	C7C	C9C	112.1(8)	C9G	C7G	C8G	111.6(12)
C11C	C10C	C6C	120.5(10)	C11G	C10G	C6G	119.9(14)
C12C	C11C	C10C	120.1(10)	C10G	C11G	C12G	120.1(14)
C11C	C12C	C13C	121.9(9)	C13G	C12G	C11G	120.5(14)
C5C	C13C	C12C	116.7(10)	C5G	C13G	C12G	118.7(14)
C5C	C13C	C14C	122.1(9)	C5G	C13G	C14G	122.2(12)
C12C	C13C	C14C	121.1(9)	C12G	C13G	C14G	119.1(12)
C13C	C14C	C15C	112.6(9)	C15G	C14G	C13G	107.2(12)
C16C	C14C	C13C	110.5(9)	C16G	C14G	C13G	111.0(13)

Table S21 Bond Angles for 4.

Atom	Atom	Atom	Angle/˚	Atom	Atom	Atom	Angle/˚
C16C	C14C	C15C	109.6(9)	C16G	C14G	C15G	109.2(13)
N3C	C17C	Ru1C	131.3(7)	N3G	C17G	Ru1G	129.2(8)
N3C	C17C	N4C	104.2(8)	N3G	C17G	N4G	104.7(10)
N4C	C17C	Ru1C	124.5(8)	N4G	C17G	Ru1G	126.1(8)
N3C	C19C	C20C	122.1(10)	N3G	C19G	C20G	121.7(10)
C21C	C19C	N3C	106.2(9)	C21G	C19G	N3G	107.0(10)
C21C	C19C	C20C	131.6(10)	C21G	C19G	C20G	131.3(10)
N4C	C21C	C22C	121.6(11)	N4G	C21G	C22G	123.8(11)
C19C	C21C	N4C	107.5(9)	C19G	C21G	N4G	105.9(9)
C19C	C21C	C22C	130.9(11)	C19G	C21G	C22G	130.2(12)
C25C	C24C	Ru1C	127.5(7)	C25G	C24G	Ru1G	128.7(8)
C26C	C24C	Ru1C	71.3(6)	C26G	C24G	Ru1G	72.5(5)
C26C	C24C	C25C	126.5(10)	C26G	C24G	C25G	124.9(11)
C26C	C24C	C32C	108.5(10)	C32G	C24G	Ru1G	73.3(6)
C32C	C24C	Ru1C	72.6(6)	C32G	C24G	C25G	125.9(12)
C32C	C24C	C25C	124.6(10)	C32G	C24G	C26G	108.2(9)
C24C	C26C	Ru1C	72.3(6)	C24G	C26G	Ru1G	70.8(5)
C24C	C26C	C27C	128.2(12)	C24G	C26G	C27G	125.7(10)
C24C	C26C	C28C	108.1(10)	C24G	C26G	C28G	107.7(9)
C27C	C26C	Ru1C	122.9(7)	C27G	C26G	Ru1G	127.2(6)
C28C	C26C	Ru1C	71.5(6)	C28G	C26G	Ru1G	71.2(5)
C28C	C26C	C27C	123.7(12)	C28G	C26G	C27G	126.4(10)
C26C	C28C	Ru1C	71.7(6)	C26G	C28G	Ru1G	71.5(5)
C26C	C28C	C29C	127.7(12)	C26G	C28G	C29G	126.0(9)
C29C	C28C	Ru1C	127.2(8)	C29G	C28G	Ru1G	126.8(8)

Table S21 Bond Angles for 4.

Atom	Atom	Atom	Angle/˚	Atom	Atom	Atom	Angle/˚
C30C	C28C	Ru1C	71.5(6)	C30G	C28G	Ru1G	72.5(5)
C30C	C28C	C26C	108.3(10)	C30G	C28G	C26G	107.5(8)
C30C	C28C	C29C	123.7(11)	C30G	C28G	C29G	126.1(9)
C28C	C30C	Ru1C	72.8(6)	C28G	C30G	Ru1G	70.9(5)
C28C	C30C	C31C	124.7(10)	C28G	C30G	C31G	124.6(10)
C28C	C30C	C32C	109.6(9)	C31G	C30G	Ru1G	129.5(7)
C31C	C30C	Ru1C	127.8(7)	C32G	C30G	Ru1G	71.9(6)
C32C	C30C	Ru1C	73.4(6)	C32G	C30G	C28G	107.9(9)
C32C	C30C	C31C	125.0(10)	C32G	C30G	C31G	127.0(11)
C24C	C32C	Ru1C	70.7(6)	C24G	C32G	Ru1G	70.2(6)
C24C	C32C	C30C	105.4(9)	C24G	C32G	C30G	108.6(10)
C24C	C32C	C33C	127.6(10)	C24G	C32G	C33G	126.3(12)
C30C	C32C	Ru1C	69.2(6)	C30G	C32G	Ru1G	71.8(6)
C30C	C32C	C33C	126.9(9)	C30G	C32G	C33G	125.1(12)
C33C	C32C	Ru1C	126.1(7)	C33G	C32G	Ru1G	123.4(8)
C1D	Ru1D	C2D	95.3(3)	C1H	Ru1H	C2H	94.9(4)
C1D	Ru1D	C17D	92.5(4)	C1H	Ru1H	C17H	94.3(5)
C1D	Ru1D	C24D	100.1(3)	C1H	Ru1H	C24H	97.8(5)
C1D	Ru1D	C26D	134.3(3)	C1H	Ru1H	C26H	131.4(4)
C1D	Ru1D	C28D	154.0(4)	C1H	Ru1H	C28H	153.8(4)
C1D	Ru1D	C30D	119.3(4)	C1H	Ru1H	C30H	119.8(4)
C1D	Ru1D	C32D	92.6(3)	C1H	Ru1H	C32H	92.3(4)
C2D	Ru1D	C24D	154.3(3)	C2H	Ru1H	C24H	156.1(4)
C2D	Ru1D	C26D	130.1(3)	C2H	Ru1H	C26H	133.3(4)
C2D	Ru1D	C28D	97.3(3)	C2H	Ru1H	C28H	99.1(4)

Table S21 Bond Angles for 4.

Atom	Atom	Atom	Angle/˚	Atom	Atom	Atom	Angle/˚
C2D	Ru1D	C30D	92.9(3)	C2H	Ru1H	C30H	95.1(3)
C2D	Ru1D	C32D	122.2(3)	C2H	Ru1H	C32H	123.3(4)
C17D	Ru1D	C2D	91.5(3)	C17H	Ru1H	C2H	89.5(4)
C17D	Ru1D	C24D	108.1(3)	C17H	Ru1H	C24H	109.5(4)
C17D	Ru1D	C26D	91.5(3)	C17H	Ru1H	C26H	92.0(4)
C17D	Ru1D	C28D	109.8(4)	C17H	Ru1H	C28H	107.8(4)
C17D	Ru1D	C30D	147.2(3)	C17H	Ru1H	C30H	144.9(4)
C17D	Ru1D	C32D	145.3(4)	C17H	Ru1H	C32H	145.8(4)
C24D	Ru1D	C26D	36.3(3)	C24H	Ru1H	C28H	62.0(4)
C24D	Ru1D	C28D	61.0(3)	C26H	Ru1H	C24H	35.8(4)
C28D	Ru1D	C26D	36.2(3)	C26H	Ru1H	C28H	36.9(4)
C30D	Ru1D	C24D	61.6(3)	C30H	Ru1H	C24H	61.0(4)
C30D	Ru1D	C26D	61.4(3)	C30H	Ru1H	C26H	60.1(4)
C30D	Ru1D	C28D	37.4(3)	C30H	Ru1H	C28H	37.1(4)
C32D	Ru1D	C24D	37.3(3)	C32H	Ru1H	C24H	36.3(4)
C32D	Ru1D	C26D	61.3(3)	C32H	Ru1H	C26H	59.5(4)
C32D	Ru1D	C28D	61.5(3)	C32H	Ru1H	C28H	61.5(4)
C32D	Ru1D	C30D	36.6(3)	C32H	Ru1H	C30H	36.2(4)
C2D	N1D	C3D	107.5(7)	C2H	N1H	C3H	109.6(8)
C2D	N2D	C5D	129.6(7)	C2H	N2H	C5H	129.6(8)
C4D	N2D	C2D	108.3(7)	C4H	N2H	C2H	108.6(7)
C4D	N2D	C5D	120.7(8)	C4H	N2H	C5H	120.3(8)
C17D	N3D	C18D	125.4(8)	C17H	N3H	C18H	125.5(9)
C17D	N3D	C19D	112.1(7)	C19H	N3H	C17H	111.9(9)
C19D	N3D	C18D	122.5(8)	C19H	N3H	C18H	122.6(9)

Table S21 Bond Angles for 4.

Atom	Atom	Atom	Angle/˚	Atom	Atom	Atom	Angle/˚
C17D	N4D	C21D	110.8(8)	C17H	N4H	C21H	110.9(9)
C17D	N4D	C23D	126.7(8)	C17H	N4H	C23H	126.3(9)
C21D	N4D	C23D	122.5(8)	C21H	N4H	C23H	122.7(9)
O1D	C1D	Ru1D	169.5(7)	O1H	C1H	Ru1H	168.4(9)
N1D	C2D	Ru1D	121.9(6)	N1H	C2H	Ru1H	123.7(6)
N1D	C2D	N2D	107.2(7)	N1H	C2H	N2H	106.3(7)
N2D	C2D	Ru1D	130.9(6)	N2H	C2H	Ru1H	130.0(6)
C4D	C3D	N1D	110.2(8)	C4H	C3H	N1H	110.1(9)
C3D	C4D	N2D	106.8(8)	C3H	C4H	N2H	105.3(8)
C6D	C5D	N2D	117.7(8)	C6H	C5H	N2H	117.0(9)
C13D	C5D	N2D	118.1(8)	C6H	C5H	C13H	122.9(10)
C13D	C5D	C6D	124.1(8)	C13H	C5H	N2H	119.7(9)
C5D	C6D	C7D	122.9(8)	C5H	C6H	C7H	123.6(9)
C10D	C6D	C5D	117.3(9)	C5H	C6H	C10H	118.0(10)
C10D	C6D	C7D	119.8(9)	C10H	C6H	C7H	118.4(10)
C6D	C7D	C8D	111.6(9)	C6H	C7H	C8H	112.6(10)
C9D	C7D	C6D	113.0(9)	C6H	C7H	C9H	111.4(10)
C9D	C7D	C8D	110.4(9)	C8H	C7H	C9H	110.5(9)
C11D	C10D	C6D	121.2(10)	C11H	C10H	C6H	120.6(11)
C10D	C11D	C12D	120.9(9)	C10H	C11H	C12H	120.2(10)
C11D	C12D	C13D	120.6(10)	C11H	C12H	C13H	121.6(11)
C5D	C13D	C12D	115.8(9)	C5H	C13H	C12H	116.5(11)
C5D	C13D	C14D	123.6(8)	C5H	C13H	C14H	121.7(10)
C12D	C13D	C14D	120.5(9)	C12H	C13H	C14H	121.7(10)
C13D	C14D	C15D	113.4(9)	C13H	C14H	C15H	112.7(11)

Table S21 Bond Angles for 4.

Atom	Atom	Atom	Angle/˚	Atom	Atom	Atom	Angle/˚
C13D	C14D	C16D	109.5(9)	C13H	C14H	C16H	109.9(10)
C15D	C14D	C16D	110.4(9)	C16H	C14H	C15H	109.9(11)
N3D	C17D	Ru1D	128.3(7)	N3H	C17H	Ru1H	125.7(8)
N3D	C17D	N4D	103.6(8)	N4H	C17H	Ru1H	131.0(8)
N4D	C17D	Ru1D	128.0(7)	N4H	C17H	N3H	103.2(9)
N3D	C19D	C20D	122.1(8)	N3H	C19H	C20H	122.4(11)
C21D	C19D	N3D	107.2(8)	C21H	C19H	N3H	107.5(9)
C21D	C19D	C20D	130.5(9)	C21H	C19H	C20H	130.0(11)
N4D	C21D	C22D	121.5(9)	N4H	C21H	C22H	120.8(10)
C19D	C21D	N4D	106.2(8)	C19H	C21H	N4H	106.3(10)
C19D	C21D	C22D	132.2(9)	C19H	C21H	C22H	132.8(10)
C25D	C24D	Ru1D	129.0(6)	C25H	C24H	Ru1H	129.4(9)
C26D	C24D	Ru1D	72.9(5)	C26H	C24H	Ru1H	71.7(6)
C26D	C24D	C25D	126.7(8)	C26H	C24H	C25H	127.6(13)
C26D	C24D	C32D	107.9(8)	C26H	C24H	C32H	105.2(10)
C32D	C24D	Ru1D	70.9(5)	C32H	C24H	Ru1H	69.4(6)
C32D	C24D	C25D	124.7(8)	C32H	C24H	C25H	126.7(12)
C24D	C26D	Ru1D	70.7(5)	C24H	C26H	Ru1H	72.5(7)
C24D	C26D	C27D	124.8(8)	C24H	C26H	C27H	124.8(12)
C24D	C26D	C28D	108.6(8)	C24H	C26H	C28H	111.9(10)
C27D	C26D	Ru1D	125.4(6)	C27H	C26H	Ru1H	123.7(7)
C28D	C26D	Ru1D	71.7(5)	C28H	C26H	Ru1H	72.2(6)
C28D	C26D	C27D	126.5(8)	C28H	C26H	C27H	123.3(12)
C26D	C28D	Ru1D	72.2(5)	C26H	C28H	Ru1H	70.9(6)
C26D	C28D	C29D	127.2(9)	C26H	C28H	C29H	129.6(11)

Table S21 Bond Angles for 4.

Atom	Atom	Atom	Angle/˚	Atom	Atom	Atom	Angle/˚
C26D	C28D	C30D	107.6(8)	C29H	C28H	Ru1H	129.6(8)
C29D	C28D	Ru1D	129.7(7)	C30H	C28H	Ru1H	69.1(6)
C30D	C28D	Ru1D	70.1(5)	C30H	C28H	C26H	102.9(10)
C30D	C28D	C29D	124.7(9)	C30H	C28H	C29H	126.9(11)
C28D	C30D	Ru1D	72.5(5)	C28H	C30H	Ru1H	73.8(6)
C28D	C30D	C31D	125.8(8)	C28H	C30H	C31H	126.2(10)
C31D	C30D	Ru1D	125.2(6)	C31H	C30H	Ru1H	126.9(7)
C32D	C30D	Ru1D	71.6(5)	C32H	C30H	Ru1H	71.6(5)
C32D	C30D	C28D	107.7(8)	C32H	C30H	C28H	109.6(9)
C32D	C30D	C31D	126.4(8)	C32H	C30H	C31H	123.7(10)
C24D	C32D	Ru1D	71.8(5)	C24H	C32H	Ru1H	74.3(6)
C24D	C32D	C33D	123.3(8)	C24H	C32H	C33H	122.8(10)
C30D	C32D	Ru1D	71.8(5)	C30H	C32H	Ru1H	72.2(5)
C30D	C32D	C24D	108.2(8)	C30H	C32H	C24H	110.4(10)
C30D	C32D	C33D	128.3(8)	C30H	C32H	C33H	126.3(11)
C33D	C32D	Ru1D	126.4(6)	C33H	C32H	Ru1H	126.9(8)

Table S22 Torsion Angles for 4.

A	B	C	D	Angle/˚	A	B	C	D	Angle/˚
Ru1A	C24A	C26A	C27A	124.0(10)	C32D	C24D	C26D	Ru1D	62.7(6)
Ru1A	C24A	C26A	C28A	-64.1(6)	C32D	C24D	C26D	C27D	-177.0(8)
Ru1A	C24A	C32A	C30A	63.7(7)	C32D	C24D	C26D	C28D	0.7(10)
Ru1A	C24A	C32A	C33A	-125.1(9)	Ru1E	C24E	C26E	C27E	121.3(8)
Ru1A	C26A	C28A	C29A	124.7(10)	Ru1E	C24E	C26E	C28E	-62.8(5)
Ru1A	C26A	C28A	C30A	-65.6(7)	Ru1E	C24E	C32E	C30E	62.5(6)

Table S22 Torsion Angles for 4.

A	B	C	D	Angle/°	A	B	C	D	Angle/°
Ru1A	C28A	C30A	C31A	118.0(10)	Ru1E	C24E	C32E	C33E	-123.7(9)
Ru1A	C28A	C30A	C32A	-61.3(7)	Ru1E	C26E	C28E	C29E	123.5(8)
Ru1A	C30A	C32A	C24A	-62.1(6)	Ru1E	C26E	C28E	C30E	-63.8(6)
Ru1A	C30A	C32A	C33A	126.7(10)	Ru1E	C28E	C30E	C31E	120.8(9)
N1A	C3A	C4A	N2A	0.3(11)	Ru1E	C28E	C30E	C32E	-62.3(6)
N2A	C5A	C6A	C7A	-2.7(15)	Ru1E	C30E	C32E	C24E	-61.4(6)
N2A	C5A	C6A	C10A	176.8(10)	Ru1E	C30E	C32E	C33E	124.7(9)
N2A	C5A	C13A	C12A	-176.5(9)	N1E	C3E	C4E	N2E	1.0(13)
N2A	C5A	C13A	C14A	2.0(14)	N2E	C5E	C6E	C7E	-4.3(13)
N3A	C19A	C21A	N4A	-0.8(13)	N2E	C5E	C6E	C10E	173.1(8)
N3A	C19A	C21A	C22A	180.0(13)	N2E	C5E	C13E	C12E	-173.7(8)
C2A	Ru1A	C1A	O1A	153(5)	N2E	C5E	C13E	C14E	7.0(13)
C2A	N1A	C3A	C4A	-0.2(11)	N3E	C19E	C21E	N4E	1.6(10)
C2A	N2A	C4A	C3A	-0.3(11)	N3E	C19E	C21E	C22E	-178.0(10)
C2A	N2A	C5A	C6A	98.5(12)	C2E	N1E	C3E	C4E	0.2(13)
C2A	N2A	C5A	C13A	-84.6(13)	C2E	N2E	C4E	C3E	-1.8(11)
C3A	N1A	C2A	Ru1A	-179.8(6)	C2E	N2E	C5E	C6E	100.8(11)
C3A	N1A	C2A	N2A	0.0(10)	C2E	N2E	C5E	C13E	-85.4(11)
C4A	N2A	C2A	Ru1A	-180.0(7)	C3E	N1E	C2E	Ru1E	-179.4(7)
C4A	N2A	C2A	N1A	0.2(10)	C3E	N1E	C2E	N2E	-1.4(11)
C4A	N2A	C5A	C6A	-98.8(12)	C4E	N2E	C2E	Ru1E	179.8(7)
C4A	N2A	C5A	C13A	78.0(12)	C4E	N2E	C2E	N1E	2.0(11)
C5A	N2A	C2A	Ru1A	-15.8(14)	C4E	N2E	C5E	C6E	-99.4(10)
C5A	N2A	C2A	N1A	164.4(9)	C4E	N2E	C5E	C13E	74.4(11)
C5A	N2A	C4A	C3A	-165.9(9)	C5E	N2E	C2E	Ru1E	-18.4(14)

Table S22 Torsion Angles for 4.

A	B	C	D	Angle/˚	A	B	C	D	Angle/˚
C5A	C6A	C7A	C8A	81.7(13)	C5E	N2E	C2E	N1E	163.8(9)
C5A	C6A	C7A	C9A	-154.1(11)	C5E	N2E	C4E	C3E	-165.4(9)
C5A	C6A	C10A	C11A	-0.9(17)	C5E	C6E	C7E	C8E	86.9(10)
C5A	C13A	C14A	C15A	-108.5(11)	C5E	C6E	C7E	C9E	-147.4(9)
C5A	C13A	C14A	C16A	127.9(11)	C5E	C6E	C10E	C11E	-0.7(14)
C6A	C5A	C13A	C12A	0.3(15)	C5E	C13E	C14E	C15E	-121.3(10)
C6A	C5A	C13A	C14A	178.8(10)	C5E	C13E	C14E	C16E	115.2(10)
C6A	C10A	C11A	C12A	1.0(18)	C6E	C5E	C13E	C12E	-0.1(13)
C7A	C6A	C10A	C11A	178.7(11)	C6E	C5E	C13E	C14E	-179.4(8)
C10A	C6A	C7A	C8A	-97.8(14)	C6E	C10E	C11E	C12E	2.2(15)
C10A	C6A	C7A	C9A	26.3(16)	C7E	C6E	C10E	C11E	176.7(9)
C10A	C11A	C12A	C13A	-0.5(18)	C10E	C6E	C7E	C8E	-90.4(11)
C11A	C12A	C13A	C5A	-0.1(16)	C10E	C6E	C7E	C9E	35.3(12)
C11A	C12A	C13A	C14A	-178.7(11)	C10E	C11E	C12E	C13E	-2.7(15)
C12A	C13A	C14A	C15A	70.0(13)	C11E	C12E	C13E	C5E	1.6(14)
C12A	C13A	C14A	C16A	-53.7(14)	C11E	C12E	C13E	C14E	-179.1(9)
C13A	C5A	C6A	C7A	-179.3(10)	C12E	C13E	C14E	C15E	59.4(12)
C13A	C5A	C6A	C10A	0.2(16)	C12E	C13E	C14E	C16E	-64.1(12)
C17A	Ru1A	C1A	O1A	-117(5)	C13E	C5E	C6E	C7E	-177.8(8)
C17A	N3A	C19A	C20A	-178.8(10)	C13E	C5E	C6E	C10E	-0.4(13)
C17A	N3A	C19A	C21A	1.5(12)	C17E	N3E	C19E	C20E	-175.3(9)
C17A	N4A	C21A	C19A	-0.1(14)	C17E	N3E	C19E	C21E	-1.1(10)
C17A	N4A	C21A	C22A	179.2(12)	C17E	N4E	C21E	C19E	-1.7(10)
C18A	N3A	C17A	Ru1A	-6.4(15)	C17E	N4E	C21E	C22E	178.0(9)
C18A	N3A	C17A	N4A	174.5(10)	C18E	N3E	C17E	Ru1E	2.5(14)

Table S22 Torsion Angles for 4.

A	B	C	D	Angle/˚	A	B	C	D	Angle/˚
C18A	N3A	C19A	C20A	5.1(15)	C18E	N3E	C17E	N4E	179.8(8)
C18A	N3A	C19A	C21A	-174.6(10)	C18E	N3E	C19E	C20E	5.0(14)
C19A	N3A	C17A	Ru1A	177.7(7)	C18E	N3E	C19E	C21E	179.2(8)
C19A	N3A	C17A	N4A	-1.5(11)	C19E	N3E	C17E	Ru1E	-177.2(7)
C20A	C19A	C21A	N4A	179.6(12)	C19E	N3E	C17E	N4E	0.1(10)
C20A	C19A	C21A	C22A	0(2)	C20E	C19E	C21E	N4E	175.5(10)
C21A	N4A	C17A	Ru1A	-178.2(8)	C20E	C19E	C21E	C22E	-4.1(18)
C21A	N4A	C17A	N3A	1.0(13)	C21E	N4E	C17E	Ru1E	178.4(6)
C23A	N4A	C17A	Ru1A	4.8(19)	C21E	N4E	C17E	N3E	1.0(10)
C23A	N4A	C17A	N3A	-176.0(13)	C23E	N4E	C17E	Ru1E	-2.4(13)
C23A	N4A	C21A	C19A	176.9(13)	C23E	N4E	C17E	N3E	-179.8(8)
C23A	N4A	C21A	C22A	-4(2)	C23E	N4E	C21E	C19E	179.1(8)
C24A	Ru1A	C1A	O1A	54(5)	C23E	N4E	C21E	C22E	-1.3(13)
C24A	C26A	C28A	Ru1A	64.3(6)	C24E	C26E	C28E	Ru1E	62.1(6)
C24A	C26A	C28A	C29A	-171.0(10)	C24E	C26E	C28E	C29E	-174.3(7)
C24A	C26A	C28A	C30A	-1.3(11)	C24E	C26E	C28E	C30E	-1.6(9)
C25A	C24A	C26A	Ru1A	-118.5(10)	C25E	C24E	C26E	Ru1E	-121.2(8)
C25A	C24A	C26A	C27A	5.6(16)	C25E	C24E	C26E	C27E	0.1(13)
C25A	C24A	C26A	C28A	177.4(9)	C25E	C24E	C26E	C28E	176.1(8)
C25A	C24A	C32A	Ru1A	119.9(9)	C25E	C24E	C32E	Ru1E	121.6(8)
C25A	C24A	C32A	C30A	-176.4(9)	C25E	C24E	C32E	C30E	-175.9(8)
C25A	C24A	C32A	C33A	-5.2(15)	C25E	C24E	C32E	C33E	-2.0(14)
C26A	Ru1A	C1A	O1A	27(5)	C26E	C24E	C32E	Ru1E	-64.2(6)
C26A	C24A	C32A	Ru1A	-62.6(6)	C26E	C24E	C32E	C30E	-1.7(9)
C26A	C24A	C32A	C30A	1.1(10)	C26E	C24E	C32E	C33E	172.1(8)

Table S22 Torsion Angles for 4.

A	B	C	D	Angle/°	A	B	C	D	Angle/°
C26A	C24A	C32A	C33A	172.3(9)	C26E	C28E	C30E	Ru1E	62.9(5)
C26A	C28A	C30A	Ru1A	63.3(7)	C26E	C28E	C30E	C31E	-176.3(8)
C26A	C28A	C30A	C31A	-178.7(10)	C26E	C28E	C30E	C32E	0.6(9)
C26A	C28A	C30A	C32A	2.0(11)	C27E	C26E	C28E	Ru1E	-121.7(8)
C27A	C26A	C28A	Ru1A	-123.8(10)	C27E	C26E	C28E	C29E	1.8(12)
C27A	C26A	C28A	C29A	0.9(16)	C27E	C26E	C28E	C30E	174.6(7)
C27A	C26A	C28A	C30A	170.6(10)	C28E	C30E	C32E	Ru1E	62.0(6)
C28A	Ru1A	C1A	O1A	-9(5)	C28E	C30E	C32E	C24E	0.7(9)
C28A	C30A	C32A	Ru1A	60.2(7)	C28E	C30E	C32E	C33E	-173.3(8)
C28A	C30A	C32A	C24A	-1.9(11)	C29E	C28E	C30E	Ru1E	-124.8(8)
C28A	C30A	C32A	C33A	-173.1(9)	C29E	C28E	C30E	C31E	-3.9(14)
C29A	C28A	C30A	Ru1A	-127.5(11)	C29E	C28E	C30E	C32E	172.9(8)
C29A	C28A	C30A	C31A	-9.5(17)	C31E	C30E	C32E	Ru1E	-121.2(9)
C29A	C28A	C30A	C32A	171.2(10)	C31E	C30E	C32E	C24E	177.5(8)
C30A	Ru1A	C1A	O1A	-18(5)	C31E	C30E	C32E	C33E	3.5(14)
C31A	C30A	C32A	Ru1A	-119.1(10)	C32E	C24E	C26E	Ru1E	64.8(6)
C31A	C30A	C32A	C24A	178.8(9)	C32E	C24E	C26E	C27E	-174.0(8)
C31A	C30A	C32A	C33A	7.6(16)	C32E	C24E	C26E	C28E	2.0(9)
C32A	Ru1A	C1A	O1A	38(5)	Ru1F	C24F	C26F	C27F	125.3(9)
C32A	C24A	C26A	Ru1A	64.2(6)	Ru1F	C24F	C26F	C28F	-62.3(6)
C32A	C24A	C26A	C27A	-171.8(9)	Ru1F	C24F	C32F	C30F	62.6(6)
C32A	C24A	C26A	C28A	0.1(10)	Ru1F	C24F	C32F	C33F	-121.3(9)
Ru1B	C24B	C26B	C27B	124.9(9)	Ru1F	C26F	C28F	C29F	120.5(9)
Ru1B	C24B	C26B	C28B	-63.8(6)	Ru1F	C26F	C28F	C30F	-61.8(6)
Ru1B	C24B	C32B	C30B	62.8(6)	Ru1F	C28F	C30F	C31F	125.3(9)

Table S22 Torsion Angles for 4.

A	B	C	D	Angle/˚	A	B	C	D	Angle/˚
Ru1B	C24B	C32B	C33B	-121.7(9)	Ru1F	C28F	C30F	C32F	-63.0(6)
Ru1B	C26B	C28B	C29B	119.3(9)	Ru1F	C30F	C32F	C24F	-62.4(6)
Ru1B	C26B	C28B	C30B	-60.1(6)	Ru1F	C30F	C32F	C33F	121.2(8)
Ru1B	C28B	C30B	C31B	126.2(9)	N1F	C3F	C4F	N2F	-0.7(12)
Ru1B	C28B	C30B	C32B	-63.5(5)	N2F	C5F	C6F	C7F	-7.8(12)
Ru1B	C30B	C32B	C24B	-62.3(6)	N2F	C5F	C6F	C10F	173.0(8)
Ru1B	C30B	C32B	C33B	121.9(8)	N2F	C5F	C13F	C12F	-172.8(8)
N1B	C3B	C4B	N2B	-1.5(12)	N2F	C5F	C13F	C14F	4.8(13)
N2B	C5B	C6B	C7B	5.0(12)	N3F	C19F	C21F	N4F	0.6(10)
N2B	C5B	C6B	C10B	-173.1(8)	N3F	C19F	C21F	C22F	-175.7(9)
N2B	C5B	C13B	C12B	173.4(8)	C2F	Ru1F	C1F	O1F	-155(4)
N2B	C5B	C13B	C14B	-6.8(12)	C2F	N1F	C3F	C4F	-2.5(12)
N3B	C19B	C21B	N4B	-1.3(9)	C2F	N2F	C4F	C3F	3.3(11)
N3B	C19B	C21B	C22B	179.3(9)	C2F	N2F	C5F	C6F	84.4(12)
C2B	Ru1B	C1B	O1B	-145(4)	C2F	N2F	C5F	C13F	-102.7(12)
C2B	N1B	C3B	C4B	0.9(11)	C3F	N1F	C2F	Ru1F	-179.7(7)
C2B	N2B	C4B	C3B	1.6(11)	C3F	N1F	C2F	N2F	4.4(11)
C2B	N2B	C5B	C6B	-101.6(11)	C4F	N2F	C2F	Ru1F	179.7(7)
C2B	N2B	C5B	C13B	86.4(11)	C4F	N2F	C2F	N1F	-4.8(11)
C3B	N1B	C2B	Ru1B	179.8(6)	C4F	N2F	C5F	C6F	-76.2(12)
C3B	N1B	C2B	N2B	0.2(10)	C4F	N2F	C5F	C13F	96.7(11)
C4B	N2B	C2B	Ru1B	179.3(7)	C5F	N2F	C2F	Ru1F	17.0(15)
C4B	N2B	C2B	N1B	-1.1(10)	C5F	N2F	C2F	N1F	-167.5(9)
C4B	N2B	C5B	C6B	98.1(10)	C5F	N2F	C4F	C3F	167.7(9)
C4B	N2B	C5B	C13B	-73.9(11)	C5F	C6F	C7F	C8F	119.7(10)

Table S22 Torsion Angles for 4.

A	B	C	D	Angle/˚	A	B	C	D	Angle/˚
C5B	N2B	C2B	Ru1B	17.0(14)	C5F	C6F	C7F	C9F	-117.1(10)
C5B	N2B	C2B	N1B	-163.4(8)	C5F	C6F	C10F	C11F	-0.4(14)
C5B	N2B	C4B	C3B	165.5(9)	C5F	C13F	C14F	C15F	-91.6(11)
C5B	C6B	C7B	C8B	147.7(8)	C5F	C13F	C14F	C16F	145.4(8)
C5B	C6B	C7B	C9B	-89.1(10)	C6F	C5F	C13F	C12F	-0.2(13)
C5B	C6B	C10B	C11B	1.3(14)	C6F	C5F	C13F	C14F	177.4(8)
C5B	C13B	C14B	C15B	123.3(10)	C6F	C10F	C11F	C12F	0.4(15)
C5B	C13B	C14B	C16B	-113.4(10)	C7F	C6F	C10F	C11F	-179.6(9)
C6B	C5B	C13B	C12B	1.5(13)	C10F	C6F	C7F	C8F	-61.2(12)
C6B	C5B	C13B	C14B	-178.7(8)	C10F	C6F	C7F	C9F	62.0(12)
C6B	C10B	C11B	C12B	-1.6(15)	C10F	C11F	C12F	C13F	-0.3(15)
C7B	C6B	C10B	C11B	-176.7(9)	C11F	C12F	C13F	C5F	0.2(14)
C10B	C6B	C7B	C8B	-34.2(12)	C11F	C12F	C13F	C14F	-177.4(9)
C10B	C6B	C7B	C9B	88.9(11)	C12F	C13F	C14F	C15F	85.9(11)
C10B	C11B	C12B	C13B	1.8(14)	C12F	C13F	C14F	C16F	-37.1(12)
C11B	C12B	C13B	C5B	-1.7(13)	C13F	C5F	C6F	C7F	179.5(8)
C11B	C12B	C13B	C14B	178.4(9)	C13F	C5F	C6F	C10F	0.3(13)
C12B	C13B	C14B	C15B	-56.9(12)	C17F	Ru1F	C1F	O1F	113(4)
C12B	C13B	C14B	C16B	66.4(12)	C17F	N3F	C19F	C20F	-178.2(9)
C13B	C5B	C6B	C7B	176.8(8)	C17F	N3F	C19F	C21F	0.2(11)
C13B	C5B	C6B	C10B	-1.3(13)	C17F	N4F	C21F	C19F	-1.2(10)
C17B	Ru1B	C1B	O1B	124(4)	C17F	N4F	C21F	C22F	175.4(8)
C17B	N3B	C19B	C20B	176.2(8)	C18F	N3F	C17F	Ru1F	1.7(13)
C17B	N3B	C19B	C21B	0.7(10)	C18F	N3F	C17F	N4F	179.6(8)
C17B	N4B	C21B	C19B	1.6(10)	C18F	N3F	C19F	C20F	1.3(14)

Table S22 Torsion Angles for 4.

A	B	C	D	Angle/˚	A	B	C	D	Angle/˚
C17B	N4B	C21B	C22B	-178.9(9)	C18F	N3F	C19F	C21F	179.7(8)
C18B	N3B	C17B	Ru1B	-2.3(13)	C19F	N3F	C17F	Ru1F	-178.8(6)
C18B	N3B	C17B	N4B	-179.2(8)	C19F	N3F	C17F	N4F	-0.9(10)
C18B	N3B	C19B	C20B	-4.3(13)	C20F	C19F	C21F	N4F	178.8(10)
C18B	N3B	C19B	C21B	-179.8(8)	C20F	C19F	C21F	C22F	2.6(18)
C19B	N3B	C17B	Ru1B	177.2(6)	C21F	N4F	C17F	Ru1F	179.1(6)
C19B	N3B	C17B	N4B	0.3(9)	C21F	N4F	C17F	N3F	1.3(10)
C20B	C19B	C21B	N4B	-176.2(9)	C23F	N4F	C17F	Ru1F	-0.7(14)
C20B	C19B	C21B	C22B	4.3(17)	C23F	N4F	C17F	N3F	-178.5(9)
C21B	N4B	C17B	Ru1B	-178.2(6)	C23F	N4F	C21F	C19F	178.6(8)
C21B	N4B	C17B	N3B	-1.1(9)	C23F	N4F	C21F	C22F	-4.7(13)
C23B	N4B	C17B	Ru1B	3.5(12)	C24F	Ru1F	C1F	O1F	-59(4)
C23B	N4B	C17B	N3B	-179.4(8)	C24F	C26F	C28F	Ru1F	61.1(6)
C23B	N4B	C21B	C19B	179.9(8)	C24F	C26F	C28F	C29F	-178.4(8)
C23B	N4B	C21B	C22B	-0.6(13)	C24F	C26F	C28F	C30F	-0.7(10)
C24B	Ru1B	C1B	O1B	-48(4)	C25F	C24F	C26F	Ru1F	-121.4(8)
C24B	C26B	C28B	Ru1B	61.9(6)	C25F	C24F	C26F	C27F	3.9(14)
C24B	C26B	C28B	C29B	-178.8(8)	C25F	C24F	C26F	C28F	176.3(8)
C24B	C26B	C28B	C30B	1.8(10)	C25F	C24F	C32F	Ru1F	120.9(8)
C25B	C24B	C26B	Ru1B	-122.5(8)	C25F	C24F	C32F	C30F	-176.5(8)
C25B	C24B	C26B	C27B	2.4(14)	C25F	C24F	C32F	C33F	-0.4(14)
C25B	C24B	C26B	C28B	173.7(8)	C26F	Ru1F	C1F	O1F	-38(4)
C25B	C24B	C32B	Ru1B	121.7(8)	C26F	C24F	C32F	Ru1F	-64.8(6)
C25B	C24B	C32B	C30B	-175.5(8)	C26F	C24F	C32F	C30F	-2.2(10)
C25B	C24B	C32B	C33B	0.1(14)	C26F	C24F	C32F	C33F	174.0(8)

Table S22 Torsion Angles for 4.

A	B	C	D	Angle/˚	A	B	C	D	Angle/˚
C26B	Ru1B	C1B	O1B	-23(4)	C26F	C28F	C30F	Ru1F	62.4(6)
C26B	C24B	C32B	Ru1B	-64.8(6)	C26F	C28F	C30F	C31F	-172.3(8)
C26B	C24B	C32B	C30B	-2.0(9)	C26F	C28F	C30F	C32F	-0.6(10)
C26B	C24B	C32B	C33B	173.5(8)	C27F	C26F	C28F	Ru1F	-126.6(9)
C26B	C28B	C30B	Ru1B	60.5(6)	C27F	C26F	C28F	C29F	-6.1(15)
C26B	C28B	C30B	C31B	-173.3(8)	C27F	C26F	C28F	C30F	171.6(9)
C26B	C28B	C30B	C32B	-3.0(9)	C28F	Ru1F	C1F	O1F	18(4)
C27B	C26B	C28B	Ru1B	-126.8(9)	C28F	C30F	C32F	Ru1F	64.1(6)
C27B	C26B	C28B	C29B	-7.5(14)	C28F	C30F	C32F	C24F	1.7(9)
C27B	C26B	C28B	C30B	173.1(8)	C28F	C30F	C32F	C33F	-174.7(8)
C28B	Ru1B	C1B	O1B	29(4)	C29F	C28F	C30F	Ru1F	-119.8(9)
C28B	C30B	C32B	Ru1B	65.3(6)	C29F	C28F	C30F	C31F	5.5(15)
C28B	C30B	C32B	C24B	3.1(9)	C29F	C28F	C30F	C32F	177.1(8)
C28B	C30B	C32B	C33B	-172.7(8)	C30F	Ru1F	C1F	O1F	4(4)
C29B	C28B	C30B	Ru1B	-118.9(8)	C31F	C30F	C32F	Ru1F	-123.9(8)
C29B	C28B	C30B	C31B	7.3(14)	C31F	C30F	C32F	C24F	173.7(8)
C29B	C28B	C30B	C32B	177.6(8)	C31F	C30F	C32F	C33F	-2.7(13)
C30B	Ru1B	C1B	O1B	15(4)	C32F	Ru1F	C1F	O1F	-33(4)
C31B	C30B	C32B	Ru1B	-124.1(8)	C32F	C24F	C26F	Ru1F	64.1(6)
C31B	C30B	C32B	C24B	173.6(8)	C32F	C24F	C26F	C27F	-170.6(8)
C31B	C30B	C32B	C33B	-2.2(13)	C32F	C24F	C26F	C28F	1.8(10)
C32B	Ru1B	C1B	O1B	-22(4)	Ru1G	C24G	C26G	C27G	122.6(9)
C32B	C24B	C26B	Ru1B	64.0(6)	Ru1G	C24G	C26G	C28G	-62.0(6)
C32B	C24B	C26B	C27B	-171.2(8)	Ru1G	C24G	C32G	C30G	61.8(7)
C32B	C24B	C26B	C28B	0.1(10)	Ru1G	C24G	C32G	C33G	-117.4(11)

Table S22 Torsion Angles for 4.

A	B	C	D	Angle/˚	A	B	C	D	Angle/˚
Ru1C	C24C	C26C	C27C	118.3(11)	Ru1G	C26G	C28G	C29G	122.5(10)
Ru1C	C24C	C26C	C28C	-62.9(7)	Ru1G	C26G	C28G	C30G	-64.0(6)
Ru1C	C24C	C32C	C30C	60.9(6)	Ru1G	C28G	C30G	C31G	125.4(10)
Ru1C	C24C	C32C	C33C	-121.3(10)	Ru1G	C28G	C30G	C32G	-62.8(7)
Ru1C	C26C	C28C	C29C	123.2(11)	Ru1G	C30G	C32G	C24G	-60.8(7)
Ru1C	C26C	C28C	C30C	-62.4(7)	Ru1G	C30G	C32G	C33G	118.4(11)
Ru1C	C28C	C30C	C31C	124.5(10)	N1G	C3G	C4G	N2G	-1.5(12)
Ru1C	C28C	C30C	C32C	-64.7(7)	N2G	C5G	C6G	C7G	-9.1(18)
Ru1C	C30C	C32C	C24C	-61.9(7)	N2G	C5G	C6G	C10G	175.1(11)
Ru1C	C30C	C32C	C33C	120.2(10)	N2G	C5G	C13G	C12G	-172.3(11)
N1C	C3C	C4C	N2C	-1.3(10)	N2G	C5G	C13G	C14G	7.2(18)
N2C	C5C	C6C	C7C	-5.7(14)	N3G	C19G	C21G	N4G	2.8(12)
N2C	C5C	C6C	C10C	173.8(9)	N3G	C19G	C21G	C22G	-180.0(12)
N2C	C5C	C13C	C12C	-174.4(9)	C2G	Ru1G	C1G	O1G	-153(4)
N2C	C5C	C13C	C14C	4.1(14)	C2G	N1G	C3G	C4G	1.4(12)
N3C	C19C	C21C	N4C	1.6(11)	C2G	N2G	C4G	C3G	1.1(12)
N3C	C19C	C21C	C22C	-179.8(11)	C2G	N2G	C5G	C6G	90.1(14)
C2C	Ru1C	C1C	O1C	-147(4)	C2G	N2G	C5G	C13G	-98.5(13)
C2C	N1C	C3C	C4C	1.2(10)	C3G	N1G	C2G	Ru1G	179.9(7)
C2C	N2C	C4C	C3C	0.9(10)	C3G	N1G	C2G	N2G	-0.6(11)
C2C	N2C	C5C	C6C	86.4(12)	C4G	N2G	C2G	Ru1G	179.1(7)
C2C	N2C	C5C	C13C	-98.3(12)	C4G	N2G	C2G	N1G	-0.3(11)
C3C	N1C	C2C	Ru1C	177.3(6)	C4G	N2G	C5G	C6G	-74.0(13)
C3C	N1C	C2C	N2C	-0.5(9)	C4G	N2G	C5G	C13G	97.3(14)
C4C	N2C	C2C	Ru1C	-177.8(6)	C5G	N2G	C2G	Ru1G	13.5(16)

Table S22 Torsion Angles for 4.

A	B	C	D	Angle/˚	A	B	C	D	Angle/˚
C4C	N2C	C2C	N1C	-0.2(9)	C5G	N2G	C2G	N1G	-166.0(10)
C4C	N2C	C5C	C6C	-76.2(11)	C5G	N2G	C4G	C3G	168.1(10)
C4C	N2C	C5C	C13C	99.1(11)	C5G	C6G	C7G	C8G	113.8(14)
C5C	N2C	C2C	Ru1C	17.6(14)	C5G	C6G	C7G	C9G	-121.3(15)
C5C	N2C	C2C	N1C	-164.8(9)	C5G	C6G	C10G	C11G	-4(2)
C5C	N2C	C4C	C3C	167.0(8)	C5G	C13G	C14G	C15G	-81.5(15)
C5C	C6C	C7C	C8C	118.4(10)	C5G	C13G	C14G	C16G	159.3(13)
C5C	C6C	C7C	C9C	-117.2(10)	C6G	C5G	C13G	C12G	-1.9(19)
C5C	C6C	C10C	C11C	0.5(16)	C6G	C5G	C13G	C14G	177.6(11)
C5C	C13C	C14C	C15C	152.6(10)	C6G	C10G	C11G	C12G	2(2)
C5C	C13C	C14C	C16C	-84.4(12)	C7G	C6G	C10G	C11G	179.8(14)
C6C	C5C	C13C	C12C	0.5(15)	C10G	C6G	C7G	C8G	-70.5(16)
C6C	C5C	C13C	C14C	179.0(9)	C10G	C6G	C7G	C9G	54.5(17)
C6C	C10C	C11C	C12C	1.0(18)	C10G	C11G	C12G	C13G	0(2)
C7C	C6C	C10C	C11C	180.0(10)	C11G	C12G	C13G	C5G	0(2)
C10C	C6C	C7C	C8C	-61.0(12)	C11G	C12G	C13G	C14G	-179.5(12)
C10C	C6C	C7C	C9C	63.4(12)	C12G	C13G	C14G	C15G	98.0(16)
C10C	C11C	C12C	C13C	-1.9(18)	C12G	C13G	C14G	C16G	-21.2(17)
C11C	C12C	C13C	C5C	1.2(16)	C13G	C5G	C6G	C7G	179.7(13)
C11C	C12C	C13C	C14C	-177.4(10)	C13G	C5G	C6G	C10G	3.9(19)
C12C	C13C	C14C	C15C	-28.9(14)	C17G	Ru1G	C1G	O1G	116(4)
C12C	C13C	C14C	C16C	94.1(12)	C17G	N3G	C19G	C20G	177.3(10)
C13C	C5C	C6C	C7C	179.3(10)	C17G	N3G	C19G	C21G	-3.3(12)
C13C	C5C	C6C	C10C	-1.3(15)	C17G	N4G	C21G	C19G	-1.5(13)
C17C	Ru1C	C1C	O1C	123(4)	C17G	N4G	C21G	C22G	-179.0(10)

Table S22 Torsion Angles for 4.

A	B	C	D	Angle/˚	A	B	C	D	Angle/˚
C17C	N3C	C19C	C20C	175.9(10)	C18G	N3G	C17G	Ru1G	4.3(16)
C17C	N3C	C19C	C21C	-1.5(11)	C18G	N3G	C17G	N4G	-175.7(10)
C17C	N4C	C21C	C19C	-1.3(12)	C18G	N3G	C19G	C20G	-4.7(16)
C17C	N4C	C21C	C22C	-180.0(10)	C18G	N3G	C19G	C21G	174.7(10)
C18C	N3C	C17C	Ru1C	2.7(15)	C19G	N3G	C17G	Ru1G	-177.8(7)
C18C	N3C	C17C	N4C	-178.7(9)	C19G	N3G	C17G	N4G	2.3(11)
C18C	N3C	C19C	C20C	-4.7(15)	C20G	C19G	C21G	N4G	-177.9(12)
C18C	N3C	C19C	C21C	178.0(9)	C20G	C19G	C21G	C22G	-1(2)
C19C	N3C	C17C	Ru1C	-177.9(7)	C21G	N4G	C17G	Ru1G	179.6(7)
C19C	N3C	C17C	N4C	0.7(11)	C21G	N4G	C17G	N3G	-0.5(12)
C20C	C19C	C21C	N4C	-175.4(11)	C23G	N4G	C17G	Ru1G	-1.5(16)
C20C	C19C	C21C	C22C	3(2)	C23G	N4G	C17G	N3G	178.5(11)
C21C	N4C	C17C	Ru1C	179.1(7)	C23G	N4G	C21G	C19G	179.5(11)
C21C	N4C	C17C	N3C	0.3(11)	C23G	N4G	C21G	C22G	2.0(17)
C23C	N4C	C17C	Ru1C	-1.2(15)	C24G	Ru1G	C1G	O1G	8(4)
C23C	N4C	C17C	N3C	-179.9(10)	C24G	C26G	C28G	Ru1G	61.8(6)
C23C	N4C	C21C	C19C	179.0(10)	C24G	C26G	C28G	C29G	-175.7(10)
C23C	N4C	C21C	C22C	0.2(16)	C24G	C26G	C28G	C30G	-2.2(10)
C24C	Ru1C	C1C	O1C	-22(5)	C25G	C24G	C26G	Ru1G	-125.4(10)
C24C	C26C	C28C	Ru1C	63.4(7)	C25G	C24G	C26G	C27G	-2.8(16)
C24C	C26C	C28C	C29C	-173.3(10)	C25G	C24G	C26G	C28G	172.5(10)
C24C	C26C	C28C	C30C	1.0(12)	C25G	C24G	C32G	Ru1G	126.1(10)
C25C	C24C	C26C	Ru1C	-123.3(11)	C25G	C24G	C32G	C30G	-172.1(10)
C25C	C24C	C26C	C27C	-5.0(18)	C25G	C24G	C32G	C33G	8.7(17)
C25C	C24C	C26C	C28C	173.8(10)	C26G	Ru1G	C1G	O1G	-28(4)

Table S22 Torsion Angles for 4.

A	B	C	D	Angle/˚	A	B	C	D	Angle/˚
C25C	C24C	C32C	Ru1C	123.9(10)	C26G	C24G	C32G	Ru1G	-64.6(7)
C25C	C24C	C32C	C30C	-175.1(10)	C26G	C24G	C32G	C30G	-2.8(11)
C25C	C24C	C32C	C33C	2.6(17)	C26G	C24G	C32G	C33G	178.0(10)
C26C	Ru1C	C1C	O1C	27(5)	C26G	C28G	C30G	Ru1G	63.3(6)
C26C	C24C	C32C	Ru1C	-62.6(7)	C26G	C28G	C30G	C31G	-171.2(9)
C26C	C24C	C32C	C30C	-1.7(11)	C26G	C28G	C30G	C32G	0.5(10)
C26C	C24C	C32C	C33C	176.1(10)	C27G	C26G	C28G	Ru1G	-122.9(9)
C26C	C28C	C30C	Ru1C	62.6(7)	C27G	C26G	C28G	C29G	-0.4(16)
C26C	C28C	C30C	C31C	-172.9(9)	C27G	C26G	C28G	C30G	173.1(9)
C26C	C28C	C30C	C32C	-2.1(11)	C28G	Ru1G	C1G	O1G	-56(4)
C27C	C26C	C28C	Ru1C	-117.7(10)	C28G	C30G	C32G	Ru1G	62.2(7)
C27C	C26C	C28C	C29C	5.5(17)	C28G	C30G	C32G	C24G	1.4(11)
C27C	C26C	C28C	C30C	179.8(9)	C28G	C30G	C32G	C33G	-179.4(10)
C28C	Ru1C	C1C	O1C	11(4)	C29G	C28G	C30G	Ru1G	-123.1(11)
C28C	C30C	C32C	Ru1C	64.3(7)	C29G	C28G	C30G	C31G	2.3(16)
C28C	C30C	C32C	C24C	2.4(11)	C29G	C28G	C30G	C32G	174.0(10)
C28C	C30C	C32C	C33C	-175.5(10)	C30G	Ru1G	C1G	O1G	-40(4)
C29C	C28C	C30C	Ru1C	-122.8(10)	C31G	C30G	C32G	Ru1G	-126.3(10)
C29C	C28C	C30C	C31C	1.7(16)	C31G	C30G	C32G	C24G	172.9(9)
C29C	C28C	C30C	C32C	172.5(10)	C31G	C30G	C32G	C33G	-7.9(17)
C30C	Ru1C	C1C	O1C	-24(4)	C32G	Ru1G	C1G	O1G	19(4)
C31C	C30C	C32C	Ru1C	-124.9(10)	C32G	C24G	C26G	Ru1G	65.1(7)
C31C	C30C	C32C	C24C	173.1(10)	C32G	C24G	C26G	C27G	-172.3(9)
C31C	C30C	C32C	C33C	-4.7(16)	C32G	C24G	C26G	C28G	3.1(10)
C32C	Ru1C	C1C	O1C	-49(5)	Ru1H	C24H	C26H	C27H	119.3(10)

Table S22 Torsion Angles for 4.

A	B	C	D	Angle/˚	A	B	C	D	Angle/˚
C32C	C24C	C26C	Ru1C	63.4(7)	Ru1H	C24H	C26H	C28H	-61.9(7)
C32C	C24C	C26C	C27C	-178.3(10)	Ru1H	C24H	C32H	C30H	63.7(7)
C32C	C24C	C26C	C28C	0.5(12)	Ru1H	C24H	C32H	C33H	-124.2(10)
Ru1D	C24D	C26D	C27D	120.3(8)	Ru1H	C26H	C28H	C29H	126.1(12)
Ru1D	C24D	C26D	C28D	-62.0(6)	Ru1H	C26H	C28H	C30H	-62.2(7)
Ru1D	C24D	C32D	C30D	63.0(6)	Ru1H	C28H	C30H	C31H	124.2(10)
Ru1D	C24D	C32D	C33D	-122.1(8)	Ru1H	C28H	C30H	C32H	-63.0(7)
Ru1D	C26D	C28D	C29D	126.7(10)	Ru1H	C30H	C32H	C24H	-65.0(7)
Ru1D	C26D	C28D	C30D	-61.5(6)	Ru1H	C30H	C32H	C33H	123.2(11)
Ru1D	C28D	C30D	C31D	121.2(8)	N1H	C3H	C4H	N2H	0.7(11)
Ru1D	C28D	C30D	C32D	-63.4(6)	N2H	C5H	C6H	C7H	7.0(15)
Ru1D	C30D	C32D	C24D	-63.0(6)	N2H	C5H	C6H	C10H	-172.7(9)
Ru1D	C30D	C32D	C33D	122.4(9)	N2H	C5H	C13H	C12H	173.9(9)
N1D	C3D	C4D	N2D	1.8(11)	N2H	C5H	C13H	C14H	-6.9(15)
N2D	C5D	C6D	C7D	6.6(12)	N3H	C19H	C21H	N4H	-2.8(12)
N2D	C5D	C6D	C10D	-174.3(8)	N3H	C19H	C21H	C22H	177.4(12)
N2D	C5D	C13D	C12D	173.3(8)	C2H	Ru1H	C1H	O1H	142(5)
N2D	C5D	C13D	C14D	-2.9(13)	C2H	N1H	C3H	C4H	-1.5(11)
N3D	C19D	C21D	N4D	-1.1(10)	C2H	N2H	C4H	C3H	0.2(10)
N3D	C19D	C21D	C22D	174.6(10)	C2H	N2H	C5H	C6H	-88.2(13)
C2D	Ru1D	C1D	O1D	153(4)	C2H	N2H	C5H	C13H	98.6(12)
C2D	N1D	C3D	C4D	-1.9(11)	C3H	N1H	C2H	Ru1H	-177.0(6)
C2D	N2D	C4D	C3D	-1.0(11)	C3H	N1H	C2H	N2H	1.5(10)
C2D	N2D	C5D	C6D	-85.2(11)	C4H	N2H	C2H	Ru1H	177.3(7)
C2D	N2D	C5D	C13D	98.8(11)	C4H	N2H	C2H	N1H	-1.0(10)

Table S22 Torsion Angles for 4.

A	B	C	D	Angle/˚	A	B	C	D	Angle/˚
C3D	N1D	C2D	Ru1D	-179.0(6)	C4H	N2H	C5H	C6H	76.4(12)
C3D	N1D	C2D	N2D	1.2(10)	C4H	N2H	C5H	C13H	-96.8(12)
C4D	N2D	C2D	Ru1D	-179.9(6)	C5H	N2H	C2H	Ru1H	-16.6(14)
C4D	N2D	C2D	N1D	-0.2(10)	C5H	N2H	C2H	N1H	165.0(9)
C4D	N2D	C5D	C6D	79.5(11)	C5H	N2H	C4H	C3H	-167.4(9)
C4D	N2D	C5D	C13D	-96.5(11)	C5H	C6H	C7H	C8H	113.8(12)
C5D	N2D	C2D	Ru1D	-13.7(14)	C5H	C6H	C7H	C9H	-121.4(11)
C5D	N2D	C2D	N1D	166.0(9)	C5H	C6H	C10H	C11H	1.2(17)
C5D	N2D	C4D	C3D	-168.6(9)	C5H	C13H	C14H	C15H	-151.7(11)
C5D	C6D	C7D	C8D	-116.8(10)	C5H	C13H	C14H	C16H	85.5(13)
C5D	C6D	C7D	C9D	118.0(10)	C6H	C5H	C13H	C12H	1.1(16)
C5D	C6D	C10D	C11D	0.8(14)	C6H	C5H	C13H	C14H	-179.7(10)
C5D	C13D	C14D	C15D	-145.9(9)	C6H	C10H	C11H	C12H	-4.2(18)
C5D	C13D	C14D	C16D	90.3(10)	C7H	C6H	C10H	C11H	-178.4(11)
C6D	C5D	C13D	C12D	-2.4(13)	C10H	C6H	C7H	C8H	-66.6(14)
C6D	C5D	C13D	C14D	-178.6(9)	C10H	C6H	C7H	C9H	58.2(13)
C6D	C10D	C11D	C12D	-2.0(15)	C10H	C11H	C12H	C13H	5.7(18)
C7D	C6D	C10D	C11D	-180.0(9)	C11H	C12H	C13H	C5H	-4.1(17)
C10D	C6D	C7D	C8D	64.0(12)	C11H	C12H	C13H	C14H	176.7(11)
C10D	C6D	C7D	C9D	-61.1(12)	C12H	C13H	C14H	C15H	27.6(15)
C10D	C11D	C12D	C13D	0.9(15)	C12H	C13H	C14H	C16H	-95.3(14)
C11D	C12D	C13D	C5D	1.2(14)	C13H	C5H	C6H	C7H	179.9(10)
C11D	C12D	C13D	C14D	177.5(9)	C13H	C5H	C6H	C10H	0.3(16)
C12D	C13D	C14D	C15D	38.1(12)	C17H	Ru1H	C1H	O1H	-128(5)
C12D	C13D	C14D	C16D	-85.6(11)	C17H	N3H	C19H	C20H	176.8(10)

Table S22 Torsion Angles for 4.

A	B	C	D	Angle/˚	A	B	C	D	Angle/˚
C13D	C5D	C6D	C7D	-177.7(8)	C17H	N3H	C19H	C21H	0.6(13)
C13D	C5D	C6D	C10D	1.5(13)	C17H	N4H	C21H	C19H	4.4(12)
C17D	Ru1D	C1D	O1D	-116(4)	C17H	N4H	C21H	C22H	-175.9(10)
C17D	N3D	C19D	C20D	176.7(9)	C18H	N3H	C17H	Ru1H	-4.3(15)
C17D	N3D	C19D	C21D	0.7(11)	C18H	N3H	C17H	N4H	179.0(10)
C17D	N4D	C21D	C19D	1.2(10)	C18H	N3H	C19H	C20H	-0.2(17)
C17D	N4D	C21D	C22D	-175.1(8)	C18H	N3H	C19H	C21H	-176.5(10)
C18D	N3D	C17D	Ru1D	-1.9(13)	C19H	N3H	C17H	Ru1H	178.7(8)
C18D	N3D	C17D	N4D	-179.2(8)	C19H	N3H	C17H	N4H	2.1(12)
C18D	N3D	C19D	C20D	-4.0(13)	C20H	C19H	C21H	N4H	-178.7(11)
C18D	N3D	C19D	C21D	179.9(8)	C20H	C19H	C21H	C22H	2(2)
C19D	N3D	C17D	Ru1D	177.3(7)	C21H	N4H	C17H	Ru1H	179.8(8)
C19D	N3D	C17D	N4D	0.0(10)	C21H	N4H	C17H	N3H	-3.8(11)
C20D	C19D	C21D	N4D	-176.7(10)	C23H	N4H	C17H	Ru1H	2.6(16)
C20D	C19D	C21D	C22D	-1.0(18)	C23H	N4H	C17H	N3H	179.0(9)
C21D	N4D	C17D	Ru1D	-178.0(7)	C23H	N4H	C21H	C19H	-178.3(10)
C21D	N4D	C17D	N3D	-0.7(10)	C23H	N4H	C21H	C22H	1.4(15)
C23D	N4D	C17D	Ru1D	1.1(14)	C24H	Ru1H	C1H	O1H	-17(5)
C23D	N4D	C17D	N3D	178.4(8)	C24H	C26H	C28H	Ru1H	62.0(8)
C23D	N4D	C21D	C19D	-178.0(8)	C24H	C26H	C28H	C29H	-171.9(11)
C23D	N4D	C21D	C22D	5.8(14)	C24H	C26H	C28H	C30H	-0.1(12)
C24D	Ru1D	C1D	O1D	-7(4)	C25H	C24H	C26H	Ru1H	-126.1(12)
C24D	C26D	C28D	Ru1D	61.4(6)	C25H	C24H	C26H	C27H	-6.8(18)
C24D	C26D	C28D	C29D	-171.9(9)	C25H	C24H	C26H	C28H	172.0(11)
C24D	C26D	C28D	C30D	-0.1(10)	C25H	C24H	C32H	Ru1H	124.5(12)

Table S22 Torsion Angles for 4.

A	B	C	D	Angle/˚	A	B	C	D	Angle/˚
C25D	C24D	C26D	Ru1D	-126.4(9)	C25H	C24H	C32H	C30H	-171.8(11)
C25D	C24D	C26D	C27D	-6.0(14)	C25H	C24H	C32H	C33H	0.3(18)
C25D	C24D	C26D	C28D	171.6(8)	C26H	Ru1H	C1H	O1H	-31(5)
C25D	C24D	C32D	Ru1D	124.8(8)	C26H	C24H	C32H	Ru1H	-63.3(7)
C25D	C24D	C32D	C30D	-172.2(8)	C26H	C24H	C32H	C30H	0.4(12)
C25D	C24D	C32D	C33D	2.8(13)	C26H	C24H	C32H	C33H	172.6(9)
C26D	Ru1D	C1D	O1D	-21(5)	C26H	C28H	C30H	Ru1H	63.4(7)
C26D	C24D	C32D	Ru1D	-64.0(6)	C26H	C28H	C30H	C31H	-172.4(9)
C26D	C24D	C32D	C30D	-1.1(9)	C26H	C28H	C30H	C32H	0.4(11)
C26D	C24D	C32D	C33D	173.9(8)	C27H	C26H	C28H	Ru1H	-119.1(9)
C26D	C28D	C30D	Ru1D	62.8(6)	C27H	C26H	C28H	C29H	7.0(17)
C26D	C28D	C30D	C31D	-176.0(8)	C27H	C26H	C28H	C30H	178.7(9)
C26D	C28D	C30D	C32D	-0.5(9)	C28H	Ru1H	C1H	O1H	20(5)
C27D	C26D	C28D	Ru1D	-121.0(9)	C28H	C30H	C32H	Ru1H	64.5(7)
C27D	C26D	C28D	C29D	5.8(15)	C28H	C30H	C32H	C24H	-0.5(12)
C27D	C26D	C28D	C30D	177.5(8)	C28H	C30H	C32H	C33H	-172.3(10)
C28D	Ru1D	C1D	O1D	34(5)	C29H	C28H	C30H	Ru1H	-124.5(11)
C28D	C30D	C32D	Ru1D	63.9(6)	C29H	C28H	C30H	C31H	-0.3(17)
C28D	C30D	C32D	C24D	1.0(9)	C29H	C28H	C30H	C32H	172.4(10)
C28D	C30D	C32D	C33D	-173.6(8)	C30H	Ru1H	C1H	O1H	44(5)
C29D	C28D	C30D	Ru1D	-125.1(9)	C31H	C30H	C32H	Ru1H	-122.6(10)
C29D	C28D	C30D	C31D	-4.0(14)	C31H	C30H	C32H	C24H	172.5(9)
C29D	C28D	C30D	C32D	171.5(8)	C31H	C30H	C32H	C33H	0.6(16)
C30D	Ru1D	C1D	O1D	56(4)	C32H	Ru1H	C1H	O1H	19(5)
C31D	C30D	C32D	Ru1D	-120.7(8)	C32H	C24H	C26H	Ru1H	61.7(7)

Table S22 Torsion Angles for 4.

A	B	C	D	Angle/˚	A	B	C	D	Angle/˚
C31D	C30D	C32D	C24D	176.4(8)	C32H	C24H	C26H	C27H	-179.0(9)
C31D	C30D	C32D	C33D	1.7(14)	C32H	C24H	C26H	C28H	-0.1(12)
C32D	Ru1D	C1D	O1D	30(4)					

Table S23 Hydrogen Atom Coordinates (Å×10[4]) and Isotropic Displacement Parameters (Å[2]×10[3]) for 4.

Atom	*x*	*y*	*z*	U(eq)
H3A	6770	5562	5285	35
H4A	7675	6486	4774	38
H7A	5070	6953	3932	58
H8AA	6288	7441	4509	117
H8AB	5181	7933	4410	117
H8AC	6434	8121	4184	117
H9AA	5302	8237	3440	127
H9AB	4115	7833	3566	127
H9AC	4962	7566	3154	127
H10A	6985	7761	3118	52
H11A	8743	7309	2819	55
H12A	9515	6296	3134	55
H14A	8219	5265	4081	47
H15A	10486	5853	3903	70
H15B	10257	5209	4255	70
H15C	9736	5947	4380	70
H16A	8302	4834	3305	72
H16B	9367	4527	3617	72

Table S23 Hydrogen Atom Coordinates (Å×10[4]) and Isotropic Displacement Parameters (Å[2]×10[3]) for 4.

**Atom**	***x***	***y***	***z***	**U(eq)**
H16C	9600	5135	3236	72
H18A	2599	5897	4774	60
H18B	3908	6193	4789	60
H18C	3681	5433	4608	60
H20A	1222	6829	4518	84
H20B	1551	7536	4249	84
H20C	2418	7214	4645	84
H22A	2443	7610	3074	107
H22B	1309	7560	3411	107
H22C	1451	7051	2975	107
H23A	4119	6456	2751	116
H23B	2759	6272	2684	116
H23C	3687	5680	2821	116
H25A	7161	3784	3880	45
H25B	6396	3321	4241	45
H25C	6663	4104	4360	45
H27A	6031	4259	2717	70
H27B	5923	3462	2857	70
H27C	6892	3923	3109	70
H29A	2750	4599	2811	96
H29B	3222	3845	2702	96
H29C	4001	4501	2560	96
H31A	2114	4613	4134	86
H31B	1754	4011	3786	86

Table S23 Hydrogen Atom Coordinates (Å×10[4]) and Isotropic Displacement Parameters (Å[2]×10[3]) for 4.

Atom	*x*	*y*	*z*	U(eq)
H31C	1938	4774	3577	86
H33A	4469	4365	4789	73
H33B	4348	3559	4708	73
H33C	3218	4047	4658	73
H3B	6236	9051	4672	37
H4B	7513	9896	5040	37
H7B	5513	10472	6024	34
H8BA	5569	11000	6785	52
H8BB	4842	11461	6408	52
H8BC	6158	11667	6544	52
H9BA	6714	11677	5683	58
H9BB	5417	11453	5531	58
H9BC	6530	11010	5371	58
H10B	7917	11247	6611	35
H11B	9758	10751	6751	41
H12B	10193	9681	6453	37
H14B	8332	8716	5684	39
H15D	10025	9279	5355	67
H15E	10254	8474	5440	67
H15F	10780	9014	5804	67
H16D	9744	8451	6511	54
H16E	9355	7876	6146	54
H16F	8386	8253	6460	54
H18D	4030	9862	5409	55

Table S23 Hydrogen Atom Coordinates (Å×10^4) and Isotropic Displacement Parameters (Å^2×10^3) for 4.

**Atom**	***x***	***y***	***z***	**U(eq)**
H18E	2647	9732	5462	55
H18F	3557	9117	5563	55
H20D	2540	10948	5678	59
H20E	1810	11189	6132	59
H20F	1394	10534	5845	59
H22D	2189	10592	7346	62
H22E	2171	11208	6959	62
H22F	3332	11056	7266	62
H23D	4637	9236	7339	37
H23E	3756	9793	7550	37
H23F	5026	10022	7361	37
H25D	5946	6811	5715	41
H25E	6990	7263	5923	41
H25F	6152	7577	5514	41
H27D	4040	7901	5365	44
H27E	2823	8018	5638	44
H27F	3306	7256	5564	44
H29D	2147	8396	6353	43
H29E	2418	8430	6911	43
H29F	1937	7735	6691	43
H31D	3721	8145	7499	38
H31E	5089	7988	7581	38
H31F	4173	7369	7554	38
H33D	6527	6842	7053	37

Table S23 Hydrogen Atom Coordinates (Å×10^4) and Isotropic Displacement Parameters (Å^2×10^3) for 4.

Atom	*x*	*y*	*z*	U(eq)
H33E	6802	7613	7201	37
H33F	7332	7322	6718	37
H3C	-575	675	5306	32
H4C	-1669	1537	4840	28
H7C	-2454	324	4207	40
H8CA	-4032	978	4487	63
H8CB	-4453	209	4398	63
H8CC	-4783	809	4023	63
H9CA	-3735	135	3344	52
H9CB	-3575	-456	3744	52
H9CC	-2453	-165	3462	52
H10C	-4039	1344	3327	47
H11C	-3372	2364	2975	52
H12C	-1526	2778	3157	48
H14C	657	1953	3866	47
H15G	664	2511	3080	77
H15H	1493	2880	3455	77
H15I	233	3201	3325	77
H16G	-541	3170	4156	67
H16H	765	2960	4315	67
H16I	-327	2505	4491	67
H18G	1963	1364	4674	53
H18H	3278	1070	4661	53
H18I	2194	580	4546	53

Table S23 Hydrogen Atom Coordinates (Å×10^4) and Isotropic Displacement Parameters (Å^2×10^3) for 4.

Atom	*x*	*y*	*z*	U(eq)
H20G	4492	1955	4377	84
H20H	3388	2465	4391	84
H20I	4390	2578	3997	84
H22G	3127	2463	2788	81
H22H	4302	2008	2808	81
H22I	4156	2645	3152	81
H23G	1809	683	2736	73
H23H	2710	1256	2546	73
H23I	1386	1464	2673	73
H25G	3030	-613	4493	65
H25H	2311	-1301	4610	65
H25I	1748	-571	4726	65
H27G	3803	-109	3763	95
H27H	3659	-186	3200	95
H27I	4075	-829	3524	95
H29G	1096	-458	2504	92
H29H	1841	-1154	2553	92
H29I	2476	-429	2613	92
H31G	-846	-766	2875	84
H31H	-1292	-1228	3318	84
H31I	-430	-1549	2931	84
H33G	-187	-1677	4359	61
H33H	-1146	-1224	4076	61
H33I	-432	-902	4508	61

Table S23 Hydrogen Atom Coordinates (Å×10⁴) and Isotropic Displacement Parameters (Å²×10³) for 4.

Atom	*x*	*y*	*z*	U(eq)
H3D	363	4107	4660	37
H4D	-1027	4894	5021	35
H7D	-1791	3643	5704	37
H8DA	-4232	3978	5692	70
H8DB	-3670	3341	5422	70
H8DC	-3332	4109	5261	70
H9DA	-2027	3238	6483	54
H9DB	-2841	2814	6132	54
H9DC	-3409	3392	6457	54
H10D	-3824	4607	6411	38
H11D	-3496	5686	6696	43
H12D	-1704	6234	6570	40
H14D	808	5488	6004	37
H15J	681	6732	6382	59
H15K	1388	6077	6585	59
H15L	56	6205	6745	59
H16J	-275	6045	5357	69
H16K	820	6495	5521	69
H16L	-482	6694	5686	69
H18J	1289	5025	7400	39
H18K	2540	4750	7585	39
H18L	1628	4233	7352	39
H20J	2878	6142	7256	59
H20K	4185	6178	7046	59

Table S23 Hydrogen Atom Coordinates (Å×10^4) and Isotropic Displacement Parameters (Å^2×10^3) for 4.

Atom	*x*	*y*	*z*	U(eq)
H20L	3860	5612	7446	59
H22J	4959	5603	5919	49
H22K	4466	6247	6201	49
H22L	3793	5991	5738	49
H23J	2800	4155	5602	46
H23K	3836	4702	5533	46
H23L	2500	4928	5438	46
H25J	2618	3167	7508	42
H25K	2309	2367	7546	42
H25L	1285	2929	7585	42
H27J	4249	3435	6374	41
H27K	4473	2743	6679	41
H27L	3984	3412	6933	41
H29J	2374	2913	5372	45
H29K	3099	2260	5567	45
H29L	3598	3016	5641	45
H31J	-591	2362	5901	42
H31K	370	1806	5750	42
H31L	359	2551	5498	42
H33J	-472	2654	7177	38
H33K	-99	1875	7088	38
H33L	-920	2277	6711	38
H3E	1224	8916	10302	43
H4E	2496	9787	9929	45

Table S23 Hydrogen Atom Coordinates (Å×10^4) and Isotropic Displacement Parameters (Å^2×10^3) for 4.

**Atom**	***x***	***y***	***z***	**U(eq)**
H7E	515	10410	8926	32
H8EA	1253	10879	9600	65
H8EB	463	11475	9361	65
H8EC	1863	11498	9310	65
H9EA	1177	11590	8372	61
H9EB	-130	11450	8561	61
H9EC	451	10950	8183	61
H10E	2935	11222	8337	35
H11E	4770	10727	8219	40
H12E	5192	9617	8527	39
H14E	3278	8629	9282	42
H15M	5734	8966	9208	73
H15N	5222	8351	9534	73
H15O	4925	9129	9658	73
H16M	3410	8225	8501	55
H16N	4315	7811	8837	55
H16O	4781	8405	8482	55
H18M	-1349	9049	9430	55
H18N	-2388	9588	9515	55
H18O	-1048	9816	9580	55
H20M	-3596	10484	9159	69
H20N	-3244	11132	8830	69
H20O	-2458	10925	9284	69
H22M	-1677	11092	7724	64

Table S23 Hydrogen Atom Coordinates (Å×10^4) and Isotropic Displacement Parameters (Å^2×10^3) for 4.

Atom	*x*	*y*	*z*	U(eq)
H22N	-2843	11224	8028	64
H22O	-2825	10640	7638	64
H23M	-37	10057	7609	35
H23N	-1309	9822	7433	35
H23O	-398	9265	7643	35
H25M	1962	7170	9055	31
H25N	896	6751	9292	31
H25O	1177	7512	9459	31
H27M	1854	7658	7805	34
H27N	1486	6874	7895	34
H27O	2293	7251	8275	34
H29M	-1237	8209	7473	38
H29N	-881	7419	7426	38
H29O	111	7998	7401	38
H31M	-2926	8308	8643	39
H31N	-3057	7741	8244	39
H31O	-2598	8494	8101	39
H33M	-963	7784	9615	44
H33N	-1776	7193	9409	44
H33O	-2160	7980	9344	44
H3F	5334	3984	10318	41
H4F	3952	4795	9954	38
H7F	3211	3601	9286	39
H8FA	1525	4131	9666	81

Table S23 Hydrogen Atom Coordinates (Å×10[4]) and Isotropic Displacement Parameters (Å[2]×10[3]) for 4.

Atom	*x*	*y*	*z*	U(eq)
H8FB	1402	3318	9618	81
H8FC	714	3825	9257	81
H9FA	1621	3384	8522	50
H9FB	2154	2788	8860	50
H9FC	3000	3218	8510	50
H10F	1195	4597	8568	40
H11F	1554	5694	8249	42
H12F	3332	6203	8384	37
H14F	5828	5427	8964	36
H15P	4538	6598	9270	75
H15Q	5871	6424	9413	75
H15R	4829	5940	9602	75
H16P	5336	6084	8172	42
H16Q	6544	6203	8449	42
H16R	5466	6722	8515	42
H18P	6719	4239	7617	39
H18Q	7546	4822	7388	39
H18R	6272	5018	7586	39
H20P	8933	5633	7529	57
H20Q	9211	6189	7923	57
H20R	7925	6151	7694	57
H22P	8752	6026	9170	55
H22Q	9651	6131	8736	55
H22R	9845	5515	9116	55

Table S23 Hydrogen Atom Coordinates (Å×10^4) and Isotropic Displacement Parameters (Å^2×10^3) for 4.

Atom	*x*	*y*	*z*	U(eq)
H23P	7616	4894	9539	50
H23Q	8880	4570	9429	50
H23R	7725	4108	9392	50
H25P	5158	2586	9452	42
H25Q	5609	1822	9357	42
H25R	4497	2125	9072	42
H27P	8596	2944	9345	46
H27Q	8131	2175	9417	46
H27R	7381	2801	9617	46
H29P	8973	3490	8099	46
H29Q	9448	2738	8242	46
H29R	9318	3307	8640	46
H31P	6269	2979	7408	39
H31Q	7262	2395	7439	39
H31R	7618	3186	7474	39
H33P	4084	2271	8298	38
H33Q	4888	1872	7926	38
H33R	4538	2658	7824	38
H3G	1731	5450	-329	51
H4G	2708	6348	153	55
H7G	3249	5122	871	71
H8GA	4797	5862	577	97
H8GB	5216	5081	643	97
H8GC	5576	5634	1028	97

Table S23 Hydrogen Atom Coordinates (Å×10^4) and Isotropic Displacement Parameters (Å^2×10^3) for 4.

Atom	*x*	*y*	*z*	U(eq)
H9GA	4701	4964	1685	97
H9GB	4254	4367	1349	97
H9GC	3341	4757	1683	97
H10G	4627	6141	1763	81
H11G	3899	7181	2045	85
H12G	2102	7625	1757	77
H14G	21	6798	1042	63
H15S	1383	7940	706	146
H15T	126	7728	502	146
H15U	1244	7243	421	146
H16S	12	7426	1759	150
H16T	-681	7816	1335	150
H16U	617	8053	1475	150
H18S	-1231	6115	166	62
H18T	-2437	5701	221	62
H18U	-1219	5342	371	62
H20S	-2607	7240	375	85
H20T	-3711	7308	724	85
H20U	-3625	6676	372	85
H22S	-3483	6858	2015	91
H22T	-3755	7351	1565	91
H22U	-2570	7446	1863	91
H23S	-1173	5545	2151	76
H23T	-2177	6087	2281	76

Table S23 Hydrogen Atom Coordinates (Å×10[4]) and Isotropic Displacement Parameters (Å[2]×10[3]) for 4.

Atom	*x*	*y*	*z*	U(eq)
H23U	-859	6342	2189	76
H25S	-891	4350	2434	98
H25T	-1635	3675	2330	98
H25U	-2165	4416	2194	98
H27S	1970	3704	1856	63
H27T	1006	3271	2148	63
H27U	1184	4068	2251	63
H29S	1632	3897	586	80
H29T	1389	3131	778	80
H29U	2178	3636	1084	80
H31S	-1801	4006	338	74
H31T	-803	3427	309	74
H31U	-483	4214	199	74
H33S	-3012	4682	1427	102
H33T	-3264	3909	1280	102
H33U	-2917	4473	881	102
H3H	4424	552	-345	36
H4H	3303	1415	100	40
H7H	2467	199	741	47
H8HA	2469	-293	1505	71
H8HB	1466	-627	1186	71
H8HC	1124	-73	1575	71
H9HA	83	585	925	70
H9HB	510	65	527	70

Table S23 Hydrogen Atom Coordinates (Å×10[4]) and Isotropic Displacement Parameters (Å[2]×10[3]) for 4.

Atom	*x*	*y*	*z*	U(eq)
H9HC	812	864	473	70
H10H	832	1174	1581	53
H11H	1421	2221	1888	54
H12H	3328	2618	1755	57
H14H	5570	1796	1092	56
H15V	5115	3047	1583	98
H15W	6379	2711	1491	98
H15X	5487	2354	1861	98
H16V	4618	2354	443	94
H16W	5733	2785	609	94
H16X	4436	3023	754	94
H18V	6103	1203	2245	71
H18W	7433	1237	2428	71
H18X	6884	519	2280	71
H20V	7973	2400	2087	78
H20W	9193	2406	1803	78
H20X	8965	1842	2215	78
H22V	9521	1798	643	80
H22W	9247	2455	955	80
H22X	8374	2254	535	80
H23V	7127	442	439	60
H23W	8233	924	324	60
H23X	6929	1233	294	60
H25V	6167	-608	2503	103

Table S23 Hydrogen Atom Coordinates (Å×10^4) and Isotropic Displacement Parameters (Å^2×10^3) for 4.

Atom	*x*	*y*	*z*	U(eq)
H25W	7533	-670	2373	103
H25X	6766	-1348	2463	103
H27V	8806	-456	1151	96
H27W	8967	-998	1584	96
H27X	8597	-223	1687	96
H29V	6629	-740	266	78
H29W	7224	-1460	414	78
H29X	7925	-760	489	78
H31V	3732	-1285	956	52
H31W	4607	-1865	769	52
H31X	4535	-1134	498	52
H33V	4117	-937	2143	89
H33W	4519	-1720	2098	89
H33X	3646	-1381	1712	89

Experimental

Single crystals of $C_{33}H_{46}N_4ORu$ **4** were grown from toluene at – 30 °C. A yellow block-shaped crystal with dimensions 0.20×0.14×0.10 mm^3 was mounted on a Kapton loop. Data were collected using a ROD, Synergy Custom DW system, Pilatus 200K diffractometer operating at T = 100.00 K.

Data were measured using *w* scans of 0.5° per frame for 4.75 s using Mo K_a radiation. The diffraction pattern was indexed and the total number of runs and images was based on the strategy calculation from the program CrysAlisPro (Rigaku, V1.171.42.90a, 2023) The maximum resolution that was achieved was Q = 26.372° (0.80 Å). The unit cell was refined using CrysAlisPro (Rigaku, V1.171.42.90a, 2023) on 106227 reflections, 42% of the observed reflections.

Data reduction, scaling and absorption corrections were performed using CrysAlisPro (Rigaku, V1.171.42.90a, 2023). The final completeness is 99.90 % out to 26.372° in Q. A multi-scan absorption correction was performed using CrysAlisPro 1.171.42.90a (Rigaku Oxford Diffraction, 2023) using spherical harmonics, implemented in SCALE3 ABSPACK scaling algorithm. The absorption coefficient m of this material is 0.535 mm^{-1} at this wavelength (l = 0.711Å) and the minimum and maximum transmissions are 0.825 and 1.000.

The structure was solved and the space group *P*1 (# 1) determined by the SHELXT structure solution program using intrinsic phasing methods and refined by Least Squares using version 2014/7 of ShelXL 2014/7 (Sheldrick, 2015). All non-hydrogen atoms were refined anisotropically. Hydrogen atom positions were calculated geometrically and refined using the riding model. Hydrogen atom positions were calculated geometrically and refined using the riding model.

_refine_special_details: Refined as a 2-component twin.

_exptl_absorpt_process_details: CrysAlisPro 1.171.42.90a (Rigaku Oxford Diffraction, 2023) using spherical harmonics,implemented in SCALE3 ABSPACK scaling algorithm.

1. Dolomanov, O.V., Bourhis, L.J., Gildea, R.J, Howard, J.A.K. & Puschmann, H. (2009), J. Appl. Cryst. 42, 339-341.

Crystal structure determination of 4

Crystal Data for $C_{33}H_{46}N_4ORu$ (*M* =615.81 g/mol): triclinic, space group P1 (no. 1), *a* = 11.37380(10) Å, *b* = 19.4936(2) Å, *c* = 28.0779(3) Å, *α* = 88.6070(10)°, *β* = 89.9770(10)°, *γ* = 89.7810(10)°, *V* = 6223.44(11) Å^3, *Z* = 8, *T* = 100.00 K, μ(Mo Kα) = 0.535 mm^{-1}, *Dcalc* = 1.314 g/cm^3, 253374 reflections measured (5.638° ≤ 2Θ ≤ 52.744°), 50364 unique (R_{int} = 0.0432, R_{sigma} = 0.0349) which were used in all calculations. The final R_1 was 0.0446 (I > 2σ(I)) and wR_2 was 0.1018 (all data).

Refinement model description

Number of restraints - 1707, number of constraints - unknown.

Details:

1. Twinned data refinement
 Scales: 0.74384
 0.25616
2. Uiso/Uaniso restraints and constraints
Uanis(C9E) ≈ Ueq, Uanis(C26D) ≈ Ueq, Uanis(C32D) ≈ Ueq, Uanis(C31D) ≈ Ueq, Uanis(C15D) ≈ Ueq, Uanis(C13D) ≈ Ueq, Uanis(C8B) ≈ Ueq, Uanis(C32B) ≈ Ueq, Uanis(C19B) ≈ Ueq, Uanis(C33B) ≈ Ueq, Uanis(C17B) ≈ Ueq, Uanis(C26B) ≈ Ueq, Uanis(C16F) ≈ Ueq: with sigma of 0.005 and sigma for terminal atoms of 0.005
Uanis(C25F) ≈ Ueq, Uanis(C25A) ≈ Ueq: with sigma of 0.001 and sigma for terminal atoms of 0.001
Uanis(C25E) ≈ Ueq: with sigma of 0.001 and sigma for terminal atoms of 0.001
Uanis(N1B) = Uanis(N2B)
3. Rigid body (RIGU) restrains
 C24C, C25C, C26C, C27C, C28C, C29C, C30C, C31C, C32C, C33C

with sigma for 1-2 distances of 0.004 and sigma for 1-3 distances of 0.004
N3C, N4C, C17C, C18C, C19C, C20C, C21C, C22C, C23C
with sigma for 1-2 distances of 0.004 and sigma for 1-3 distances of 0.004
C5C, C6C, C7C, C8C, C9C, C10C, C11C, C12C, C13C, C14C, C15C, C16C
with sigma for 1-2 distances of 0.004 and sigma for 1-3 distances of 0.004
C24D, C25D, C26D, C27D, C28D, C29D, C30D, C31D, C32D, C33D
with sigma for 1-2 distances of 0.004 and sigma for 1-3 distances of 0.004
C5D, C6D, C7D, C8D, C9D, C10D, C11D, C12D, C13D, C14D, C15D, C16D
with sigma for 1-2 distances of 0.004 and sigma for 1-3 distances of 0.004
C5A, C6A, C7A, C8A, C9A, C10A, C11A, C12A, C13A, C14A, C15A, C16A
with sigma for 1-2 distances of 0.004 and sigma for 1-3 distances of 0.004
N3A, N4A, C17A, C18A, C19A, C20A, C21A, C22A, C23A
with sigma for 1-2 distances of 0.004 and sigma for 1-3 distances of 0.004
C24A, C25A, C26A, C27A, C28A, C29A, C30A, C31A, C32A, C33A
with sigma for 1-2 distances of 0.004 and sigma for 1-3 distances of 0.004
C5B, C6B, C7B, C8B, C9B, C10B, C11B, C12B, C13B, C14B, C15B, C16B
with sigma for 1-2 distances of 0.004 and sigma for 1-3 distances of 0.004
C24B, C25B, C26B, C27B, C28B, C29B, C30B, C31B, C32B, C33B
with sigma for 1-2 distances of 0.004 and sigma for 1-3 distances of 0.004
C24E, C25E, C26E, C27E, C28E, C29E, C30E, C31E, C32E, C33E
with sigma for 1-2 distances of 0.004 and sigma for 1-3 distances of 0.004
C5E, C6E, C7E, C8E, C9E, C10E, C11E, C12E, C13E, C14E, C15E, C16E
with sigma for 1-2 distances of 0.004 and sigma for 1-3 distances of 0.004
N3E, N4E, C17E, C18E, C19E, C20E, C21E, C22E, C23E
with sigma for 1-2 distances of 0.004 and sigma for 1-3 distances of 0.004
C24G, C25G, C26G, C27G, C28G, C29G, C30G, C31G, C32G, C33G
with sigma for 1-2 distances of 0.004 and sigma for 1-3 distances of 0.004
C5G, C6G, C7G, C8G, C9G, C10G, C11G, C12G, C13G, C14G, C15G, C16G
with sigma for 1-2 distances of 0.004 and sigma for 1-3 distances of 0.004
O1G, C1G
with sigma for 1-2 distances of 0.004 and sigma for 1-3 distances of 0.004
C3E, N3G, N4G, C17G, C18G, C19G, C20G, C21G, C22G, C23G
with sigma for 1-2 distances of 0.004 and sigma for 1-3 distances of 0.004
C24F, C25F, C26F, C27F, C28F, C29F, C30F, C31F, C32F, C33F
with sigma for 1-2 distances of 0.004 and sigma for 1-3 distances of 0.004
C5F, C6F, C7F, C8F, C9F, C10F, C11F, C12F, C13F, C14F, C15F, C16F
with sigma for 1-2 distances of 0.004 and sigma for 1-3 distances of 0.004
O1F, C1F
with sigma for 1-2 distances of 0.004 and sigma for 1-3 distances of 0.004
C24H, C25H, C26H, C27H, C28H, C29H, C30H, C31H, C32H, C33H
with sigma for 1-2 distances of 0.004 and sigma for 1-3 distances of 0.004
N3H, N4H, C17H, C18H, C19H, C20H, C21H, C22H, C23H
with sigma for 1-2 distances of 0.004 and sigma for 1-3 distances of 0.004
C5H, C6H, C7H, C8H, C9H, C10H, C11H, C12H, C13H, C14H, C15H, C16H
with sigma for 1-2 distances of 0.004 and sigma for 1-3 distances of 0.004

4.a Ternary CH refined with riding coordinates:
C7A(H7A), C14A(H14A), C7B(H7B), C14B(H14B), C7C(H7C), C14C(H14C), C7D(H7D), C14D(H14D), C7E(H7E), C14E(H14E), C7F(H7F), C14F(H14F), C7G(H7G), C14G(H14G), C7H(H7H), C14H(H14H)
4.b Aromatic/amide H refined with riding coordinates:
C3A(H3A), C4A(H4A), C10A(H10A), C11A(H11A), C12A(H12A), C3B(H3B), C4B(H4B), C10B(H10B), C11B(H11B), C12B(H12B), C3C(H3C), C4C(H4C), C10C(H10C), C11C(H11C), C12C(H12C), C3D(H3D), C4D(H4D), C10D(H10D), C11D(H11D), C12D(H12D), C3E(H3E), C4E(H4E), C10E(H10E), C11E(H11E), C12E(H12E), C3F(H3F), C4F(H4F), C10F(H10F), C11F(H11F), C12F(H12F), C3G(H3G), C4G(H4G), C10G(H10G), C11G(H11G), C12G(H12G), C3H(H3H), C4H(H4H), C10H(H10H), C11H(H11H), C12H(H12H)
4.c Idealised Me refined as rotating group:
C8A(H8AA,H8AB,H8AC), C9A(H9AA,H9AB,H9AC), C15A(H15A,H15B,H15C), C16A(H16A, H16B,H16C), C18A(H18A,H18B,H18C), C20A(H20A,H20B,H20C), C22A(H22A,H22B,H22C), C23A(H23A,H23B,H23C), C25A(H25A,H25B,H25C), C27A(H27A,H27B,H27C), C29A(H29A, H29B,H29C), C31A(H31A,H31B,H31C), C33A(H33A,H33B,H33C), C8B(H8BA,H8BB,H8BC), C9B(H9BA,H9BB,H9BC), C15B(H15D,H15E,H15F), C16B(H16D,H16E,H16F), C18B(H18D, H18E,H18F), C20B(H20D,H20E,H20F), C22B(H22D,H22E,H22F), C23B(H23D,H23E,H23F), C25B(H25D,H25E,H25F), C27B(H27D,H27E,H27F), C29B(H29D,H29E,H29F), C31B(H31D, H31E,H31F), C33B(H33D,H33E,H33F), C8C(H8CA,H8CB,H8CC), C9C(H9CA,H9CB,H9CC), C15C(H15G,H15H,H15I), C16C(H16G,H16H,H16I), C18C(H18G,H18H,H18I), C20C(H20G, H20H,H20I), C22C(H22G,H22H,H22I), C23C(H23G,H23H,H23I), C25C(H25G,H25H,H25I), C27C(H27G,H27H,H27I), C29C(H29G,H29H,H29I), C31C(H31G,H31H,H31I), C33C(H33G, H33H,H33I), C8D(H8DA,H8DB,H8DC), C9D(H9DA,H9DB,H9DC), C15D(H15J,H15K,H15L), C16D(H16J,H16K,H16L), C18D(H18J,H18K,H18L), C20D(H20J,H20K,H20L), C22D(H22J, H22K,H22L), C23D(H23J,H23K,H23L), C25D(H25J,H25K,H25L), C27D(H27J,H27K,H27L), C29D(H29J,H29K,H29L), C31D(H31J,H31K,H31L), C33D(H33J,H33K,H33L), C8E(H8EA, H8EB,H8EC), C9E(H9EA,H9EB,H9EC), C15E(H15M,H15N,H15O), C16E(H16M,H16N,H16O), C18E(H18M,H18N,H18O), C20E(H20M,H20N,H20O), C22E(H22M,H22N,H22O), C23E(H23M, H23N,H23O), C25E(H25M,H25N,H25O), C27E(H27M,H27N,H27O), C29E(H29M,H29N,H29O), C31E(H31M,H31N,H31O), C33E(H33M,H33N,H33O), C8F(H8FA,H8FB,H8FC), C9F(H9FA,H9FB, H9FC), C15F(H15P,H15Q,H15R), C16F(H16P,H16Q,H16R), C18F(H18P,H18Q,H18R), C20F(H20P,H20Q,H20R), C22F(H22P,H22Q,H22R), C23F(H23P,H23Q,H23R), C25F(H25P, H25Q,H25R), C27F(H27P,H27Q,H27R), C29F(H29P,H29Q,H29R), C31F(H31P,H31Q,H31R), C33F(H33P,H33Q,H33R), C8G(H8GA,H8GB,H8GC), C9G(H9GA,H9GB,H9GC), C15G(H15S,H15T, H15U), C16G(H16S,H16T,H16U), C18G(H18S,H18T,H18U), C20G(H20S,H20T,H20U), C22G(H22S,H22T,H22U), C23G(H23S,H23T,H23U), C25G(H25S,H25T,H25U), C27G(H27S, H27T,H27U), C29G(H29S,H29T,H29U), C31G(H31S,H31T,H31U), C33G(H33S,H33T,H33U), C8H(H8HA,H8HB,H8HC), C9H(H9HA,H9HB,H9HC), C15H(H15V,H15W,H15X), C16H(H16V,H16W, H16X), C18H(H18V,H18W,H18X), C20H(H20V,H20W,H20X), C22H(H22V,H22W,H22X), C23H(H23V,H23W,H23X), C25H(H25V,H25W,H25X), C27H(H27V,H27W,H27X), C29H(H29V, H29W,H29X), C31H(H31V,H31W,H31X), C33H(H33V,H33W,H33X)

DippP=(IMe_4) (5)

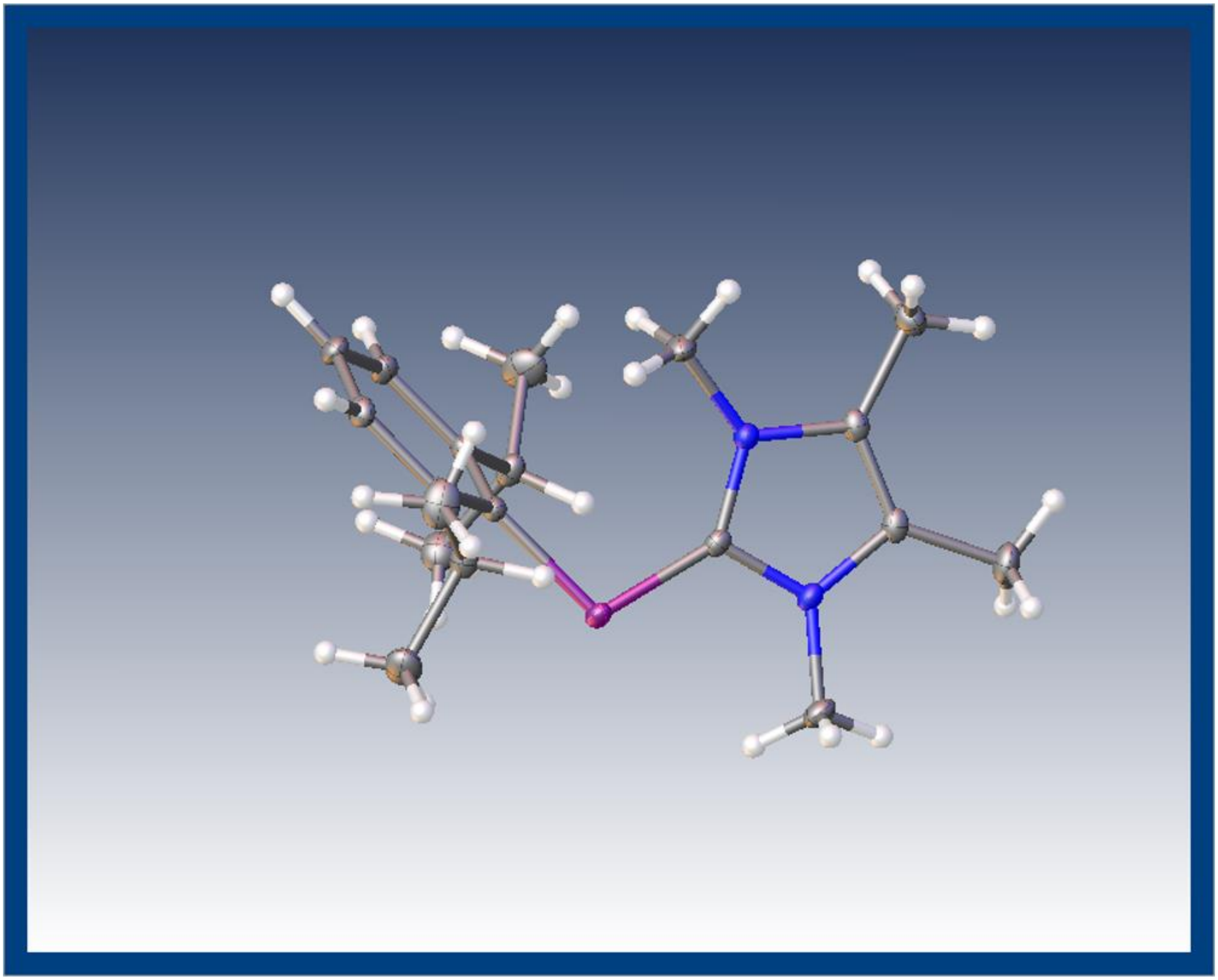

Table S24 Crystal data and structure refinement for 5.

Identification code	TSD65_Tilley
Empirical formula	$C_{19}H_{29}N_2P$
Formula weight	316.41
Temperature/K	100
Crystal system	monoclinic

Space group	$P2_1/n$
a/Å	14.2059(7)
b/Å	9.7392(4)
c/Å	14.5362(7)
α/°	90
β/°	115.659(6)
γ/°	90
Volume/Å^3	1812.82(17)
Z	4
ρ_{calc}g/cm^3	1.159
μ/mm^{-1}	0.151
F(000)	688.0
Crystal size/mm^3	0.22 × 0.19 × 0.12
Radiation	Mo Kα (λ = 0.71073)
2Θ range for data collection/°	6.218 to 56.558
Index ranges	-18 ≤ h ≤ 18, -12 ≤ k ≤ 12, -19 ≤ l ≤ 19
Reflections collected	28672
Independent reflections	4489 [R_{int} = 0.0437, R_{sigma} = 0.0291]
Data/restraints/parameters	4489/0/207
Goodness-of-fit on F^2	1.068
Final R indexes [I>=2σ (I)]	R_1 = 0.0329, wR_2 = 0.0835
Final R indexes [all data]	R_1 = 0.0402, wR_2 = 0.0874
Largest diff. peak/hole / e Å^{-3}	0.31/-0.23

Table S25 Fractional Atomic Coordinates (×10^4) and Equivalent Isotropic Displacement Parameters (Å^2×10^3) for 5. U_{eq} is defined as 1/3 of the trace of the orthogonalised U_{IJ} tensor.

**Atom**	***x***	***y***	***z***	**U(eq)**
P1	3393.6(2)	3446.5(3)	5654.5(2)	14.18(8)
N1	4048.5(7)	6058.4(9)	5561.4(6)	13.53(18)
N2	2353.8(7)	6062.2(9)	4903.6(7)	13.39(18)
C7	3204.9(8)	5214.0(11)	5337.4(7)	12.5(2)
C2	3730.2(8)	7410.7(11)	5282.2(8)	15.1(2)
C8	2061.9(8)	2723.5(10)	5014.9(8)	12.4(2)
C16	1611.6(8)	2143.8(10)	5628.0(8)	13.2(2)
C9	1498.6(8)	2658.9(11)	3937.1(8)	13.2(2)
C4	2678.7(8)	7418.1(11)	4863.5(8)	14.8(2)
C15	606.0(8)	1596.4(11)	5162.3(8)	15.8(2)
C13	493.0(8)	2101.4(11)	3502.7(8)	15.8(2)
C14	45.3(8)	1591.7(11)	4108.9(8)	17.1(2)
C10	1970.0(8)	3138.4(12)	3229.5(8)	16.2(2)
C1	5120.6(8)	5606.7(12)	6142.9(8)	18.3(2)
C17	2214.1(9)	2088.5(12)	6786.1(8)	17.6(2)
C6	1284.7(8)	5710.2(12)	4705.6(9)	20.1(2)
C5	1923.7(9)	8572.1(12)	4433.5(9)	21.5(2)
C3	4493.3(9)	8544.8(12)	5479.6(9)	21.9(2)
C11	2499.9(10)	1918.6(13)	2979.0(9)	23.9(2)
C19	2957.7(10)	853.9(13)	7098.7(9)	24.9(3)
C18	1523.5(11)	2059.9(14)	7354.3(9)	27.7(3)
C12	1176.6(10)	3796.8(15)	2245.1(9)	28.5(3)

Table 26 Anisotropic Displacement Parameters (Å^2×10^3) for 5. The Anisotropic displacement factor exponent takes the form: $-2\pi^2[h^2a^{*2}U_{11}+2hka^*b^*U_{12}+\ldots]$.

Atom	U_{11}	U_{22}	U_{33}	U_{23}	U_{13}	U_{12}
P1	10.42(13)	13.02(14)	15.13(14)	2.0(1)	1.82(10)	-1.28(10)
N1	11.3(4)	14.3(4)	14.1(4)	-1.1(3)	4.7(3)	-3.1(3)
N2	12.0(4)	11.7(4)	15.4(4)	-0.4(3)	5.1(3)	-1.0(3)
C7	11.3(4)	14.9(5)	10.9(4)	-1.2(4)	4.2(4)	-2.4(4)
C2	19.4(5)	14.0(5)	13.3(5)	-1.2(4)	8.3(4)	-3.4(4)
C8	11.2(4)	10.2(5)	14.5(5)	0.6(4)	4.3(4)	-0.1(4)
C16	15.6(5)	9.9(5)	13.9(5)	0.0(4)	6.3(4)	0.6(4)
C9	13.7(5)	10.0(5)	14.7(5)	1.6(4)	5.1(4)	0.6(4)
C4	19.2(5)	12.7(5)	13.8(5)	-0.2(4)	8.3(4)	-1.3(4)
C15	16.4(5)	13.3(5)	19.8(5)	2.0(4)	9.9(4)	-1.3(4)
C13	14.2(5)	14.7(5)	14.4(5)	0.2(4)	2.5(4)	-1.2(4)
C14	11.8(5)	15.7(5)	21.0(5)	-0.5(4)	4.4(4)	-2.9(4)
C10	16.2(5)	17.7(5)	13.2(5)	1.1(4)	4.9(4)	-3.8(4)
C1	11.3(5)	21.5(6)	19.1(5)	-1.5(4)	3.7(4)	-3.1(4)
C17	22.6(5)	16.6(5)	12.7(5)	0.0(4)	6.8(4)	-4.5(4)
C6	12.1(5)	16.0(5)	31.2(6)	-1.1(4)	8.3(4)	0.3(4)
C5	23.6(6)	15.6(5)	25.6(6)	2.6(4)	10.9(5)	2.2(4)
C3	23.6(6)	17.7(6)	24.4(6)	-2.4(4)	10.4(5)	-8.6(4)
C11	24.9(6)	25.8(6)	25.8(6)	-2.1(5)	15.5(5)	-2.0(5)
C19	26.5(6)	22.7(6)	18.6(5)	6.0(4)	3.2(5)	0.4(5)
C18	37.5(7)	30.9(7)	19.6(6)	-3.1(5)	17.2(5)	-7.8(6)
C12	27.8(6)	34.9(7)	20.1(6)	13.3(5)	7.9(5)	0.0(5)

Table 26 Bond Lengths for 5.

Atom	Atom	Length/Å	Atom	Atom	Length/Å
P1	C7	1.7722(11)	C16	C15	1.3948(14)
P1	C8	1.8482(10)	C16	C17	1.5237(14)
N1	C7	1.3713(13)	C9	C13	1.3974(14)
N1	C2	1.3945(14)	C9	C10	1.5236(14)
N1	C1	1.4535(13)	C4	C5	1.4912(15)
N2	C7	1.3717(13)	C15	C14	1.3867(15)
N2	C4	1.4084(14)	C13	C14	1.3830(15)
N2	C6	1.4589(13)	C10	C11	1.5323(16)
C2	C4	1.3470(15)	C10	C12	1.5272(15)
C2	C3	1.4859(15)	C17	C19	1.5338(17)
C8	C16	1.4198(14)	C17	C18	1.5321(16)
C8	C9	1.4189(14)			

Table 27 Bond Angles for 5.

Atom	Atom	Atom	Angle/˚	Atom	Atom	Atom	Angle/˚
C7	P1	C8	103.45(5)	C15	C16	C8	119.50(9)
C7	N1	C2	110.94(9)	C15	C16	C17	119.34(9)
C7	N1	C1	122.99(9)	C8	C9	C10	121.96(9)
C2	N1	C1	125.53(9)	C13	C9	C8	119.58(9)
C7	N2	C4	110.14(8)	C13	C9	C10	118.44(9)
C7	N2	C6	125.75(9)	N2	C4	C5	122.34(9)
C4	N2	C6	122.95(9)	C2	C4	N2	107.18(9)
N1	C7	P1	120.17(8)	C2	C4	C5	130.47(10)
N1	C7	N2	104.69(9)	C14	C15	C16	121.00(10)
N2	C7	P1	135.11(8)	C14	C13	C9	120.91(10)

Table 27 Bond Angles for 5.

Atom	Atom	Atom	Angle/˚	Atom	Atom	Atom	Angle/˚
N1	C2	C3	121.89(10)	C13	C14	C15	119.97(10)
C4	C2	N1	107.03(9)	C9	C10	C11	109.12(9)
C4	C2	C3	131.06(11)	C9	C10	C12	113.54(9)
C16	C8	P1	118.49(7)	C12	C10	C11	110.01(10)
C9	C8	P1	122.51(8)	C16	C17	C19	109.90(9)
C9	C8	C16	118.91(9)	C16	C17	C18	114.31(10)
C8	C16	C17	121.15(9)	C18	C17	C19	110.23(10)

Table 28 Torsion Angles for 5.

A	B	C	D	Angle/˚	A	B	C	D	Angle/˚
P1	C8	C16	C15	179.78(8)	C16	C8	C9	C10	-174.28(9)
P1	C8	C16	C17	-1.19(14)	C16	C15	C14	C13	1.76(17)
P1	C8	C9	C13	-179.88(8)	C9	C8	C16	C15	-3.63(15)
P1	C8	C9	C10	2.17(14)	C9	C8	C16	C17	175.40(10)
N1	C2	C4	N2	0.97(11)	C9	C13	C14	C15	-1.72(17)
N1	C2	C4	C5	179.98(11)	C4	N2	C7	P1	178.19(9)
C7	P1	C8	C16	-116.40(8)	C4	N2	C7	N1	0.02(11)
C7	P1	C8	C9	67.14(9)	C15	C16	C17	C19	97.47(12)
C7	N1	C2	C4	-1.01(12)	C15	C16	C17	C18	-27.07(15)
C7	N1	C2	C3	177.54(9)	C13	C9	C10	C11	-86.32(12)
C7	N2	C4	C2	-0.64(11)	C13	C9	C10	C12	36.78(14)
C7	N2	C4	C5	-179.75(10)	C10	C9	C13	C14	177.00(10)
C2	N1	C7	P1	-177.91(7)	C1	N1	C7	P1	-5.94(13)
C2	N1	C7	N2	0.59(11)	C1	N1	C7	N2	172.56(9)
C8	P1	C7	N1	-170.76(8)	C1	N1	C2	C4	-172.73(9)

Table 28 Torsion Angles for 5.

A	B	C	D	Angle/˚	A	B	C	D	Angle/˚
C8	P1	C7	N2	11.29(12)	C1	N1	C2	C3	5.82(16)
C8	C16	C15	C14	0.94(16)	C17	C16	C15	C14	-178.10(10)
C8	C16	C17	C19	-81.56(12)	C6	N2	C7	P1	10.27(17)
C8	C16	C17	C18	153.90(10)	C6	N2	C7	N1	-167.90(10)
C8	C9	C13	C14	-1.03(16)	C6	N2	C4	C2	167.68(10)
C8	C9	C10	C11	91.66(12)	C6	N2	C4	C5	-11.42(15)
C8	C9	C10	C12	-145.25(11)	C3	C2	C4	N2	-177.39(11)
C16	C8	C9	C13	3.68(15)	C3	C2	C4	C5	1.6(2)

Table 29 Hydrogen Atom Coordinates (Å×10^4) and Isotropic Displacement Parameters (Å^2×10^3) for 5.

Atom	*x*	*y*	*z*	U(eq)
H15	300.67	1221.01	5572.95	19
H13	111.75	2072.16	2781.09	19
H14	-646.1	1238.57	3803.7	21
H10	2519.11	3837.23	3603.46	19
H1A	5290.32	5609.34	6872.14	27
H1B	5592.95	6230.93	6016.58	27
H1C	5201.29	4675.24	5932.13	27
H17	2652.95	2936.32	7006.27	21
H6A	919.31	5336.73	4014.01	30
H6B	921.96	6535.41	4768.16	30
H6C	1294.74	5021.96	5201.57	30
H5A	1403.69	8329.28	3746.27	32
H5B	2299	9400.33	4399.14	32

Table 29 Hydrogen Atom Coordinates (Å×10^4) and Isotropic Displacement Parameters (Å^2×10^3) for 5.

Atom	*x*	*y*	*z*	U(eq)
H5C	1572.27	8745.1	4872.31	32
H3A	4930.55	8623.16	6215.48	33
H3B	4117.46	9408.83	5221.84	33
H3C	4935.15	8350.23	5131.8	33
H11A	3042.62	1543.65	3612.26	36
H11B	2816.84	2225.21	2534.9	36
H11C	1979.21	1206.68	2629.76	36
H19A	2551.77	3.55	6881.8	37
H19B	3364.65	847.55	7841.85	37
H19C	3431.9	921.77	6772.1	37
H18A	1002.06	2794.27	7095.17	41
H18B	1957.47	2197	8085.63	41
H18C	1169.08	1170.18	7245.61	41
H12A	659.02	3109.52	1840.2	43
H12B	1537.07	4147.82	1851.43	43
H12C	824.61	4555.72	2412.17	43

Experimental

Single crystals of $C_{19}H_{29}N_2P$ **5** were grown from toluene at – 30 °C. A suitable crystal was selected and mounted on a **ROD, Synergy Custom DW system, Pilatus 200K** diffractometer. The crystal was kept at 100 K during data collection. Using Olex2 [1], the structure was solved.

1. Dolomanov, O.V., Bourhis, L.J., Gildea, R.J, Howard, J.A.K. & Puschmann, H. (2009), J. Appl. Cryst. 42, 339-341.

Crystal structure determination of 5.

Crystal Data for $C_{19}H_{29}N_2P$ (M =316.41 g/mol): monoclinic, space group P2$_1$/n (no. 14), a = 14.2059(7) Å, b = 9.7392(4) Å, c = 14.5362(7) Å, β = 115.659(6)°, V = 1812.82(17) Å^3, Z = 4, T = 100 K, μ(Mo Kα) = 0.151 mm^{-1}, $Dcalc$ = 1.159 g/cm^3, 28672 reflections measured (6.218° ≤ 2Θ ≤ 56.558°), 4489 unique (R_{int} = 0.0437, R_{sigma} = 0.0291) which were used in all calculations. The final R_1 was 0.0329 (I > 2σ(I)) and wR_2 was 0.0874 (all data).

Refinement model description

Number of restraints - 0, number of constraints - unknown.

Details:

1.a Ternary CH refined with riding coordinates:
C10(H10), C17(H17)
1.b Aromatic/amide H refined with riding coordinates:
C15(H15), C13(H13), C14(H14)
1.c Idealized Me refined as rotating group:
C1(H1A,H1B,H1C), C6(H6A,H6B,H6C), C5(H5A,H5B,H5C), C3(H3A,H3B,H3C), C11(H11A,H11B,H11C), C19(H19A,H19B,H19C), C18(H18A,H18B,H18C), C12(H12A,H12B,H12C)

Iminophosphanide (6)

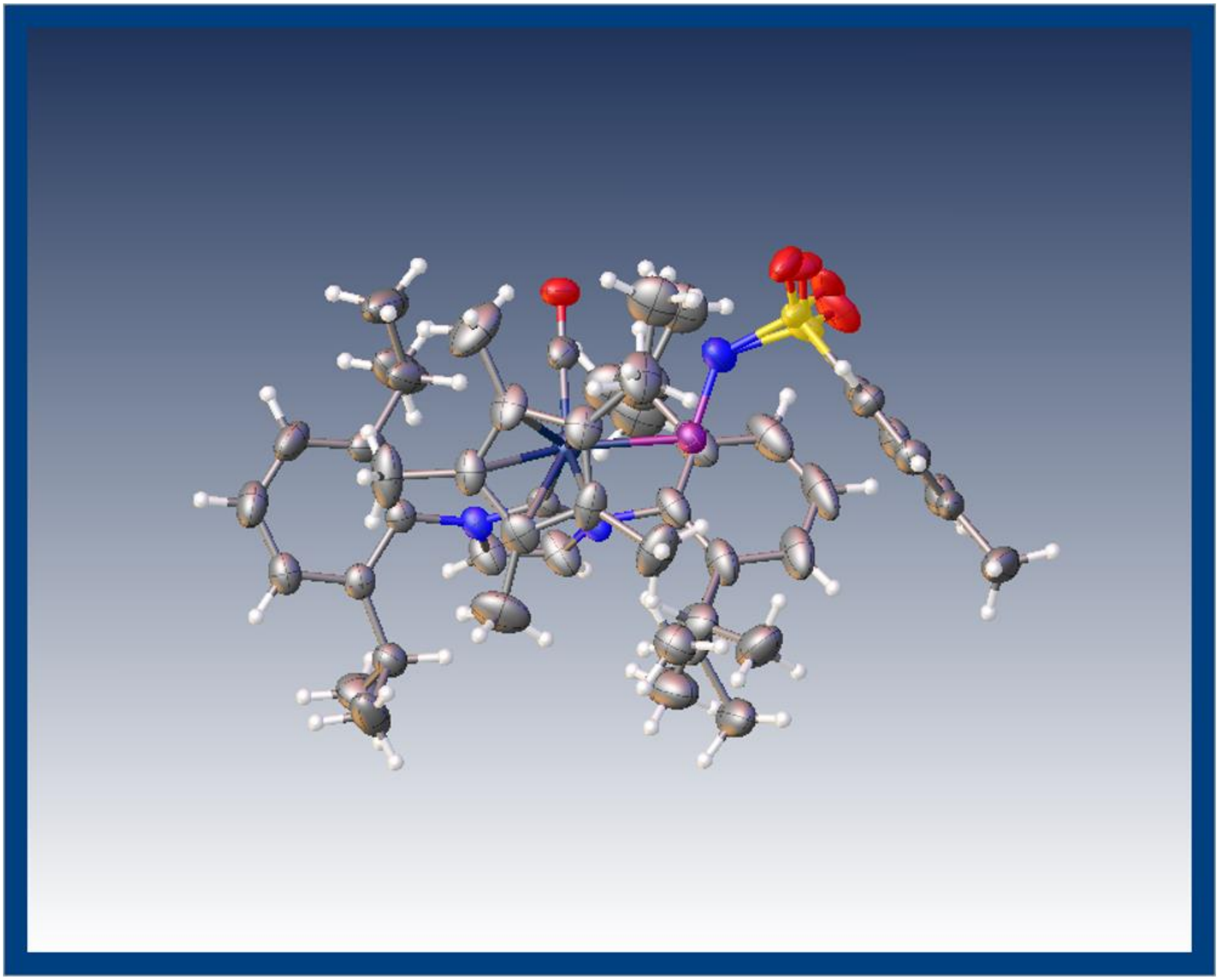

Table 1 Crystal data and structure refinement for 6.

Identification code	TSD49B_Tilley
Empirical formula	$C_{45}H_{58}N_3O_3PRuS$
Formula weight	853.04
Temperature/K	100
Crystal system	monoclinic
Space group	$P2_1/c$
a/Å	11.84530(10)
b/Å	15.3524(2)

c/Å	23.2358(3)
α/°	90
β/°	91.4090(10)
γ/°	90
Volume/Å^3	4224.24(9)
Z	4
ρ_{calc}g/cm^3	1.341
μ/mm^{-1}	4.154
F(000)	1792.0
Crystal size/mm^3	0.14 × 0.08 × 0.05
Radiation	Cu Kα (λ = 1.54184)
2Θ range for data collection/°	6.902 to 149.002
Index ranges	-14 ≤ h ≤ 14, -19 ≤ k ≤ 19, -26 ≤ l ≤ 29
Reflections collected	60880
Independent reflections	8602 [R_{int} = 0.0513, R_{sigma} = 0.0275]
Data/restraints/parameters	8602/123/564
Goodness-of-fit on F^2	1.045
Final R indexes [I>=2σ (I)]	R_1 = 0.0509, wR_2 = 0.1258
Final R indexes [all data]	R_1 = 0.0563, wR_2 = 0.1290
Largest diff. peak/hole / e Å^{-3}	0.75/-1.26

Table S30 Fractional Atomic Coordinates (×10^4) and Equivalent Isotropic Displacement Parameters (Å^2×10^3) for 6. U_{eq} is defined as 1/3 of the trace of the orthogonalised U_{IJ} tensor.

Atom	*x*	*y*	*z*	U(eq)
Ru1	7611.6(2)	6026.6(2)	7755.9(2)	30.72(9)
P1	7154.5(7)	5911.2(6)	6816.1(4)	34.34(19)

Table S30 Fractional Atomic Coordinates (×10^4) and Equivalent Isotropic Displacement Parameters (Å^2×10^3) for 6. U_{eq} is defined as 1/3 of the trace of the orthogonalised U_{IJ} tensor.

Atom	*x*	*y*	*z*	U(eq)
S2	5869(7)	6798(10)	5949(5)	44.4(17)
N3	6733(2)	4532.7(19)	8604.3(12)	33.2(6)
N2	6222(3)	4285(2)	7729.3(14)	41.4(7)
O1	5556(3)	7148.8(19)	7852.8(12)	51.9(7)
C12	6831(3)	4865(2)	8058.3(15)	30.9(7)
C27	5890(3)	4306(3)	7130.3(18)	46.3(10)
C21	7945(3)	4333(2)	9464.0(15)	36.4(7)
O2A	6030(20)	7723(11)	5900(10)	58(4)
C13	7159(3)	4850(2)	9156.3(15)	34.4(7)
C39	6832(3)	6369(3)	5495.2(15)	41.6(8)
N1	6172(3)	6570(2)	6613.8(14)	47.9(8)
C25	6073(3)	3780(3)	8602.8(18)	48.5(10)
O3A	4767(10)	6480(17)	5774(9)	69(5)
C40	6532(4)	5564(3)	5279.9(18)	52.6(10)
C44	7834(3)	6750(3)	5347.7(17)	45.3(9)
C20	8287(4)	4626(3)	10010.0(17)	47.1(9)
C3	9480(3)	5755(3)	8033.0(19)	47.1(9)
C43	8546(4)	6317(3)	4982.1(18)	50.1(10)
C26	5760(4)	3622(3)	8062(2)	54.5(11)
C11	6306(4)	6678(2)	7823.6(16)	41.9(8)
C22	8375(3)	3456(3)	9260.0(18)	45.7(9)
C5	8955(4)	6962(3)	7492.7(18)	48.9(10)
C2	9065(3)	6409(3)	8405.2(17)	46.3(9)
C42	8241(4)	5495(3)	4757.8(16)	54.5(12)

Table S30 Fractional Atomic Coordinates (×10^4) and Equivalent Isotropic Displacement Parameters (Å^2×10^3) for 6. U_{eq} is defined as 1/3 of the trace of the orthogonalised U_{IJ} tensor.

Atom	*x*	*y*	*z*	U(eq)
C35	6573(4)	3885(3)	6736.2(19)	56.0(11)
C41	7232(4)	5135(3)	4919.1(18)	55.3(11)
C28	4814(4)	4655(3)	6991(2)	60.8(13)
C14	6648(4)	5574(3)	9401.4(17)	45.6(9)
C4	9462(3)	6123(3)	7461.2(17)	49.3(11)
C18	7013(5)	5827(3)	9952.4(19)	58.8(12)
C23	9657(4)	3341(4)	9347(2)	74.4(17)
C1	8715(4)	7132(3)	8077.4(19)	53.7(11)
C19	7835(5)	5366(3)	10246.6(19)	58.8(12)
C32	4397(5)	4524(4)	6444(3)	73.8(17)
C34	6116(5)	3781(3)	6180(2)	68.8(15)
C10	8868(5)	7609(4)	7009(2)	72.4(15)
C9	9949(4)	5748(4)	6937(2)	71.8(15)
C15	5642(4)	6021(3)	9115(2)	60.4(13)
C33	5029(6)	4089(4)	6048(3)	80(2)
C17	4534(4)	5561(4)	9282(3)	82.3(19)
C24	7794(5)	2716(3)	9582(2)	67.2(13)
C29	4121(4)	5138(4)	7423(3)	84.2(14)
C7	9186(4)	6419(5)	9049(2)	77.4(17)
C36	7780(6)	3646(5)	6875(4)	60(2)
C8	9964(4)	4906(4)	8211(3)	82.1(18)
C6	8257(6)	7985(4)	8316(3)	92(2)
C45	9006(6)	5044(4)	4347(2)	77.5(17)
C16	5589(6)	6985(4)	9232(3)	95(2)

Table S30 Fractional Atomic Coordinates (×10^4) and Equivalent Isotropic Displacement Parameters (Å^2×10^3) for 6. U_{eq} is defined as 1/3 of the trace of the orthogonalised U_{IJ} tensor.

Atom	*x*	*y*	*z*	U(eq)
C31A	3630(20)	6050(11)	7296(15)	84.2(14)
C37	8508(7)	3686(6)	6354(4)	82(2)
C30A	3340(20)	4348(14)	7545(14)	84.2(14)
C38	7766(9)	2736(6)	7136(5)	94(3)
S1	5973(15)	7023(12)	5995(5)	53(2)
O2	6350(30)	7914(13)	5996(10)	66(6)
O3	4821(17)	6851(19)	5825(8)	70(5)
C37A	8694(15)	3811(12)	6988(9)	57(5)
C38A	7911(19)	2389(12)	6647(9)	66(6)
C36A	7598(13)	3272(11)	6935(9)	48(5)
C30	3217(6)	4621(6)	7748(4)	84.2(14)
C31	3526(7)	5904(6)	7111(5)	84.2(14)

Table S31 Anisotropic Displacement Parameters (Å^2×10^3) for 6. The Anisotropic displacement factor exponent takes the form: $-2\pi^2[h^2a^{*2}U_{11}+2hka^*b^*U_{12}+…]$.

Atom	U_{11}	U_{22}	U_{33}	U_{23}	U_{13}	U_{12}
Ru1	31.69(15)	30.24(14)	30.29(15)	1.61(9)	2.3(1)	-4.41(10)
P1	32.4(4)	37.4(5)	33.1(4)	3.1(3)	-0.7(3)	0.4(3)
S2	40(2)	48(4)	45(3)	14(2)	4.2(13)	18.4(19)
N3	28.0(13)	36.3(15)	35.1(15)	6.2(12)	-0.6(11)	-6.2(11)
N2	40.7(17)	40.0(16)	42.9(17)	8.5(13)	-11.3(14)	-14.5(13)
O1	59.9(18)	46.8(16)	49.5(16)	9.6(12)	13.4(13)	23.0(14)
C12	22.3(14)	33.0(16)	37.4(17)	4.0(13)	-2.0(13)	-4.2(12)
C27	46(2)	47(2)	45(2)	10.5(17)	-16.6(18)	-17.3(18)
C21	29.1(16)	45(2)	35.3(18)	1.8(15)	3.3(14)	-5.2(14)

Table S31 Anisotropic Displacement Parameters (Å^2×10^3) for 6. The Anisotropic displacement factor exponent takes the form: -2π^2[h^{2}a*^{2}U$_{11}$+2hka*b*U$_{12}$+…].

Atom	U_{11}	U_{22}	U_{33}	U_{23}	U_{13}	U_{12}
O2A	93(10)	33(6)	49(8)	14(5)	27(6)	25(5)
C13	30.4(17)	40.2(18)	32.7(17)	2.3(14)	4.6(13)	-3.4(14)
C39	38.8(19)	54(2)	31.8(18)	2.9(16)	-6.1(15)	12.3(17)
N1	49.1(19)	56(2)	39.0(17)	10.2(15)	4.4(14)	11.9(16)
C25	47(2)	49(2)	50(2)	13.7(18)	-6.2(18)	-21.2(18)
O3A	33(4)	104(11)	70(7)	31(8)	-8(4)	14(5)
C40	53(2)	65(3)	39(2)	5.3(19)	-11.8(18)	-3(2)
C44	48(2)	45(2)	42(2)	-6.6(16)	-3.0(17)	8.2(17)
C20	46(2)	58(2)	38(2)	3.7(17)	-2.4(16)	-9.0(18)
C3	21.7(16)	62(2)	57(2)	4(2)	-3.0(16)	-8.8(16)
C43	45(2)	60(3)	46(2)	3.0(19)	4.2(18)	11.1(19)
C26	52(2)	54(2)	57(3)	17(2)	-14(2)	-26(2)
C11	55(2)	35.2(18)	35.5(19)	6.6(15)	7.0(16)	-0.7(17)
C22	44(2)	50(2)	43(2)	2.7(17)	0.6(17)	5.9(17)
C5	50(2)	48(2)	49(2)	3.6(18)	9.3(18)	-23.9(19)
C2	40(2)	62(3)	36(2)	-2.0(17)	-2.5(16)	-22.7(18)
C42	80(3)	55(2)	27.6(18)	-1.1(17)	-3.1(19)	32(2)
C35	62(3)	61(3)	44(2)	5(2)	-20(2)	-21(2)
C41	76(3)	53(2)	37(2)	-1.4(18)	-11(2)	-2(2)
C28	53(3)	55(3)	73(3)	20(2)	-25(2)	-24(2)
C14	50(2)	44(2)	44(2)	8.3(17)	18.4(17)	5.7(17)
C4	27.1(17)	82(3)	39(2)	-14.0(19)	8.4(15)	-18.6(19)
C18	88(4)	49(2)	41(2)	-4.8(18)	24(2)	4(2)
C23	52(3)	120(5)	52(3)	18(3)	5(2)	35(3)

Table S31 Anisotropic Displacement Parameters (Å^2×10^3) for 6. The Anisotropic displacement factor exponent takes the form: $-2\pi^2[h^2a^{*2}U_{11}+2hka^*b^*U_{12}+\ldots]$.

Atom	U_{11}	U_{22}	U_{33}	U_{23}	U_{13}	U_{12}
C1	66(3)	44(2)	51(2)	-12.1(18)	2(2)	-29(2)
C19	83(3)	58(3)	36(2)	-6.1(19)	2(2)	-14(2)
C32	73(3)	61(3)	85(4)	18(3)	-44(3)	-22(3)
C34	96(4)	61(3)	48(3)	0(2)	-25(3)	-32(3)
C10	91(4)	64(3)	62(3)	14(2)	9(3)	-30(3)
C9	45(3)	105(4)	66(3)	-31(3)	15(2)	-11(3)
C15	69(3)	62(3)	52(3)	21(2)	30(2)	30(2)
C33	107(5)	66(3)	63(3)	21(3)	-53(3)	-39(3)
C17	58(3)	113(5)	77(4)	40(3)	27(3)	34(3)
C24	84(4)	44(2)	73(3)	10(2)	-4(3)	1(2)
C29	40.2(17)	102(3)	110(4)	4(3)	1(2)	-6.7(19)
C7	59(3)	134(5)	39(2)	-4(3)	-4(2)	-38(3)
C36	61(5)	49(4)	68(4)	-17(4)	-14(3)	-4(3)
C8	39(2)	84(4)	123(5)	20(4)	-8(3)	11(2)
C6	117(5)	64(3)	97(4)	-35(3)	29(4)	-27(3)
C45	111(5)	74(3)	48(3)	1(2)	16(3)	42(3)
C16	144(6)	72(4)	69(4)	12(3)	36(4)	53(4)
C31A	40.2(17)	102(3)	110(4)	4(3)	1(2)	-6.7(19)
C37	76(5)	86(5)	83(5)	-30(4)	1(4)	18(4)
C30A	40.2(17)	102(3)	110(4)	4(3)	1(2)	-6.7(19)
C38	108(7)	60(5)	112(7)	0(4)	-34(5)	14(4)
S1	79(4)	50(5)	28.9(18)	3(3)	9(2)	36(3)
O2	119(17)	44(7)	36(6)	-2(5)	18(7)	24(7)
O3	58(7)	112(13)	39(5)	6(8)	0(5)	63(8)

Table S31 Anisotropic Displacement Parameters (Å^2×10^3) for 6. The Anisotropic displacement factor exponent takes the form: -2π^2[h^{2}a*^{2}U$_{11}$+2hka*b*U$_{12}$+…].

Atom	U11	U22	U33	U23	U13	U12
C37A	49(9)	54(9)	66(12)	2(8)	-6(8)	-7(7)
C38A	77(13)	56(10)	64(12)	-16(9)	-21(10)	9(9)
C36A	46(9)	52(11)	45(9)	-14(9)	2(7)	-21(8)
C30	40.2(17)	102(3)	110(4)	4(3)	1(2)	-6.7(19)
C31	40.2(17)	102(3)	110(4)	4(3)	1(2)	-6.7(19)

Table S32 Bond Lengths for 6.

Atom	Atom	Length/Å	Atom	Atom	Length/Å
Ru1	P1	2.2440(9)	C3	C8	1.480(7)
Ru1	C12	2.135(3)	C43	C42	1.410(7)
Ru1	C3	2.327(4)	C22	C23	1.537(6)
Ru1	C11	1.852(4)	C22	C24	1.533(6)
Ru1	C5	2.239(4)	C5	C4	1.423(6)
Ru1	C2	2.336(4)	C5	C1	1.419(6)
Ru1	C4	2.317(4)	C5	C10	1.502(6)
Ru1	C1	2.259(4)	C2	C1	1.404(6)
P1	N1	1.604(3)	C2	C7	1.499(6)
S2	O2A	1.438(10)	C42	C41	1.377(7)
S2	C39	1.705(13)	C42	C45	1.500(6)
S2	N1	1.615(11)	C35	C34	1.398(6)
S2	O3A	1.443(10)	C35	C36	1.503(8)
N3	C12	1.375(4)	C35	C36A	1.596(16)
N3	C13	1.451(4)	C28	C32	1.368(7)
N3	C25	1.396(5)	C28	C29	1.506(9)

Table S32 Bond Lengths for 6.

Atom	Atom	Length/Å	Atom	Atom	Length/Å
N2	C12	1.368(4)	C14	C18	1.396(6)
N2	C27	1.437(5)	C14	C15	1.515(6)
N2	C26	1.397(5)	C4	C9	1.477(6)
O1	C11	1.147(5)	C18	C19	1.373(7)
C27	C35	1.395(7)	C1	C6	1.526(7)
C27	C28	1.412(6)	C32	C33	1.373(9)
C21	C13	1.405(5)	C34	C33	1.399(9)
C21	C20	1.397(5)	C15	C17	1.548(7)
C21	C22	1.520(6)	C15	C16	1.507(7)
C13	C14	1.394(5)	C29	C31A	1.542(12)
C39	C40	1.376(6)	C29	C30A	1.554(12)
C39	C44	1.374(6)	C29	C30	1.545(8)
C39	S1	1.857(11)	C29	C31	1.542(8)
N1	S1	1.610(12)	C36	C37	1.506(12)
C25	C26	1.325(6)	C36	C38	1.523(11)
C40	C41	1.363(7)	S1	O2	1.438(12)
C44	C43	1.383(6)	S1	O3	1.436(12)
C20	C19	1.376(6)	C37A	C36A	1.542(16)
C3	C2	1.420(6)	C38A	C36A	1.561(16)
C3	C4	1.443(6)			

Table S33 Bond Angles for 6.

Atom	Atom	Atom	Angle/˚	Atom	Atom	Atom	Angle/˚
P1	Ru1	C3	117.40(11)	C44	C43	C42	120.2(4)
P1	Ru1	C2	143.59(10)	C25	C26	N2	106.7(3)

Table S33 Bond Angles for 6.

Atom	Atom	Atom	Angle/˚	Atom	Atom	Atom	Angle/˚
P1	Ru1	C4	85.69(10)	O1	C11	Ru1	173.6(3)
P1	Ru1	C1	120.34(12)	C21	C22	C23	113.5(4)
C12	Ru1	P1	99.10(9)	C21	C22	C24	110.3(3)
C12	Ru1	C3	100.14(14)	C24	C22	C23	107.7(4)
C12	Ru1	C5	160.30(15)	C4	C5	Ru1	74.8(2)
C12	Ru1	C2	108.39(13)	C4	C5	C10	125.6(4)
C12	Ru1	C4	124.82(15)	C1	C5	Ru1	72.3(2)
C12	Ru1	C1	140.41(15)	C1	C5	C4	108.1(4)
C3	Ru1	C2	35.46(16)	C1	C5	C10	125.6(4)
C11	Ru1	P1	86.75(12)	C10	C5	Ru1	126.3(3)
C11	Ru1	C12	93.09(15)	C3	C2	Ru1	71.9(2)
C11	Ru1	C3	149.74(17)	C3	C2	C7	126.1(5)
C11	Ru1	C5	106.11(17)	C1	C2	Ru1	69.2(2)
C11	Ru1	C2	114.41(17)	C1	C2	C3	109.3(4)
C11	Ru1	C4	142.06(17)	C1	C2	C7	123.6(5)
C11	Ru1	C1	92.51(18)	C7	C2	Ru1	134.3(3)
C5	Ru1	P1	86.61(11)	C43	C42	C45	119.7(5)
C5	Ru1	C3	60.86(16)	C41	C42	C43	118.3(4)
C5	Ru1	C2	59.87(15)	C41	C42	C45	122.0(5)
C5	Ru1	C4	36.35(17)	C27	C35	C34	116.2(5)
C5	Ru1	C1	36.78(15)	C27	C35	C36	122.4(5)
C4	Ru1	C3	36.21(15)	C27	C35	C36A	122.2(8)
C4	Ru1	C2	59.16(14)	C34	C35	C36	121.0(6)
C1	Ru1	C3	60.28(17)	C34	C35	C36A	118.3(8)
C1	Ru1	C2	35.53(16)	C40	C41	C42	121.2(4)

Table S33 Bond Angles for 6.

Atom	Atom	Atom	Angle/˚	Atom	Atom	Atom	Angle/˚
C1	Ru1	C4	60.37(16)	C27	C28	C29	122.6(4)
N1	P1	Ru1	113.05(13)	C32	C28	C27	117.4(5)
O2A	S2	C39	104.0(10)	C32	C28	C29	120.0(5)
O2A	S2	N1	105.3(9)	C13	C14	C18	118.0(4)
O2A	S2	O3A	115.5(11)	C13	C14	C15	121.8(4)
N1	S2	C39	111.8(6)	C18	C14	C15	119.9(4)
O3A	S2	C39	108.0(8)	C3	C4	Ru1	72.3(2)
O3A	S2	N1	112.0(9)	C3	C4	C9	127.5(5)
C12	N3	C13	131.0(3)	C5	C4	Ru1	68.9(2)
C12	N3	C25	111.4(3)	C5	C4	C3	107.7(4)
C25	N3	C13	117.6(3)	C5	C4	C9	124.7(5)
C12	N2	C27	130.9(3)	C9	C4	Ru1	127.6(3)
C12	N2	C26	111.9(3)	C19	C18	C14	120.8(4)
C26	N2	C27	116.7(3)	C5	C1	Ru1	70.9(2)
N3	C12	Ru1	131.3(2)	C5	C1	C6	126.0(5)
N2	C12	Ru1	125.9(2)	C2	C1	Ru1	75.3(2)
N2	C12	N3	102.6(3)	C2	C1	C5	108.1(4)
C35	C27	N2	118.4(4)	C2	C1	C6	125.8(4)
C35	C27	C28	124.1(4)	C6	C1	Ru1	123.8(4)
C28	C27	N2	117.0(4)	C18	C19	C20	120.3(4)
C13	C21	C22	124.4(3)	C28	C32	C33	120.3(5)
C20	C21	C13	117.0(4)	C35	C34	C33	119.8(6)
C20	C21	C22	118.5(3)	C14	C15	C17	110.3(4)
C21	C13	N3	118.2(3)	C16	C15	C14	113.7(5)
C14	C13	N3	118.9(3)	C16	C15	C17	111.3(5)

Table S33 Bond Angles for 6.

Atom	Atom	Atom	Angle/˚	Atom	Atom	Atom	Angle/˚
C14	C13	C21	122.1(3)	C32	C33	C34	122.1(5)
C40	C39	S2	113.7(6)	C28	C29	C31A	121.9(15)
C40	C39	S1	125.0(8)	C28	C29	C30A	94.2(12)
C44	C39	S2	125.8(6)	C28	C29	C30	117.9(6)
C44	C39	C40	120.5(4)	C28	C29	C31	108.3(6)
C44	C39	S1	114.5(8)	C31A	C29	C30	107.4(13)
P1	N1	S2	124.0(5)	C35	C36	C37	112.2(7)
P1	N1	S1	128.6(5)	C35	C36	C38	106.9(7)
C26	C25	N3	107.3(3)	C37	C36	C38	111.7(7)
C41	C40	C39	120.2(4)	N1	S1	C39	104.7(7)
C39	C44	C43	119.5(4)	O2	S1	C39	110.0(14)
C19	C20	C21	121.4(4)	O2	S1	N1	111.8(12)
C2	C3	Ru1	72.6(2)	O3	S1	C39	105.1(9)
C2	C3	C4	106.7(4)	O3	S1	N1	106.4(11)
C2	C3	C8	126.2(5)	O3	S1	O2	117.9(14)
C4	C3	Ru1	71.5(2)	C37A	C36A	C35	109.8(13)
C4	C3	C8	126.8(5)	C37A	C36A	C38A	107.0(15)
C8	C3	Ru1	126.5(3)	C38A	C36A	C35	125.1(14)

Table S34 Torsion Angles for 6.

A	B	C	D	Angle/˚	A	B	C	D	Angle/˚
Ru1	P1	N1	S2	165.1(6)	C40	C39	S1	O3	-34.9(12)
Ru1	P1	N1	S1	149.1(10)	C44	C39	C40	C41	0.3(6)
Ru1	C3	C2	C1	-59.4(3)	C44	C39	S1	N1	-102.7(10)
Ru1	C3	C2	C7	132.1(4)	C44	C39	S1	O2	17.6(13)

Table S34 Torsion Angles for 6.

A	B	C	D	Angle/˚	A	B	C	D	Angle/˚
Ru1	C3	C4	C5	60.1(3)	C44	C39	S1	O3	145.4(9)
Ru1	C3	C4	C9	-124.2(4)	C44	C43	C42	C41	-0.9(6)
Ru1	C5	C4	C3	-62.3(3)	C44	C43	C42	C45	178.1(4)
Ru1	C5	C4	C9	121.9(4)	C20	C21	C13	N3	-176.0(3)
Ru1	C5	C1	C2	66.6(3)	C20	C21	C13	C14	-6.5(5)
Ru1	C5	C1	C6	-118.4(5)	C20	C21	C22	C23	-48.4(5)
Ru1	C2	C1	C5	-63.8(3)	C20	C21	C22	C24	72.6(5)
Ru1	C2	C1	C6	121.3(5)	C3	C2	C1	Ru1	61.0(3)
P1	N1	S1	C39	17.5(16)	C3	C2	C1	C5	-2.7(5)
P1	N1	S1	O2	-101.6(16)	C3	C2	C1	C6	-177.7(4)
P1	N1	S1	O3	128.4(11)	C43	C42	C41	C40	0.9(6)
S2	C39	C40	C41	-178.8(5)	C26	N2	C12	Ru1	-176.1(3)
S2	C39	C44	C43	178.6(5)	C26	N2	C12	N3	0.3(4)
N3	C13	C14	C18	175.1(3)	C26	N2	C27	C35	-93.9(5)
N3	C13	C14	C15	1.8(5)	C26	N2	C27	C28	78.1(5)
N3	C25	C26	N2	-0.5(5)	C22	C21	C13	N3	-0.5(5)
N2	C27	C35	C34	168.4(4)	C22	C21	C13	C14	169.0(4)
N2	C27	C35	C36	-18.3(7)	C22	C21	C20	C19	-172.8(4)
N2	C27	C35	C36A	9.2(9)	C2	C3	C4	Ru1	-64.6(2)
N2	C27	C28	C32	-167.5(4)	C2	C3	C4	C5	-4.5(4)
N2	C27	C28	C29	11.2(6)	C2	C3	C4	C9	171.2(4)
C12	N3	C13	C21	-116.7(4)	C35	C27	C28	C32	4.0(6)
C12	N3	C13	C14	73.4(5)	C35	C27	C28	C29	-177.3(4)
C12	N3	C25	C26	0.7(5)	C35	C34	C33	C32	2.3(8)
C12	N2	C27	C35	94.3(5)	C28	C27	C35	C34	-3.0(6)

Table S34 Torsion Angles for 6.

A	B	C	D	Angle/˚	A	B	C	D	Angle/˚
C12	N2	C27	C28	-93.8(5)	C28	C27	C35	C36	170.3(5)
C12	N2	C26	C25	0.1(5)	C28	C27	C35	C36A	-162.1(8)
C27	N2	C12	Ru1	-3.9(6)	C28	C32	C33	C34	-1.3(8)
C27	N2	C12	N3	172.5(4)	C14	C18	C19	C20	-2.3(7)
C27	N2	C26	C25	-173.3(4)	C4	C3	C2	Ru1	63.9(2)
C27	C35	C34	C33	-0.2(7)	C4	C3	C2	C1	4.5(4)
C27	C35	C36	C37	-147.6(6)	C4	C3	C2	C7	-164.0(4)
C27	C35	C36	C38	89.7(7)	C4	C5	C1	Ru1	-66.8(3)
C27	C35	C36A	C37A	-91.4(15)	C4	C5	C1	C2	-0.2(5)
C27	C35	C36A	C38A	139.3(16)	C4	C5	C1	C6	174.8(5)
C27	C28	C32	C33	-1.8(7)	C18	C14	C15	C17	-87.8(6)
C27	C28	C29	C31A	127.6(13)	C18	C14	C15	C16	38.0(6)
C27	C28	C29	C30A	-100.8(13)	C1	C5	C4	Ru1	65.2(3)
C27	C28	C29	C30	-95.7(7)	C1	C5	C4	C3	2.9(4)
C27	C28	C29	C31	141.6(5)	C1	C5	C4	C9	-172.9(4)
C21	C13	C14	C18	5.7(6)	C32	C28	C29	C31A	-53.7(14)
C21	C13	C14	C15	-167.6(4)	C32	C28	C29	C30A	77.8(13)
C21	C20	C19	C18	1.3(7)	C32	C28	C29	C30	83.0(7)
O2A	S2	C39	C40	-153.3(10)	C32	C28	C29	C31	-39.8(7)
O2A	S2	C39	C44	27.7(12)	C34	C35	C36	C37	25.4(8)
O2A	S2	N1	P1	-120.3(12)	C34	C35	C36	C38	-97.3(8)
C13	N3	C12	Ru1	-2.2(5)	C34	C35	C36A	C37A	109.8(13)
C13	N3	C12	N2	-178.3(3)	C34	C35	C36A	C38A	-19(2)
C13	N3	C25	C26	178.7(4)	C10	C5	C4	Ru1	-124.1(4)
C13	C21	C20	C19	2.9(6)	C10	C5	C4	C3	173.6(4)

Table S34 Torsion Angles for 6.

A	B	C	D	Angle/˚	A	B	C	D	Angle/˚
C13	C21	C22	C23	136.2(4)	C10	C5	C4	C9	-2.2(7)
C13	C21	C22	C24	-102.8(4)	C10	C5	C1	Ru1	122.5(5)
C13	C14	C18	C19	-1.1(7)	C10	C5	C1	C2	-170.9(4)
C13	C14	C15	C17	85.4(5)	C10	C5	C1	C6	4.1(8)
C13	C14	C15	C16	-148.8(4)	C15	C14	C18	C19	172.3(4)
C39	S2	N1	P1	-8.0(10)	C29	C28	C32	C33	179.5(5)
C39	C40	C41	C42	-0.6(6)	C7	C2	C1	Ru1	-130.1(4)
C39	C44	C43	C42	0.6(6)	C7	C2	C1	C5	166.1(4)
N1	S2	C39	C40	93.5(8)	C7	C2	C1	C6	-8.8(7)
N1	S2	C39	C44	-85.5(8)	C36	C35	C34	C33	-173.6(5)
C25	N3	C12	Ru1	175.5(3)	C8	C3	C2	Ru1	-122.9(4)
C25	N3	C12	N2	-0.6(4)	C8	C3	C2	C1	177.8(4)
C25	N3	C13	C21	65.7(4)	C8	C3	C2	C7	9.3(6)
C25	N3	C13	C14	-104.1(4)	C8	C3	C4	Ru1	122.2(4)
O3A	S2	C39	C40	-30.1(9)	C8	C3	C4	C5	-177.8(4)
O3A	S2	C39	C44	150.9(8)	C8	C3	C4	C9	-2.1(7)
O3A	S2	N1	P1	113.4(9)	C45	C42	C41	C40	-178.1(4)
C40	C39	C44	C43	-0.3(6)	S1	C39	C40	C41	-179.4(5)
C40	C39	S1	N1	76.9(11)	S1	C39	C44	C43	179.4(5)
C40	C39	S1	O2	-162.7(12)	C36A	C35	C34	C33	159.8(9)

Table S35 Hydrogen Atom Coordinates (Å×10^4) and Isotropic Displacement Parameters (Å^2×10^3) for 6.

Atom	*x*	*y*	*z*	U(eq)
H25	5883.6	3442.35	8929.39	58

Table S35 Hydrogen Atom Coordinates (Å×10[4]) and Isotropic Displacement Parameters (Å[2]×10[3]) for 6.

Atom	*x*	*y*	*z*	U(eq)
H40	5836.53	5307.38	5383.06	63
H44	8035.08	7307.34	5495.89	54
H20	8843.54	4307.48	10222.78	57
H43	9243.55	6574.83	4881.65	60
H26	5309.03	3149.94	7925.68	65
H22	8184.67	3396.37	8840.21	55
H41	7019.32	4577.06	4776.23	66
H18	6687.21	6324.77	10125.85	71
H23A	10048.81	3827.97	9165.85	112
H23B	9891.79	2792.67	9170.51	112
H23C	9847.23	3330.12	9759.65	112
H19	8092.94	5558.83	10615.38	71
H32	3667.53	4734.28	6337.2	89
H34	6542.85	3501.06	5892.42	83
H10A	8888.55	7301.49	6639.99	109
H10B	9503.21	8017.23	7038.32	109
H10C	8156.63	7930.86	7033.68	109
H9A	10142.03	5135.61	7006.17	108
H9B	10631.7	6071.58	6840.16	108
H9C	9396.38	5788.28	6617.37	108
H33	4717.6	3994.46	5672.36	96
H17A	4587.13	4938.2	9194.85	123
H17B	3897.92	5815.13	9062.65	123
H17C	4413.99	5638.88	9694.92	123

Table S35 Hydrogen Atom Coordinates (Å×10^4) and Isotropic Displacement Parameters (Å^2×10^3) for 6.

**Atom**	***x***	***y***	***z***	**U(eq)**
H24A	7984.91	2757.68	9994.39	101
H24B	8052.82	2154.05	9434.8	101
H24C	6974.4	2762.59	9524.41	101
H7A	8522.56	6698.5	9212.43	116
H7B	9866.36	6745.87	9163.15	116
H7C	9248.6	5820.42	9192.22	116
H36	8087.47	4061.61	7170.96	72
H8A	9544.9	4675.23	8535.99	123
H8B	10758.77	4982.57	8326.65	123
H8C	9908.51	4496.14	7887.85	123
H6A	7573.34	8152.19	8096.66	138
H6B	8827.76	8442.35	8282.39	138
H6C	8074.03	7906.36	8721.7	138
H45A	8657.61	5047.27	3960.57	116
H45B	9127.77	4440.95	4472.88	116
H45C	9732.35	5348.99	4340.94	116
H16A	5418.56	7082.98	9637.36	142
H16B	4996.5	7248.83	8987.02	142
H16C	6318.32	7251.77	9146.98	142
H31A	2810.38	6008.68	7239.66	126
H31B	3965.21	6283.73	6946	126
H31C	3807.32	6438.52	7620.35	126
H37A	9302.39	3616.78	6472.53	123
H37B	8404.13	4250.75	6162.38	123

Table S35 Hydrogen Atom Coordinates (Å×10^4) and Isotropic Displacement Parameters (Å^2×10^3) for 6.

Atom	*x*	*y*	*z*	U(eq)
H37C	8291.42	3218.15	6086.31	123
H30A	2612.3	4557.64	7677.39	126
H30B	3697.46	3981.15	7844.06	126
H30C	3229.56	4006.72	7192.28	126
H38A	7490.49	2318.21	6845.69	140
H38B	7267.25	2728.44	7465.74	140
H38C	8533.11	2574.19	7263.01	140
H37D	8988.31	3915.75	6604.02	85
H37E	9255.55	3489.67	7221.01	85
H37F	8533.7	4369	7173.65	85
H38D	7243.4	2011.81	6626.74	99
H38E	8509.79	2102.21	6875.55	99
H38F	8176.24	2497.5	6257.16	99
H36A	7426.17	3111.4	7340.78	57
H30D	2864.68	5000.72	8030.46	126
H30E	3573.35	4124.56	7945.56	126
H30F	2639.51	4408.36	7472.65	126
H31D	3059.64	6220.7	7383.87	126
H31E	3046.78	5679.84	6794.98	126
H31F	4094.43	6298.65	6956.85	126
H15	5690(30)	5970(20)	8710(18)	34(10)
H29	4640(40)	5350(30)	7750(20)	64(14)

Table S36 Atomic Occupancy for 6.

Atom	*Occupancy*	Atom	*Occupancy*	Atom	*Occupancy*
S2	0.54(4)	O2A	0.54(4)	O3A	0.54(4)
C36	0.751(8)	H36	0.751(8)	C31A	0.248(8)
H31A	0.248(8)	H31B	0.248(8)	H31C	0.248(8)
C37	0.751(8)	H37A	0.751(8)	H37B	0.751(8)
H37C	0.751(8)	C30A	0.248(8)	H30A	0.248(8)
H30B	0.248(8)	H30C	0.248(8)	C38	0.751(8)
H38A	0.751(8)	H38B	0.751(8)	H38C	0.751(8)
S1	0.46(4)	O2	0.46(4)	O3	0.46(4)
C37A	0.249(8)	H37D	0.249(8)	H37E	0.249(8)
H37F	0.249(8)	C38A	0.249(8)	H38D	0.249(8)
H38E	0.249(8)	H38F	0.249(8)	C36A	0.249(8)
H36A	0.249(8)	C30	0.751(8)	H30D	0.751(8)
H30E	0.751(8)	H30F	0.751(8)	C31	0.751(8)
H31D	0.751(8)	H31E	0.751(8)	H31F	0.751(8)

Experimental

Single crystals of $C_{45}H_{58}N_3O_3PRuS$ **6** were grown from pentane at – 30 ^oC. A suitable crystal was selected and mounted on a **ROD, Synergy Custom DW system, Pilatus 200K** diffractometer. The crystal was kept at 100 K during data collection. Using Olex2 [1], the structure was solved with the ShelX [2] structure solution.

1. Dolomanov, O.V., Bourhis, L.J., Gildea, R.J, Howard, J.A.K. & Puschmann, H. (2009), J. Appl. Cryst. 42, 339-341.

Crystal structure determination of [TSD49B_Tilley]

Crystal Data for $C_{45}H_{57.9975}N_3O_3PRuS$ (M =853.04 g/mol): monoclinic, space group $P2_1/c$ (no. 14), a = 11.84530(10) Å, b = 15.3524(2) Å, c = 23.2358(3) Å, β = 91.4090(10)°, V = 4224.24(9) Å^3, Z = 4, T = 100 K, μ(Cu Kα) = 4.154 mm^{-1}, $Dcalc$ = 1.341 g/cm^3, 60880 reflections measured (6.902° ≤ 2Θ ≤ 149.002°), 8602 unique (R_{int} = 0.0513, R_{sigma} = 0.0275) which were used in all calculations. The final R_1 was 0.0509 (I > 2σ(I)) and wR_2 was 0.1290 (all data).

Refinement model description

Number of restraints - 123, number of constraints - unknown.

Details:

1. Restrained distances
 C35-C36 = C35-C36A
 1.47 with sigma of 0.02
 C36A-C38A = C36A-C37A
 1.53 with sigma of 0.02
 C29-C30A
 1.54 with sigma of 0.02
 C29-C31A
 1.54 with sigma of 0.02
 C29-C30
 1.5 with sigma of 0.02
 C29-C31
 1.5 with sigma of 0.02
 S1-O2 ≈ S2-O2A
 with sigma of 0.02
 S1-O3 ≈ S2-O3A
 with sigma of 0.02
 C15-C17 ≈ C15-C16
 with sigma of 0.02
 C17-C16 ≈ C17-C14 ≈ C16-C14
 with sigma of 0.04
 C29-C31A ≈ C29-C30A ≈ C29-C30 ≈ C29-C31
 with sigma of 0.02
 C31A-C30A ≈ C31-C30
 with sigma of 0.04
2. Uiso/Uaniso restraints and constraints
S1 ≈ O2 ≈ O3: within 2A with sigma of 0.04 and sigma for terminal atoms of 0.08 within 2A
S2 ≈ O2A ≈ O3A: within 2A with sigma of 0.04 and sigma for terminal atoms of 0.08 within 2A
C36A ≈ C38A ≈ C38 ≈ C36 ≈ C37A ≈ C37: within 2A with sigma of 0.04 and sigma for terminal atoms of 0.08 within 2A
C29 ≈ C15: within 2A with sigma of 0.04 and sigma for terminal atoms of 0.08 within 2A
Uanis(C29) = Uanis(C31A) = Uanis(C30A)
Uanis(C31) = Uanis(C31A) = Uanis(C30A) = Uanis(C30) = Uanis(C29)
3. Rigid body (RIGU) restrains
 C36, C37, C36, C38
 with sigma for 1-2 distances of 0.004 and sigma for 1-3 distances of 0.004
 C36A, C38A, C36A, C37A
 with sigma for 1-2 distances of 0.004 and sigma for 1-3 distances of 0.004
4. Others
 1*[Sof(C36)+Sof(H36)+Sof(C37)+Sof(H37A)+Sof(H37B)+Sof(H37C)+Sof(C38)+

Sof(H38A)+Sof(H38B)+Sof(H38C)+Sof(C37A)+Sof(H37D)+Sof(H37E)+Sof(H37F)+
Sof(C38A)+Sof(H38D)+Sof(H38E)+Sof(H38F)+Sof(C36A)+Sof(H36A)+Sof(C30)+Sof(H30D)+
Sof(H30E)+Sof(H30F)+Sof(C31)+Sof(H31D)+Sof(H31E)+Sof(H31F)]+1*[Sof(C31A)+
Sof(H31A)+Sof(H31B)+Sof(H31C)+Sof(C30A)+Sof(H30A)+Sof(H30B)+Sof(H30C)]=1 with
esd of 0.001
Sof(S2)=Sof(O2A)=Sof(O3A)=1-FVAR(1)
Sof(S1)=Sof(O2)=Sof(O3)=FVAR(1)
Sof(C37A)=Sof(H37D)=Sof(H37E)=Sof(H37F)=Sof(C38A)=Sof(H38D)=Sof(H38E)=
Sof(H38F)=Sof(C36A)=Sof(H36A)=1-FVAR(2)
Sof(C36)=Sof(H36)=Sof(C37)=Sof(H37A)=Sof(H37B)=Sof(H37C)=Sof(C38)=Sof(H38A)=
Sof(H38B)=Sof(H38C)=Sof(C30)=Sof(H30D)=Sof(H30E)=Sof(H30F)=Sof(C31)=Sof(H31D)=
Sof(H31E)=Sof(H31F)=FVAR(2)
Sof(C31A)=Sof(H31A)=Sof(H31B)=Sof(H31C)=Sof(C30A)=Sof(H30A)=Sof(H30B)=
Sof(H30C)=FVAR(3)
5.a Ternary CH refined with riding coordinates:
C22(H22), C36(H36), C36A(H36A)
5.b Aromatic/amide H refined with riding coordinates:
C25(H25), C40(H40), C44(H44), C20(H20), C43(H43), C26(H26), C41(H41),
C18(H18), C19(H19), C32(H32), C34(H34), C33(H33)
5.c Idealized Me refined as rotating group:
C23(H23A,H23B,H23C), C10(H10A,H10B,H10C), C9(H9A,H9B,H9C), C17(H17A,H17B,
H17C), C24(H24A,H24B,H24C), C7(H7A,H7B,H7C), C8(H8A,H8B,H8C), C6(H6A,H6B,H6C),
C45(H45A,H45B,H45C), C16(H16A,H16B,H16C), C31A(H31A,H31B,H31C), C37(H37A,H37B,
H37C), C30A(H30A,H30B,H30C), C38(H38A,H38B,H38C), C37A(H37D,H37E,H37F),
C38A(H38D,H38E,H38F), C30(H30D,H30E,H30F), C31(H31D,H31E,H31F)

Phosphaformazan (7)

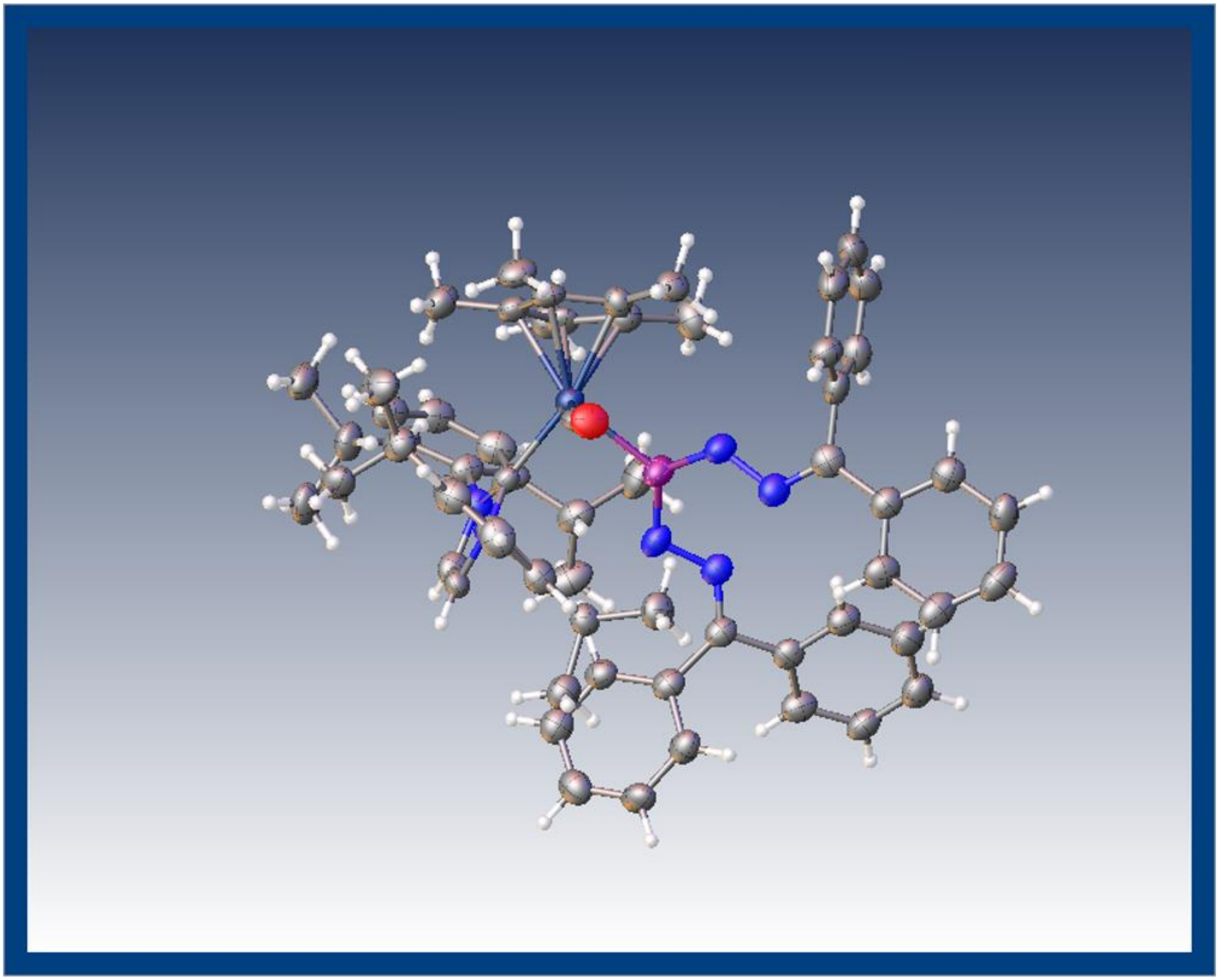

Table S36 Crystal data and structure refinement for 7.

Identification code	TSD63_Tilley
Empirical formula	$C_{64}H_{71}N_6OPRu$
Formula weight	1072.30
Temperature/K	100
Crystal system	triclinic
Space group	P-1
a/Å	11.9317(6)
b/Å	12.1876(8)

c/Å	20.8438(10)
α/°	87.709(5)
β/°	86.970(4)
γ/°	72.349(5)
Volume/$Å^3$	2883.4(3)
Z	2
ρ_{calc}g/cm^3	1.235
μ/mm^{-1}	2.811
F(000)	1128.0
Crystal size/mm^3	0.31 × 0.16 × 0.1
Radiation	Cu Kα (λ = 1.54184)
2Θ range for data collection/°	7.614 to 133.19
Index ranges	-9 ≤ h ≤ 14, -14 ≤ k ≤ 14, -24 ≤ l ≤ 24
Reflections collected	54880
Independent reflections	10180 [R_{int} = 0.1882, R_{sigma} = 0.1228]
Data/restraints/parameters	10180/0/671
Goodness-of-fit on F^2	0.999
Final R indexes [I>=2σ (I)]	R_1 = 0.0824, wR_2 = 0.1987
Final R indexes [all data]	R_1 = 0.1311, wR_2 = 0.2320
Largest diff. peak/hole / e $Å^{-3}$	1.44/-1.04

Table S37 Fractional Atomic Coordinates (×10^4) and Equivalent Isotropic Displacement Parameters ($Å^2$×10^3) for 7. U_{eq} is defined as 1/3 of the trace of the orthogonalised U_{IJ} tensor.

**Atom**	***x***	***y***	***z***	**U(eq)**
Ru1	6806.6(4)	2064.9(5)	2158.6(3)	35.83(19)
P1	5484.1(16)	3836.9(16)	1976.0(11)	45.1(5)

Table S37 Fractional Atomic Coordinates (×10^4) and Equivalent Isotropic Displacement Parameters (Å^2×10^3) for 7. U_{eq} is defined as 1/3 of the trace of the orthogonalised U_{IJ} tensor.

Atom	*x*	*y*	*z*	U(eq)
O1	4693(4)	1327(5)	2588(3)	51.3(14)
N2	6523(4)	2770(5)	3586(3)	34.6(12)
N1	8054(4)	3171(5)	3180(3)	33.6(12)
N5	4821(5)	6033(5)	2043(3)	41.2(14)
N6	5479(5)	4989(5)	2319(3)	43.1(14)
N3	3429(5)	4675(5)	1488(3)	46.4(15)
N4	4435(5)	3751(5)	1554(3)	46.2(15)
C12	7219(5)	2655(6)	3023(3)	34.8(15)
C34	3585(6)	2463(7)	4049(4)	44.0(17)
C26	6867(5)	3400(6)	4041(3)	35.6(15)
C1	5506(7)	1653(6)	2502(4)	43.4(17)
C52	4450(6)	6933(6)	2384(4)	39.1(16)
C14	9250(6)	4036(6)	2429(4)	40.6(16)
C53	3793(5)	7974(6)	2012(4)	38.6(15)
C13	9188(5)	3136(6)	2846(4)	38.1(16)
C27	5617(6)	2250(6)	3767(4)	37.5(15)
C4	7417(6)	1919(6)	1100(4)	43.1(17)
C3	6793(6)	1126(6)	1261(4)	41.2(16)
C20	11288(6)	2307(7)	2788(4)	47.4(19)
C28	5931(6)	1070(6)	3918(4)	41.4(16)
C35	4448(6)	2977(6)	3827(4)	39.0(16)
C38	3712(6)	4919(7)	4317(4)	50.7(19)
C59	4576(6)	7053(6)	3084(4)	40.7(16)
C25	7803(5)	3671(6)	3773(4)	39.5(16)

Table S37 Fractional Atomic Coordinates (×10[4]) and Equivalent Isotropic Displacement Parameters (Å^2×10^3) for 7. U_{eq} is defined as 1/3 of the trace of the orthogonalised U_{IJ} tensor.

Atom	*x*	*y*	*z*	U(eq)
C36	4090(6)	4263(6)	3693(4)	40.7(16)
C54	3144(6)	7849(7)	1500(4)	47.3(18)
C29	7192(7)	297(7)	3862(4)	49.4(18)
C6	8453(6)	653(6)	1860(4)	41.9(16)
C19	11361(6)	3145(7)	2358(4)	50(2)
C18	10366(7)	4014(7)	2182(4)	50.3(19)
C58	3833(6)	9072(6)	2146(4)	45.5(18)
C48	28(6)	6565(7)	276(4)	52(2)
C40	3222(6)	3781(6)	483(4)	44.6(17)
C46	1679(6)	5574(6)	924(4)	41.6(17)
C47	1072(6)	5686(7)	364(4)	49.2(19)
C8	5737(6)	1051(7)	931(4)	46.3(18)
C45	3929(6)	3989(7)	-28(4)	50.7(19)
C014	5613(6)	6479(6)	3413(4)	43.8(17)
C15	8201(7)	5062(7)	2287(4)	49.1(19)
C21	10184(6)	2283(6)	3067(4)	44.0(17)
C2	7446(6)	310(6)	1720(4)	43.0(17)
C42	3303(7)	1998(7)	11(5)	59(2)
C61	3756(7)	7991(7)	4090(4)	49.5(18)
C60	3653(6)	7790(7)	3448(4)	47.4(18)
C39	2823(6)	4651(6)	997(4)	43.7(17)
C56	2563(6)	9883(7)	1278(4)	51.5(19)
C51	1201(6)	6364(7)	1403(4)	48.9(19)
C5	8448(5)	1655(6)	1457(4)	40.5(16)

Table S37 Fractional Atomic Coordinates (×10^4) and Equivalent Isotropic Displacement Parameters (Å^2×10^3) for 7. U_{eq} is defined as 1/3 of the trace of the orthogonalised U_{IJ} tensor.

Atom	*x*	*y*	*z*	U(eq)
C41	2889(6)	2788(7)	500(4)	47.7(18)
C33	3888(7)	1310(7)	4188(4)	50(2)
C57	3218(6)	10025(6)	1774(4)	46.5(18)
C22	10157(6)	1379(7)	3589(4)	52(2)
C44	4314(7)	3193(8)	-519(4)	60(2)
C32	5025(7)	623(7)	4136(4)	51.5(19)
C30	7872(7)	396(8)	4444(4)	53.0(19)
C63	5713(7)	6707(7)	4041(4)	50.6(19)
C10	9464(6)	2109(7)	1326(4)	53(2)
C31	7280(9)	-964(8)	3769(5)	68(2)
C50	170(6)	7243(7)	1312(5)	53(2)
C7	7187(7)	-753(7)	1971(5)	54(2)
C49	-409(7)	7351(7)	755(5)	58(2)
C37	3115(6)	4652(7)	3213(4)	52(2)
C9	7095(8)	2840(8)	571(4)	58(2)
C43	4006(7)	2200(8)	-490(4)	57(2)
C55	2528(7)	8796(7)	1143(4)	55(2)
C23	11110(7)	223(7)	3509(5)	60(2)
C62	4792(7)	7463(7)	4377(4)	51.0(19)
C11	9442(7)	8(7)	2260(5)	55(2)
C17	8321(8)	6112(7)	2611(6)	67(3)
C24	10279(7)	1828(9)	4248(5)	62(2)
C16	8048(8)	5298(8)	1562(5)	65(2)

Table S38 Anisotropic Displacement Parameters (Å^2×10^3) for 7. The Anisotropic displacement factor exponent takes the form: $-2\pi^2[h^2a^{*2}U_{11}+2hka^*b^*U_{12}+\ldots]$.

Atom	U_{11}	U_{22}	U_{33}	U_{23}	U_{13}	U_{12}
Ru1	33.7(3)	33.7(3)	40.6(3)	-1.1(2)	-1.1(2)	-10.86(19)
P1	42.1(9)	35.4(9)	57.3(13)	-2.8(9)	-9.0(9)	-9.3(7)
O1	43(3)	57(3)	64(4)	-8(3)	0(2)	-30(2)
N2	34(3)	33(3)	39(3)	-4(2)	-1(2)	-13(2)
N1	31(3)	36(3)	39(3)	2(3)	-5(2)	-17(2)
N5	37(3)	39(3)	48(4)	1(3)	1(3)	-13(2)
N6	36(3)	37(3)	52(4)	5(3)	0(3)	-6(2)
N3	41(3)	44(3)	55(4)	-2(3)	-7(3)	-13(3)
N4	42(3)	39(3)	54(4)	-7(3)	-5(3)	-6(3)
C12	33(3)	31(3)	36(4)	3(3)	4(3)	-5(3)
C34	40(4)	55(4)	45(5)	0(4)	-2(3)	-26(3)
C26	39(3)	38(3)	31(4)	0(3)	-8(3)	-12(3)
C1	54(4)	37(4)	37(4)	-15(3)	-2(3)	-10(3)
C52	35(3)	35(4)	50(5)	-1(3)	1(3)	-14(3)
C14	45(4)	39(4)	42(4)	-10(3)	1(3)	-18(3)
C53	32(3)	42(4)	42(4)	0(3)	6(3)	-12(3)
C13	30(3)	40(4)	49(5)	-6(3)	1(3)	-17(3)
C27	37(3)	42(4)	40(4)	-4(3)	3(3)	-22(3)
C4	40(4)	44(4)	47(5)	-4(3)	-1(3)	-15(3)
C3	42(4)	45(4)	36(4)	1(3)	-1(3)	-13(3)
C20	33(3)	54(4)	60(5)	-10(4)	1(3)	-19(3)
C28	53(4)	42(4)	33(4)	3(3)	2(3)	-21(3)
C35	34(3)	46(4)	41(4)	-1(3)	1(3)	-19(3)
C38	44(4)	49(4)	60(6)	1(4)	-4(4)	-14(3)

Table S38 Anisotropic Displacement Parameters (Å^2×10^3) for 7. The Anisotropic displacement factor exponent takes the form: -2π^2[h^{2}a*^{2}U$_{11}$+2hka*b*U$_{12}$+…].

Atom	U$_{11}$	U$_{22}$	U$_{33}$	U$_{23}$	U$_{13}$	U$_{12}$
C59	40(4)	33(3)	50(5)	1(3)	1(3)	-14(3)
C25	33(3)	35(4)	53(5)	1(3)	-8(3)	-15(3)
C36	39(3)	43(4)	43(4)	2(3)	-5(3)	-17(3)
C54	40(4)	44(4)	57(5)	-5(4)	-2(3)	-13(3)
C29	54(4)	44(4)	49(5)	5(4)	1(4)	-16(3)
C6	39(3)	38(4)	44(5)	-8(3)	4(3)	-5(3)
C19	36(4)	61(5)	60(5)	-11(4)	5(3)	-25(3)
C18	53(4)	55(5)	53(5)	-4(4)	11(4)	-33(4)
C58	39(4)	43(4)	58(5)	-6(4)	5(3)	-17(3)
C48	38(4)	60(5)	55(5)	11(4)	-5(3)	-11(3)
C40	36(3)	45(4)	47(5)	8(3)	-6(3)	-4(3)
C46	35(3)	41(4)	50(5)	0(3)	2(3)	-14(3)
C47	43(4)	53(5)	52(5)	2(4)	3(3)	-16(3)
C8	45(4)	50(4)	49(5)	-9(4)	-7(3)	-18(3)
C45	41(4)	59(5)	52(5)	11(4)	-1(3)	-16(3)
C014	48(4)	36(4)	45(5)	-1(3)	3(3)	-11(3)
C15	48(4)	44(4)	56(5)	2(4)	10(4)	-18(3)
C21	37(4)	44(4)	53(5)	-5(4)	-1(3)	-14(3)
C2	44(4)	35(4)	48(5)	-14(3)	5(3)	-7(3)
C42	60(5)	47(5)	68(6)	-3(4)	-23(5)	-11(4)
C61	56(4)	46(4)	40(5)	-2(4)	10(4)	-8(3)
C60	44(4)	43(4)	50(5)	7(4)	2(3)	-8(3)
C39	41(4)	46(4)	49(5)	2(3)	5(3)	-21(3)
C56	41(4)	52(5)	55(5)	3(4)	-2(4)	-6(3)

Table S38 Anisotropic Displacement Parameters (Å^2×10^3) for 7. The Anisotropic displacement factor exponent takes the form: -2π^2[h^{2}a*^{2}U$_{11}$+2hka*b*U$_{12}$+…].

Atom	U_{11}	U_{22}	U_{33}	U_{23}	U_{13}	U_{12}
C51	35(4)	53(5)	60(5)	-6(4)	6(3)	-15(3)
C5	32(3)	49(4)	37(4)	-6(3)	14(3)	-9(3)
C41	45(4)	44(4)	53(5)	-5(4)	-1(3)	-10(3)
C33	46(4)	61(5)	56(5)	8(4)	0(4)	-35(4)
C57	39(4)	37(4)	60(5)	-1(4)	5(3)	-7(3)
C22	34(4)	59(5)	60(6)	9(4)	-13(3)	-9(3)
C44	54(5)	71(6)	45(5)	-3(4)	2(4)	-6(4)
C32	60(5)	51(5)	51(5)	3(4)	5(4)	-29(4)
C30	52(4)	58(5)	44(5)	0(4)	9(4)	-10(4)
C63	45(4)	49(4)	60(6)	3(4)	-3(4)	-17(3)
C10	44(4)	57(5)	59(6)	-2(4)	0(4)	-19(4)
C31	90(6)	47(5)	61(6)	10(4)	8(5)	-16(4)
C50	37(4)	45(4)	80(7)	-2(4)	-2(4)	-15(3)
C7	56(4)	38(4)	70(6)	-15(4)	0(4)	-14(3)
C49	39(4)	45(4)	87(7)	7(4)	1(4)	-7(3)
C37	43(4)	59(5)	59(6)	3(4)	-9(4)	-19(4)
C9	66(5)	67(5)	49(5)	11(4)	-7(4)	-31(4)
C43	58(5)	65(5)	42(5)	-4(4)	-11(4)	-5(4)
C55	45(4)	56(5)	59(6)	-1(4)	-5(4)	-9(4)
C23	51(4)	53(5)	74(7)	-3(5)	-21(4)	-9(4)
C62	63(5)	50(4)	44(5)	4(4)	-4(4)	-24(4)
C11	46(4)	46(4)	70(6)	-5(4)	-8(4)	-6(3)
C17	59(5)	42(4)	101(8)	-7(5)	18(5)	-21(4)
C24	38(4)	78(6)	63(6)	10(5)	-11(4)	-5(4)

Table S38 Anisotropic Displacement Parameters (Å^2×10^3) for 7. The Anisotropic displacement factor exponent takes the form: $-2\pi^2[h^2a^{*2}U_{11}+2hka^*b^*U_{12}+…]$.

Atom	U_{11}	U_{22}	U_{33}	U_{23}	U_{13}	U_{12}
C16	58(5)	51(5)	81(7)	20(5)	-8(5)	-10(4)

Table S39 Bond Lengths for 7.

Atom	Atom	Length/Å	Atom	Atom	Length/Å
Ru1	P1	2.2870(19)	C28	C32	1.399(10)
Ru1	C12	2.101(7)	C35	C36	1.512(10)
Ru1	C1	1.870(8)	C38	C36	1.529(11)
Ru1	C4	2.284(8)	C59	C014	1.421(10)
Ru1	C3	2.235(7)	C59	C60	1.401(10)
Ru1	C6	2.261(7)	C36	C37	1.526(10)
Ru1	C2	2.255(7)	C54	C55	1.379(12)
Ru1	C5	2.321(6)	C29	C30	1.523(12)
P1	N6	1.599(6)	C29	C31	1.528(12)
P1	N4	1.599(6)	C6	C2	1.433(10)
O1	C1	1.159(8)	C6	C5	1.452(11)
N2	C12	1.387(9)	C6	C11	1.481(11)
N2	C26	1.395(8)	C19	C18	1.385(12)
N2	C27	1.438(8)	C58	C57	1.399(11)
N1	C12	1.389(8)	C48	C47	1.390(11)
N1	C13	1.477(8)	C48	C49	1.379(13)
N1	C25	1.378(9)	C40	C45	1.387(11)
N5	N6	1.397(8)	C40	C39	1.493(11)
N5	C52	1.279(9)	C40	C41	1.382(10)
N3	N4	1.383(8)	C46	C47	1.386(11)

Table S39 Bond Lengths for 7.

Atom	Atom	Length/Å	Atom	Atom	Length/Å
N3	C39	1.289(10)	C46	C39	1.492(10)
C34	C35	1.409(9)	C46	C51	1.390(11)
C34	C33	1.364(11)	C45	C44	1.399(12)
C26	C25	1.346(9)	C014	C63	1.368(12)
C52	C53	1.486(10)	C15	C17	1.518(12)
C52	C59	1.493(11)	C15	C16	1.538(13)
C14	C13	1.389(10)	C21	C22	1.524(12)
C14	C18	1.395(10)	C2	C7	1.490(11)
C14	C15	1.509(11)	C42	C41	1.396(12)
C53	C54	1.390(11)	C42	C43	1.366(13)
C53	C58	1.394(10)	C61	C60	1.388(12)
C13	C21	1.406(10)	C61	C62	1.365(12)
C27	C28	1.399(10)	C56	C57	1.375(11)
C27	C35	1.410(10)	C56	C55	1.378(12)
C4	C3	1.409(10)	C51	C50	1.381(11)
C4	C5	1.417(10)	C5	C10	1.487(10)
C4	C9	1.518(12)	C33	C32	1.362(12)
C3	C8	1.495(9)	C22	C23	1.528(11)
C3	C2	1.429(11)	C22	C24	1.528(13)
C20	C19	1.351(12)	C44	C43	1.366(13)
C20	C21	1.419(10)	C63	C62	1.379(12)
C28	C29	1.515(11)	C50	C49	1.364(13)

Table S40 Bond Angles for 7.

Atom	Atom	Atom	Angle/˚	Atom	Atom	Atom	Angle/˚
P1	Ru1	C5	113.6(2)	C27	C28	C32	117.1(7)
C12	Ru1	P1	89.34(18)	C32	C28	C29	120.5(7)
C12	Ru1	C4	140.0(2)	C34	C35	C27	116.9(7)
C12	Ru1	C3	166.2(2)	C34	C35	C36	119.2(6)
C12	Ru1	C6	104.5(3)	C27	C35	C36	123.8(6)
C12	Ru1	C2	130.8(3)	C014	C59	C52	123.5(6)
C12	Ru1	C5	109.3(2)	C60	C59	C52	119.9(6)
C1	Ru1	P1	85.2(2)	C60	C59	C014	116.5(7)
C1	Ru1	C12	95.8(3)	C26	C25	N1	107.7(6)
C1	Ru1	C4	124.2(3)	C35	C36	C38	110.6(6)
C1	Ru1	C3	90.6(3)	C35	C36	C37	112.4(6)
C1	Ru1	C6	118.7(3)	C37	C36	C38	110.2(6)
C1	Ru1	C2	87.5(3)	C55	C54	C53	120.6(7)
C1	Ru1	C5	148.2(3)	C28	C29	C30	111.2(7)
C4	Ru1	P1	91.63(19)	C28	C29	C31	112.8(7)
C4	Ru1	C5	35.8(3)	C30	C29	C31	109.8(7)
C3	Ru1	P1	103.4(2)	C2	C6	Ru1	71.3(4)
C3	Ru1	C4	36.3(2)	C2	C6	C5	108.2(6)
C3	Ru1	C6	61.7(3)	C2	C6	C11	126.8(7)
C3	Ru1	C2	37.1(3)	C5	C6	Ru1	73.8(4)
C3	Ru1	C5	61.0(2)	C5	C6	C11	124.3(6)
C6	Ru1	P1	150.1(2)	C11	C6	Ru1	128.3(6)
C6	Ru1	C4	60.7(3)	C20	C19	C18	121.2(7)
C6	Ru1	C5	36.9(3)	C19	C18	C14	121.1(8)
C2	Ru1	P1	139.8(2)	C53	C58	C57	120.2(7)

Table S40 Bond Angles for 7.

Atom	Atom	Atom	Angle/˚	Atom	Atom	Atom	Angle/˚
C2	Ru1	C4	60.8(3)	C49	C48	C47	119.3(8)
C2	Ru1	C6	37.0(2)	C45	C40	C39	118.8(7)
C2	Ru1	C5	61.4(3)	C41	C40	C45	119.5(8)
N6	P1	Ru1	124.6(2)	C41	C40	C39	121.7(7)
N6	P1	N4	123.2(3)	C47	C46	C39	120.6(7)
N4	P1	Ru1	111.5(2)	C47	C46	C51	117.7(7)
C12	N2	C26	112.1(5)	C51	C46	C39	121.6(7)
C12	N2	C27	128.9(5)	C46	C47	C48	121.6(8)
C26	N2	C27	118.6(6)	C40	C45	C44	120.3(8)
C12	N1	C13	129.5(6)	C63	C014	C59	120.6(7)
C25	N1	C12	112.0(5)	C14	C15	C17	109.4(7)
C25	N1	C13	118.1(5)	C14	C15	C16	112.3(7)
C52	N5	N6	120.2(6)	C17	C15	C16	110.7(7)
N5	N6	P1	117.1(5)	C13	C21	C20	116.3(7)
C39	N3	N4	115.4(6)	C13	C21	C22	124.8(6)
N3	N4	P1	121.1(5)	C20	C21	C22	118.9(7)
N2	C12	Ru1	124.3(4)	C3	C2	Ru1	70.6(4)
N2	C12	N1	101.7(6)	C3	C2	C6	107.4(6)
N1	C12	Ru1	133.4(5)	C3	C2	C7	126.7(6)
C33	C34	C35	120.4(7)	C6	C2	Ru1	71.7(4)
C25	C26	N2	106.2(6)	C6	C2	C7	125.8(7)
O1	C1	Ru1	166.0(6)	C7	C2	Ru1	126.4(5)
N5	C52	C53	113.1(7)	C43	C42	C41	121.3(8)
N5	C52	C59	129.0(7)	C62	C61	C60	119.2(7)
C53	C52	C59	117.9(6)	C61	C60	C59	122.1(7)

Table S40 Bond Angles for 7.

Atom	Atom	Atom	Angle/˚	Atom	Atom	Atom	Angle/˚
C13	C14	C18	116.8(7)	N3	C39	C40	123.7(7)
C13	C14	C15	123.2(6)	N3	C39	C46	118.2(7)
C18	C14	C15	119.8(7)	C46	C39	C40	118.0(6)
C54	C53	C52	119.1(6)	C57	C56	C55	119.7(8)
C54	C53	C58	118.6(7)	C50	C51	C46	120.6(8)
C58	C53	C52	122.2(6)	C4	C5	Ru1	70.7(4)
C14	C13	N1	119.6(6)	C4	C5	C6	106.3(6)
C14	C13	C21	123.5(6)	C4	C5	C10	126.3(7)
C21	C13	N1	115.9(6)	C6	C5	Ru1	69.3(4)
C28	C27	N2	119.3(6)	C6	C5	C10	125.7(7)
C28	C27	C35	122.7(6)	C10	C5	Ru1	136.2(5)
C35	C27	N2	117.8(6)	C40	C41	C42	119.1(8)
C3	C4	Ru1	69.9(4)	C32	C33	C34	121.9(6)
C3	C4	C5	109.9(7)	C56	C57	C58	120.1(7)
C3	C4	C9	124.7(6)	C21	C22	C23	114.9(7)
C5	C4	Ru1	73.5(4)	C21	C22	C24	110.1(7)
C5	C4	C9	125.1(6)	C23	C22	C24	107.7(7)
C9	C4	Ru1	127.9(6)	C43	C44	C45	119.9(9)
C4	C3	Ru1	73.8(4)	C33	C32	C28	121.0(8)
C4	C3	C8	125.2(7)	C014	C63	C62	120.8(7)
C4	C3	C2	108.1(6)	C49	C50	C51	120.9(8)
C8	C3	Ru1	127.1(5)	C50	C49	C48	119.9(8)
C2	C3	Ru1	72.2(4)	C44	C43	C42	119.9(9)
C2	C3	C8	126.1(7)	C56	C55	C54	120.7(8)
C19	C20	C21	120.9(7)	C61	C62	C63	120.6(8)

Table S40 Bond Angles for 7.

Atom	Atom	Atom	Angle/˚	Atom	Atom	Atom	Angle/˚
C27	C28	C29	122.4(6)				

Table S41 Torsion Angles for 7.

A	B	C	D	Angle/˚	A	B	C	D	Angle/˚
Ru1	P1	N6	N5	164.2(4)	C35	C27	C28	C29	-178.9(7)
Ru1	P1	N4	N3	167.6(5)	C35	C27	C28	C32	0.1(11)
Ru1	C4	C3	C8	124.2(8)	C59	C52	C53	C54	-147.3(6)
Ru1	C4	C3	C2	-64.6(5)	C59	C52	C53	C58	34.7(9)
Ru1	C4	C5	C6	60.5(5)	C59	C014	C63	C62	2.6(11)
Ru1	C4	C5	C10	-133.8(7)	C25	N1	C12	Ru1	164.5(5)
Ru1	C3	C2	C6	-62.7(5)	C25	N1	C12	N2	-6.1(7)
Ru1	C3	C2	C7	121.6(8)	C25	N1	C13	C14	-90.6(8)
Ru1	C6	C2	C3	62.0(5)	C25	N1	C13	C21	77.8(8)
Ru1	C6	C2	C7	-122.2(8)	C54	C53	C58	C57	0.8(10)
Ru1	C6	C5	C4	-61.4(5)	C29	C28	C32	C33	180.0(8)
Ru1	C6	C5	C10	132.7(7)	C6	Ru1	C1	O1	-68(3)
P1	Ru1	C1	O1	93(3)	C19	C20	C21	C13	3.2(11)
N2	C26	C25	N1	-2.7(7)	C19	C20	C21	C22	-175.1(7)
N2	C27	C28	C29	-3.5(11)	C18	C14	C13	N1	173.3(6)
N2	C27	C28	C32	175.5(7)	C18	C14	C13	C21	5.8(11)
N2	C27	C35	C34	-175.6(6)	C18	C14	C15	C17	-66.6(10)
N2	C27	C35	C36	2.0(10)	C18	C14	C15	C16	56.8(9)
N1	C13	C21	C20	-174.6(6)	C58	C53	C54	C55	-1.7(11)
N1	C13	C21	C22	3.6(10)	C40	C45	C44	C43	-0.8(12)
N5	C52	C53	C54	31.7(9)	C46	C51	C50	C49	0.5(12)

Table S41 Torsion Angles for 7.

A	B	C	D	Angle/˚	A	B	C	D	Angle/˚
N5	C52	C53	C58	-146.2(7)	C47	C48	C49	C50	-1.5(12)
N5	C52	C59	C014	39.2(10)	C47	C46	C39	N3	171.0(7)
N5	C52	C59	C60	-142.8(7)	C47	C46	C39	C40	-6.5(10)
N6	P1	N4	N3	-3.0(8)	C47	C46	C51	C50	-1.5(11)
N6	N5	C52	C53	178.5(5)	C8	C3	C2	Ru1	-123.3(8)
N6	N5	C52	C59	-2.6(10)	C8	C3	C2	C6	174.0(7)
N4	P1	N6	N5	-26.4(7)	C8	C3	C2	C7	-1.8(12)
N4	N3	C39	C40	-7.1(10)	C45	C40	C39	N3	-86.4(9)
N4	N3	C39	C46	175.4(6)	C45	C40	C39	C46	91.1(8)
C12	Ru1	C1	O1	-178(3)	C45	C40	C41	C42	2.0(11)
C12	N2	C26	C25	-1.2(8)	C45	C44	C43	C42	1.3(12)
C12	N2	C27	C28	71.4(10)	C014	C59	C60	C61	2.6(11)
C12	N2	C27	C35	-112.9(8)	C014	C63	C62	C61	0.6(12)
C12	N1	C13	C14	97.4(8)	C15	C14	C13	N1	-1.0(10)
C12	N1	C13	C21	-94.1(8)	C15	C14	C13	C21	-168.5(7)
C12	N1	C25	C26	5.8(8)	C15	C14	C18	C19	173.1(7)
C34	C35	C36	C38	67.6(8)	C21	C20	C19	C18	0.8(12)
C34	C35	C36	C37	-56.0(9)	C2	Ru1	C1	O1	-48(3)
C34	C33	C32	C28	-1.9(13)	C2	C6	C5	Ru1	63.4(5)
C26	N2	C12	Ru1	-167.4(4)	C2	C6	C5	C4	2.1(8)
C26	N2	C12	N1	4.4(7)	C2	C6	C5	C10	-163.8(7)
C26	N2	C27	C28	-101.7(8)	C60	C59	C014	C63	-4.1(10)
C26	N2	C27	C35	73.9(8)	C60	C61	C62	C63	-2.2(12)
C52	N5	N6	P1	156.6(5)	C39	N3	N4	P1	159.3(6)
C52	C53	C54	C55	-179.8(7)	C39	C40	C45	C44	-179.8(7)

Table S41 Torsion Angles for 7.

A	B	C	D	Angle/˚	A	B	C	D	Angle/˚
C52	C53	C58	C57	178.7(7)	C39	C40	C41	C42	-179.1(7)
C52	C59	C014	C63	173.9(7)	C39	C46	C47	C48	-177.5(7)
C52	C59	C60	C61	-175.5(7)	C39	C46	C51	C50	177.0(7)
C14	C13	C21	C20	-6.7(11)	C51	C46	C47	C48	1.0(11)
C14	C13	C21	C22	171.5(7)	C51	C46	C39	N3	-7.4(10)
C53	C52	C59	C014	-141.9(6)	C51	C46	C39	C40	175.0(7)
C53	C52	C59	C60	36.1(9)	C51	C50	C49	C48	1.0(12)
C53	C54	C55	C56	1.4(12)	C5	Ru1	C1	O1	-36(3)
C53	C58	C57	C56	0.6(11)	C5	C4	C3	Ru1	63.0(5)
C13	N1	C12	Ru1	-23.1(10)	C5	C4	C3	C8	-172.8(7)
C13	N1	C12	N2	166.2(6)	C5	C4	C3	C2	-1.6(9)
C13	N1	C25	C26	-167.5(6)	C5	C6	C2	Ru1	-65.1(5)
C13	C14	C18	C19	-1.4(11)	C5	C6	C2	C3	-3.0(8)
C13	C14	C15	C17	107.6(8)	C5	C6	C2	C7	172.7(7)
C13	C14	C15	C16	-129.1(7)	C41	C40	C45	C44	-0.9(11)
C13	C21	C22	C23	147.9(7)	C41	C40	C39	N3	94.8(9)
C13	C21	C22	C24	-90.4(8)	C41	C40	C39	C46	-87.8(9)
C27	N2	C12	Ru1	19.1(9)	C41	C42	C43	C44	-0.1(12)
C27	N2	C12	N1	-169.2(6)	C33	C34	C35	C27	-0.8(11)
C27	N2	C26	C25	173.1(6)	C33	C34	C35	C36	-178.6(7)
C27	C28	C29	C30	80.7(9)	C57	C56	C55	C54	0.0(12)
C27	C28	C29	C31	-155.5(8)	C32	C28	C29	C30	-98.3(8)
C27	C28	C32	C33	0.9(12)	C32	C28	C29	C31	25.5(11)
C27	C35	C36	C38	-110.0(8)	C49	C48	C47	C46	0.5(12)
C27	C35	C36	C37	126.4(8)	C9	C4	C3	Ru1	-122.9(8)

Table S41 Torsion Angles for 7.

A	B	C	D	Angle/˚	A	B	C	D	Angle/˚
C4	Ru1	C1	O1	4(3)	C9	C4	C3	C8	1.3(12)
C4	C3	C2	Ru1	65.6(5)	C9	C4	C3	C2	172.5(7)
C4	C3	C2	C6	2.8(8)	C9	C4	C5	Ru1	125.1(8)
C4	C3	C2	C7	-172.9(7)	C9	C4	C5	C6	-174.4(7)
C3	Ru1	C1	O1	-11(3)	C9	C4	C5	C10	-8.6(12)
C3	C4	C5	Ru1	-60.8(5)	C43	C42	C41	C40	-1.6(12)
C3	C4	C5	C6	-0.3(8)	C55	C56	C57	C58	-0.9(12)
C3	C4	C5	C10	165.5(7)	C62	C61	C60	C59	0.5(12)
C20	C19	C18	C14	-1.8(12)	C11	C6	C2	Ru1	124.3(8)
C20	C21	C22	C23	-34.0(10)	C11	C6	C2	C3	-173.6(7)
C20	C21	C22	C24	87.8(8)	C11	C6	C2	C7	2.1(13)
C28	C27	C35	C34	-0.1(11)	C11	C6	C5	Ru1	-125.7(7)
C28	C27	C35	C36	177.5(7)	C11	C6	C5	C4	173.0(7)
C35	C34	C33	C32	1.9(13)	C11	C6	C5	C10	7.1(12)

Table S42 Hydrogen Atom Coordinates (Å×10^4) and Isotropic Displacement Parameters (Å^2×10^3) for 7.

Atom	*x*	*y*	*z*	U(eq)
H34	2786.71	2922.2	4103	53
H26	6512.59	3596.66	4455.84	43
H20	11984.39	1725.96	2904.93	57
H38A	3019.47	4744.11	4513.42	76
H38B	3516.2	5748.8	4222.49	76
H38C	4356.88	4686.57	4613.52	76
H25	8216.02	4125.45	3958.79	47

Table S42 Hydrogen Atom Coordinates (Å×10⁴) and Isotropic Displacement Parameters (Å²×10³) for 7.

Atom	*x*	*y*	*z*	U(eq)
H36	4794.16	4458.06	3501.78	49
H54	3125.51	7103.56	1394.81	57
H29	7578.1	564.76	3475.37	59
H19	12107.91	3138.3	2173.58	60
H18	10444.74	4604.15	1888.02	60
H58	4279.49	9174.09	2490.17	55
H48	-379.41	6623.9	-109.02	62
H47	1375.55	5148.24	33.18	59
H8A	5322.01	615.15	1205.6	69
H8B	5988.24	659.54	522.74	69
H8C	5210.21	1828.41	848.54	69
H45	4151.75	4675.33	-45.04	61
H014	6244.41	5931.41	3194.97	53
H15	7479.22	4897.09	2475.95	59
H42	3091.18	1305.69	26.06	71
H61	3114.28	8491.22	4326.43	59
H60	2932.69	8164.52	3249.33	57
H56	2136.29	10531.2	1030.16	62
H51	1588.19	6298.82	1795.51	59
H41	2384.93	2645.46	839.67	57
H33	3289.71	976.12	4324.46	60
H57	3253.66	10771.68	1864.72	56
H22	9373.67	1238.62	3588.75	63
H44	4790.13	3343.12	-870.69	72

Table S42 Hydrogen Atom Coordinates (Å×10[4]) and Isotropic Displacement Parameters (Å[2]×10[3]) for 7.

Atom	*x*	*y*	*z*	U(eq)
H32	5205.66	-172.57	4248.87	62
H30A	7857.33	1198.47	4485.32	80
H30B	8689.45	-93.42	4389.28	80
H30C	7507.9	146.13	4832.66	80
H63	6424.86	6340.05	4249.13	61
H10A	9177.99	2949.29	1274.54	79
H10B	9895.11	1776.4	931.84	79
H10C	9989.89	1900.26	1686.95	79
H31A	7003.55	-1280.17	4164.2	102
H31B	8101.51	-1402.79	3667.41	102
H31C	6792.29	-1016.81	3414.48	102
H50	-142.07	7779.16	1643.04	64
H7A	7519.08	-963.7	2394.88	82
H7B	7539.24	-1383.17	1675.39	82
H7C	6333.23	-612.13	2008.05	82
H49	-1111.05	7966.7	696.05	70
H37A	3373.67	4237.34	2812.33	79
H37B	2938.99	5481.69	3125.36	79
H37C	2406.13	4487.22	3393.43	79
H9A	6236.33	3140.86	553.84	87
H9B	7427.43	2505.64	157.59	87
H9C	7413.41	3468.01	660.88	87
H43	4279.72	1651.37	-818.36	69
H55	2075.17	8699.34	800.6	66

Table S42 Hydrogen Atom Coordinates (Å×10[4]) and Isotropic Displacement Parameters (Å[2]×10[3]) for 7.

Atom	*x*	*y*	*z*	U(eq)
H23A	11111.01	-44.27	3071.51	90
H23B	10949.87	-343.19	3818.75	90
H23C	11880.02	313.79	3584.82	90
H62	4878.65	7617.52	4811.49	61
H11A	9717.7	552.22	2493.28	83
H11B	10086.94	-455.77	1983.05	83
H11C	9174.13	-498.35	2567.33	83
H17A	8366.26	5962.28	3076.19	101
H17B	7636.21	6776.69	2523.58	101
H17C	9038.9	6274.28	2443.48	101
H24A	11017.32	2023.8	4250.41	94
H24B	10284.12	1231.96	4578.78	94
H24C	9614.84	2516.08	4337.77	94
H16A	8775.19	5392.31	1360.3	98
H16B	7396.84	6003.44	1492.5	98
H16C	7874.72	4649.23	1370.71	98

Table S43 Solvent masks information for TSD63_Tilley.

Number	X	Y	Z	Volume	Electron count	Content
1	0.000	0.000	0.000	64.4	0.0	?
2	0.000	0.500	0.500	305.3	34.1	?

Experimental

Single crystals of $C_{64}H_{71}N_6OPRu$ **7** were **grown from toluene at – 30 ºC**. A suitable crystal was selected and mounted on a **ROD, Synergy Custom DW system, Pilatus 200K** diffractometer. The

crystal was kept at 100 K during data collection. Using Olex2 [1], the structure was solved with the ShelX [2] structure solution program.

1. Dolomanov, O.V., Bourhis, L.J., Gildea, R.J, Howard, J.A.K. & Puschmann, H. (2009), J. Appl. Cryst. 42, 339-341.

Crystal structure determination of 7

Crystal Data for $C_{64}H_{71}N_6OPRu$ (*M* =1072.30 g/mol): triclinic, space group P-1 (no. 2), a = 11.9317(6) Å, b = 12.1876(8) Å, c = 20.8438(10) Å, α = 87.709(5)°, β = 86.970(4)°, γ = 72.349(5)°, V = 2883.4(3) Å^3, Z = 2, T = 100 K, μ(Cu Kα) = 2.811 mm^{-1}, *Dcalc* = 1.235 g/cm^3, 54880 reflections measured (7.614° ≤ 2Θ ≤ 133.19°), 10180 unique (R_{int} = 0.1882, R_{sigma} = 0.1228) which were used in all calculations. The final R_1 was 0.0824 (I > 2σ(I)) and wR_2 was 0.2320 (all data).

Refinement model description

Number of restraints - 0, number of constraints - unknown.

Details:

1.a Ternary CH refined with riding coordinates:
C36(H36), C29(H29), C15(H15), C22(H22)
1.b Aromatic/amide H refined with riding coordinates:
C34(H34), C26(H26), C20(H20), C25(H25), C54(H54), C19(H19), C18(H18), C58(H58), C48(H48), C47(H47), C45(H45), C014(H014), C42(H42), C61(H61), C60(H60), C56(H56), C51(H51), C41(H41), C33(H33), C57(H57), C44(H44), C32(H32), C63(H63), C50(H50), C49(H49), C43(H43), C55(H55), C62(H62)
1.c Idealized Me refined as rotating group:
C38(H38A,H38B,H38C), C8(H8A,H8B,H8C), C30(H30A,H30B,H30C), C10(H10A,H10B, H10C), C31(H31A,H31B,H31C), C7(H7A,H7B,H7C), C37(H37A,H37B,H37C), C9(H9A,H9B, H9C), C23(H23A,H23B,H23C), C11(H11A,H11B,H11C), C17(H17A,H17B,H17C), C24(H24A, H24B,H24C), C16(H16A,H16B,H16C)

DFT

Reaction Mechanism for the formation of 2

Overview: All calculations have been performed with the ORCA program package.[1] The geometries have been optimized using the BP86,[2] functional together with the def2-SVP[3] basis set. The dispersion correction has been incorporated via the D4[4] scheme and the solvent effects by using the CPCM[5] model with the dielectric constant of THF. The nature of the stationary points has been confirmed by frequency calculations at this level. The final energies have been calculated as single point calculations using the ωB97X-D4[6] functional together with the def2-TZVPPD[7] basis set. The RIJCOSX[8] approximation and the CPCM(THF) corrections for solvent effects has been incorporated. To identify reaction coordinates and to find saddle points representing transition states the Nudged Elastic Band (NEB)[9] method as implemented in Orca has been used. The Electrostatic potential for 2 has been calculated using Gaussian 16.[10]

---

1 a) R. A. Kendall, H. A. Früchtl,. *Theor. Chem. Acc.* **1997**, 97, 158–163. b) F. Neese, *WIREs Comput. Mol. Sci.* **2012**, *2*, 73–78. c) F. Neese, *WIREs Comput. Mol. Sci.* **2018**, *8*, 1327.

2 A. D. Becke, *Phys. Rev. A* **1988**, *38*, 3098-3100; J. P. Perdew, *Phys. Rev. B* **1986**, *33*, 8822-8824. Erratum: *Phys. Rev. B* **1986**, *34*, 7406-7406.

3 F. Weigend, R. Ahlrichs, *Phys. Chem. Chem. Phys.* **2005**, *7*, 3297-3305.

4 E. Caldeweyher, S. Ehlert, A. Hansen, H. Neugebauer, S. Spicher, C. Bannwarth, S. Grimme, *J. Chem. Phys.* **2019**, *150*, 154122.

5 a) J. Tomasi, B. Mennucci, R. Cammi, *Chem. Rev.* **2005**, *105*, 2999-3094; b) V. Barone, M. Cossi, *J. Phys. Chem. A* **1998**, *102*, 1995-2001.

6 J.-D. Chai, M. Head-Gordon, *Phys. Chem. Chem. Phys.* **2008**, *10*, 6615-6620.

7 D. Rappoport, F. Furche, *J. Chem. Phys.* **2010**, *133*, 134105.

8 a) F. Jensen, *J. Chem. Theory Comput.* **2015**, *11*, 132-138; b)F. Neese, F. Wennmohs, A. Hansen, U. Becker, *Chem. Phys.* **2009**, *356*, 98-109.

9 a) V. Ásgeirsson, B. O. Birgisson, R. Bjornsson, U. Becker, F. Neese, C. Riplinger, H. Jónsson, *J. Chem. Theory Comput.* **2021**, *17*, 4929-4945; b) G. Mills, H. Jónsson, G. Schenter *Surface Science*, **1995**, *324*, 305; c) G. Henkelman, H. Jónsson, *J. Chem. Phys.*, **2000**, 113, 9978.

10 Gaussian 16, Revision C.01, M. J. Frisch, G. W. Trucks, H. B. Schlegel, G. E. Scuseria, M. A. Robb, J. R. Cheeseman, G. Scalmani, V. Barone, G. A. Petersson, H. Nakatsuji, X. Li, M. Caricato, A. V. Marenich, J. Bloino, B. G. Janesko, R. Gomperts, B. Mennucci, H. P. Hratchian, J. V. Ortiz, A. F. Izmaylov, J. L. Sonnenberg, D. Williams-Young, F. Ding, F. Lipparini, F. Egidi, J. Goings, B. Peng, A. Petrone, T. Henderson, D. Ranasinghe, V. G. Zakrzewski, J. Gao, N. Rega, G. Zheng, W. Liang, M. Hada, M. Ehara, K. Toyota, R. Fukuda, J. Hasegawa, M. Ishida, T. Nakajima, Y. Honda, O. Kitao, H. Nakai, T. Vreven, K. Throssell, J. A. Montgomery, Jr., J. E. Peralta, F. Ogliaro, M. J. Bearpark, J. J. Heyd, E. N. Brothers, K. N. Kudin, V. N. Staroverov, T. A. Keith, R. Kobayashi, J. Normand, K. Raghavachari, A. P. Rendell, J. C. Burant, S. S. Iyengar, J. Tomasi, M. Cossi, J. M. Millam, M. Klene, C. Adamo, R. Cammi, J. W. Ochterski, R. L. Martin, K. Morokuma, O. Farkas, J. B. Foresman, and D. J. Fox, Gaussian, Inc., Wallingford CT, 2019.

Table S44. Calculated energies (Hartree) and thermodynamic parameters.

	TPSS/def2-SVP					
	Cp*(IDipp)Ru-PCO	TS1	Cp*(IDippP)Ru CO (**2**)	Cp*(IDippPCNXy) Ru(CO) (**TS2**)	XyNC	Cp*(IDipp)Ru-PCNXy
Electronic energy	-2099.083601	-2099.072517	-2099.112289	-2502.028045	-402.9202235	
Total enthalpy	-2098.241214	-2098.238911	-2098.271279	-2501.025031	-402.7582149	
Final entropy term	0.12239442	0.10715511	0.1189404	0.1409139	0.04503762	
Gibbs free energy	-2098.363609	-2098.346067	-2098.390219	-2501.165945	-402.8032525	
Correction for G (G-E(el))	0.71999217	0.72645007	0.72206979	0.86210042	0.11697104	
	wB97X-D4/def2-TZVPPD					
SCF energy	-2101.396197	-2101.388608	-2101.426358	-2504.822207	-403.43556098	

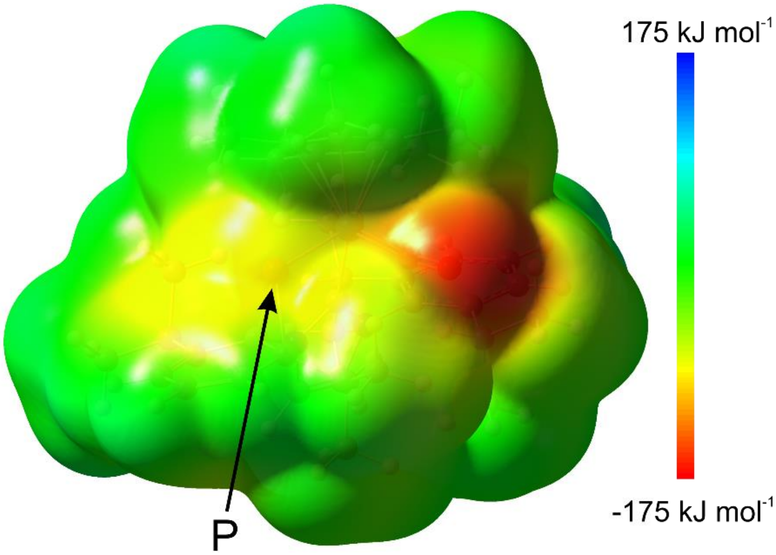

Figure S2. Electrostatic potential of [Cp*(IDippP)Ru(CO)] (**2**) calculated at the wB97X-D4/def2-TZVPPD level.

Cartesian coordinates of [Cp*(IDipp)Ru(PCO)].

Ru	5.72452674879693	17.20407708322002	9.39658550614910
P	7.38069549963531	17.07140158953061	11.07776233869197
N	5.31920544613951	14.13656034565067	8.89907037179427
N	4.31069616276946	14.74773967224440	10.72179349763569
C	4.98650626115940	15.26646517099139	9.62595194936079
C	5.84814282904618	14.09412386371149	7.55166411647763
C	7.24625334721122	14.06097902967498	7.35236637383791
C	3.56296690020842	15.52026569062617	11.68937717138214
C	4.01872241830362	15.60160327263948	13.02623546519206
C	6.02867329977775	19.36619437305950	9.30830405862721
C	1.73196483439677	15.82504528966644	9.90210840180627
H	2.54277821560411	15.96043305716630	9.16440387611054
C	4.61624419160302	19.01671561448022	9.2057203594[illegible]
C	8.20152814652250	14.06591050701194	8.531556159[illegible]
H	7.66009517842777	14.52782548979578	9.368177990[illegible]
C	4.29181911014186	13.35153319475086	10.69669973[illegible]
H	3.81171996240555	12.76840166660660	11.47865276[illegible]
C	4.93040346077382	13.97720599644167	6.478442372[illegible]
C	4.92959785754754	12.96876557864558	9.559741444[illegible]
H	5.14144229798884	11.98294934374083	9.153367992[illegible]
C	3.10392479564346	17.92876068818827	7.344299282[illegible]
H	3.05704174081999	16.88318329673518	7.009036602[illegible]
H	2.94717381111608	18.56944040539654	6.454793098[illegible]
H	2.26707529197457	18.10944173752871	8.031480894[illegible]
C	3.42221621616524	13.94507130604691	6.698315401[illegible]
H	3.21526954052926	14.48150838689225	7.64008587356632
C	5.20583348306593	14.80175602525192	13.54135281835194

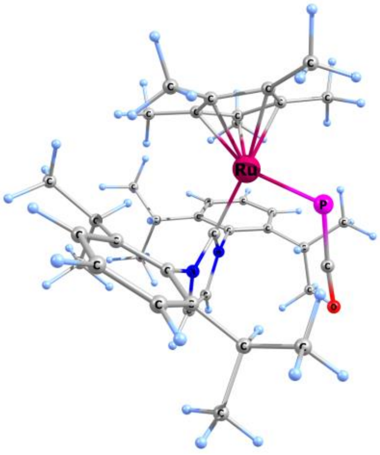

H	5.77264731278848	14.43629188184229	12.67325322029978
C	4.41770329752668	18.26042654172408	7.98439224882750
C	2.32905658252209	16.09663664008495	11.28161036028625
C	6.70996234158005	18.72533567104044	8.22360456095101
C	5.45763958038619	13.84920214768100	5.18054000774054
H	4.77455564129586	13.75950236514383	4.33061788740366
C	6.16024572534406	15.63235995553077	14.41846741559514
H	5.67892926903199	15.93416212448081	15.36593812738533
H	6.49630639812368	16.53885029392019	13.88839857764946
H	7.05109886837647	15.03223396433344	14.67294601494329
C	7.72286274361398	13.94190191334440	6.03479430953351
H	8.80221466476015	13.91814653490560	5.85560022871200
C	3.27044517897431	16.37921229225236	13.93112365623147
H	3.61125366945762	16.46632195935378	14.96765717577662
C	5.71903264097692	18.01553644623458	7.41293165666762
C	0.57632320401012	16.76703921248110	9.52987696800638
H	-0.31279135942215	16.57689050952999	10.15747655033495
H	0.28340939350586	16.59383727634893	8.48032910837807
H	0.84317714222391	17.83185236298001	9.63863643982069
C	6.83825810913301	13.83775111205151	4.95698279760021
H	7.22569137001482	13.74106540432328	3.93692640344909
C	1.22549688746688	14.36756130935917	9.79747293777625
H	2.02922420183613	13.63260210854373	9.95362072088662
H	0.79731322988050	14.18908607034311	8.79544827332165
H	0.43497732720256	14.18340760972504	10.54713338769065
C	5.99790640095631	17.34234921919661	6.10423947144531
H	6.96184517881046	16.80937440742967	6.12152873923905
H	6.04007646406439	18.09201728266421	5.28894672654940
H	5.21466516757339	16.61207074850818	5.85428925823715
C	3.54444321902982	19.51625994321406	10.12657535955165
H	2.68862427490315	18.82582002300985	10.16030827077583
H	3.17363652062595	20.50518765898581	9.79168028698458
H	3.92787068153878	19.63036833271988	11.15347751901660
C	6.63509114350711	20.26694438002329	10.34495808567270
H	6.13009790293189	20.15552688611165	11.31918323477244
H	6.54452925796855	21.32642217909779	10.03762652187136
H	7.70582897869349	20.04932546710733	10.49155522797787
C	8.17125052298249	18.82714217762157	7.90152011318421
H	8.77705155836242	18.97858832805976	8.80915211411336
H	8.35343121104712	19.68108465554488	7.22042743562889
H	8.52992287684819	17.91627772284571	7.39569038416711
C	2.91837441693443	12.49293734666293	6.85416288169526
H	3.13113673934304	11.91553748250584	5.93631893848052
H	1.82681511737732	12.48402946042869	7.02169598691479
H	3.39754802364881	11.97935595489023	7.70203648395163
C	1.62470458671498	16.86366454122714	12.22520173757476
H	0.67981185582918	17.33344014514900	11.94083593608725
C	4.70218760592272	13.56769020930641	14.32396461797635

H	5.55611348061879	12.94415030841579	14.64415967905089
H	4.02838804684892	12.94208732157203	13.71366021584758
H	4.14635430188795	13.87878177644479	15.22716485210739
C	2.09952057054640	17.02474816462155	13.53234046064421
H	1.53561854402592	17.63304866885803	14.24777424492707
C	2.63382207121835	14.63836068777632	5.57109013871972
H	3.04646039107913	15.63135119201266	5.32744449904119
H	1.58114965194516	14.76704889820467	5.87755565088941
H	2.63759610659226	14.03480195012103	4.64618349882381
C	8.55623486518947	12.61950015443571	8.93584682539312
H	7.64953961796072	12.03160474611610	9.15821466713717
H	9.19206101970665	12.62144915948433	9.83813698668150
H	9.10344610364314	12.10891350960629	8.12223964019974
C	9.46951117075930	14.89749754226044	8.28934664025799
H	10.11714369307465	14.44755503663672	7.51556658627035
H	10.05757560016987	14.96042142173622	9.22153318762192
H	9.21686593500842	15.92419327278845	7.97623193427600
O	8.00337703611256	14.38745834998828	11.81562007795304
C	7.69766945861282	15.48992583035356	11.49164074874057

Cartesian coordinates of [Cp*(IDipp)Ru(η^2-PCO)] (**TS1**).

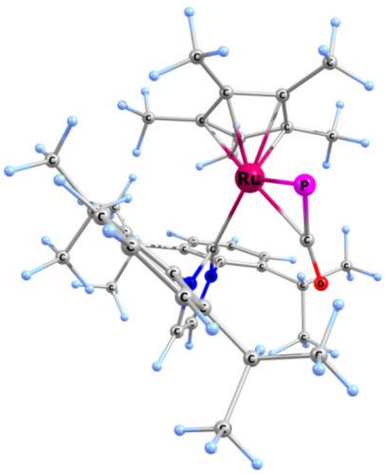

Ru	0.21066101257065	1.59126548955022	-0.58882526560337
P	1.29803009166714	3.10214523770109	0.816433474[illegible]
N	0.16078656340229	-0.41508759915879	1.743587572[illegible]
N	0.62733733573105	-1.54422395014090	-0.036661362[illegible]
O	2.98169827272224	0.81257392241548	0.780109412[illegible]
C	2.10390397956807	1.63272126876650	0.589109927[illegible]
C	-0.30911227386804	0.57106972599390	2.68813991[illegible]
C	0.58257158069544	1.07336813142942	3.650299889[illegible]
C	0.09825907978561	2.03362548130742	4.546332285[illegible]
H	0.77027585486414	2.44930031976739	5.291564603[illegible]
C	-1.22009250774834	2.46725612801342	4.49215367[illegible]
H	-1.57266029351243	3.22886057629810	5.18277464[illegible]
C	-2.10092205611726	1.89989882159761	3.57417631[illegible]
H	-3.13908856631983	2.21337227285554	3.57314257[illegible]
C	-1.67504716638220	0.92122848210586	2.67172280944111
C	0.27659560826133	-0.27187531143465	0.36681397906598
C	2.01278271736751	0.58286003471945	3.79600350226650
H	2.26497268224724	-0.01141845153755	2.91617044723705
C	3.02650870014329	1.73481169919756	3.88492687147043
H	2.93822188558706	2.40048795856927	3.02158969028765
H	4.04372391806720	1.32930285952962	3.91101049283871
H	2.87828805729214	2.32395335876510	4.79694244905662
C	2.13163219606592	-0.31652066765124	5.04365811617986
H	1.91491660305552	0.25723588006753	5.95227049744033
H	3.14932636956814	-0.71487933040429	5.12531746891324
H	1.43415206891367	-1.15950482066846	5.00460339100370

C	-2.66981839373117	0.21340710443842	1.76392441088649
H	-2.23282944504870	0.18775098302016	0.75862545969295
C	-4.03144087322051	0.91353439297791	1.68487152102197
H	-4.55283325483167	0.87170659501618	2.64825877140564
H	-4.65982685320404	0.40362570741285	0.94793656080194
H	-3.94425163011470	1.96472848347587	1.39331036199682
C	-2.89834879605422	-1.24067159040082	2.22993912922930
H	-1.97831171654695	-1.82798689583704	2.21883619074995
H	-3.62166114397580	-1.73158749578595	1.56920870072465
H	-3.30362524157290	-1.24994997985760	3.24842289327061
C	0.76616452832856	-2.40944011510259	1.04659000407581
H	1.05632931519629	-3.43769708724741	0.91089431673717
C	0.47308105954610	-1.70414791853641	2.15694558326200
H	0.43272803863045	-1.99343286782074	3.19377790017972
C	-0.08018965434507	3.37017588303501	-1.90943789842682
C	-0.40073561430167	-2.80113284066056	-1.85698277067268
C	0.71758347969780	-2.09843362662595	-1.36937442067271
C	0.45688171753033	2.24223335597624	-2.63341045474627
C	-0.53597382592247	1.19920217827801	-2.64300577017952
C	1.94364128298751	-2.06321120832560	-2.05502555512706
C	-1.33697566506633	2.97096180950459	-1.36201931681499
C	-0.27002244583309	-3.45661766368121	-3.08609812598079
H	-1.11646825991022	-4.00289828021242	-3.49083659199122
C	-1.63256747936035	1.62781773940653	-1.82260507124145
C	2.01670749864597	-2.72087921797792	-3.28774842015159
H	2.94817764520702	-2.70186481351689	-3.84474833700082
C	3.16661415160795	-1.40859524255045	-1.44824717666202
H	2.82685606538280	-0.60726259820043	-0.78906391187421
C	4.11511808309488	-0.79939856751733	-2.48921464698667
H	4.61309936223806	-1.57077484069954	-3.08814174306526
H	4.89455557733620	-0.22362643769093	-1.97873788287931
H	3.58679483269549	-0.12711530739160	-3.17097840574610
C	0.92279001080344	-3.41244944515408	-3.79994971465953
H	1.00220202036966	-3.92328252716979	-4.75598991027346
C	-1.71053953029756	-2.88456685560910	-1.08782027375244
H	-1.74713680260193	-2.03385392815472	-0.39965448815973
C	-2.24522467960244	3.84719370465675	-0.55975248269920
H	-2.86264251022613	3.26282085273843	0.12554292904233
H	-2.91861463282474	4.40582293590881	-1.22457572627479
H	-1.67465210032413	4.57179522519934	0.02688498405440
C	0.52225547061724	4.73972928044973	-1.85628908418589
H	0.21825563902869	5.28254421478545	-0.95759390827882
H	0.18997069146251	5.31718526590485	-2.73006060809207
H	1.61446869024218	4.69915791599469	-1.87349899909605
C	3.93538888177456	-2.42761131256751	-0.58106886874863
H	3.29836630084870	-2.85758358552162	0.19700706587044
H	4.78518897858312	-1.93752692124125	-0.09269067720931
H	4.31885126607480	-3.24678053235669	-1.20127106983952

C	-1.78566824174493	-4.18192061121941	-0.25586793728472
H	-1.73030236727042	-5.05438666573068	-0.91701914268180
H	-2.73645643959535	-4.22289401130792	0.28712851236613
H	-0.97447210023944	-4.24831643558004	0.47200576562883
C	1.71475812758848	2.24954117849292	-3.44119228286829
H	2.51257701080825	2.80406439494071	-2.94118014505761
H	1.52908820906090	2.72782891049013	-4.41372818777613
H	2.06281655246176	1.23254706018630	-3.63156247230797
C	-0.52416681724936	0.02296939926215	-3.56347893382552
H	0.46739168848318	-0.42439093573826	-3.65453075352117
H	-0.83714770525790	0.35184599096274	-4.56535702994370
H	-1.21725102858532	-0.75029014409904	-3.23668644931820
C	-2.96120777191844	0.94705224342438	-1.70746297876522
H	-2.86096716804627	-0.11446129522944	-1.47456002128374
H	-3.50686463085868	1.02866289306217	-2.65776113135143
H	-3.57418244100924	1.41004703437536	-0.93319167265934
C	-2.94478524538372	-2.81779734141816	-2.00379618857257
H	-2.89581240145164	-1.98066691547399	-2.70582790756751
H	-3.84690418998116	-2.70227929061020	-1.39354742780026
H	-3.05490719445187	-3.73954665320995	-2.58533820777983

VIBRATIONAL FREQUENCIES

0: 0.00 cm**-1
1: 0.00 cm**-1
2: 0.00 cm**-1
3: 0.00 cm**-1
4: 0.00 cm**-1
5: 0.00 cm**-1
6: -69.32 cm-1 ***imaginary mode*****
7: 21.62 cm**-1
8: 37.34 cm**-1
9: 38.64 cm**-1
10: 40.92 cm**-1

Cartesian coordinates of [Cp*(IDippP)Ru(CO)] (**2**).

Ru	3.73769001931908	6.44975141050418	14.64237619351443
P	3.30041703569373	7.26615258259200	16.82206611652048
N	3.24093800153813	4.48997834219359	16.89629365184293
N	3.77101063560377	3.29195679717169	15.17505617325300
O	6.70971400406459	6.83922172773933	14.81112222604040
C	5.55665134168405	6.59040529630591	14.82630727131118
C	2.99782657980935	5.62688149056048	17.76434263093461
C	4.27102675598077	6.36070342964625	18.22668709548516
C	4.12304095197216	7.01294231470669	19.54712106657412

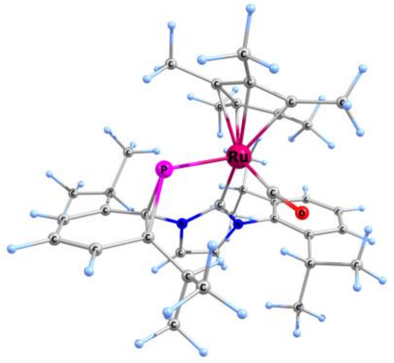

H	4.99238232848916	7.55458906294378	19.93689172775187
C	2.98745960775616	6.93514878250564	20.29203222929396
H	2.93867940740351	7.44154180626286	21.26288397859949
C	1.83862244214548	6.15811538349862	19.86063414929077
H	0.96230925656059	6.13052956867954	20.51482732214573
C	1.83019412915465	5.48066743550210	18.67578653854423
C	3.55118398473258	4.60337199611361	15.55640529553562
C	5.65962204970221	5.76302345699331	17.95261206026514
H	5.62962505150445	5.32510811259069	16.94021365011087
C	6.74544672351151	6.85060695711078	17.96436414277202
H	6.46247388371843	7.70123129554218	17.32290594698851
H	7.70121253497307	6.44238802678855	17.59245468971919
H	6.92176683057210	7.23139737582957	18.98665380985208
C	6.00609979746027	4.64243001592608	18.95191441009446
H	6.04104608054664	5.03443444245021	19.98487900352431
H	6.99604725130466	4.20975310328303	18.71822136036850
H	5.26526748451078	3.82658588751180	18.92304526141675
C	0.62039039690174	4.71878754125165	18.15412712337267
H	0.95250912549552	3.70808980488769	17.84856239735099
C	0.08914069894619	5.41295297544397	16.88249884143679
H	-0.30668586593797	6.41443779538406	17.12863218996825
H	-0.72287656722772	4.81866181800954	16.42731391673955
H	0.88892090075354	5.54124170677554	16.13457545188665
C	-0.49375360628188	4.53069482705785	19.19113896940457
H	-0.12219817284766	4.02409344282669	20.09907610920567
H	-1.30464386094154	3.91598562936328	18.76274536075907
H	-0.93216163955111	5.49883924112894	19.49375621679023
C	3.61874105132873	2.41829566979929	16.25365344871257
H	3.77513139680528	1.34797169152647	16.14799393323048
C	3.29282023710820	3.17363242961081	17.33686085393201
H	3.10072251852033	2.89678063601664	18.37102632938142
C	3.22574959209766	8.33927340135751	13.56324430418863
C	3.18424905437418	2.08899171487988	13.11134322374242
C	4.14907591913212	2.78595413495566	13.87672414932821
C	3.88752885731525	7.42937927236904	12.65135654483041
C	3.06388358700716	6.25650900079362	12.49142877842712
C	5.50339082247441	2.88613287978336	13.47781030863337
C	1.95137880281143	7.74609384614093	13.91741325516328
C	3.59880584083016	1.53740418393630	11.88441223976483
H	2.87507154470647	0.99501651171160	11.26826931901162
C	1.86480740090699	6.46343286174898	13.28641299199638
C	5.85892469323637	2.33834159009726	12.23304869025858
H	6.89494675935664	2.41128577733339	11.89020976271889
C	6.56084730823325	3.46424330052788	14.40892495754154
H	6.07097589018312	4.24623807030350	15.01025437064236
C	7.74050243641834	4.10565970119171	13.66344636208398
H	8.35316689355252	3.34654827885061	13.14455739042836
H	8.39498383537230	4.62876101260813	14.38041538467238

H	7.39606831557431	4.84152054176194	12.91747224974434
C	4.91611023630381	1.67461239345565	11.43963316440142
H	5.21599344269549	1.24759570408932	10.47654248899162
C	1.75167348933945	1.87477376743971	13.58993617318455
H	1.54567858370422	2.62130653634594	14.37765423919527
C	0.90394413905596	8.42787323818527	14.74583875940808
H	0.01162947716843	7.79593376380663	14.87214945890402
H	0.59484685616531	9.37292844827256	14.26337343978248
H	1.28820575930350	8.67708364630697	15.75463066468043
C	3.66777916856816	9.72903578898160	13.92760184462447
H	3.36752133815725	9.98553838050505	14.95748206180551
H	3.21562135264515	10.47910723957049	13.24977765857805
H	4.76291750190115	9.83097605993581	13.85790093855186
C	7.06717544976118	2.37603611694467	15.38212225790386
H	6.24289916686036	1.93819743297427	15.96903330235290
H	7.79555643478282	2.81006216543684	16.09049257721627
H	7.56616053681311	1.55983666066830	14.82878396192814
C	1.58799052736315	0.46952846931932	14.21219654353238
H	1.78853112149846	-0.30950811813509	13.45475739376939
H	0.55589421181146	0.33300360263211	14.58133395045822
H	2.27774666291586	0.30792581957358	15.05551715100856
C	5.15575081310291	7.70648047106341	11.89267403862999
H	5.81576489469831	8.39683977437047	12.44108450789061
H	4.91767739852188	8.17048451038653	10.91684226210422
H	5.71541148597003	6.77761482442991	11.69630775820029
C	3.27398883788994	5.17255142094933	11.47545629058699
H	4.33783391084120	4.90142101760037	11.38272301555324
H	2.92088421576580	5.50061497739847	10.47743563114002
H	2.72304092433429	4.25967190958788	11.74127424919984
C	0.67314756823952	5.54886045722865	13.29242984507186
H	0.93587543105848	4.54061535305878	13.64414735221263
H	0.26375572545033	5.44925175647434	12.27016910305652
H	-0.12729950693175	5.93492575275998	13.94067237665744
C	0.71418972776831	2.05816424786680	12.46489800457723
H	0.86213524480163	3.00278189641873	11.91729800580347
H	-0.30429975203335	2.06120427101197	12.89059111624876
H	0.76559928734103	1.23070285269794	11.73543498568797

Theoretical versus experimental IR spectroscopy of 6

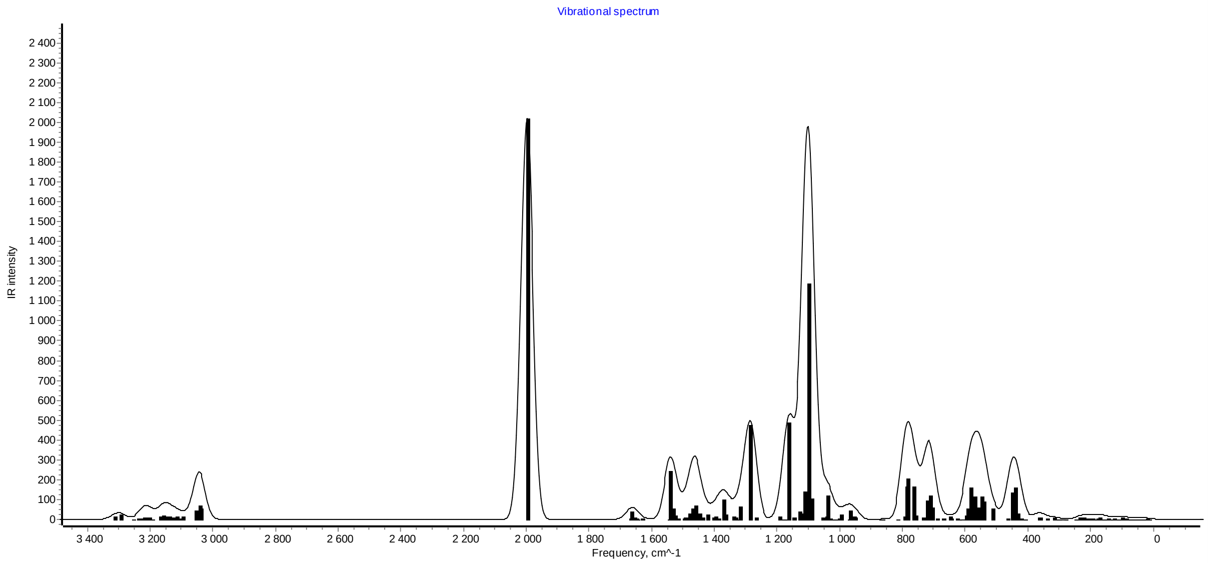

*Figure S3 Theoretical IR spectrum of **6***

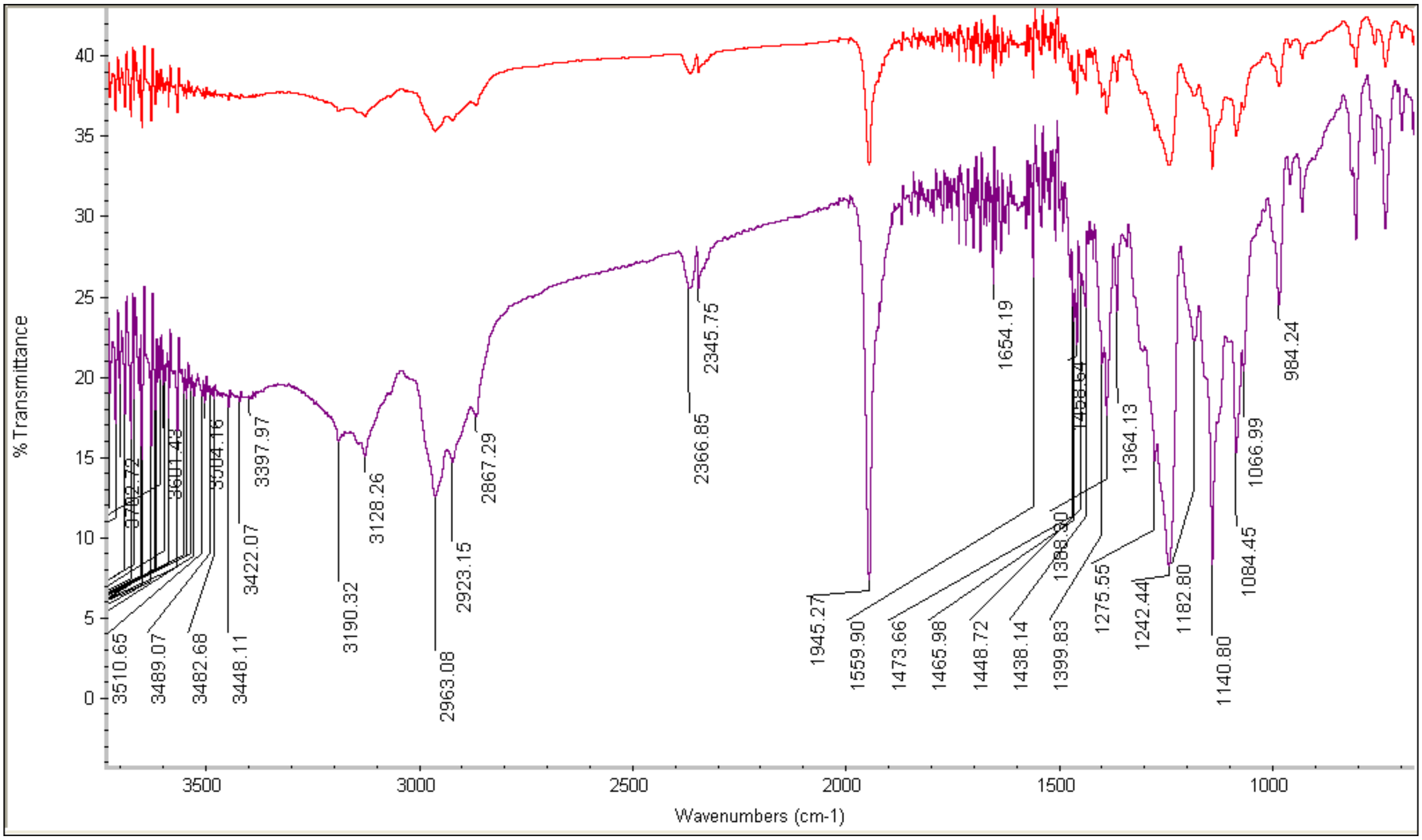

*Figure S4 Experimental IR spectrum of **6***

Commentary: To locate the P=N stretching frequency, calculations were performed at the PBE0 level of theory with a dispersion (D3BJ) corrected def2-TZVP (def2/J RIJCOSX auxiliary) basis in the gas phase on a truncated model of **6**. The calculations predict a CO stretching mode at 1998 cm^{-1}, and the P=N stretching mode at 1100 cm^{-1}, with roughly equal intensities (Figure S3). These are in excellent

agreement with the experimental IR stretching frequencies of 1945 cm^{-1} for a CO stretch and 1140 cm^{-1} for a P=N stretch (Figure S4. Further, the strong agreement between experimental and computational IR spectra allowed the identification of symmetric SO stretches (theory = 1162 cm^{-1}, experimental = 1182 cm^{-1}) and asymmetric SO stretches (theory = 1242 cm^{-1}, experimental = 1285 cm^{-1})."

* xyz 0 1

Table S45. Cartesian coordinates of 6.

Ru	8.647841000	9.201391000	18.036063000
P	7.806298000	9.301868000	16.000376000
S	5.676097000	10.482143000	14.512060000
N	7.297972000	7.184855000	19.986134000
N	7.141195000	6.488367000	17.978876000
O	6.606477000	11.197083000	18.884380000
C	7.593733000	7.544474000	18.705268000
C	7.184278000	6.325656000	16.563458000
C	8.326904000	7.321986000	22.168817000
O	5.403931000	11.903537000	14.611110000
C	7.539502000	7.909269000	21.189033000
C	6.841310000	10.278044000	13.194551000
N	6.354586000	10.012269000	15.867863000
C	6.695547000	5.942453000	20.043337000
H	6.386508000	5.504487000	20.976713000
O	4.528562000	9.649093000	14.213183000
C	6.718203000	9.215968000	12.312081000
H	5.874348000	8.544176000	12.394176000
C	7.917012000	11.155546000	13.109032000
H	8.001281000	11.981298000	13.805252000
C	8.512153000	7.972539000	23.379269000
H	9.132289000	7.516611000	24.142016000
C	10.663136000	8.240083000	18.615132000

C	8.877137000	10.961815000	12.129403000
H	9.719885000	11.639806000	12.060269000
C	6.592140000	5.505698000	18.777564000
H	6.182716000	4.599868000	18.364464000
C	7.366585000	10.385510000	18.577721000
C	10.557187000	10.029147000	17.162074000
C	10.493999000	9.418865000	19.397352000
C	8.762266000	9.897565000	11.242571000
C	8.299646000	5.754794000	15.972604000
C	7.683382000	9.029325000	11.331563000
H	7.591278000	8.201295000	10.638238000
C	6.065099000	6.650960000	15.810713000
C	6.909715000	9.120871000	21.421535000
C	10.719598000	8.609854000	17.236279000
C	7.114500000	9.775333000	22.626458000
H	6.627617000	10.726875000	22.805563000
C	10.389872000	10.521936000	18.492854000
C	7.916593000	9.205001000	23.605848000
H	8.068261000	9.716831000	24.549027000
C	6.075990000	6.418136000	14.443833000
H	5.206773000	6.677202000	13.852445000
C	8.308372000	5.538371000	14.603474000
H	9.183908000	5.103879000	14.135567000
C	10.731626000	10.846207000	15.930388000
H	10.330919000	10.337164000	15.051734000
H	11.797359000	11.024355000	15.752891000
H	10.237781000	11.814757000	16.015353000
C	11.071226000	7.747024000	16.076619000
H	11.071985000	6.691981000	16.344976000

H	12.072750000	7.998604000	15.713248000
H	10.376904000	7.887755000	15.243646000
C	7.197764000	5.870156000	13.838928000
H	7.206589000	5.699800000	12.768643000
C	10.607756000	9.541054000	20.875826000
H	9.828279000	10.175716000	21.299548000
H	11.574260000	9.985132000	21.138270000
H	10.547520000	8.570088000	21.362151000
C	10.879560000	6.868486000	19.151678000
H	10.366446000	6.724942000	20.102289000
H	11.947243000	6.697386000	19.321643000
H	10.528050000	6.104960000	18.458095000
C	10.321154000	11.954236000	18.894453000
H	9.865133000	12.567670000	18.117165000
H	11.329880000	12.339277000	19.076425000
H	9.747319000	12.080201000	19.813285000
H	5.201384000	7.088388000	16.295996000
H	9.146792000	5.480325000	16.586682000
H	6.244962000	9.534545000	20.677633000
H	8.795551000	6.363342000	21.978817000
H	9.516603000	9.746605000	10.478817000

Additional figures and discussions:

NMR spectra of compounds:

phosphanorcaradiene (2)

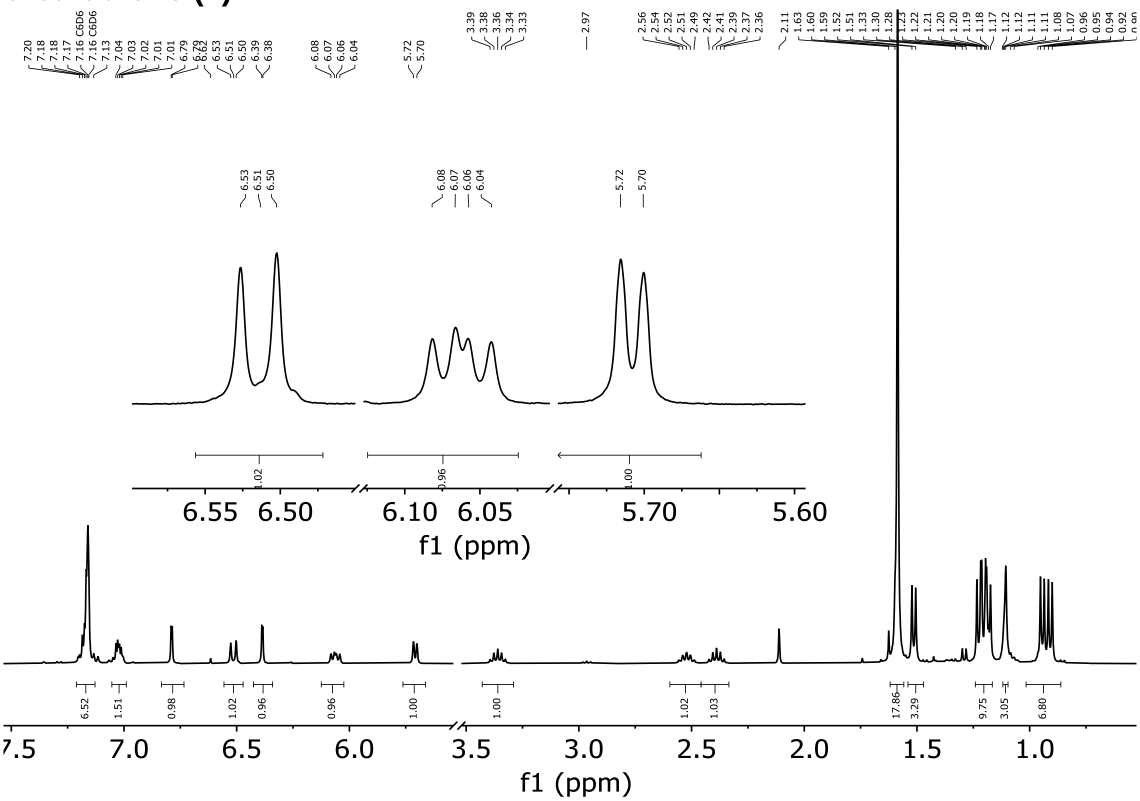

Figure S5. ^{1}H NMR Spectrum of ***2***

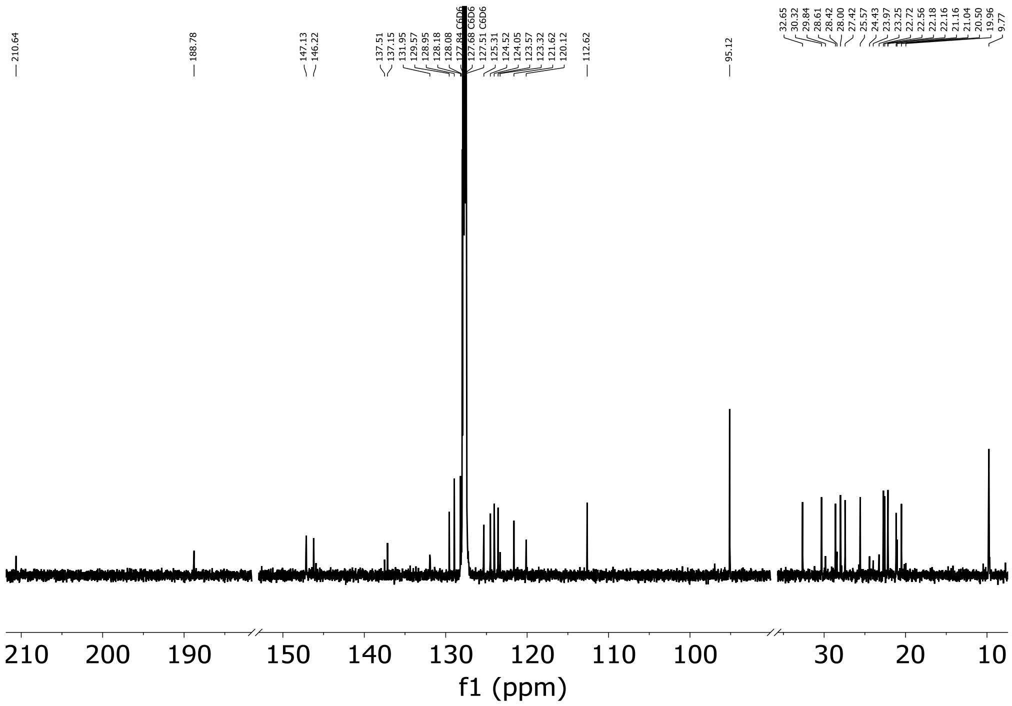

Figure S6. ^{13}C NMR Spectrum of ***2***

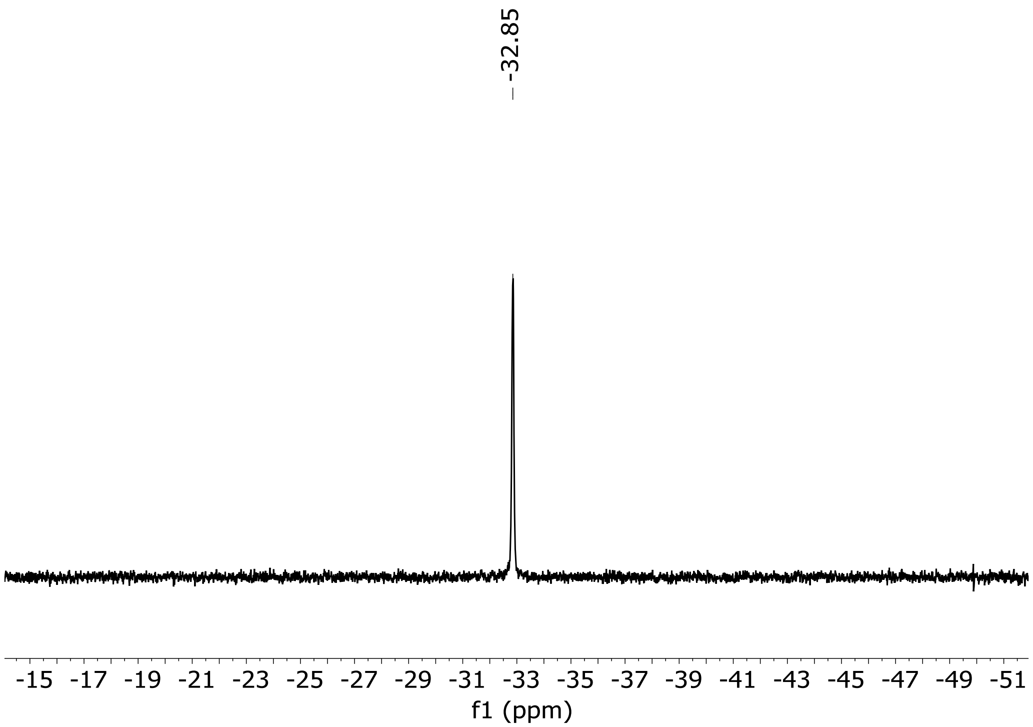

*Figure S7. ^{31}P NMR Spectrum of **2***

Phosphaazaallene (3)

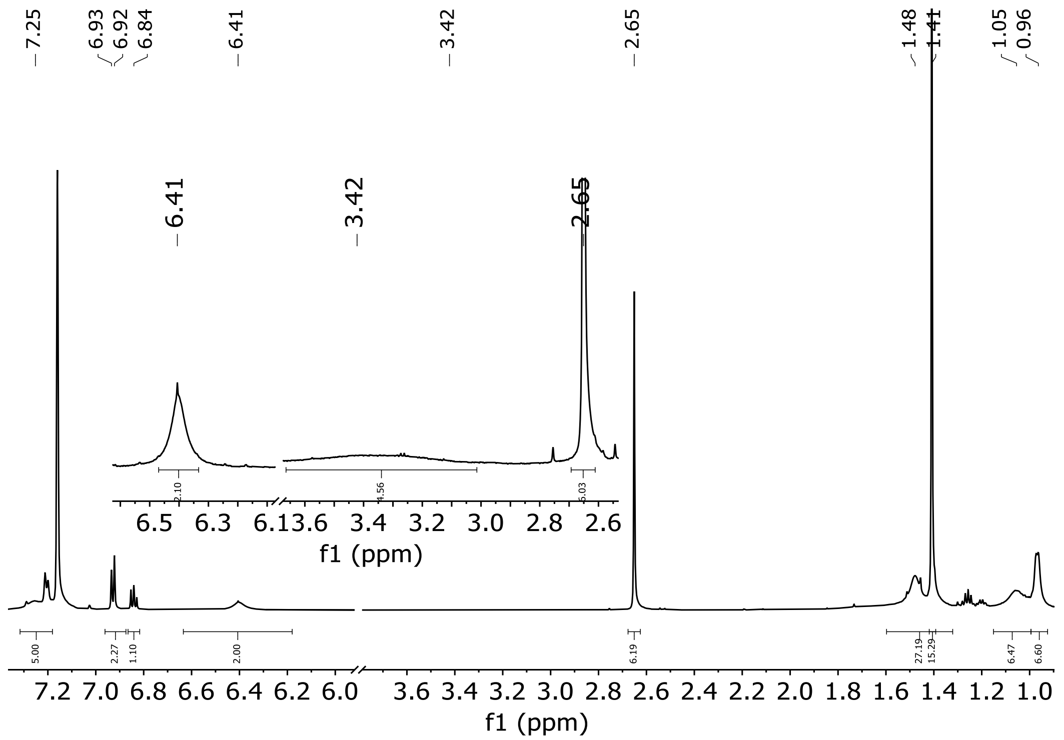

*Figure S8. ^{1}H NMR Spectrum of **3.**[*]*

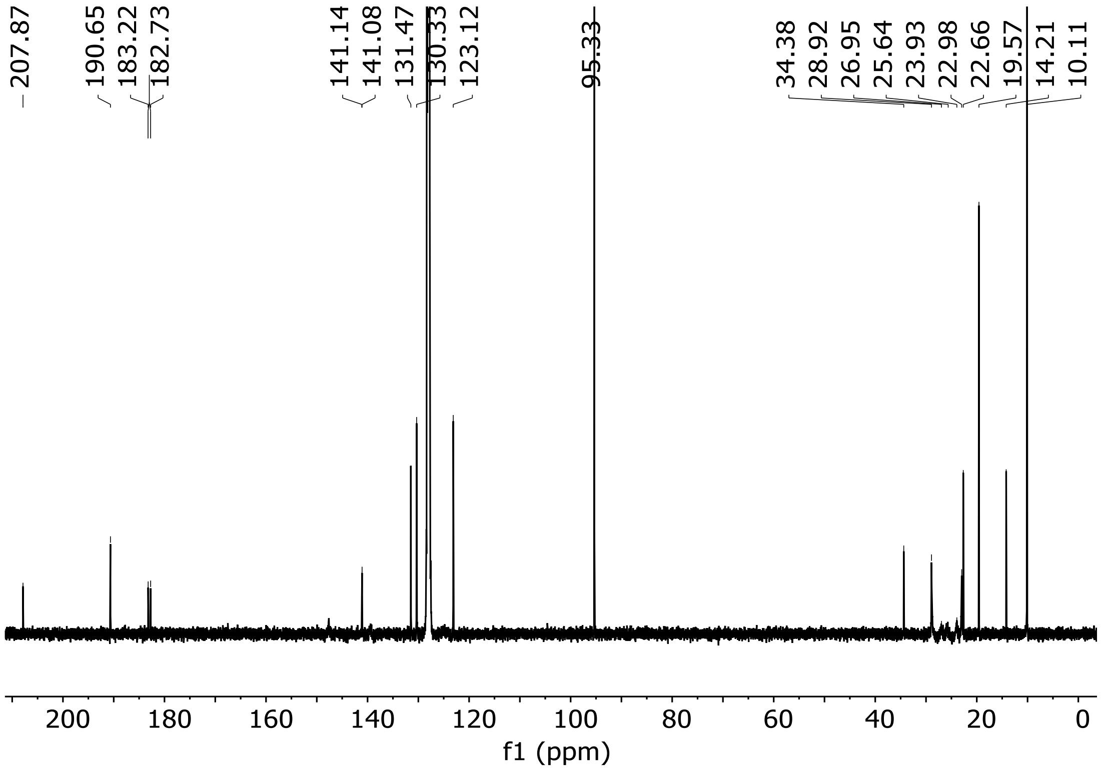

*Figure S9. ^{13}C NMR Spectrum of **3***

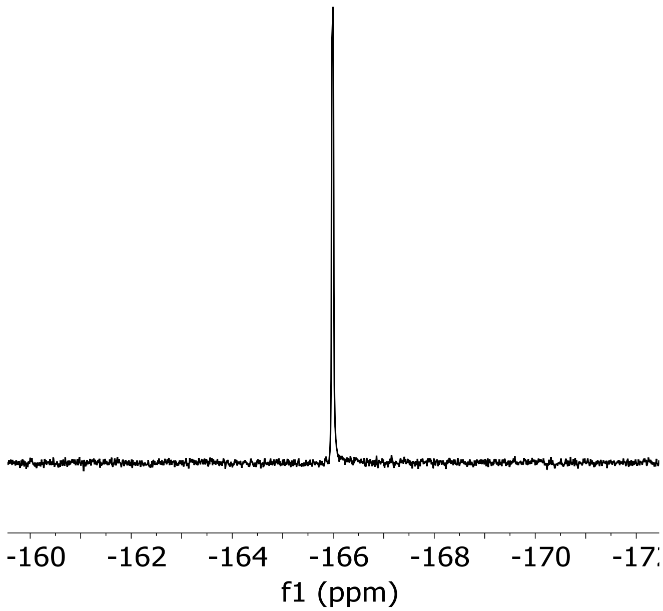

Figure S10. ^{31}P NMR Spectrum of ***3***

Ru-imidazol-2-yl (4) and DippP=(IMe$_4$) (5)

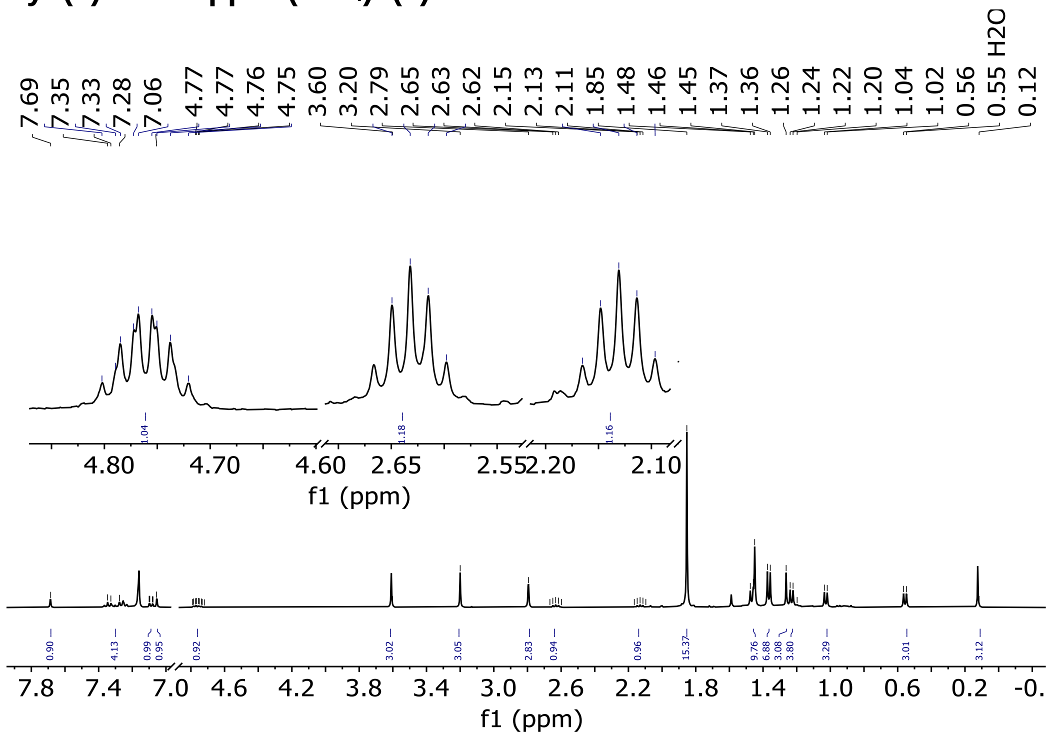

Figure S11. ^{1}H NMR Spectrum of ***4 + 5***

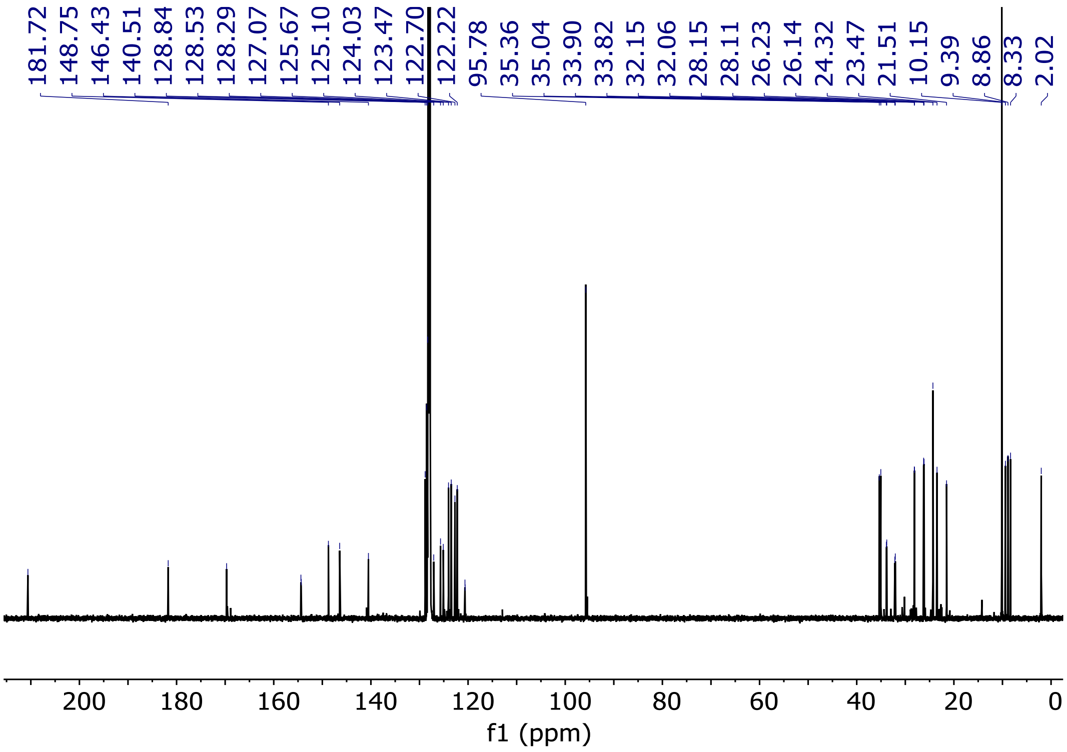

Figure S12. ^{13}C NMR Spectrum of ***4 + 5***

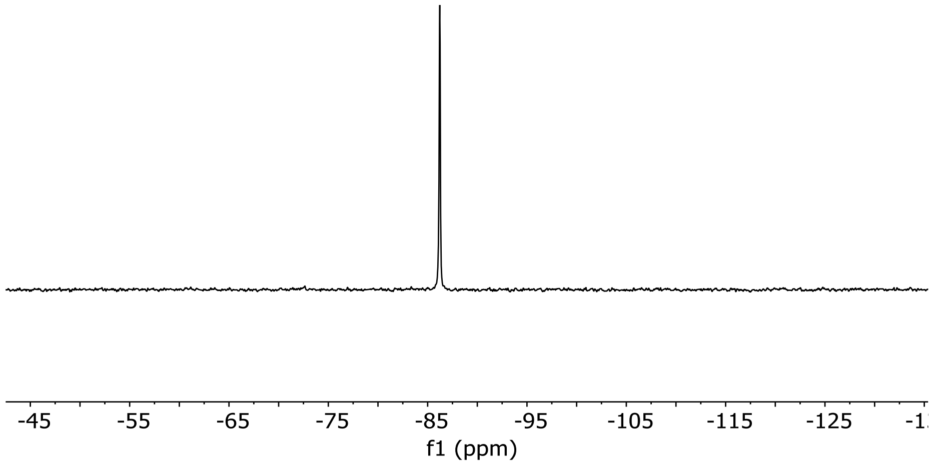

*Figure S13. ^{31}P NMR Spectrum of **4 + 5***

Iminophosphanide (6)

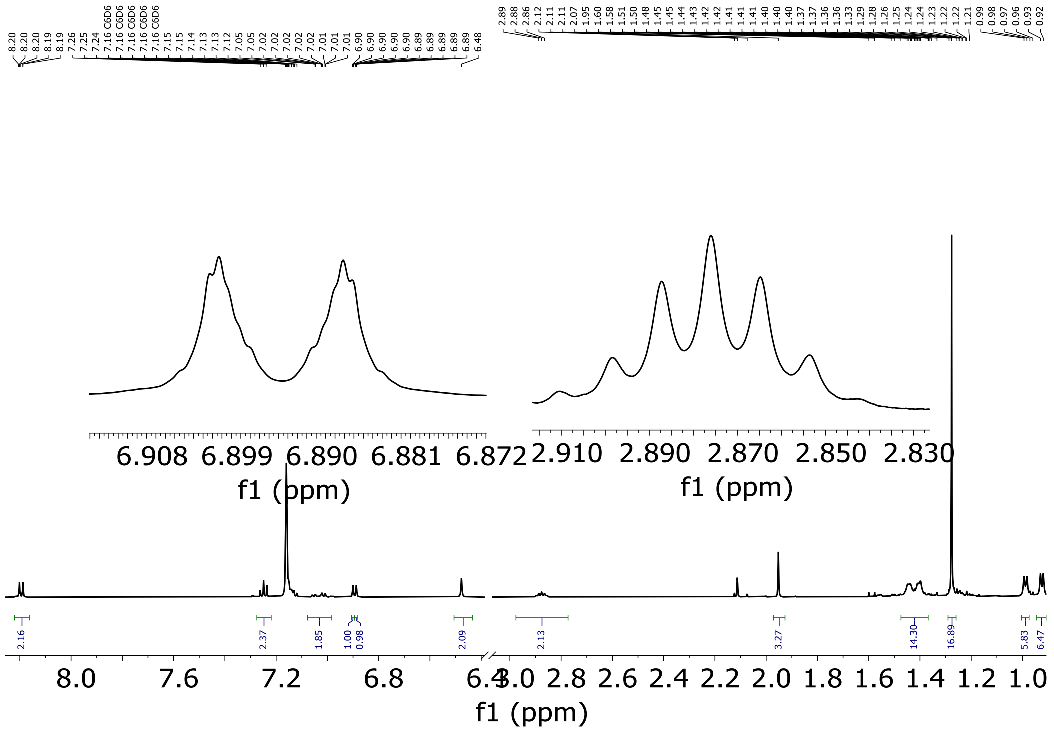

*Figure S14. ^{1}H NMR Spectrum of **6***

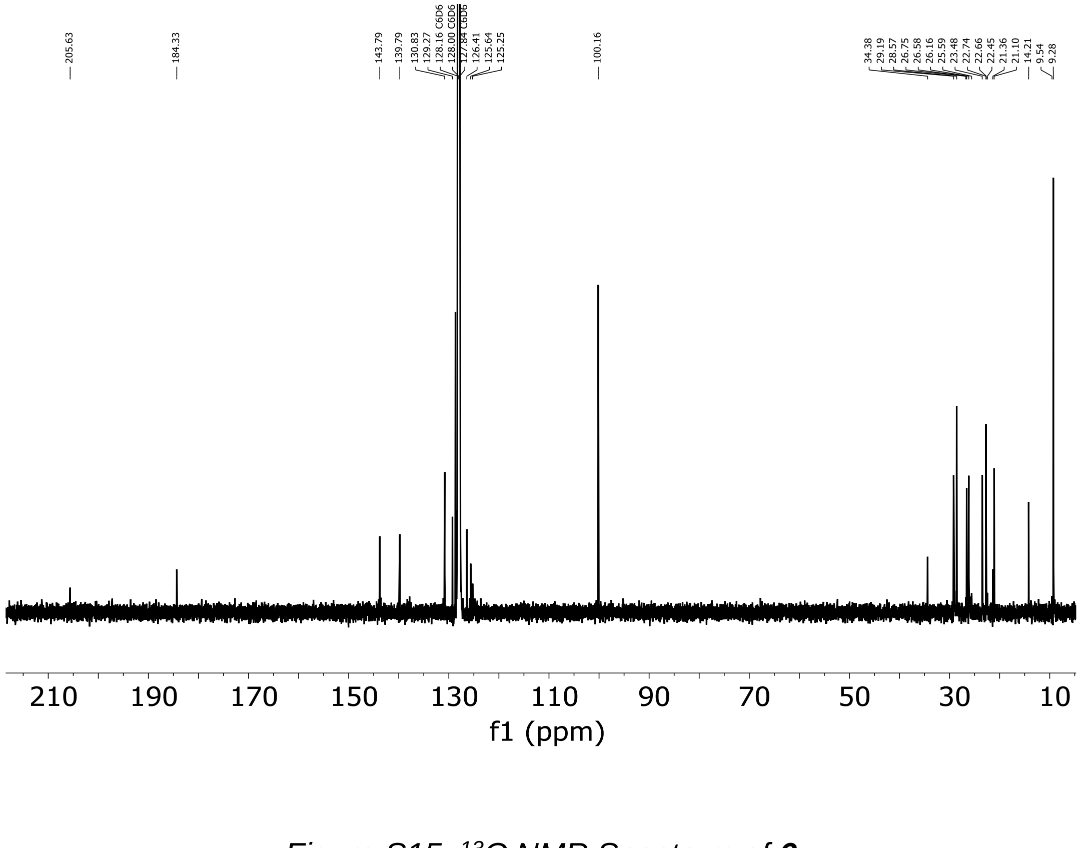

*Figure S15. ^{13}C NMR Spectrum of **6***

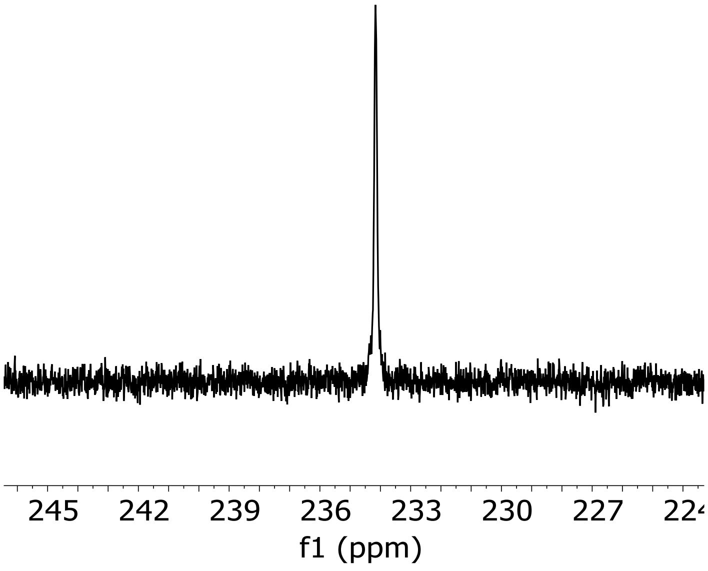

Figure S16. ^{31}P NMR Spectrum of ***6***

Phosphaformazan (7)

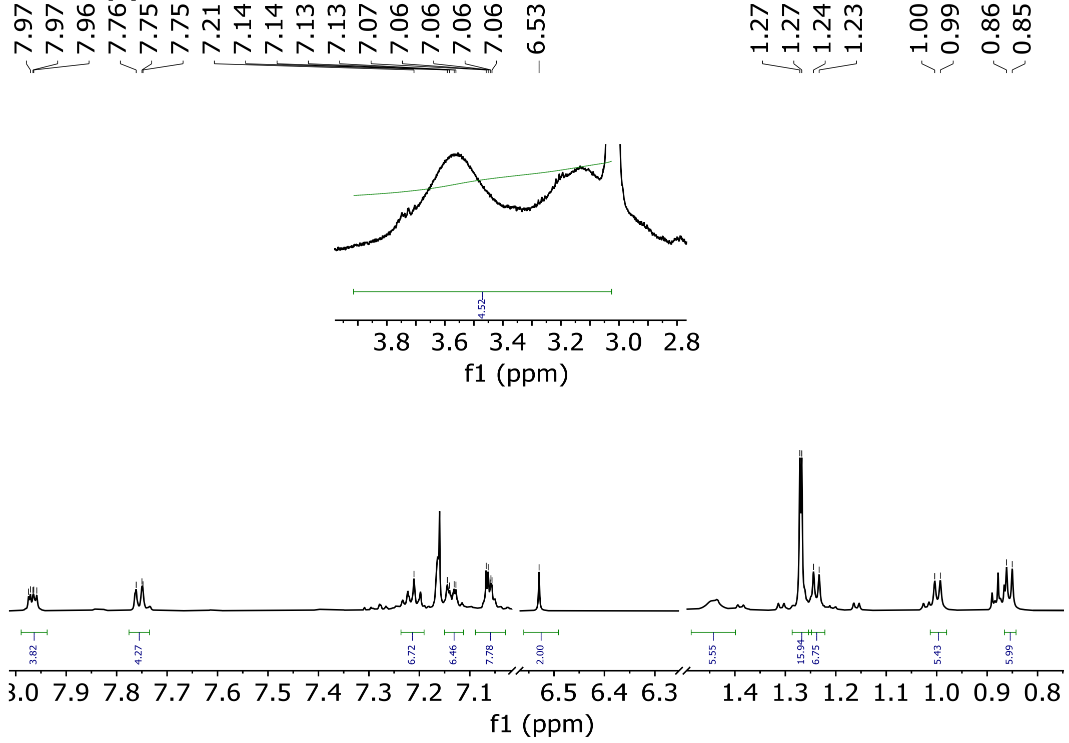

Figure S17. ^{1}H NMR Spectrum of ***7.*** **, ‡, †, ᴛ*

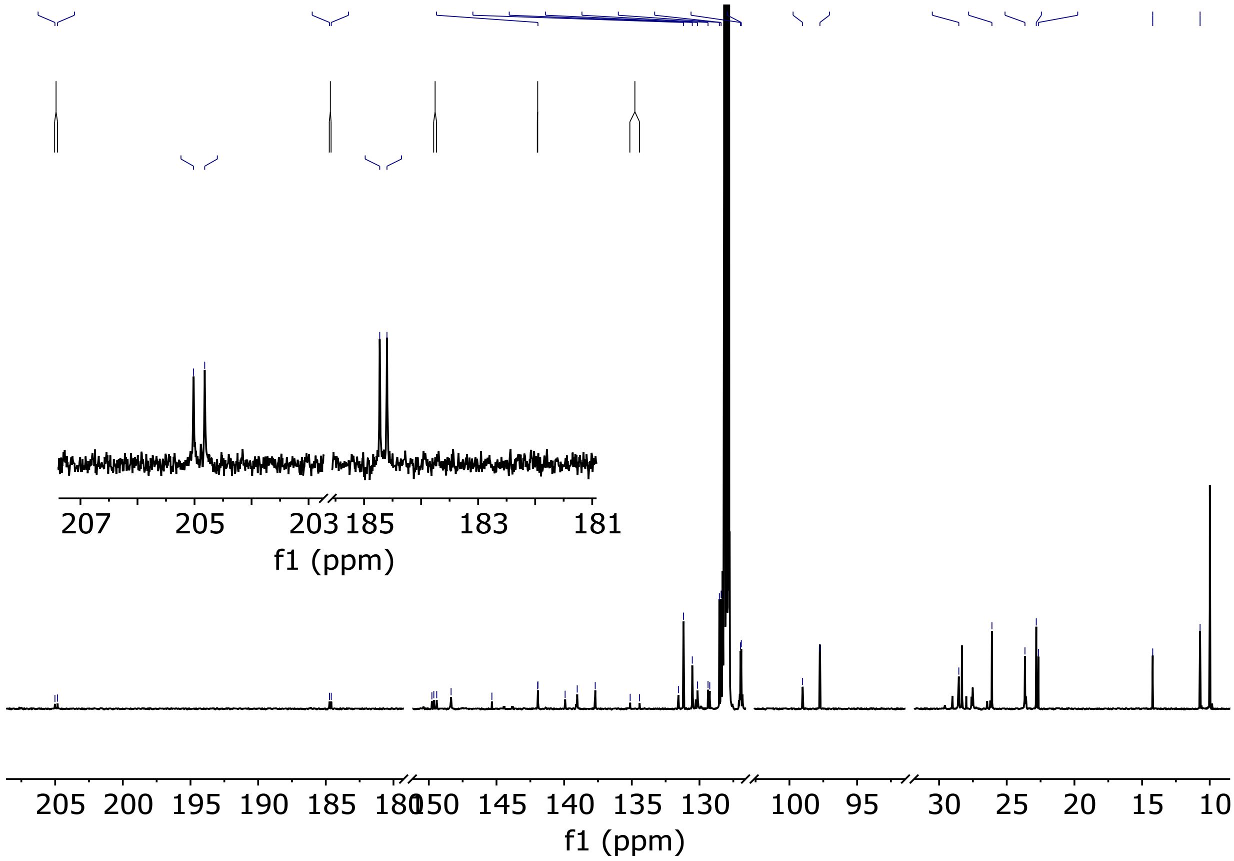

Figure S18. ^{13}C NMR Spectrum of ***7.*** *‡, †, ᴛ*

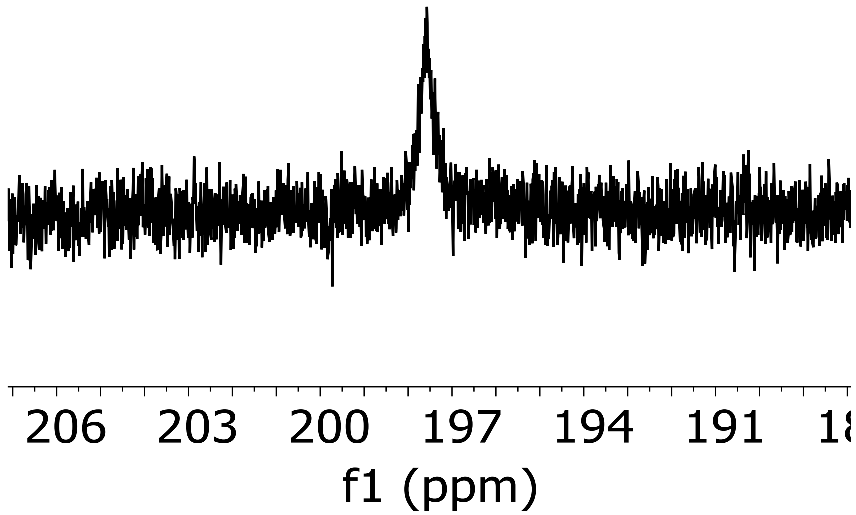

Figure S19. ^{31}P NMR Spectrum of ***7***

References:

(1) Huang, J.; Stevens, E. D.; Nolan, S. P.; Petersen, J. L. Olefin Metathesis-Active Ruthenium Complexes Bearing a Nucleophilic Carbene Ligand. *J. Am. Chem. Soc.* **1999**, *121* (12), 2674–2678. https://doi.org/10.1021/ja9831352.

(2) Puschmann, F. F.; Stein, D.; Heift, D.; Hendriksen, C.; Gal, Z. A.; Grützmacher, H.-F.; Grützmacher, H. Phosphination of Carbon Monoxide: A Simple Synthesis of Sodium Phosphaethynolate (NaOCP). *Angew. Chemie Int. Ed.* **2011**, *50* (36), 8420–8423. https://doi.org/https://doi.org/10.1002/anie.201102930.

(3) Smith, L. I.; Howard, K. L. Diphenyldiazaomethane. *Org. Synth.* **1944**, *24* (53). https://doi.org/10.15227/orgsyn.024.0053.

(4) Kuhn Thomas, N. K. Synthesis of Imidazol-2-Ylidenes by Reduction of Imidazole-2(3H)-Thiones. *Synthesis (Stuttg).* **1993**, *1993* (06), 561–562. https://doi.org/10.1055/s-1993-25902.

(5) Siewert, J.-E.; Schumann, A.; Hering-Junghans, C. Phosphine-Catalysed Reductive Coupling of Dihalophosphanes. *Dalt. Trans.* **2021**, *50* (42), 15111–15117. https://doi.org/10.1039/D1DT03095G.

Notes:

*The broad signals in the ^{1}H NMR spectrum are likely due to the presence of rotamers.

†Note that several aromatic resonances are obscured by the residual C_6D_6 solvent signal.

‡Residual pentane can be seen from purification by crystallization.

[T]Residual toluene can be seen from purification by crystallization.
